# Investigating ancient human DNA preservation on cave walls and in rock art

**DOI:** 10.1038/s41467-026-74234-2

**Published:** 2026-06-23

**Authors:** Alba Bossoms Mesa, Elena Essel, Louisa Jáuregui, Aurore Galtier, Elena I. Zavala, Kevin Nota, Merlin Szymanski, Julia Zorn, Hugo Gomes, George H. Nash, Pierluigi Rosina, Virginia Lattao, Luiz Oosterbeek, Carlos Carpetudo, Nelson A. Almeida, Carmen de las Heras, Pilar Fatás, Alfredo Prada, Lucía M. Díaz-González, M. Elena Sánchez-Moral, Alberto Martínez Villa, Mario Menéndez Fernández, José Julio García Arranz, Genevieve von Petzinger, Pedro Cantalejo, Luis-Efrén Fernández, José Ramos-Muñoz, Diego S. Fernández Sánchez, Hugo A. Mira, Emilio Muñoz Fernández, Ramón Montes-Barquín, Roberto Ontañón, Janet Kelso, Kay Prüfer, Benjamin Vernot, Mateja Hajdinjak, Qingfeng Shao, Sara Garcês, Hipólito Collado Giraldo, Matthias Meyer

**Affiliations:** 1 Max Planck Institute for Evolutionary Anthropology, Leipzig, Germany; 2 Department of Forensic Medicine, University of Copenhagen, Copenhagen, Denmark; 3 The Globe Institute, University of Copenhagen, Copenhagen, Denmark; 4 Polytechnic University of Tomar, Tomar, Portugal; 5 Geosciences Center, Earth and Memory Institute, Mação, Portugal; 6 Department of Archaeology, Classics & Egyptology, University of Liverpool, England, UK; 7 University of Coimbra, Coimbra, Portugal; 8 Museum of Prehistoric and Sacred Art of the Tagus Valley, Municipality of Mação, Mação, Portugal; 9 Municipality of Montemor-o-Novo, Montemor-o-Novo, Portugal; 10 Culture Unit, Alentejo Regional Coordination and Development Commission. Crato Extention, Crato, Portugal; 11 Museo Nacional y Centro de Investigación de Altamira, Santillana del Mar, Spain; 12 Departamento de Arte y Musicología, Universidad de Oviedo, Oviedo, Spain; 13 Universidad Nacional de Educación a Distancia, Madrid, Spain; 14 University of Extremadura, Cáceres, Spain; 15 LIACARA Project, Abu Dhabi, UAE; 16 Centre for the Exploration of the Deep Human Journey, Department of Anatomical Sciences, University of the Witwatersrand, Johannesburg, South Africa; 17 University of Cádiz, Cádiz, Spain; 18 Ardales and Rincón de Victoria Caves, Málaga, Spain; 19 Nerja Cave Research Institute, Málaga, Spain; 20 Complutense University of Madrid, Madrid, Spain; 21 Instituto de Estudios Campogibraltareños, Algeciras, Spain; 22 Colectivo para la Ampliación de Estudios de Arqueología Prehistórica, Santander, Spain; 23 Itinerario Cultural Europeo, PRAT-CARP, Spain; 24 Museo de Prehistoria y Arqueología de Cantabria, Santander, Spain; 25 Cuevas Prehistóricas de Cantabria, Gobierno de Cantabria, Cantabria, Spain; 26 Nanjing Normal University, Nanjing, China; 27 Junta de Extremadura, Mérida, Spain

**Keywords:** Evolutionary biology, Archaeology, DNA

## Abstract

Previous efforts to link Palaeolithic cultural records to specific populations through DNA analysis have focused on materials from archaeological floor deposits such as bones, sediments, and artefacts. In this study, we explore whether rock art, a spatially distinct expression of human activity, can also preserve DNA traces from its creators. We analyse DNA preservation in pigment samples collected in and around 24 rock art panels from 11 caves across Spain and Portugal, including simple marks (from nine sites), hand stencils (Maltravieso Cave, Extremadura, Spain), and figurative paintings (Cave of Altamira, Cantabria, Spain). We recover traces of ancient human mitochondrial and nuclear DNA, unaccompanied by faunal DNA, from a pigmented calcite crust at Escoural Cave (Portugal), as well as from an unpigmented cave wall sample from the same site. The absence of faunal DNA in both samples suggests direct DNA deposition through human contact. In contrast, three additional unpigmented samples, from Escoural and Covarón Cave (Asturias, Spain), yielded mixtures of human and faunal DNA, suggesting indirect deposition. Although our results do not conclusively link ancient human DNA preservation to the generation of cave art, we show that traces of human DNA can persist on cave walls for thousands of years.

## Abstract (web summary)

Ancient DNA can be preserved on materials from archaeological floor deposits such as bones, sediments and artifacts. Here, the authors investigate whether Palaeolithic wall art can preserve human DNA for study.

## Introduction

A major challenge in the study of human prehistory is linking cultural artefacts in the archaeological record to the human groups who created them. Ancient DNA research has helped bridge this gap through the analysis of human DNA from skeletal remains^1–3^, sediments^4,5^, and more recently, from artifacts themselves^6^. However, rock art, an important expression of human culture, remains beyond the reach of palaeogenetics, as it usually lacks direct association with excavated cave floors. As a result, ancient DNA analysis cannot currently contribute to debates about authorship, including whether Neandertals, in addition to early modern humans, created rock art^7–12^.

Given the various potential techniques used to apply pigments to cave walls^13^ and the ability of DNA to bind to mineral surfaces^4,14^, it is tempting to speculate that rock art might preserve DNA from its creators. For example, sprayed motifs, if created by blowing pigment directly from the mouth or by using hollow bones as an airbrush, would likely contain saliva, a rich source of DNA^15^. However, conclusive evidence of ancient DNA preservation in rock art is lacking. The only reported case involves the recovery of ungulate DNA, attributed to the use of organic binders, from pictographs in Texas nearly three decades ago using PCR-based methods^16^, though these results could not be replicated in subsequent studies^17^.

**Fig. 1:**
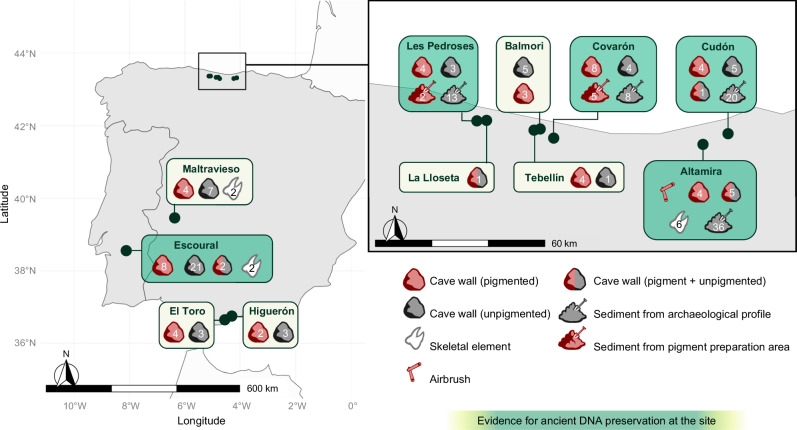
Archaeological sites and types of material analysed in this study. Icons indicate the types of material sampled at each site; numbers indicate how many samples were collected. Green shadings mark sites where ancient mammalian mtDNA was recovered from at least one sample.

Here we report on a systematic effort to investigate DNA preservation in and around rock art, using state-of-the-art sample preparation and sequencing methods. This work was carried out within the framework of the First Art project^18^, a multidisciplinary study focused on the dating and chemical analysis of pigment, which also provided the opportunity to collect samples for DNA analysis with minimal impact on the conservation integrity of the rock art. Samples were taken from 24 rock art panels distributed across 11 caves on the Iberian Peninsula (Fig. 1, Supplementary Figs. S1–S11, Supplementary Data S1, Supplementary Notes S1–S12) and included various types of motifs: linear traces, dots, triangles, amorphous stains, blown disks, large claviforms, negative hand stencils, and, importantly, pigment residues likely associated with more complex motifs that have not been preserved. Most of the motifs were created with red ochre (iron oxide) and belong to the non-figurative or “aniconic” tradition, thought to represent the earliest stage of Palaeolithic rock art^19–23^. We also collected comparative samples from unpigmented areas of the cave walls. Furthermore, we analysed two types of materials potentially associated with pigment application: (i) sediment samples from specific areas of cave floors where colouration patterns suggest pigment processing activity, and (ii) the diaphysis of a bird bone (ALT2617) from the Cave of Altamira, coated with red ochre on its interior. The latter likely served as an airbrush, i.e., a tool used to apply pigment by blowing it onto the cave wall^24^ (Supplementary Fig. S12).

## Results

### DNA preservation in sediments and bones from the rock art sites

To assess overall DNA preservation at the rock art sites, we extracted DNA from faunal skeletal remains and/or sediments collected at six sites where such material was available and performed library preparation and hybridization capture of mammalian mitochondrial (mt) DNA using a probe set targeting 242 mammalian species^25^. Ancient DNA was successfully recovered from skeletal remains at Altamira (4/6 positive) and Escoural (1/2) (Supplementary Data S2), but not in two samples from Maltravieso. Sediment samples from Covarón Cave (Asturias, Spain, 8/8), Cudón Cave (Cantabria, Spain, 6/20), and Les Pedroses Cave (Asturias, Spain, 11/13) yielded a rich record of ancient mammalian mtDNA (Supplementary Figs. S13–S15), including DNA from *Physeteridae* (sperm whale) at Les Pedroses, supporting previous evidence of marine mammal exploitation by regional hunter-gatherers^26^.

In addition, we performed hominin mtDNA capture on all sediment samples. For this analysis, we added a set of 36 sediment samples from Altamira, which were collected specifically for hominin DNA analysis and not screened for mammalian DNA. Modern human mtDNA was identified in the Upper Palaeolithic (UP) levels 4, 6, 7 and 8 of Altamira^27^, in UP levels L-1, L-2, L-3, L-4 and L-6 of Les Pedroses, and in the UP layers of Covarón^28^ (Supplementary Fig. S16, Supplementary Data S3). Neandertal mtDNA was recovered from the Mousterian levels 3 and 4 from sector L7 in Cudón^29^. Notably, all eight samples from Covarón yielded ancient human mtDNA, some in exceptional abundance. The richest sample produced 26,946 unique human mtDNA fragments (89-fold coverage of the mtDNA genome), with only minimal evidence of present-day human contamination (0.1% as estimated using AuthentiCT^30^, Supplementary Data S3).

At Covarón and Les Pedroses, we also collected sediment samples from putative pigment preparation areas located on or just below the cave floor (*n* = 5 and *n* = 2, Supplementary Notes S4 and S9, respectively). At Les Pedroses, these did not produce evidence for the presence of ancient human mtDNA (Supplementary Data S3). However, at Covarón, one sample was positive (SP.C.5568), yielding 682 deaminated mtDNA fragments. Nonetheless, since all regular sediment samples from Covarón contained ancient human DNA in even higher quantities, this finding does not indicate a specific association with pigment preparation.

### Ancient human mtDNA in rock art samples

**Fig. 2:**
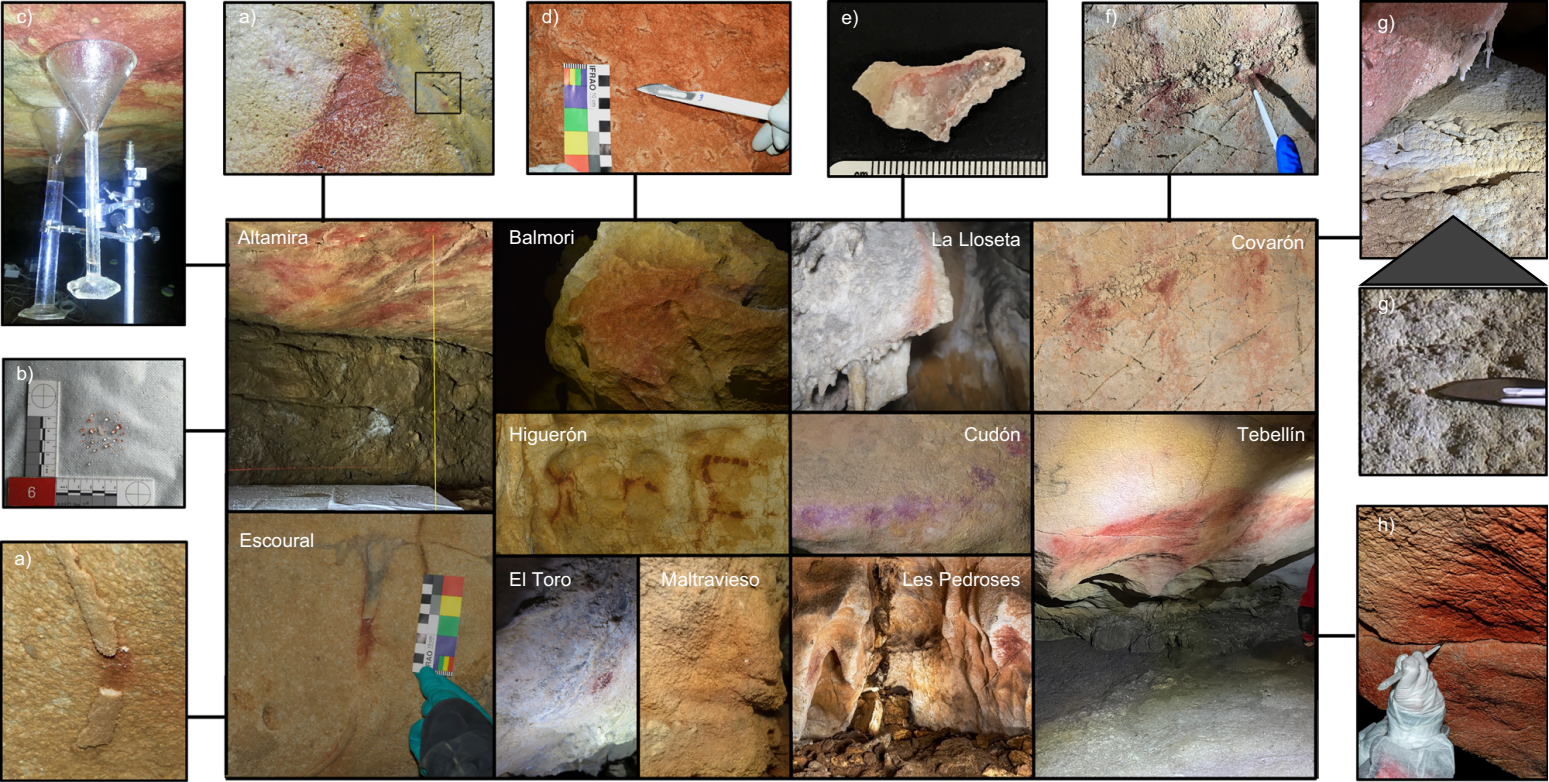
Palaeolithic rock art figures sampled in this study. The central images show one representative rock art figure from each of the 11 archaeological sites. Surrounding these, different methodological approaches for pigment collection are illustrated. **a** Scalpel collection of calcite, **b** Tyvek gauze collection, **c** Funnel collection, **d** Scalpel collection from a wall protuberance, **e** Crust sample collection for dating, **f** Removal of a calcite nodule with scalpel, **g** Scalpel collection from the ground, **h** Scalpel collection from a rock fissure.

Different types of pigment samples were utilized in this study, selected based on site-specific circumstances and opportunities (Fig. 2). Most samples were collected with scalpels, targeting pigment-containing calcite nodules or crusts (*n* = 37). At Tebellín (Spain), three samples were taken from a natural fissure inside a large claviform motif. At Maltravieso, samples were collected using a dentistry drill (*n* = 2) and sterile swabs (*n* = 2). At Covarón, four pigment-containing mineral flakes were collected from the ground beneath two figures. Finally, at Altamira, five pigment-containing flakes were recovered from a fabric placed under the polychrome ceiling to collect material naturally washed away by dripping water, and a pigment sample was collected from one of the funnels used to measure dripping water. In total, 54 samples were collected from 24 rock art panels. Subsequent subdivision yielded 64 subsamples for analysis (Supplementary Data S1).

DNA was extracted using a silica-based method developed for ancient bones and sediments but also effective for recovering DNA from other mineral surfaces^4^. To maximize DNA recovery, large or hard samples were first subjected to an initial extraction, then homogenized into finer powder and re-extracted. Extracts were converted into single-stranded libraries^31^ and enriched for hominin mitochondrial DNA using hybridization capture^4^.

**Fig. 3:**
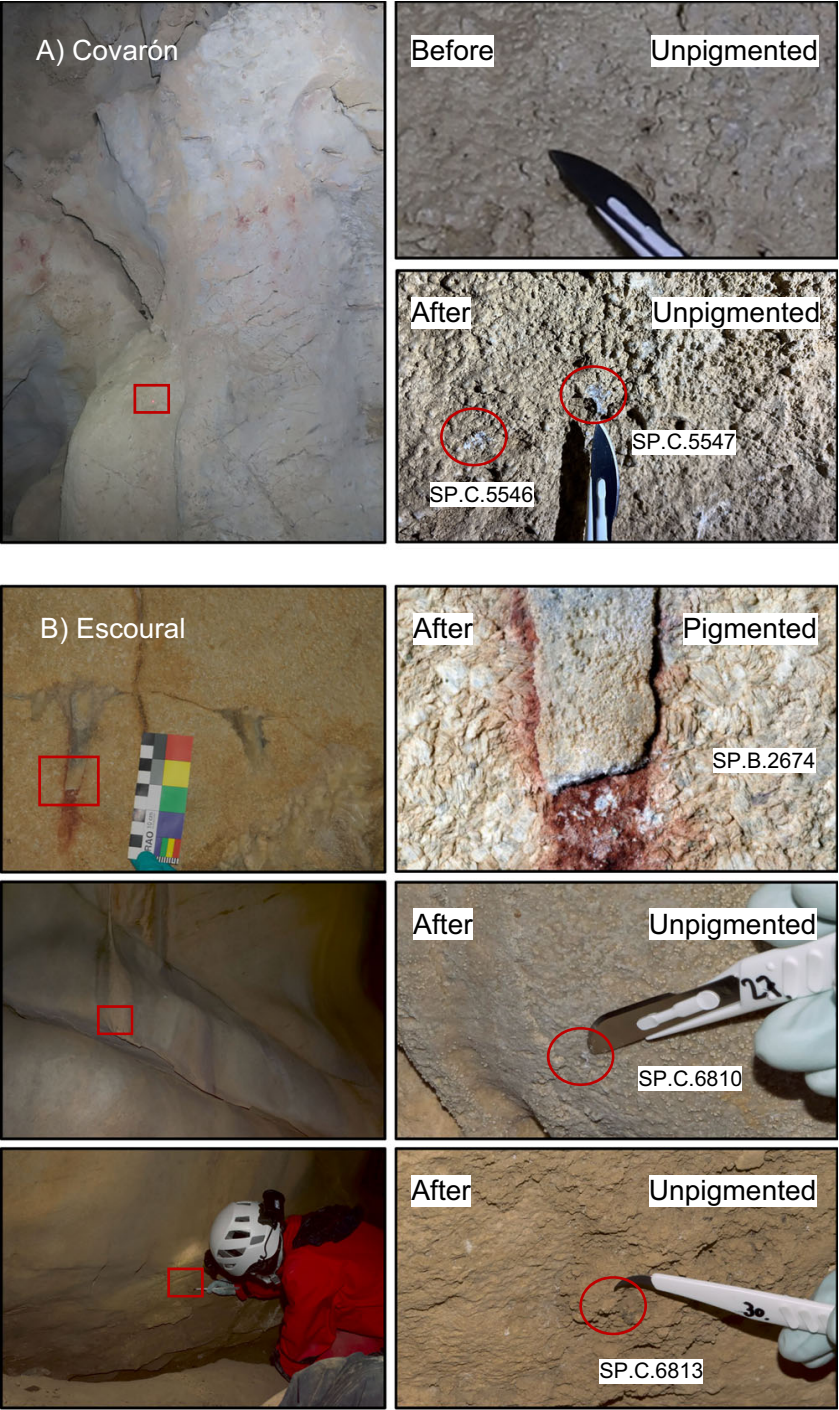
Cave wall samples (pigmented and unpigmented) yielding ancient human DNA. The images on the left provide a broader view of the sample context for **A** Covarón and **B** Escoural. The images on the right show close-up views taken before and/or after sampling.

Among all pigment-containing samples, only one – SP.B.2674, a sample taken from a pigmented calcite crust fragment from Panel 11 at Escoural (Fig. 3) – yielded hominin mtDNA sequences with significantly elevated frequencies of C-to-T substitutions at their ends, indicative of the presence of authentic ancient DNA^32^. These patterns were observed in two extracts prepared from the sample, one pre- and one post-homogenization, containing 526 and 436 hominin mtDNA molecules, respectively (Supplementary Data S2). C-to-T substitution frequencies were 20.6% (95% binomial confidence interval: 13.4–29.5%) and 28.0% (19.5–37.9%) at the 5’ end, and 10.5% (5.6–17.7%) and 15.5% (8.9–24.2%) on the 3’ end, for the two extracts, respectively (Supplementary Fig. S17), and present-day human contamination was estimated at 54.8% and 32.4% (Supplementary Data S3). A conditional substitution test, which estimates deamination in authentic ancient molecules from contaminated samples^33^, revealed C-to-T substitution frequencies of 30.8% (9.1–61.4%) at the 5’ end and 26.7% (7.8–55.1%) at the 3’ end of the molecules from both extracts, consistent with the presence of a population of highly deaminated hominin mtDNA fragments.

**Fig. 4:**
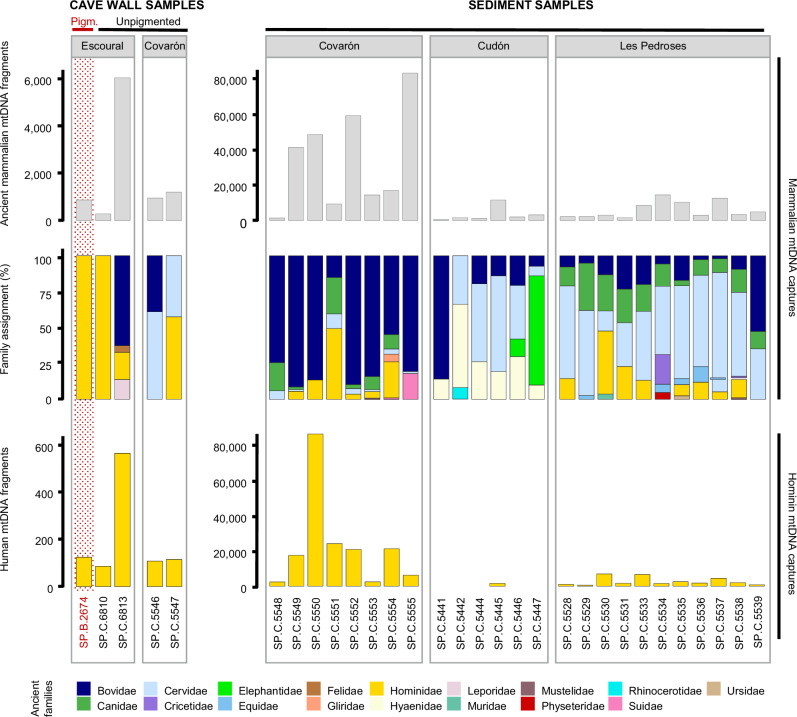
Comparison of mammalian and human mtDNA recovery in sediment and cave wall samples. Shown are samples for which both mammalian (top and middle) and hominin mtDNA captures (bottom) were performed. These include sediment samples positive in mammalian mtDNA capture as well as pigmented or unpigmented cave wall samples positive in hominin mtDNA capture. Human mtDNA fragments are shown without deamination filtering, but only for samples showing evidence of ancient human DNA.

Enrichment of the libraries from the two extracts containing ancient hominin DNA using mammalian mtDNA probes revealed no evidence of ancient faunal DNA (Fig. 4). Further analysis of hominin mtDNA molecules overlapping diagnostic positions that distinguish modern from archaic humans showed exclusive support for the modern human-specific state, even after restricting to the 59 and 64 putatively deaminated fragments from the two extracts to minimize contamination (Supplementary Data S3). We therefore conclude that ancient DNA from one or more modern humans, but no faunal DNA, is present in the sample from Panel 11 at Escoural.

Following this initial positive result from Panel 11, additional subsamples were analysed from the pigmented calcite crust and an adjacent pigmented crust below. One crust fragment was also embedded in resin^34^ to allow targeted sampling of pigmented and unpigmented areas (Supplementary Fig. S18), resulting in a total of 17 subsamples analysed from Panel 11. However, no further ancient hominin DNA was recovered (Supplementary Data S3), suggesting that the initial human DNA signal was confined to a small area rather than being distributed throughout the pigmented crusts.

### Ancient human and faunal mtDNA in unpigmented cave wall samples

To assess whether ancient DNA can be recovered from cave walls outside of pigmented areas, we also analysed unpigmented samples – mostly collected near pigmented motifs – from bare cave walls (*n* = 50), pigment-free mineral flakes at Covarón (*n* = 2), and unpigmented samples from pigment-containing crusts (*n* = 4). Further subdivision of these samples, along with the inclusion of unpigmented tyvek gauze pieces from Altamira (*n* = 5), increased the total number of unpigmented subsamples to 66.

Significant evidence of ancient hominin mtDNA, all of modern human type, was found in four unpigmented cave wall samples (Figs. 3 and 4, Supplementary Fig. S17; Supplementary Data S3). Two (SP.C.6810 and SP.C.6813) were collected near Panel 64 at Escoural; the others (SP.C.5546 and SP.C.5547) were from Covarón, also in the proximity of a pigmented area. For the latter two, a second extraction after homogenizing recovered no additional ancient human mtDNA molecules with statistically significant deamination, suggesting most DNA had been released during the first extraction (Supplementary Data S3). The number of molecules retrieved from the four unpigmented samples ranged from 351 to 1980 (a mean of 1002), with contamination estimates between 0.1 and 67.6% (Supplementary Data S3).

Unlike the pigmented sample from Escoural Panel 11, which showed no detectable faunal DNA, targeted enrichment of mammalian mtDNA recovered ancient faunal DNA in one of the two unpigmented samples from Escoural (SP.C.6813) and both samples from Covarón (Fig. 4). In all but one case (SP.C.5547 from Covarón), the number of faunal mtDNA fragments – originating from felids, leporids, and large herbivores such as bovids and cervids – exceeded those of human mtDNA. The co-occurrence of faunal and human DNA in three of the samples suggests possible contamination with cave floor sediments. Sediments typically contain a broad range of faunal taxa, irrespective of whether human DNA is present^4,5,35,36^, a pattern also observed in sediment data from Covarón. Notably, the Covarón sediment samples showed remarkably good preservation of ancient human DNA, further supporting the possibility that the DNA on the wall was introduced via sediment contamination.

### A metagenomic analysis of rock art and unpigmented wall samples

Non-human ancient DNA may occur in rock art samples due to the use of diverse organic substances in pigment preparation, including animal fat and other plant- or animal-derived binders^16,37^. To investigate this, we generated between 94,192 and 13,280,964 shotgun sequences from most of the DNA libraries prepared from both pigmented and unpigmented cave walls samples, as well as from sediment samples for comparison (Supplementary Data S4, Supplementary Figs. S19–S21). Among mammals, the shotgun data were dominated by various bat families (*Vespertilionidae, Rhinolophidae, and Miniopteridae*) whereas other taxa – *Bovidae, Cricetidae, Felidae*, *Muridae*, and *Mustelidae* – were detected in only seven cave wall samples, all of them unpigmented. Notably, only one of the four unpigmented samples that contained both ancient human and faunal DNA in mtDNA capture also yielded faunal DNA in the shotgun analysis, highlighting the greater sensitivity of targeted mtDNA enrichment for detecting low-level mammalian DNA. Plant, vertebrate and invertebrate taxa identified through metagenomic analyses appeared sporadic or cave-specific, with no consistent enrichment in pigment-containing samples (Supplementary Figs. S19, S20).

### Ancient human nuclear DNA from cave walls

To further characterize the human DNA in both the rock art and unpigmented control samples, we enriched the libraries containing ancient human mtDNA for ~1.35 million ancestry-informative single nucleotide polymorphisms (SNPs) in the nuclear genome^38^. The two libraries from the Escoural rock art sample exhibited terminal C-to-T substitution frequencies below 10% after nuclear DNA enrichment, and too few SNPs (180 and 379, respectively) were covered by deaminated fragments to enable population genetic analyses (Supplementary Data S5). In contrast, C-to-T substitution frequencies were higher in the unpigmented samples from Escoural and Covarón, ranging from 30.7 to 39.6% at the 5’ and from 21.4 to 28.4% at the 3’ end. In the Covarón samples, 5949 and 7819 SNPs were covered by deaminated fragments, and in the Escoural samples, 1223 and 3772 SNPs were covered, respectively.

**Fig. 5:**
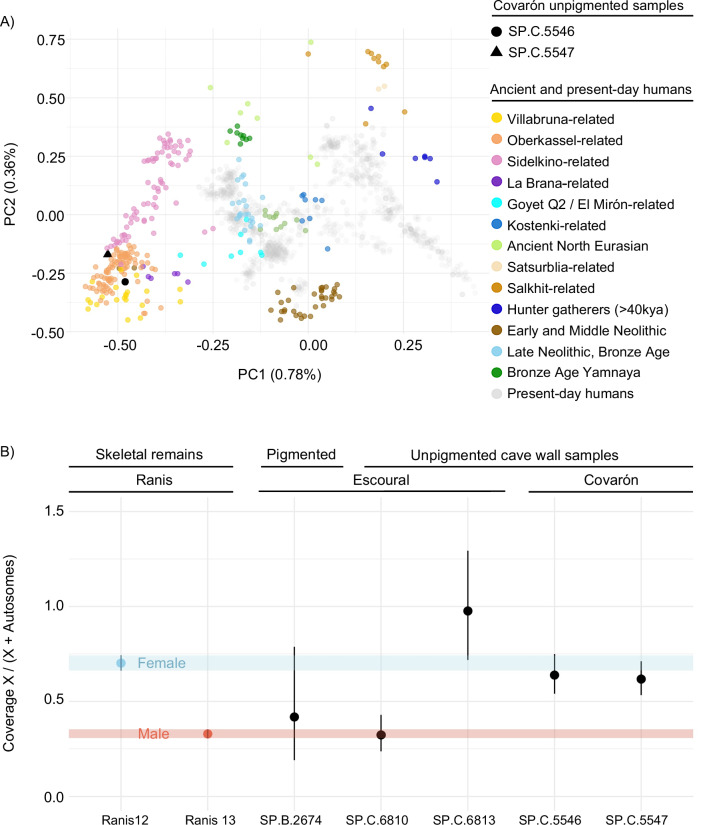
Human nuclear DNA recovered from the pigmented and unpigmented cave wall samples. **A** Cave wall samples projected into a Principal Component Analysis (PCA) including present-day Eurasian (grey) and ancient genomes (coloured by genetic cluster^40^). The Covarón samples (SP.C.5546 and SP.C.5547) are highlighted in black. **B** Sexing. X-to-autosome coverage ratios calculated using deaminated sequences only. Reference values from previously sequenced individuals^80^ of known sex (Ranis13, male; Ranis12, female) are included. Circles show mean estimates of the X/(X + Autosomes) coverage ratio for each individual (number of single-nucleotide polymorphisms = 41,952; 61,828; 559; 5975; 7818; 3772; 1223; as ordered on the x-axis); error bars represent 95% binomial confidence intervals. Source data are provided as a Source Data file.

In a principal component analysis (PCA) together with data from 315 prehistoric and 1187 present-day Eurasian individuals, the two unpigmented samples from Covarón clustered within the variation of western hunter-gatherers (WHG), aligning closely with the ‘Villabruna’ and ‘Oberkassel’ genetic clusters, which are comprised of individuals dating from ~16,700 to 5200 cal. BP^39,40^ (Fig. 5A, Supplementary Fig. S22). The two unpigmented samples from Escoural did not show any clear affinities to ancient or present-day humans given the limited data available (Supplementary Fig. S22). Coverage ratios between autosomes and sex chromosomes indicate that the human DNA in the three unpigmented samples containing mixtures of faunal and human DNA (SP.C.5546 and SP.C.5547 from Covarón, SP.C.6813 from Escoural) is predominantly female-derived (Fig. 5B). In contrast, SP.C.6810 from Escoural, the unpigmented sample without faunal DNA, shows a coverage ratio consistent with a predominantly male origin. The Escoural rock art sample did not yield sufficient data for sex determination.

### Temporal constraints on ancient human DNA recovered from cave walls

In the absence of direct chronological information, we attempted to constrain the potential age of the human DNA recovered from the pigmented and unpigmented cave wall samples by combining evidence from patterns of DNA damage, mitochondrial haplogroup analysis, and site-specific archaeological context.

Previous studies have reported correlations between sample age and cytosine deamination in ancient DNA^5,41,42^, though with substantial variation, especially across samples with different thermal histories. A comparison of inferred deamination rates in single-stranded overhangs estimated using mapDamage 2.0^43^ between our cave wall samples and a dataset of 185 published ancient DNA samples^42^ suggests a minimum age of ~1000 years for one of the samples from Covarón, and ~2000 years for all other samples, while remaining consistent with much greater ages (Supplementary Fig. S23). We also compared deamination patterns across sample types within our study. At Covarón, where DNA was also retrieved from sediments associated with the Magdalenian (~14.5−11.5 ka BP in Cantabria), deamination rates between the cave wall and sediment samples are broadly comparable (Supplementary Figs. S24–S26). At Escoural, the only possible comparison is to a bovid bone derived from a Middle Palaeolithic deposit, which, based on a uranium-thorium date of a horse tooth, may have formed around 49 ka BP^44^. As far as can be inferred given the large confidence intervals, deamination signals in the human mtDNA from the cave wall samples are approximately half those observed in the bovid bone (Supplementary Fig. S24), suggesting an Upper Palaeolithic or later origin.

To obtain additional age constraints, we attempted mitochondrial haplogroup calling using mixEMT^45^. However, coverage was too low to yield conclusive results with this approach. We therefore directly examined putatively deaminated DNA fragments overlapping haplogroup-defining positions in the mitochondrial genome (Supplementary Note S13). For the pigmented sample from Escoural, analysis of deaminated fragments could not resolve the haplogroup beyond haplogroup N and its many subclades, providing no additional constraint on the possible age of the DNA. Of the remaining two cave wall samples from Escoural, one (SP.D.6810) contained insufficient data for haplogroup assessment. The other (SP.D.6813) yielded ambiguous results supporting both R0 and U or their subclades, suggesting that the DNA was not deposited by direct contact from a single human individual, as indicated also by the presence of faunal DNA in the sample. The two cave wall samples from Covarón both support assignments to haplogroup U5a’b, the most abundant haplogroup in European hunter-gatherers^46^, consistent with their assignment to Western hunter-gatherers based on nuclear DNA analysis.

Finally, site history provides another constraint on the minimum age of the human DNA recovered from the cave walls of Escoural, indicating it is at least 4000–5000 years old. The site was accessible only until the Chalcolithic period, after which it remained sealed and was not reopened until 1963 as the result of quarrying activities^47^. In addition to Chalcolithic deposits, Escoural contains Neolithic burials and Upper and Middle Palaeolithic archaeological remains, reflecting a long history of human use^48,49^. Stylistically, the pigmented calcite crust that yielded ancient human DNA is consistent with motifs observed in Palaeolithic contexts^19^, although similar motifs were also produced as late as the Neolithic^50^. At Covarón, archaeological excavations have revealed a wide range of human occupational contexts, including evidence from the Lower Magdalenian, as well as other archaeological remains documented in various areas of the cave corresponding to the Azilian period and even to recent prehistory^51^. Black figures painted in the inner gallery have been attributed to the Magdalenian, whereas the wall samples containing human DNA were taken from the main hall, near the red stains forming panel 1. As in the case of Escoural, those aniconic motifs are common in Palaeolithic art, but a more recent origin cannot be ruled out.

### DNA preservation in the airbrush

Unlike pigments from rock art, where the application methods remain uncertain, the Altamira airbrush was likely used to blow pigment, making the presence of saliva, and thus the potential for ancient human DNA preservation, more likely. To protect this rare artefact, less than 1 mg of pigment was collected from inside the bone in six samples using a dentistry drill without rotation. Although we recovered between 1050 and 17,122 human mtDNA fragments, no significant deamination signals were observed, including in the conditional substitution test (Supplementary Fig. S27). This indicates either the absence of ancient human DNA or that it is present at levels too low to be distinguished from contaminant DNA. Similarly, hybridization capture with mammalian mtDNA probes did not recover ancient faunal DNA (Supplementary Data S2).

## Discussion

In conclusion, our study expands the known sources of ancient human DNA in caves – previously limited to skeletal remains, osseous artefacts, and sediments – to include cave walls. The human DNA detected in the cave wall samples likely derives from multiple depositional processes. Two cave wall samples from Escoural (one pigmented and one unpigmented) contained ancient human but no detectable faunal DNA. This contrasts with findings from multiple studies^4,5,35,36,52^, which have shown that when human DNA is present in sediments, it is almost always mixed with faunal DNA, indicating that other mammalian species generally contribute more DNA to the environment than humans. Similarly, ancient faunal, but not human DNA, is also the only type of mammalian DNA recovered from stalagmites, which have been shown to incorporate environmental DNA during mineral crust formation^53^. The exclusive presence of human DNA among the mammalian DNA identified in the two Escoural cave wall samples therefore points to direct human contact (such as touching, rubbing, or deposition of bodily fluids) as the most likely source of DNA deposition on the cave wall. In contrast, in three additional unpigmented cave wall samples, two from Covarón and one from Escoural, human DNA co-occurred with faunal DNA, consistent with indirect deposition. This may have occurred via sediment transfer (for example through people touching the ground and subsequently the cave walls as they moved through the cave) or through other mechanisms such as movement of DNA in percolating water^53^.

In principle, the human DNA recovered from the Escoural rock art could be directly or indirectly associated with the individual(s) involved in its creation, for example through pigment preparation or application. However, in the absence of precise chronological constraints on the deposition of both the pigment and the DNA, it remains possible that their deposition occurred thousands or tens of thousands of years apart. Furthermore, the detection of human, and no faunal, DNA in an unpigmented sample highlights that human contact unrelated to artistic production can also contribute to the deposition of ancient DNA on cave walls.

Establishing a direct link between DNA and art production would require evidence that the DNA in the pigment sample consistently differs from that in unpigmented areas, whether in its abundance, the likelihood of successful recovery, or its population genetic affinities to past human groups. Alternatively, when pigment is trapped beneath carbonate crusts, the pigmented areas as well as overlying and potentially underlying unpigmented parts can be sampled and analysed separately to determine whether DNA recovery is consistently restricted to the pigmented portions, as we have attempted here for the Escoural crust. This approach is also commonly used in uranium-thorium dating of rock art^9,20,54^, which can in principle be combined with DNA analysis. If sufficient human DNA from a single individual were obtained in future studies, genetic dating may also become possible, as has been demonstrated for DNA recovered from sediments^5,35^.

With ancient human DNA obtained from only one of 24 rock art panels sampled, despite generally favourable preservation conditions at many rock art sites, our findings suggest that pigmented surfaces themselves rarely retain enough DNA to remain detectable after thousands of years. The absence of ancient DNA in the Altamira airbrush further supports this notion. We also detected no faunal or other non-human DNA that could be clearly linked to the use of organic binders. Nevertheless, given the diversity of rock art techniques and contexts, it remains possible that rock art from other sites, periods, or styles could more consistently yield ancient DNA. For example, it would be desirable to test DNA preservation in additional samples from figurative rock art, or in hand stencils from sites with better DNA preservation than Maltravieso. As demonstrated here, systematic sampling of both the artwork and adjacent surfaces, along with sensitive methods for faunal DNA detection, should be incorporated into future research to help distinguish DNA related to art production from that introduced by other processes. It should also be investigated whether DNA extraction protocols can be improved specifically for cave wall surfaces.

The notion that ancient human DNA can be recovered from cave walls opens new possibilities for research beyond the study of rock art, even in caves that do not contain art. Analyses of cave wall samples could reveal which populations occupied caves without the need for floor excavations, how deeply they ventured, and whether activities were spatially concentrated or linked to individuals of a particular sex. This approach complements genetic research on sediments, skeletal remains, and artefacts, with the potential to reveal aspects of past human presence and behaviour that other sources of DNA cannot elucidate.

## Methods

### Sampling of bones, sediments and pigment preparation areas

The provenance of the samples and the permits obtained for sampling are described in Supplementary Notes S1–S12. Faunal skeletal remains from Altamira, Escoural and Maltravieso were sampled in the cleanroom facilities of the Max Planck Institute for Evolutionary Anthropology (MPI-EVA) in Leipzig, Germany. A sterile dentistry drill, set to the lowest speed, was used to remove a thin surface layer from the selected sampling areas. A fresh drill bit was then used to collect sample powder from the interior of the specimens. Supplementary Data S1 provides an overview of the types of skeletal remains sampled, their archaeological contexts, and the amount of powder obtained.

Sediment samples were collected from exposed profiles of Altamira, Cudón, Les Pedroses and Covarón. To prevent cross-contamination from falling material, sampling was performed from the bottom-up in vertical columns. After removing 1–2 cm of surface sediment with sterile, single-use scalpels, 5 mL screw-cap tubes were pressed into the cleaned profile to collect sediment samples. To minimize contamination with modern DNA, precautions such as wearing face masks and gloves were implemented. In the case of Altamira, personal protective equipment was also employed, following the internal access protocols established by the Plan de Conservación y Régimen de Acceso a la Cueva de Altamira^55^. The stratigraphic locations of all samples were recorded (Supplementary Notes S1, S3, S4, S9).

To assess the preservation not only of ancient faunal but also of hominin DNA, which tends to be rare in sediments^4^, a relatively large number of sediment samples were collected from all sites to build a basis for potential follow-up studies. In total, 20 sediment samples were collected from the Middle and Upper Palaeolithic layers of Cudón, 13 from the Upper Palaeolithic layers from sectors I and III in Les Pedroses, 8 from the Upper Palaeolithic levels of Covarón and 64 from the Upper Palaeolithic layers in Altamira. In addition, in the Galería de las Pinturas in Covarón and in front of Figs. 3 and 4 in Les Pedroses, sediment samples were collected from spots on the cave floor identified as putative pigment preparation areas. All samples, except for 21 from Altamira, were screened for DNA preservation. In the cleanroom at MPI-EVA, we used sterile, single-use spatulas to remove smaller sub-samples for DNA extraction, ranging between 45 mg and 85 mg in weight. The remaining material, including the unscreened samples, was stored for further work.

### Sampling of rock art and unpigmented controls

Precautions were taken during sample collection to avoid present-day human DNA contamination, including the use of double layers of nitrile and/or latex gloves, disposable arm covers and face masks. Sampling strategies were adapted for each site and panel to account for the variable contexts in which pigments occurred, while minimizing visual impact on the rock art and adhering to site-specific restrictions defined by the permit-granting authorities. Most samples were collected using sterile, disposable scalpels — either scraping off a small amount of pigment with the tip of the scalpel or cutting small fragments of pigment-containing calcite if the material was soft. Alternatively, directed pressure was applied to detach hard fragments of pigment-containing minerals. At La Lloseta (Spain), a very hard calcite crust was removed primarily for dating using a clean screwdriver as a chisel. For two calcite crusts at Escoural, samples were obtained by gently tapping the scalpel with a hammer to release fragments for further sampling. A fresh scalpel was used for each sample, and any scalpels retaining traces of pigment were secured and stored. At Maltravieso, two hand stencils were sampled using a portable dentistry drill and a sterile swab.

Whenever possible, samples were directly collected in 2.0 ml Eppendorf tubes. Aluminium foil was placed underneath during sampling to prevent loss of pigment material. Larger pieces of mineral were collected directly on aluminium foil and later transferred to zip-lock bags. If samples broke during collection, they were later treated as independent sub-samples. Before collecting unpigmented control samples from the cave walls, images of the walls were taken and analysed using D-Stretch (https://www.dstretch.com/) for colour enhancement to verify the absence of pigment.

We also utilised flakes of pigment-containing minerals that had detached from cave walls due to natural erosion processes. At Altamira (Supplementary Note S1), Tyvek fabric was placed on the cave floor beneath areas where water was dripping from the ceiling, in order to collect small amounts of pigment carried by the water. This synthetic, dense polyethylene fabric is waterproof and resistant to bacterial growth and aging. Pieces of this fabric containing pigment fragments were cut, as well as additional pieces without pigment as controls. Additionally, at Altamira, pigment is regularly washed out in drip water, which was collected on the cave floor using funnels and beakers. One water sample was dried and pigment collected using a swab. In Covarón, eight calcite fragments with pigment were found on the cave floor of Galería de las Pinturas. These were collected using scalpels, along with two similar detached fragments without visible pigment which were collected as controls.

Detailed information about the sampling is provided in Supplementary Notes S1–S11. To mitigate costs and preserve material for potential follow-up studies, including chemical analyses, not all samples were screened for the purposes of this study. In all cases, documentation was performed using the following schemes: where applicable, images displaying sample IDs highlighted in yellow indicate samples that were screened for aDNA. Conversely, images displaying sample IDs on a white background were not processed for the purpose of this study. Sampled areas with red circles indicate pigment samples. Sampled areas with white circles are samples determined to be free from pigment (i.e. herein unpigmented samples or controls). An attempt was made in the field to photograph each sample with a scale. This was not always possible. In images where scales are indicated as approximate, e.g. “ ~1 cm” these have been scaled based of other images.

### Sub-sampling of pigment-containing samples

In the cleanroom of the MPI-EVA, large pigment-containing samples were divided into smaller sub-samples while separating pigmented from unpigmented parts. For SP.B.2674, a soft, pigmented calcite fragment from Panel 11 in Escoural, a scalpel was used to separate the fragment into two parts (Supplementary Note S8), which were further subdivided into flakes (*n* = 6). Moreover, pigment powder from the tube in which the original sample had been stored, as well as the tube itself, were later also included in DNA extraction. A similar strategy was used for SP.C.5459, a calcite fragment with pigment from Galería SESS in Cudón. Using a disposable scalpel, pigment was gradually scraped from the sample, resulting in four sub-samples with decreasing amounts of pigment. For SP.C.7182, the very hard calcite crust from La Lloseta, a dentistry drill was used to obtain five sub-samples: two from the included pigment and three from the calcite below and above.

The fragility of the calcite crust fragments collected at Escoural made it difficult to isolate pigment-containing from unpigmented parts during subsampling. We therefore impregnated some of them in resin using a method that does not interfere with DNA preservation or retrieval^34^. Using disposable shells as moulds (Supplementary Fig. S18), we first prepared a resin bed consisting of a mix of Viscovoss N55S unpromoted polyester resin and methyl ethyl ketone peroxide (MEKP) in the same ratio and incubated it at 60 °C for at least 24 h until solid. After that, we added the sample, impregnated it with the same resin mix, and hardened the resin again at 60 °C for between 2 and 7 days. After removing the mould with pliers, at least two sub-samples each of pigmented and unpigmented sample powder were collected by drilling. As additional controls, for some blocks we also took samples of the resin outside the embedded fragment.

### Sampling pigment from the airbrush

In the Museum of Prehistory and Archaeology of Cantabria (MUPAC, Santander, Spain), pigment samples were collected from inside the fractured diaphysis ALT2617 to avoid visible alterations. Using a 1.8 mm dentistry drill bit (without rotation), we gently scratched pigment from ~8 mm inside the bone into a 2 ml Eppendorf tube, targeting clumps of pigment near the ‘ALT2617’ label. This yielded a very small amount of pigment-containing powder. Two deeper attempts (up to ~2 cm) recovered no additional material. We repeated the process from the opposite end, obtaining small amounts of material in the first two attempts, with visible pigment only in the second. A final subsample, also with visible pigment, was collected by tapping the bone over a 2 ml tube.

### DNA extraction, library preparation and sequencing

DNA extraction was carried out on sediments, bone powders, and powders or fragments of cave wall samples in the clean room of the Max Planck Institute for Evolutionary Anthropology (MPI-EVA), using a silica-based method optimised for ancient DNA isolation^56^ (in the implementation with binding buffer ‘D’). For some of the pigment-containing pieces, we prepared two DNA extracts. Initially, the entire piece was submerged in 1 mL of extraction buffer, and the released DNA was purified from 150 µL of the supernatant. Subsequently, the piece was crushed into powder using a bead beater with ceramic beads (2.8 mm Precellys ceramic beads, 2000 rpm for at least 1 min), followed by a second incubation in extraction buffer using the same volumes. The DNA purification step, along with all subsequent sample preparation steps, was carried out using automated protocols on a Bravo NGS workstation (Agilent Technologies).

The entire volume of each DNA extract (30 µL) was converted into a DNA library using a single-stranded method^31,57^. A synthetic oligonucleotide was spiked into each reaction to monitor the efficiency of this process^58^. DNA libraries were quantified using quantitative PCR, amplified and indexed with two sample-specific barcodes^57^, then sequenced directly and/or enriched for desired genomic targets by hybridization capture prior to sequencing on Illumina’s MiSeq, HiSeq, NextSeq or NovaSeq X platforms in 2× 75 cycles paired-end mode. Hybridization capture of mtDNA was performed using an established method^59^, automated on a Hamilton NGS Star workstation^60^, using probes encompassing the revised Cambridge reference sequence of the human mtDNA genome^61^ or probes containing mtDNA sequences from 242 mammals^25^. In addition, some libraries were enriched for 1.35 million polymorphic sites in the human nuclear genome using the Ancient Human DNA panel and associated kit reagents from Twist Bioscience^38^ in an automated implementation of the manufacturer’s protocol on a Hamilton NGS Star workstation.

### Mitochondrial DNA sequence analysis

Paired-end sequences from the libraries enriched for human or mammalian mtDNA were overlap-merged into single-molecule sequences using leeHom^62^ (https://github.com/grenaud/leeHom) and taxonomically identified using ‘quicksand’^63^ (version 2.3). Quicksand assigns taxonomies using the kmer-based alignment-free classifier KrakenUniq^64^, bins sequences by mammalian biological families and maps them to their best matching reference genome within each family using BWA^65^, or in the case of sequences identified as *Hominidae*, to the revised Cambridge reference sequence of the human mtDNA genome^61^. For mammalian mtDNA captures, we disregarded families represented by fewer than 1% of the identified sequences and applied a minimum threshold on coverage breadth of 0.3^63^. For the human mtDNA captures, no coverage breadth filter was applied.

Sequences from each sample and biological family were evaluated for evidence of deamination. C-to-T substitution frequencies significantly exceeding 10% at both molecule ends - determined using binomial 95% confidence intervals - were considered indicative of ancient DNA. Since metagenomic analyses are prone to taxonomic misidentification, especially when only few sequences are assigned to a specific taxon, we verified the plausibility of all taxa showing evidence for ancient DNA based on geographic distribution. This further analysis removed a small number of taxa (*Cebidae*, *Cercopithecidae* and *Hylobatidae*), all of which were represented by <20 DNA sequences. For human mtDNA analysis of non-sediment samples, the deamination threshold was relaxed to maximize sensitivity, requiring a significant enrichment of C to T substitution (>10%, as described above) at only one end of the DNA molecules, rather than both. To assign human mtDNA sequences to a specific hominin lineage (modern human, Neandertals, Denisovans, or the Sima de los Huesos hominins), we analysed “diagnostic” positions in the mitochondrial genome^5^. Lineage support was assessed using either all sequences identified as human from a given sample or only the putatively deaminated fraction, defined by C-to-T substitutions at the first or last three positions of the sequence alignments.

### Nuclear DNA sequence analysis

Sequence data from libraries enriched for human nuclear DNA were analysed following an established workflow^35^ for the analysis of nuclear DNA from sediment. We re-implemented this workflow in snakemake (version: 5.17.0) introducing minor simplifications, modifications for broader applicability to different capture targets, and improvements for ease of use with SediQuest^66^. Specifically, sequences were first overlap-merged, mapped to a modified version of the human reference genome (hg19/GRCH37), in which the target positions were replaced by randomly selected third alleles. Off-target sequences, sequences shorter than 35 bp, or those with a mapping below 25 were removed. PCR duplicates were collapsed using bam-rmdup (version: 0.2.2; https://github.com/mpieva/biohazard-tools). To control for potential faunal contamination, mammalian ‘burden scores’ were computed for the Twist capture targets^35^. These scores allow analyses to be restricted to positions in the human genome where faunal sequences are unlikely to map due to high sequence divergence in the flanking regions. Contamination with present-day human DNA was estimated using AuthentiCT^30^.

Analyses were performed using different burden score thresholds, either on all sequences from each library or only on the subset of putatively deaminated sequences identified by C-to-T substitutions within the first or last three alignment positions. For compatibility with previously published data, we restricted our analysis to sites overlapping the “1240k” array, i.e. informative for human population genetics^67,68^ using samtools mpileup^69^ (version: 1.3) with parameters ‘-B’ to avoid base quality recalibration, ‘-R’ to treat all reads in the BAM file as one samples, ‘-Q 30’ for base quality and ‘-q 25’ for map quality filtering. We generated pseudo-haploid genotypes by random allele sampling from the low-coverage data using pileupCaller (https://github.com/stschiff/sequenceTools) with the ‘-single-stranded’ mode, to attenuate biases due to ancient DNA damage. We combined our data with those from 315 other prehistoric humans genotyped for the 1240k sites, 1187 present-day Eurasians genotyped for the Human Origin array sites, and three present-day Mbuti individuals from the Simons Genome Diversity Project (SGDP^70^). All genotypes were recovered from the Allen Ancient DNA Resource (version 54.1, https://www.nature.com/articles/s41597-024-03031-7, https://dataverse.harvard.edu/dataset.xhtml?persistentId=doi:10.7910/DVN/FFIDCW) using Poseidon (version: 2.7.1; poseidon-adna.org). Principal component analysis (PCA) was performed using smartpca^71,72^ (https://github.com/chrchang/eigensoft/blob/master/POPGEN/README), with lsqproject and Shrinkmode enabled, and using the ellipsoid estimation to plot the 0.95% confidence region. The ancient individuals were projected onto the variation defined by the present-day Eurasians in our dataset genotyped for the Human Origin target sites (*n* = 597,573 SNPs).

DNA sequences obtained by shotgun sequencing were taxonomically classified to flora (plants and algae) and fauna (vertebrates and invertebrates) with the ‘NED-flow’ pipeline (version NED-flow v1.0.0). In short, sequences were first filtered for low-quality and putative duplicate reads were merged with fastp^73^. Stretches of low-complexity sequences were identified using DustMasker^74^, and sequences containing more than 10 such bases were removed. The remaining sequences were mapped independently to all available plant (*n* = 2298 species), vertebrate (*n* = 4531 species) and invertebrate (*n* = 5483 species) nuclear reference genomes obtained from GenBank (Supplementary Data S6). DNA sequences were mapped using bowtie2^75,76^ with the ‘-very-sensitive’ setting, the ‘-rdg’ and ‘-rfg’ flags set to 999,999 to avoid gapped alignments, and -score-min L,−1.2,−1.2 to reduce the maximum identity score for considering the best match. All sequences were also mapped to a ‘bacterial sink‘ containing 20,720 concatenated bacterial genomes obtained from GenBank. Each DNA sequence mapping to the ‘sink’ with a minimal identify score of 90% were excluded from taxonomic classifying to flora and fauna. For each assembly, sequences mapping to flagged regions by the Foreign Contamination Screen^77^ (FCS) were masked, sequences with a higher-than-expected coverage were removed (coverage filter), as well as contigs/scaffolds with an excessive number of sequences mapping (contig filter). Of the remaining DNA sequences, those mapping with >95% identity score were assigned to the lowest common ancestor of all retained mappings, either at genus, family or order level. DNA damage was assessed at each taxonomic rank by calculating the C->T deamination frequency in the first and last three bases of the molecule. Families with more than 200 DNA sequences assigned (summed genus and family), and point estimates of deamination frequency >10% in both ends of the sequence were retained.

### Reporting summary

Further information on research design is available in the Nature Portfolio Reporting Summary linked to this article.

## Supplementary information

Supplementary Information

Description of Additional Supplementary Files

Supplementary Data 1

Supplementary Data 2

Supplementary Data 3

Supplementary Data 4

Supplementary Data 5

Supplementary Data 6

Supplementary Data 7

Reporting Summary

Transparent Peer Review file

## Source data

Source Data

## Data Availability

The genetic data from this study have been deposited in the European Nucleotide Archive under accession number PRJEB97209. DNA libraries prepared in this study, residual sample material as well side fractions retained from sample preparation are stored at the Max Planck Institute for Evolutionary Anthropology, Leipzig, Germany. Access can be requested by contacting Hipólito Collado Giraldo (hipolitocollado@gmail.com) and Matthias Meyer (mmeyer@eva.mpg.de). Source data are provided with this paper.

## Code availability

The computational pipelines used for metagenomic analyses in this study are available at https://github.com/Kevinnota/NED-flow and Zenodo [10.5281/zenodo.19453717] under the MIT license^78^, at https://github.com/aurore-bit/SediQuest and Zenodo [10.5281/zenodo.19389658]^66^ under the Creative Commons Attribution 4.0 International license and at https://github.com/mpieva/quicksand and Zenodo [10.5281/zenodo.19450770]^79^ under the Creative Commons Attribution 4.0 International license.
