# Supplementary material for: Investigating ancient human DNA preservation on cave walls and in rock art
Source: Nat Commun. 2026 Jun 23;17:5561. doi: 10.1038/s41467-026-74234-2 (PMC13291361; doi:10.1038/s41467-026-74234-2)
Supplement: Supplementary file 1 — Supplementary Information [file 41467_2026_74234_MOESM1_ESM.pdf]

Supplementary Information

Note S1 – Cueva de Altamira samples 2
Note S2 – Cueva de Balmori samples 3
Note S3 – Cueva de Cudón samples 4
Note S4 – Cueva del Covarón samples 5
Note S5 – Cueva del Higuerón samples 6
Note S6 – Gruta do Escoural samples 7
Note S7 – Cueva de La Lloseta sample 9
Note S8 – Cueva de Maltravieso samples 10
Note S9 – Cueva de Les Pedroses samples 11
Note S10 – Cueva del Tebellín samples 12
Note S11 – Cueva del Toro samples 13
Note S12 – Revelant permits for sampled caves and archeological objects 14
Note S13 – Mitochondrial DNA haplogroup analysis 16
Tables S1–S8 18
Figures S1–S28 26
Supplementary References 85

Note S1 – Cueva de Altamira samples

The samples from the Cave of Altamira (Fig. S1.1-S1.12) consist of six taken from a single Palaeolithic airbrush (Fig. 13), 43 taken from sediments from the stratigraphy located in the vestibule of the cave (Fig. S12.1), and nine from pigments from the decorated Ceiling of the Polychrome Hall (Fig. S1.2) and a wall located in the cave in Sector V (Fig. S1.3). (The pigment samples were collected between 2014 and 2021 (Fig. S1.2-S1.3).

The periodic control to which we submit different areas of the ceiling of the Polychrome Hall allows us to speak of a differential state of conservation. In this context, there are areas on the surface of the ceiling where the painting is occasionally affected by drips of infiltration water. This leads to the migration and washing away of particles of the mineral components that make up the paint. At the same time, there is a large central crack that crosses the ceiling from west to east, a crack that was sealed with cement mortar in the 1930s. This sealing has affected and continues to affect the infiltration of water into the ceiling of the room, causing some of the dripping with pigment particles, especially those corresponding to samples SP.B.1808 and SP.B.1805. The dragging and falling of pigment associated with the water that bathes the surface of the ceiling of the Polychrome Hall has been historically confirmed. However, it is only since April 2013 that we have clearly observed these episodes of migration and dripping affecting the painting in real time. In this context, sample SP.B.1805 (Fig. S1.4) of migrated pigment from a nearby claviform sign was taken on the surface of the aforementioned cement covering the central crack. The rest of the selected samples were obtained by placing containers and plate-like supports on the floor of the room (Fig. S1.5-S1.10), below the aforementioned active water dripping points, which occasionally drag paint particles. In no case was direct contact with the paint necessary to obtain them. Only the samples from Sector V were obtained by direct extraction of small pigment particles associated with a small calcite crust (Fig. S1.11-S1.12).

Note S2 – Cueva de Balmori samples

In Cueva de Balmori (Fig. S2.1-S2.10), a total of fifteen samples from three distinct areas in Conjunto 6, relatively close proximity to each other, were sampled for DNA analyses (Fig. S2.1).

From the Big red stein of Conjunto 6, five pigment samples (SP.C.6761-65) were taken, three of which were processed (Fig. S2.2-S5). Directly below this figure, five control samples were taken from an area free from visible pigment (SP.C.6766-70) of which two were screened (Fig. S2.2; S2.6-S2.7). The last five samples (SP.C.6771-75) were taken from a second area free from pigment further away from the first ten samples, three of which were screened (Fig. S2.2; S2.8-S2.10). Of the fifteen samples, eight were screened for aDNA: three of the five pigment samples and five of the ten controls.

Note S3 – Cueva de Cudón samples

A total of 20 samples of surface material from the cave walls were taken from Cueva de Cudón (Fig. S3.1-S3.12), in addition to 20 sediment samples from the stratigraphy (Fig. S12.2). Of these samples, ten were screened for aDNA: five of the eleven pigment samples and five of the nine controls. One pigment sample could-be subsampled from the backside providing an additional control.

Cave walls were sampled in four areas of the cave: *Galería del Techo de los Puntos* (Sector 5, Panel 108, Unidad gráfica 281) (Fig. S3.2-S4), *Hilera de los discos* (Sector 4, Panel 62, Unidad gráfica 167) (Fig. S3.5-S3.7), *Galería de SESS* (Sector 4, Panel 92, Unidad gráfica 224c) (Fig. S3.8-S9), and *Sala de los signos rojos* (Sector 1, Panel 19, Unidad gráfica 62) (Fig. S3.10-S3.12).

At *Galería del Techo de los Puntos* (Sector 5, Panel 108, Unidad gráfica 281), two pigment samples (SP.C.5449 & SP.C.5450) and three control samples (SP.C.5451-SP.C.5453) were taken (Fig. S3.2). The pigment samples were taken at an existing blemish to minimize damage to the surface of the cave wall (Fig. S3.2-S3.3). Control samples were taken in the immediately adjacent area (~2-5 cm away) from places devoid of any visible pigment (Fig. S3.4). Of the five samples taken at *Techo de los Puntos*, two were screened for aDNA: one of the two pigment samples, and one of the three controls.

At *Hilera de los discos* (Sector 4, Panel 62, Unidad gráfica 167), three pigment samples (including the scalpel used for sampling) (SP.C.5454/55 & SP.C.5456) and two control samples (SP.C.5457 & SP.C.5458) were taken (Fig. S3.5-S3.7). The pigment samples were again taken at an existing blemish to minimize damage to the existing cave art (Fig. S3.6). Samples deemed free from visible pigment were taken from the same general area but 4-10 cm away (Fig. S3.5, S3.7). Of the five samples taken at Signo de los Discos, two were screened for aDNA: one of the three pigment samples (the scalpel), and one of the two controls.

In *Galería de SESS* (Sector 4), three Unidades gráficas were sampled: Unidad gráfica 224a and Unidad gráfica 224c in Panel 92, Unidad gráfica 227 at Panel 93. At Panel 92, one pigment sample (SP.C.5459) and two control samples (SP.C.5460-SP.C.5461) were taken (Fig. S3.8). A nearby Unidad gráfica 224a provided an additional two samples: one with pigment and one without (used as a control). At Panel 93, opposite Panel 92, two additional samples were taken: one pigment (SP.C. 5464) and one control (SP.C.5465) a few centimeters to the left (Fig. S3.9). Of the seven samples taken from Galería SESS, four were screened for aDNA: two of the three pigment samples, and two of the four controls, plus an additional control sample was able to be taken from the pigment-free portion of pigment sample SP.C.5459.

At *Sala de los Signos Rojos* (Sector 1, Panel 19, Unidad gráfica 62), two pigment samples (including the scalpel used for sampling) (SP.C.5466 /67) and one control (SP.C.5468) were taken (Fig. S3.10-S3.12). Of the three samples taken here, two were screened for aDNA: the scalpel from the pigment sample (Fig. S3.11) and the sole control (Fig. S3.12).

Note S4 – Cueva del Covarón samples

In Cueva del Covarón (Fig. S4.1-S4.10), two main areas were sampled: an open area referred to as the Main hall not far from the southern entrance to the cave and an upper gallery deeper in the cave known as Galería de las Pinturas (Fig. S4.1). In the main area, there were 8 sediment samples taken from the stratigraphy (Fig. S12.3) and 8 samples from the Panel 1. Of these samples, all 8 sediment samples were screened for aDNA, as were 6 of the 8 cave wall samples; the two samples excluded from the screened set were tubes containing debris from sampling attempts that were already being included in the screening.

In the Main Hall, the first graphic unit sampled on the Panel 1 was that of a grid symbol over which a calcite deposit had developed (Fig. S4.2). Here the intent was to remove the cauliflower-shaped calcite for dating and access the pigment below the sample for DNA extraction. The initial attempt at removing the "cauliflower" resulted in crumbled calcite that appeared to be free from pigment and was therefore not screened for aDNA. The second attempt successfully removed the cauliflower and the resultant debris from the removal process was caught on clean aluminum foil and stored in a separate tube (Fig. S4.3). A second location on the grid symbol was targeted to the lower left of the first sampling spot (Fig. S4.2). Here there was an existing crack with pigment that was scraped and collected into a tube (Fig. S4.4). Around the corner to the left of the grid symbol, an area originally intended to be a control sample free from pigment was observed a small red stain and thereby served as another pigment sample (Fig. S4.1, S4.5). Below this sample, two control samples were taken in an area free from visible pigment (Fig. S4.1, S4.6). Of these eight samples, six were screened for aDNA: four of the six pigment samples and both controls.

In Galería de las Pinturas, damage to the cave wall was circumvented due to the discovery of small flakes of pigment on the cave floor directly adjacent to the pigment (Fig. S4.8). The flakes measuring 1 to 2 mm in the largest dimension were collected in sterile tubes amounting to a total of 8 pigment samples (Fig. S4.9). Two samples from the cave floor in the same general area but free from any visible pigment were then taken to serve as controls (Fig. S4.10). Of these ten samples, six were screened for aDNA: four of the eight pigment samples, as well as both controls.

Opposite the area where the flakes of pigment were collected, an area with visible pigment in the sediment was sampled in five distinct locations: three with what appeared to be obvious pigment in the sediment and two that appeared to be free of visible pigment (Fig. S4.7). All five were screened for aDNA.

Note S5 – Cueva del Higuerón samples

The Angular Sign in Panel B-VIII in Sector B of Cueva del Higuerón was sampled during October of 2023 (Fig. S5.1-S5.5). In total, five cave wall samples were taken from the panel, two targeting the visible pigment (Fig. S5.2). Three controls in the surrounding area were taken in areas free from visible pigment, including one to the upper right (Fig. S5.3), the second above the angular sign (Fig. S5.4), and the third farther to the left (Fig. S5.5).

Note S6 – Gruta do Escoural samples

Gruta do Escoural (Fig. S6.1-S6.19) was first sampled for DNA analyses in 2021 and then again in 2023. Two samples (Fig. S6.2) from Panel 11 were provided for DNA analysis in 2021. The first sample consisted of a piece of carbonate crust with red pigment adhered to the reverse side, which allowed for both the sampling of a pigment-free controls in addition to the pigment on the same sample. The second sample collected pigment from the freshly exposed wall below the carbonate crust. In 2023 further sampling was performed at Panel 11, as well as three additional areas: Panel 38, Panel 26 and Panel 64

Collected from the same area as in 2021 (Panel 11), four distinct sampling attempts resulted in nine samples (Fig. S6.3-S6.6), including scalpels retained due to residual pigment adhered to the blade). The first two sampling attempts used scalpels to cut the crust and resulted in multiple fragmented pieces, which further disintegrated during transport. For the latter two samples, a small hammer was used in conjunction with the scalpel to remove more intact pieces from the wall; these did not crumble during transport. Ten additional samples determined to be free of visible pigment were collected to screen as controls from the area below where the pigment samples were collected (including two scalpels used in the sampling); two of these were screened (Fig. S6.7).

A second sampling area, Panel 38, was sampled with a scalpel and produced three samples: the sample of the cave wall, the scalpel used in the sampling, and the resultant fragments collected on a collection tray during the sampling. Only the main sample was screened for aDNA (Fig. S6.8). Five samples free of visible pigment were later taken as controls from the area above the pigment sampling; three of these were screened for aDNA (Fig. S6.9-S6.10).

The third sampling area, Panel 26, required a ladder to sample two distinct locations exhibiting concentrated areas of pigment, resulting in four samples (two samples and each of the scalpels with visible pigment adhered to the blade). Only the two main samples were screened for DNA (Fig. S6.11). Five pigment-free samples were collected in the surrounding area: three on the same wall as the pigment samples (Fig. S6.12) and two on the opposite wall (Fig. S6.13); four of the five were screened for aDNA.

The fourth sampled area, Panel 64, sampled one spot of pigment with a scalpel, resulting in a single sample (Fig. S6.14-S6.15). Areas of the surrounding cave walls determined to be pigment-free were then targeted to collect samples to serve as controls (Fig. S6.14). Six such control samples were taken from the cave wall immediately adjacent to the pigment sample, three of which were screened for aDNA (Fig. S6.16). A second equally hard to reach area on the wall opposite the pigment sample area, produced five additional control samples (Fig. S6.17); three of these were screened for aDNA. And below the first two areas sampled, two additional areas were then sampled for controls: one below the controls taken from the opposite wall (Fig. S6.18) and the other in the region below the pigment sample (Fig. S6.19). Five controls were collected from each of these areas. Once more, three from each set were screened for aDNA.

In total 58 samples were collected (this number includes sampling scalpels retained due to material adhered to the blade): 17 pigment samples (8 of which were scalpels) and 41 pigment-free controls (including 2 scalpels). Of these, eight pigment samples and 21 controls were screened for aDNA.

Note S7 – Cueva de La Lloseta sample

A single sample (SP.C.7182) from Conjunto 9 of Cueva de la Lloseta was provided for DNA analysis in 2024 (Fig. S7.1-S7.5). The sample exhibited clear bands of pigment sandwiched between calcite enabling an approach to sample pigment-free calcite to either side of the pigment in the sample in addition to the pigment itself. The sample was drilled in two rounds. The first round of sampling, which produced three subsamples (Fig. S7.3-S7.4), aimed to sample the inner calcite, then the external calcite, and lastly an area visually rich in pigment. The second round produced just two additional subsamples: one from the inner calcite and the other from the band of pigment (Fig. S7.5); a third sample was not attempted because it would have risked breaking the sample. Of the five total subsamples collected, four were screened for aDNA for the purposes of this study: both of pigment subsamples and two of the three control subsamples.

Note S8 – Cueva de Maltravieso samples

Two hand stencils were sampled in 2019 at Cueva de Maltravieso (Fig. S8.1-S8.4) from two nearby Panels (Fig. S8.2) in Galería de la Serpiente (GS). The first stencil, Mano 53 (GS-III 3), was sampled in two distinct locations (Fig. S8.3). The first location aimed to sample pigment exposed by a U/Th sample taken in 2018. The first four attempts used scalpels and only served to remove surface calcite, one of which was screened as a pigment-free control sample. A sterile dentistry drill was then applied to reach the pigment and even still the first two samples only produced pigment-free calcite, one of which was screened as a control. The third drilling attempt was able to retrieve a sample of the pigment. The second spot sampled at GS-III 3 (between the thumb and forefinger) forewent the scalpel attempts and utilized the drilling method straight away. After removing five samples of pigment-free calcite (two of which were screened as controls), two sterile swabs were taken of the exposed pigment, one of which was screened for aDNA. The first sampling location was then returned to using a sterile swab to collect the exposed pigment from the surface, followed by two additional drilled samples.

The second of the two hand stencils, Mano 59 (GS-V 4), was sampled at one location and used the approach previously described: drilling followed by swabbing (Fig. S8.4). The first drilling attempts resulted in three samples free from pigment, two of which were screened as controls, after which the sampled location was wiped with two successive sterile swabs, one of which was screened for aDNA. Of the 22 total samples collected, 11 were screened for aDNA: four of the eight pigment samples and seven of the 14 controls.

Note S9 – Cueva de Les Pedroses samples

Seven samples were taken from the cave walls at Les Pedroses (Fig. S9.1-S9.13), in addition to 13 sediment samples taken from the stratigraphy (five in Sector I and eight samples in Sector III near the cave entrance, Fig. S12.4). The cave wall samples consisted of four samples of visible pigment and three controls. As in Cueva del Covarón, there was also an area where there was apparent pigment in the sediment of the cave floor from which two successive sediment samples were taken (Fig. S9.2-S9.3); no control was taken from this area in light of the other sediment samples taken from the exposed profiles elsewhere in the cave.

The uppermost dot near another cluster of black dots known as Figure 8A/8B was sampled in the area to the right of Galería I (Fig. S9.4). Sampling this mark (Fig. S9.5) revealed more black material beneath the surface; this was not sampled further in the interest of minimizing surface alterations. Approximately twenty centimeters to the left of this sample, the cave wall free from pigment was sampled to serve as a control (Fig. S9.6).

The three other pigment samples and two remaining controls came from an area known as Galería I (Fig. S9.7). Three graphic units from this area were sampled: a blown disc known as Figure 3 (Fig. S9.11), a big red stein suggestive of having been made with fingertips known as Figure 4 (Fig. S9.12) and a small group of red stains known as Figure 3A, located at right of blown disc (Fig. 3, S9.9). The first of two controls was taken in an area free from pigment between Figure 3 and Figure 3A (Fig. S9.10). The second control sample was taken in a deeper part of Galería I between Figure 4 and Figure 3 (Fig. S9.13). Of the nine total samples, seven were screened for aDNA: four of the six pigment samples along with all three of the controls.

Note S10 – Cueva del Tebellín samples

In Cueva del Tebellín (Fig. S10.1-S10.7) sampling for DNA analyses were taken from the Sala Central. Two pigment samples were collected from a red disk-shaped graphic unit known as Figure 7, located on the right wall, directly opposite Conjunto I (Fig. S10.2-S10.3). Also, three controls were taken from areas free of any visible pigment around the corner towards the southeast from the red disk (Fig. S10.4). Another six pigment samples were collected from within a deep crack in the big claviform figures known as Figure 1a/1b, located in Conjunto I, leaving no detectable alteration to the cave wall – half of these were screened for aDNA (Fig. S10.5-S10.7). Of the eleven total samples, five were screened for aDNA: four of the eight pigment samples and one of the three controls.

Note S11 – Cueva del Toro samples

Cueva del Toro (Fig. S11.1-S11.9) was initially sampled for DNA analyses in 2022 and again in 2023. In 2022, a single pigment sample from Panel 1 of Galería de los Discos in figure CTO-3 (SP.C.5469) was provided and screened for aDNA (Fig. S11.2-S11.3). A sampling campaign targeting additional samples for aDNA analysis followed in 2023 wherein 12 samples were taken from two sampled areas: *Galería de los Discos*, where Figure CTO-2 from Panel 1 was sampled, along with a series of control samples taken from the wall of this narrow corridor to the left of Panel 1 (Fig. S11.4-S11.7) and then at *Adjacent wall of Sala de las Pinturas* (Fig. S11.8-S11.9). Of these samples, seven were screened for aDNA: all four of the pigment samples and three of the eight controls.

Note S12 – Revelant permits for sampled caves and archeological objects

The sampling activities presented in this article were carried out within the framework of the project **FIRST ART (Ref. No. 0497_FIRST_ART_4_E)**, funded under the Interreg V-A Spain–Portugal (POCTEP) cross-border cooperation programme and coordinated by the Government of Extremadura.

The aforementioned FIRST ART project has established collaborative agreements with the research teams responsible for the caves and archaeological objects sampled. In all cases, the work was conducted under the appropriate intervention authorisations issued by the competent heritage management authorities. The corresponding permit records are detailed below:

- **Cave of Altamira**: Research collaboration agreement authorised by Pilar Fatás Monforte, Director of the National Museum and Research Centre of Altamira (12/09/2023).

- **Maltravieso**: The Government of Extremadura designed Hipolito Collado Giraldo as responsible for the the documentation, conservation and the intervention permits for the Maltravieso cave (25/02/2013).

- **Tebellín, Balmori, Lloseta, and Pedroses Caves**: File No. 01248/22 issued by the Directorate-General for Cultural Heritage of the Principality of Asturias, in favour of Mr. Alberto Martínez Villa.

- **Higuerón Cave**: File AA. No. 174/24-17653 issued by the Territorial Delegation of Tourism, Culture, and Sport of the Regional Government of Andalusia, authorising the archaeological intervention entitled *"Test excavation, reproduction, and direct study of rock art in Victoria and Higuerón Caves, Rincón de la Victoria, Málaga"*, in favour of Ms. Mª del Mar Espejo Herrerías.

- **Toro Cave**: File AA. No. 106/25-19240 issued by the Territorial Delegation of Tourism, Culture, and Sport of the Regional Government of Andalusia, authorising the archaeological intervention entitled *"Study and graphic documentation of prehistoric rock art in Toro (or Calamorro) Cave. Archaeometric characterisation and U/Th dating of Palaeolithic art in Málaga Bay, Benalmádena, Málaga"*, in favour of Mr. Diego Salvador Fernández Sánchez.

- **Escoural Cave**: File No. 1367237/DBC/2019 issued by the Ministry of Culture of Portugal for the archaeological activity *"Conservation, documentation, and management of the earliest rock art manifestations in the southwest of the Iberian Peninsula: Escoural Cave"*, in favour of Ms. Sara Garcés.

- **Cudón Cave**: Registration No. 01758 authorisation granted by the Regional Ministry of Education, Culture, and Sport of the Government of Cantabria for the archaeological intervention *"The parietal record of Cudón Cave: micro-topographic mapping,*

documentation, characterisation, and chrono-stylistic analysis", in favour of Mr. Ramón Montes Barquín. In addition, File AAA 128/22 issued by the Regional Ministry of Universities, Equality, Culture, and Sport of the Government of Cantabria authorised the archaeological intervention *"Geo-archaeological sampling of Cudón Cave"*, also in favour of Mr. Ramón Montes Barquín.

- **Covarón**: File No. AUTO/2022/11149 issued by the Comisión Permanente del Consejo de Patrimonio Cultural de Asturias *"Proyecto 2022. Cueva de El Covarón (La Pereda, Llanes). Estudio integral del yacimiento y de las manifestaciones rupestres"*, in favour of Dr. Mario Menéndez.

- **Altamira Aerograph**: File No. 045-23 issued by the Museum of Prehistory and Archaeology of Cantabria, authorising sampling of aerographs (Altamira Cave), in favour of Mr. Hipólito Collado Giraldo.

Note S13 – Mitochondrial DNA haplogroup analysis

Modern human mtDNA genomes can be classified into haplogroups. In high-quality samples, for example from bones, haplogroups are called from the complete mtDNA consensus sequences by evaluating the full set of haplogroup-specific diagnostic positions. However, due to the very low coverages and presence of recent human contamination in the cave wall samples containing ancient human DNA, mtDNA consensus-calling is infeasible (Data S3 and Table S1). We therefore tested the haplogroup caller mixEMT[1], which is a consensus-free haplogroup caller designed to in detect and quantify mixtures of haplogroups in forensic samples.

To account for low coverage and missing data, we ran mixEMT with settings optimized for high sensitivity (-f 0.3 -r 5 -R 2). To mitigate the effect of ancient DNA damage, we trimmed three bases from each end of the sequences before calling mtDNA haplogroups. We also run mixEMT using only putatively deaminated sequences, which were identified by the presence of C-to-T substitutions in their first three or last three positions.

Unfortunately, mixEMT detected only a single mtDNA haplogroup in each library (Table S2), indicating that haplogroup mixtures resulting from contamination could not be deconvoluted. This result is consistent with the reported sensitivity of mixEMT, which requires around 7x genomic coverage to call more than one haplogroup. When using only deaminated sequences to remove present-day human contamination, mixEMT failed to identify any haplogroup.

We therefore attempted a different approach to identify the best fitting haplogroup, making use of the fact that human mtDNA haplogroup nomenclature follows the evolutionary tree and assigns haplogroup labels (defined by specific mutations) not only to the terminal leaves, which correspond to present-day individuals, but also to internal nodes. Using only deaminated sequences, we generated coverage and support statistics for each haplogroup node, based on the PhyloTree 17 release[2]. We then followed the tree from the root toward the tips, following the most strongly supported lineage and assigning each sample to the most downstream (i.e., most specific) haplogroup for which support was observed. A standalone implementation of this analysis workflow can be found on github (https://github.com/merszym/alphabet).

Specifically, we mapped all 'Hominidae' sequences classified by quicksand to the Reconstructed Sapiens Reference Sequence (RSRS[3]) using BWA[4] and removed PCR duplicates with bam-rmdup (https://github.com/mpieva/biohazard-tools). We then removed all sequences overlapping low-complexity poly-C-stretches in the mtDNA reference genome (positions 303-315, 513-576, 3565-3576 and 16184-16193)[5] using bedtools[6]. Next, sequences were extracted that showed a C-to-T substitution in the first or last three bases relative to the reference. In this process, we disregarded C-to-T substitutions at positions where the majority of overlapping sequences showed a base difference to the reference, as these likely reflect actual evolutionary sequence differences rather than deamination.[7]

To account for DNA damage, we disregarded all T's (in the orientation as sequenced) in the first and last three sequence positions when calculating coverage and support statistics. Traversing the PhyloTree 17 phylogeny, we then counted for each haplogroup the total number of covered and supported haplogroup-defining positions (position support) as well as the accumulated number of covered and supported positions in all parent haplogroups (branch support). In addition, we recorded detailed coverage information per position, including also for uncovered haplogroup-defining positions (sequence support).

We then simplified the tree by removing all branches in the tree that I) show less than 70% branch support, or II) are separated from the nearest supported haplogroup by two or more unsupported intermediate nodes, or III) have a position support of zero and no supported haplogroup in any of the descendants. In an example provided in Table S3, haplogroups U1a1a1 and U1a1a2 are removed because of zero position support, haplogroup U1a1 and its descendants are excluded because of a branch support of less than 70%. Finally, the full U1 branch is removed (despite a branch-support larger than zero) because U1a1a+16129G is 4 nodes away from U. In this example, the best supported next haplogroup is U5. The filtered tables for the five cave wall samples (produced using https://github.com/merszym/alphabet in version v0.5) can be found in Data S7.

After filtering, we selected the path with the highest branch support and the least number of interruptions to arrive at the most supported haplogroup, while further limiting the maximum number of unsupported intermediate nodes to one. Using this approach, for the pigmented cave wall samples from Escoural (SP.B.2674), we found support for the path from the RSRS to haplogroup N but not for subclades within N (Data S7.1 and Table S4). Among the unpigmented cave wall samples from Escoural, the first, SP.C.6810, yielded almost no resolution, with the path ending at haplogroup L3‘4‘6 (Data S7.2 and Table S5). For the other sample (SP.C.6813), which yielded the highest number of deaminated sequences from any of the cave wall samples (Table S1), we found support for both haplogroups U and R0, which derive from haplogroup R, but not for subclades within U and R0 (Data S7.3). The defining positions for haplogroups R0 and U are each supported by approximately half of the sequences (Table S6, 11719G: 1/2; 12308G: 3/7), indicating a possible mixture of haplogroups in the data. The analysis of the cave wall samples from Covaron (SP.C.5546 and SP.C.5547; Data S7.4-7.5 and Tables S5-S6) support the path to haplogroup U5a'b for SP.C.5547 and to N1'5 for SP.C.5546.

Following these automated assignments, we visually evaluated alternative possibilities. For the pigmented cave wall sample from Covarón (SP.B.2674), assignment to haplogroup HV would appear possible when accepting two intermediate nodes (R and R0) without sequence coverage. However, inspection of the two DNA fragments supporting the defining position for HV showed one to be a likely PCR artefact and the other to provide questionable evidence for deamination (Fig. S28).

In another case, SP.C.5546 from Covarón, haplogroup N1'5 is supported by only one of two DNA fragments overlapping the haplogroup-defining position, with the preceding N node not covered.

Along an alternative path via R and U (both lacking coverage at their defining positions), support exists for haplogroup U5 (2/2 DNA fragments covering one of its two defining positions) and its subclade U5a'b (1/1 fragment covering one of its three defining positions). Manual inspection therefore suggests a better match to U5a'b for this sample.

These ambiguities highlight the limitations of this approach. The low mitochondrial sequence coverage obtained from the samples analysed here, makes the analysis prone to haplogroup misassignments due to DNA damage, sequencing errors, or small amounts of residual human or faunal contamination. The results presented here should therefore be considered tentative.

Table S1: Overview of the samples, libraries and data used for haplogroup assignment

Capture ID	Sample ID	Description	Unique Sequences	Genomic Coverage	Contamination	Deaminated sequences
Cap.I.6139	SP.B.2674	Escoural, wall, pigmented	526	1.74	54.8 %	54
Cap.I.6143	SP.B.2674		436	1.49	32.4 %	53
Cap.L.2829	SP.C.6810	Escoural, wall	351	1.04	33.4 %	69
Cap.L.2831	SP.C.6813	Escoural, wall	1,980	6.37	0.1 %	545
Cap.J.5788	SP.C.5546	Covarón, wall	904	3.15	67.6 %	105
Cap.J.5789	SP.C.5547	Covarón, wall	773	2.61	59.3 %	111

Table S2: mixEMT haplogroup calls

Capture ID	Sequences	Genomic Coverage	mixEMT Results (unique sequences)	mixEMT Results (deaminated)
Cap.I.6139	Unique sequences	1.74x	H1ax	-
Cap.I.6143	Unique sequences	1.49x	H16e	-
SP.B.2674	Merged sequence	3.23x	H5a1i	-
Cap.L.2829	Unique sequences	1.04x	-	-
Cap.L.2831	Unique sequences	6.37x	H5f	-
Cap.J.5788	Unique sequences	3.15x	H4a1a4b	-
Cap.J.5789	Unique sequences	2.61x	H4a1a4b	-

Table S3: Example of Haplogroup estimate output table prior to filtering. The example starts with a hypothetical branch support of (15/15) on the U node. The ‘Penalty‘ column shows the distance to the closest supported haplogroup.

Haplogroup Tree	Branch Support	Position Support	Penalty
+-- **U**	15/15 (100%)	2/2	0
I -- U1	15/20 (75%)	0/5	*4
I I-- U1a	15/21 (71.42%)	0/1	*3
I I I-- U1a1	15/23 (*65.21%)	0/2	2
I I I I-- U1a1a	15/25 (*60%)	0/2	1
I I I I I-- U1a1a1	15/25 (*60%)	0/0	*-1
I I I I I +-- U1a1a1a	15/26 (*57.69%)	0/1	*-1
I I I I I-- U1a1a2	15/26 (*57.69%)	0/1	*-1
I I I I +-- **U1a1a+16129G!**	16/26 (*61.53%)	1/1	0
I I I I +-- U1a1a3	16/27 (*59.26%)	0/1	*-1
…			
I -- **U5**	16/16 (100%)	1/1	0
**Bold:** haplogroups with position-support.<br>* Filter conditions apply			

Table S4: Haplogroup estimate for sample SP.B.2674 (merged sequences)

Haplogroup	Branch Support	Position Support	Sequence Support
L1'2'3'4'5'6	2/2 (100%)	2/2	146T (0.00% 0/0); 182T (0.00% 0/0); **4312C** (100.00% 1/1); 10664C (0.00% 0/0); **10915T** (100.00% 1/1); 11914G (0.00% 0/0); 13276A (0.00% 0/0); 16230A (0.00% 0/0)
└──── L2'3'4'5'6	4/4 (100%)	2/2	152T (0.00% 0/0); 2758G (0.00% 0/0); 2885T (0.00% 0/0); **7146A** (100.00% 2/2); **8468C** (100.00% 1/1)
└──── L2'3'4'6	5/5 (100%)	1/1	195T (0.00% 0/0); 247G (0.00% 0/0); 825T (0.00% 0/0); 8655C (0.00% 0/0); 10688G (0.00% 0/0); **10810T** (100.00% 1/1); 13105A (0.00% 0/0); 13506C (0.00% 0/0); 15301A (0.00% 0/0); 16129G (0.00% 0/0); 16187C (0.00% 0/0); 16189T (0.00% 0/0)
└──── L3'4'6	5/5 (100%)	0/0	4104A (0.00% 0/0); 7521G (0.00% 0/0)
└──── L3'4	6/6 (100%)	1/1	182C! (0.00% 0/0); 3594C (0.00% 0/0); **7256C** (100.00% 1/1); 13650C (0.00% 0/0); 16278C (0.00% 0/0)
└──── L3	8/9 (88.89%)	2/3	**769G** (100.00% 1/1); **1018G** (0.00% 0/1); **16311T** (100.00% 1/1)
└──── N	9/10 (90%)	1/1	8701A (0.00% 0/0); **9540T** (100.00% 1/1); 10398A (0.00% 0/0); 10873T (0.00% 0/0); 15301G! (0.00% 0/0)
└──── R	9/10 (90%)	0/0	12705C (0.00% 0/0); 16223C (0.00% 0/0)
└──── R0	9/10 (90%)	0/0	73A (0.00% 0/0); 11719G (0.00% 0/0)
└──── HV	10/11 (90.91%)	1/1	**14766C** (100.00% 2/2)
**Bold:** covered positions.			

Table S5: Haplogroup estimate for sample SP.C.6810 (Cap.L.2829)

Haplogroup	Branch Support	Position Support	Sequence Support
L1'2'3'4'5'6	2/2	2/2	146T (0.00% 0/0); 182T (0.00% 0/0); 4312C (0.00% 0/0); **10664C** (100.00% 1/1); 10915T (0.00% 0/0); 11914G (0.00% 0/0); **13276A** (100.00% 1/1); 16230A (0.00% 0/0)
└── L2'3'4'5'6	3/3	1/1	152T (0.00% 0/0); 2758G (0.00% 0/0); **2885T** (100.00% 1/1); 7146A (0.00% 0/0); 8468C (0.00% 0/0)
└── L2'3'4'6	4/4	1/1	195T (0.00% 0/0); 247G (0.00% 0/0); 825T (0.00% 0/0); 8655C (0.00% 0/0); 10688G (0.00% 0/0); **10810T** (100.00% 1/1); 13105A (0.00% 0/0); 13506C (0.00% 0/0); 15301A (0.00% 0/0); 16129G (0.00% 0/0); 16187C (0.00% 0/0); 16189T (0.00% 0/0)
└── L3'4'6		0/0	4104A (0.00% 0/0); 7521G (0.00% 0/0)
└── L3'4	5/5	1/1	182C! (0.00% 0/0); 3594C (0.00% 0/0); **7256C** (100.00% 4/4); 13650C (0.00% 0/0); 16278C (0.00% 0/0)
**Bold:** covered positions.			

Table S6: Haplogroup estimate for sample SP.C.6813 (Cap.L.2831)

Haplogroup	Branch Support	Position Support	Sequence Support
L1'2'3'4'5'6	5/7 (71.43%)	5/7	**146T** (100.00% 2/2); **182T** (0.00% 0/1); **4312C** (0.00% 0/1); **10664C** (100.00% 1/1); **10915T** (100.00% 2/2); **11914G** (100.00% 3/3); **13276A** (100.00% 2/2); 16230A (0.00% 0/0)
└── L2'3'4'5'6	10/12 (83.33%)	5/5	**152T** (100.00% 2/2); **2758G** (100.00% 2/2); **2885T** (100.00% 3/3); **7146A** (100.00% 3/3); **8468C** (100.00% 1/1)
└── L2'3'4'6	19/22 (86.36%)	9/10	**195T** (100.00% 1/1); **247G** (100.00% 1/1); **825T** (100.00% 2/2); **8655C** (100.00% 1/1); **10688G** (100.00% 1/1); **10810T** (100.00% 2/2); **13105A** (100.00% 3/3); **13506C** (100.00% 2/2); **15301A** (0.00% 0/2); **16129G** (100.00% 1/1); 16187C (0.00% 0/0); 16189T (0.00% 0/0)
└── L3'4'6	21/24 (87.50%)	2/2	**4104A** (100.00% 2/2); **7521G** (100.00% 2/2)
└── L3'4	26/28 (92.86%)	4/4	**182C**! (100.00% 1/1); **3594C** (100.00% 3/3); **7256C** (100.00% 3/3); 13650C (0.00% 0/0); **16278C** (100.00% 1/1)
└── L3	29/31 (93.55%)	3/3	**769G** (100.00% 3/3); **1018G** (100.00% 1/1); **16311T** (100.00% 1/1)
└── N	33/34 (97.06%)	3/3	8701A (0.00% 0/0); **9540T** (100.00% 2/2); 10398A (0.00% 0/0); **10873T** (100.00% 1/1); ****15301G**! (100.00% 2/2)
└── R	33/34 (97.06%)	0/0	12705C (0.00% 0/0); 16223C (0.00% 0/0)
├── R0	34/35 (97.14%)	1/1	73A (0.00% 0/0); **11719G** (50.00% 1/2)
└── U	35/36 (97.22%)	2/2	**11467G** (100.00% 1/1); **12308G** (42.86% 3/7); 12372A (0.00% 0/0)

Bold: covered positions.

** Position 15301G! is counted twice in the branch support. ‚G!‘ indicates a back-mutation of 15301A that was unsupported in L2'3'4'6. Position 15301G! therefore supports haplogroup N, as well as L2'3'4'6.

Table S7: Haplogroup estimate for sample SP.C.5546 (Cap.J.5788)

Haplogroup	Branch Support	Position Support	Sequence Support
L1'2'3'4'5'6	3/3 (100%)	3/3	146T (0.00% 0/0); 182T (0.00% 0/0); 4312C (0.00% 0/0); **10664C** (100.00% 1/1); 10915T (0.00% 0/0); **11914G** (100.00% 2/2); **13276A** (100.00% 1/1); 16230A (0.00% 0/0)
└── L2'3'4'5'6	5/5 (100%)	2/2	152T (0.00% 0/0); 2758G (0.00% 0/0); 2885T (0.00% 0/0); **7146A** (100.00% 1/1); **8468C** (100.00% 1/1)
└── L2'3'4'6	7/7 (100%)	2/2	195T (0.00% 0/0); **247G** (100.00% 1/1); 825T (0.00% 0/0); 8655C (0.00% 0/0); 10688G (0.00% 0/0); **10810T** (100.00% 1/1); 13105A (0.00% 0/0); 13506C (0.00% 0/0); 15301A (0.00% 0/0); 16129G (0.00% 0/0); 16187C (0.00% 0/0); 16189T (0.00% 0/0)
└── L3'4'6	8/8 (100%)	1/1	**4104A** (100.00% 1/1); 7521G (0.00% 0/0)
└── L3'4	10/10 (100%)	2/2	182C! (0.00% 0/0); **3594C** (100.00% 1/1); 7256C (0.00% 0/0); 13650C (0.00% 0/0); **16278C** (100.00% 2/2)
└── L3	11/11 (100%)	1/1	769G (0.00% 0/0); **1018G** (100.00% 1/1); 16311T (0.00% 0/0)
└── N	11/11 (100%)	0/0	8701A (0.00% 0/0); 9540T (0.00% 0/0); 10398A (0.00% 0/0); 10873T (0.00% 0/0); 15301G! (0.00% 0/0)
├── N1‘5	12/12 (100%)	1/1	**1719A** (50.00% 1/2)
└── R	11/11 (100%)	0/0	12705C (0.00% 0/0); 16223C (0.00% 0/0)
├── R0	12/12 (100%)	1/1	**73A** (100.00% 1/1); 11719G (0.00% 0/0)
└── U	11/11 (100%)	0/0	11467G (0.00% 0/0); 12308G (0.00% 0/0); 12372A (0.00% 0/0)
└── U5	12/12 (100%)	1/1	16192T (0.00% 0/0); **16270T** (100.00% 2/2)
└── U5a’b	13/13 (100%)	1/1	3197C (0.00% 0/0); **9477A** (100.00% 1/1); 13617C (0.00% 0/0)

Table S8: Haplogroup estimate for sample SP.C.5547 (Cap.J.5789)

**Haplogroup**	**Branch Support**	**Position Support**	**Sequence Support**
L1'2'3'4'5'6	0/0 (0%)	0/0	146T (0.00% 0/0); 182T (0.00% 0/0); 4312C (0.00% 0/0); 10664C (0.00% 0/0); 10915T (0.00% 0/0); 11914G (0.00% 0/0); 13276A (0.00% 0/0); 16230A (0.00% 0/0)
└── L2'3'4'5'6	1/1 (100%)	1/1	152T (0.00% 0/0); 2758G (0.00% 0/0); 2885T (0.00% 0/0); 7146A (0.00% 0/0); **8468C** (100.00% 1/1)
└── L2'3'4'6	4/5 (80%)	3/4	195T (0.00% 0/0); 247G (0.00% 0/0); 825T (0.00% 0/0); **8655C** (100.00% 1/1); 10688G (0.00% 0/0); **10810T** (100.00% 1/1); **13105A** (100.00% 1/1); 13506C (0.00% 0/0); **15301A** (0.00% 0/1); 16129G (0.00% 0/0); 16187C (0.00% 0/0); 16189T (0.00% 0/0)
└── L3'4'6	5/6 (83.33%)	1/1	**4104A** (100.00% 1/1); 7521G (0.00% 0/0)
└── L3'4	7/9 (77.78%)	2/3	182C! (0.00% 0/0); **3594C** (0.00% 0/1); **7256C** (100.00% 1/1); **13650C** (100.00% 1/1); 16278C (0.00% 0/0)
└── L3	9/11 (81.82%)	2/2	**769G** (100.00% 2/2); **1018G** (100.00% 1/1); 16311T (0.00% 0/0)
└── N	11/12 (91.67%)	1/1	8701A (0.00% 0/0); 9540T (0.00% 0/0); 10398A (0.00% 0/0); 10873T (0.00% 0/0); ****15301G**! (100.00% 1/1)
└── R	11/12 (91.67%)	0/0	12705C (0.00% 0/0); 16223C (0.00% 0/0)
└── U	12/13 (92.31%)	1/1	11467G (0.00% 0/0); 12308G (0.00% 0/0); **12372A** (100.00% 1/1)
└── U5	12/13 (92.31%)	0/0	16192T (0.00% 0/0); 16270T (0.00% 0/0)
└── U5a'b	13/14 (92.86%)	1/1	**3197C** (100.00% 1/1); 9477A (0.00% 0/0); 13617C (0.00% 0/0)

Bold: covered positions.

** see Table S6

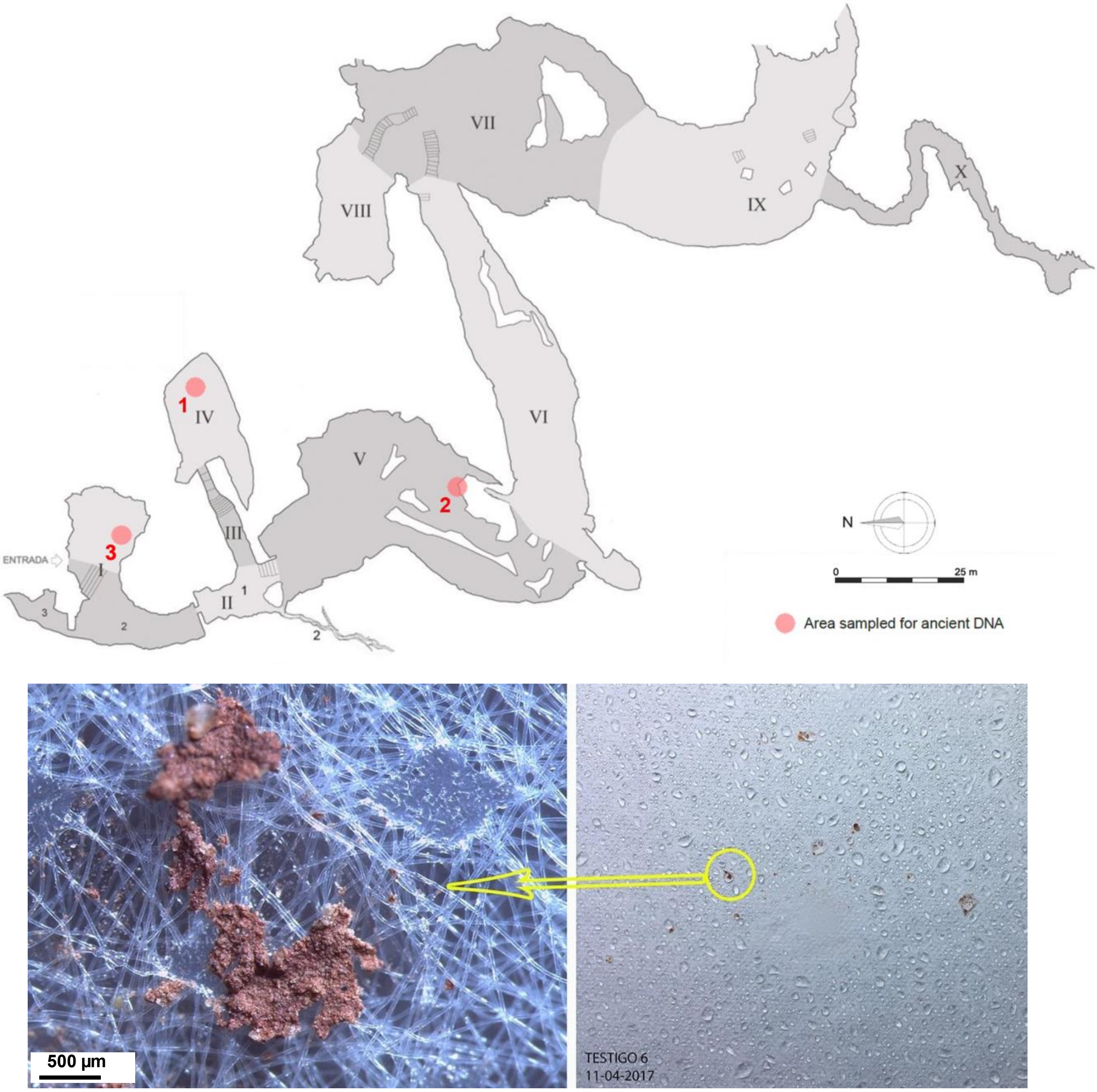

Sample ID	a.k.a.	Area	Sample Type
SP.B.1805	ALT-5_2020	Sector IV: Polícromos	Pigment (Ceiling)
SP.B.1806	ALT-6_2020	Sector IV: Polícromos	Pigment & Control (Passive)
SP.B.1807	ALT-7_2020	Sector IV: Polícromos	Pigment & Control (Passive)
SP.B.1808	ALT-8_2020	Sector IV: Polícromos	Pigment (Passive)
SP.B.2202	ALT-12_2021	Sector IV: Polícromos	Pigment & Control (Passive)
SP.B.2203	ALT-13_2021	Sector IV: Polícromos	Pigment & Control (Passive)
SP.B.2204	ALT-14_2021	Sector IV: Polícromos	Pigment & Control (Passive)
SP.B.2206	ALT-16_2021	Sector V	Pigment (Wall)
SP.B.2207	ALT-17_2021	Sector V	Pigment (Wall)

Fig. S1.1. Plan indicating areas sampled for DNA analyses at Cave of Altamira: Polícromos (1); Hybrid panel (2) and sediment sampling Sector I (3) (upper); magnification of a pigment sample passively collected from the cave floor underlayment (middle); list of samples screened (lower).

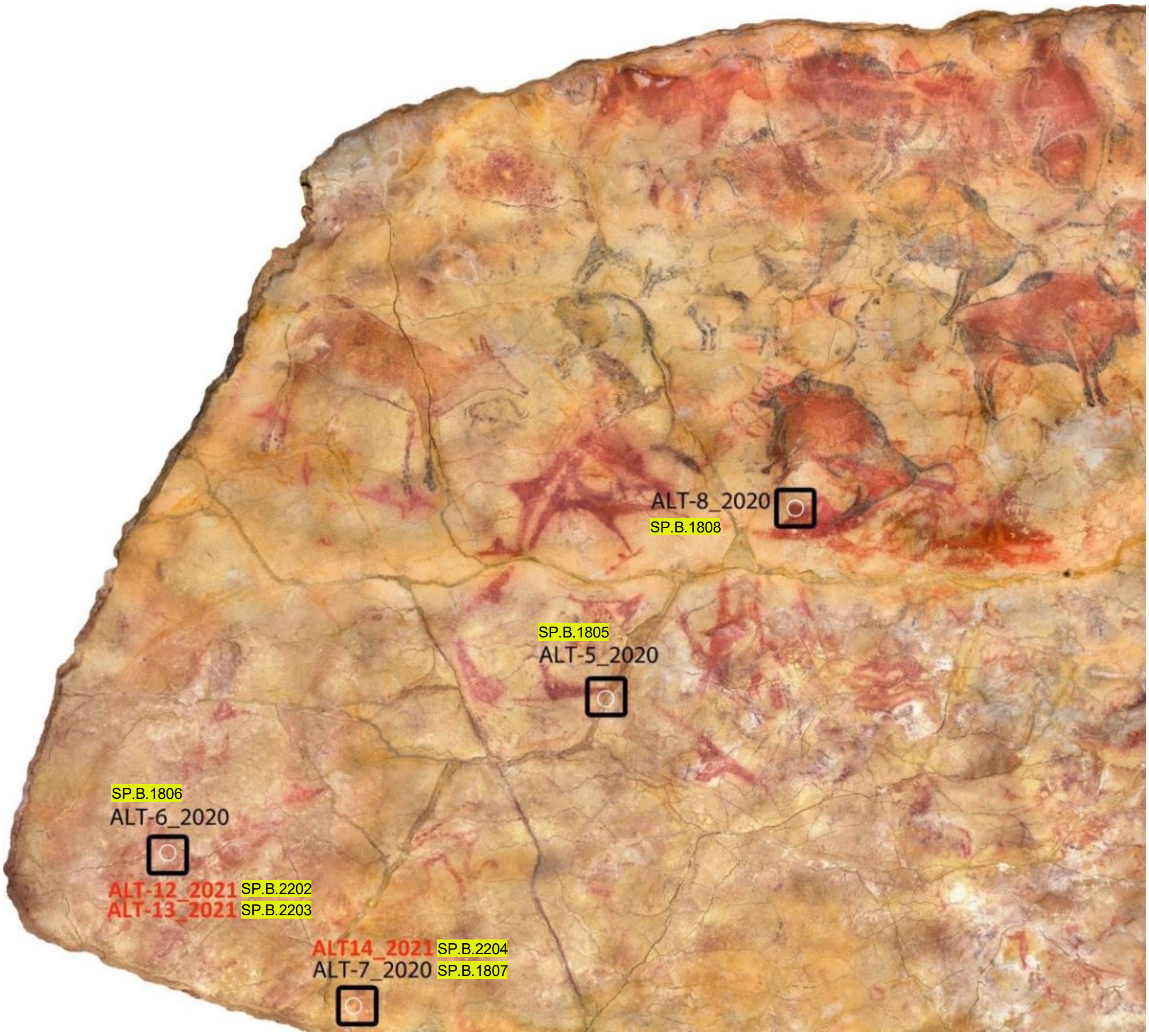

Fig. S1.2. Location of pigment samples sent for DNA analysis from the Sector IV Polychrome ceiling (2020 samples labeled in black (SP.B.1805-08); 2021 samples labeled in red (SP.B.2202-04)).

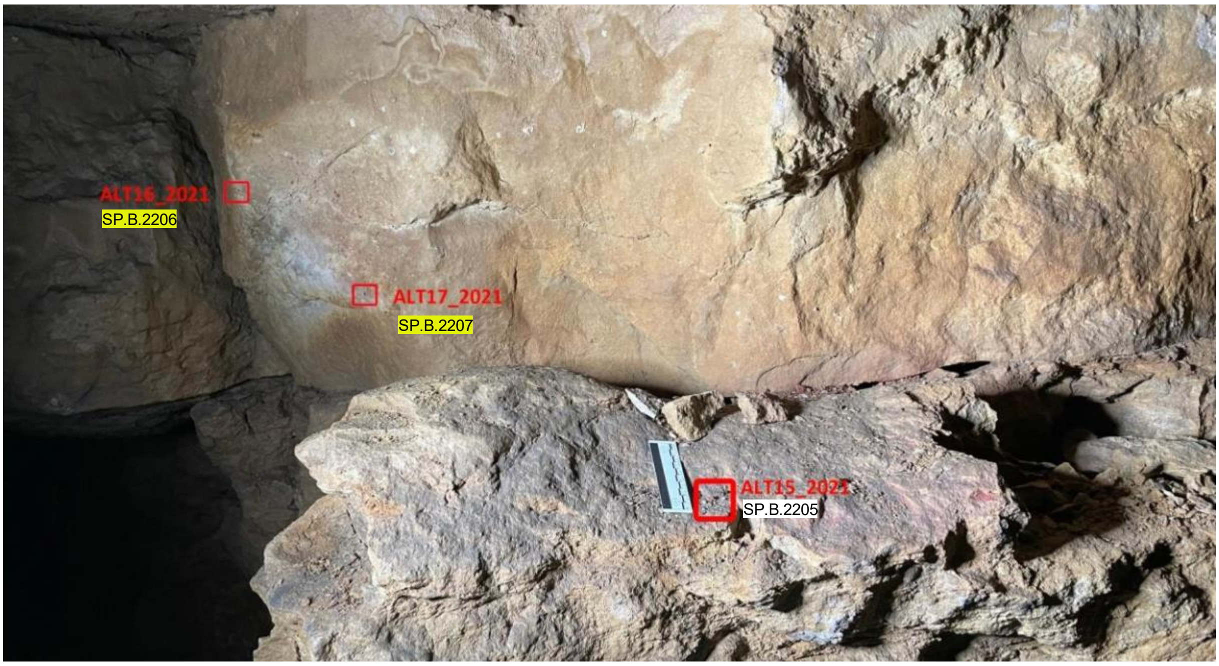

Fig. S1.3. Location of pigment samples sent for DNA analysis from Sector V in 2021 (SP.B.2205-07).

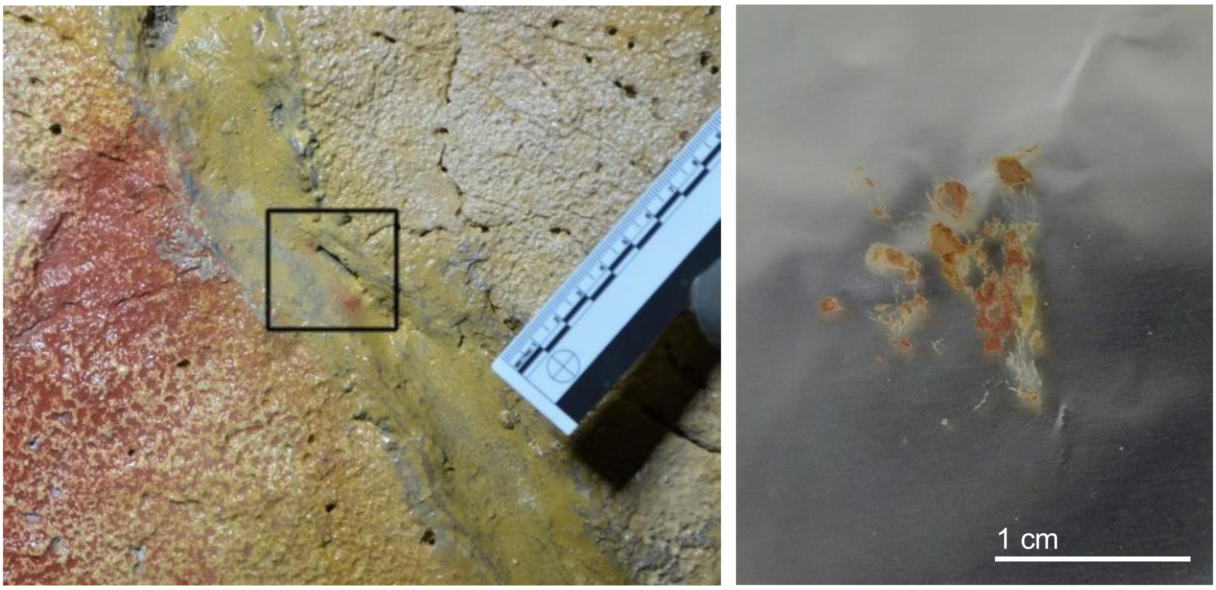

Fig. S1.4. Pigment sample SP.B.1805 in the field (left) and as received by the laboratory (right).

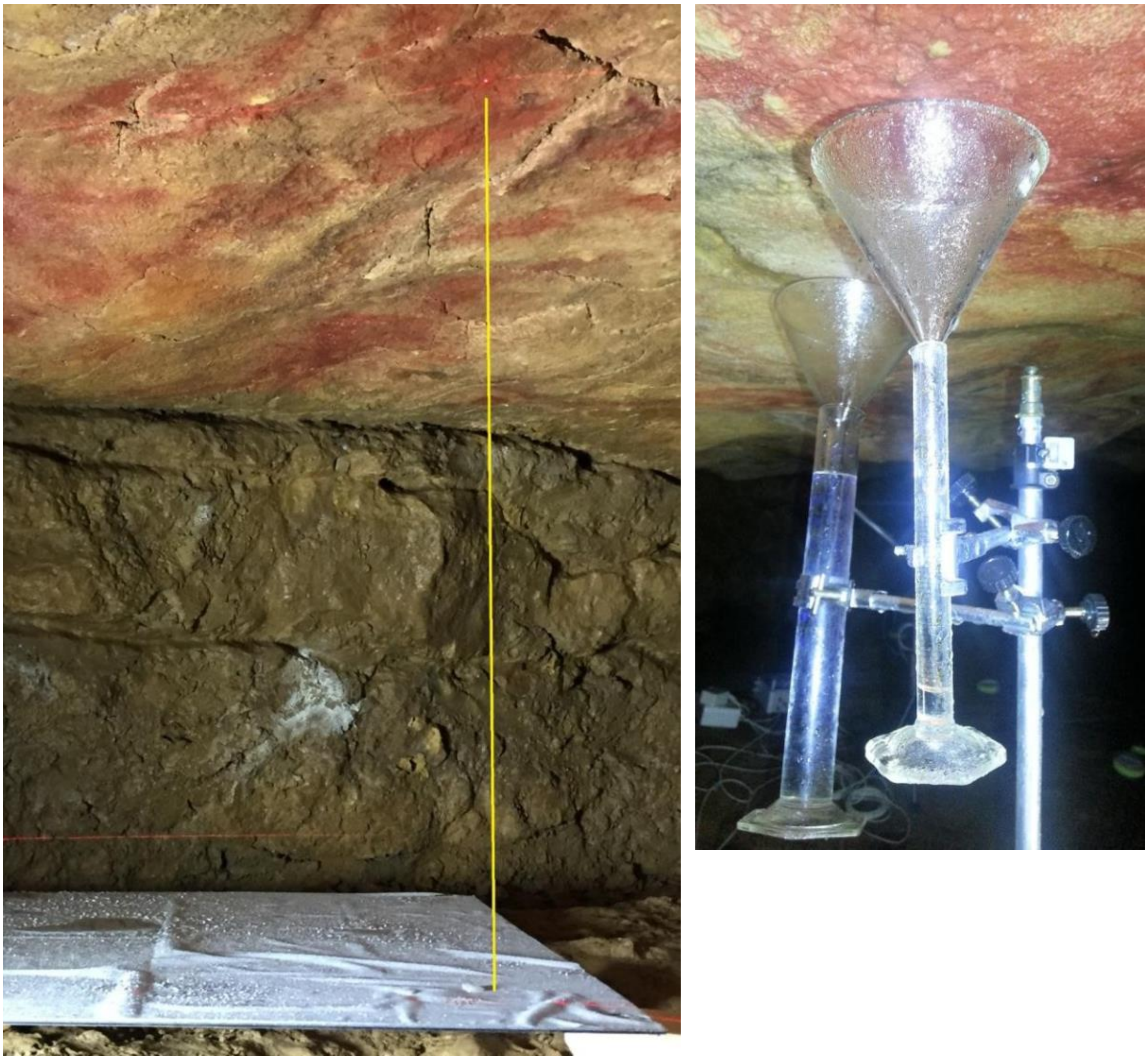

Fig. S1.5. Passive pigment collection set-ups. Collection set-up used for pigment samples SP.B.1806 & SP.B.1807 (left); set-up used to collect pigment sample SP.B.1808 (right).

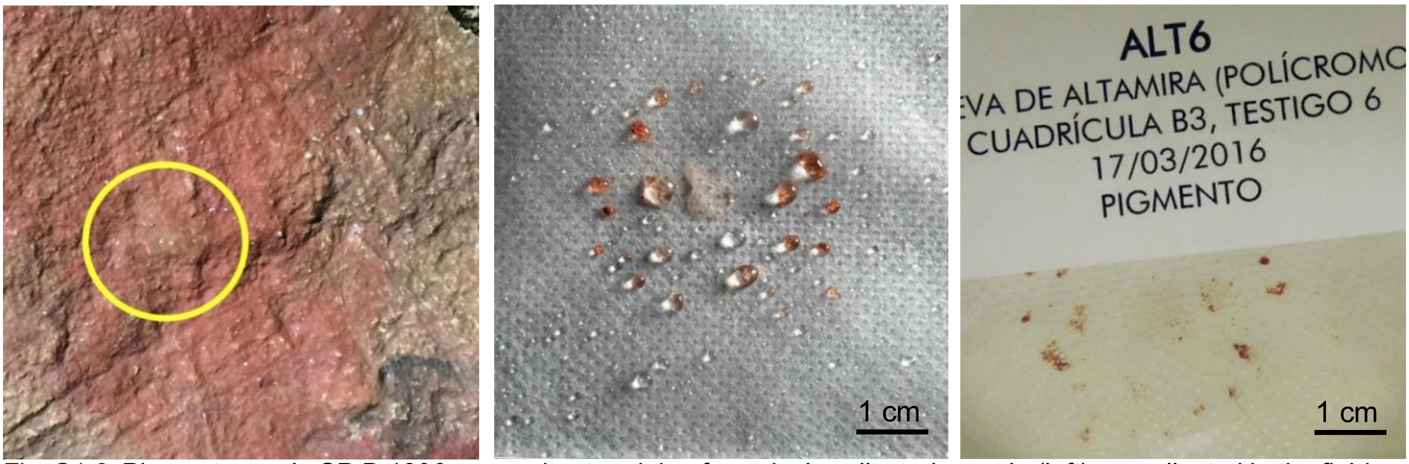

Fig. S1.6. Pigment sample SP.B.1806: approximate origin of passively collected sample (left); as collected in the field on the underlayment (middle); as received by the laboratory (right).

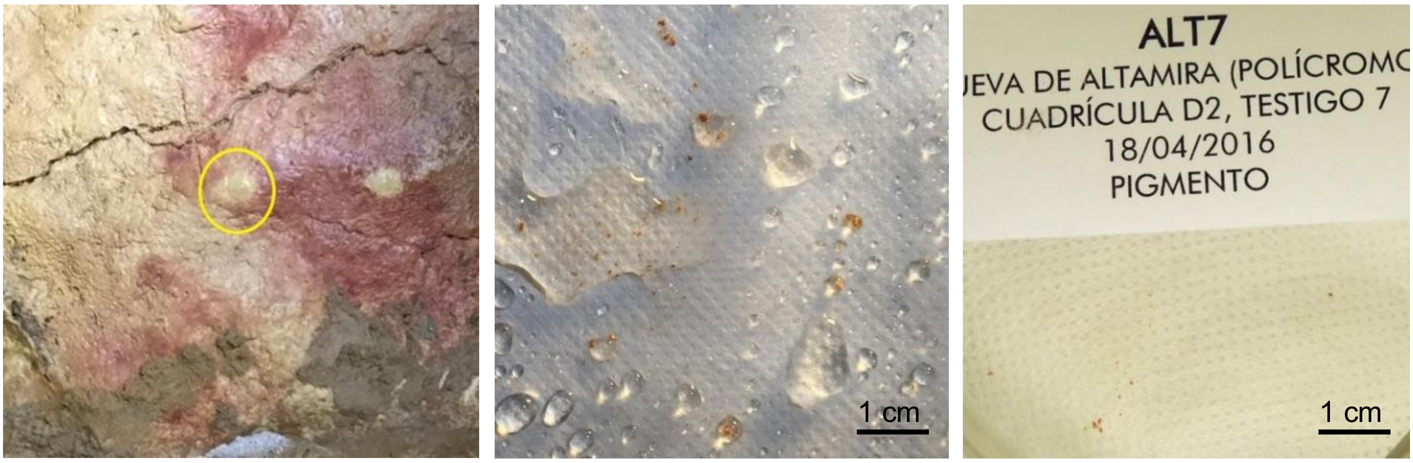

Fig. S1.7. Pigment sample SP.B.1807: approximate origin of passively collected sample (left); as collected in the field on the underlayment (middle); as received by the laboratory (right).

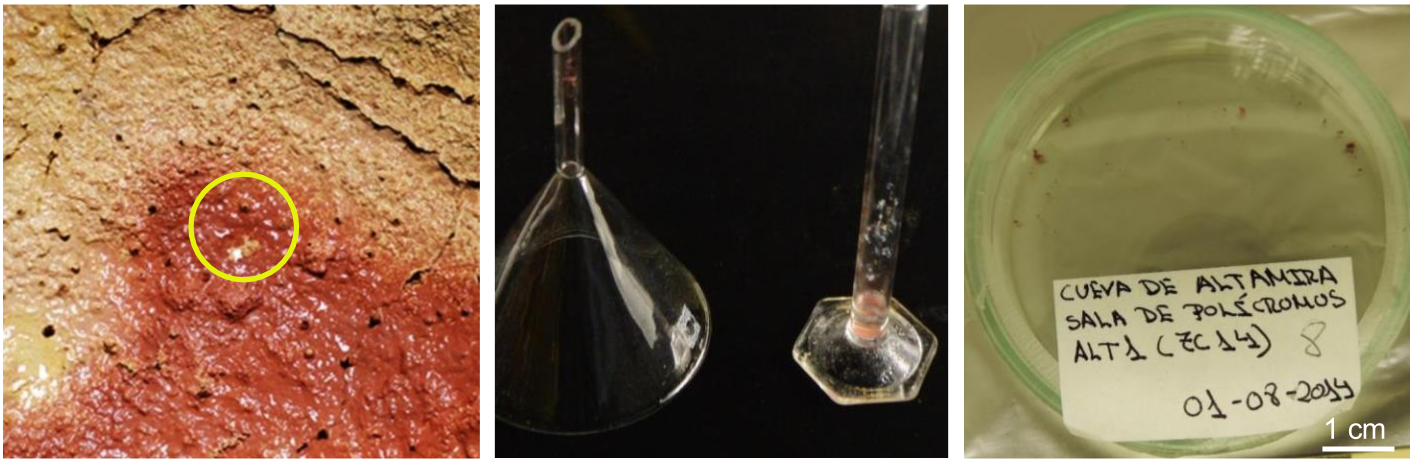

Fig. S1.8. Pigment sample SP.B.1808: approximate origin of passively collected sample (left); as collected from the field in the funnel and graduated cylinder (middle); as received by the laboratory (right).

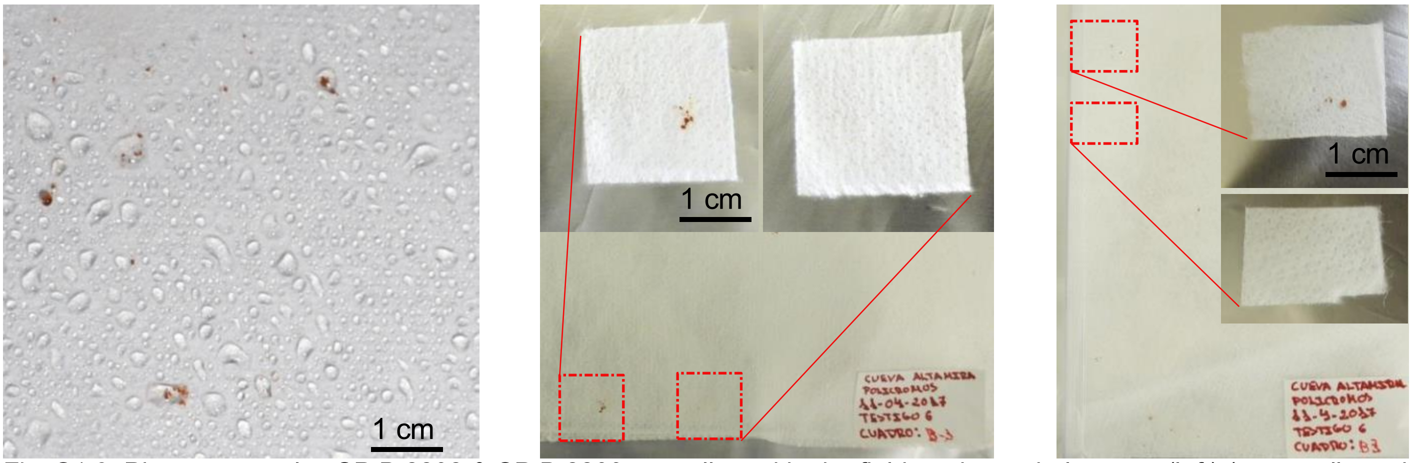

Fig. S1.9. Pigment samples SP.B.2202 & SP.B.2203: as collected in the field on the underlayment (left) (note: collected from the same area as SP.B.1806 of the 2020 campaign); SP.B.2202 as received by the laboratory including negative control cut from the same gauze (middle); SP.B.2203 as received the by laboratory including negative control cut from the same gauze (left).

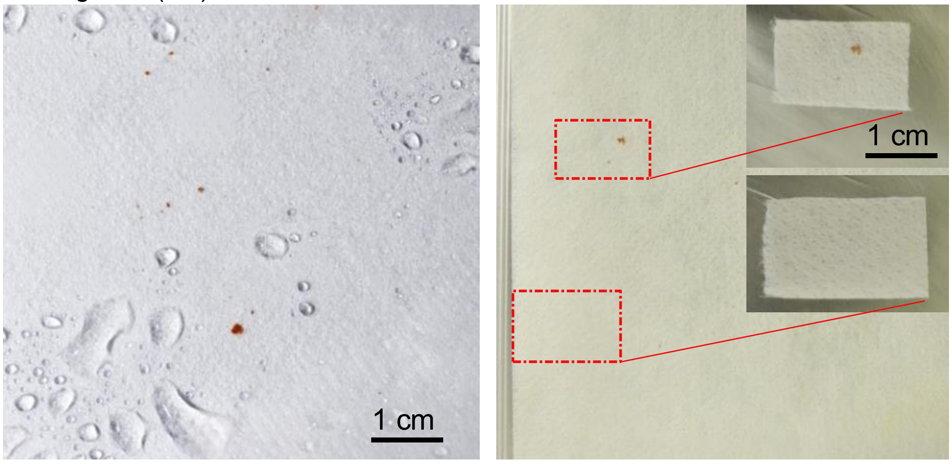

Fig. S1.10. Pigment sample SP.B.2204: as collected in the field on the underlayment (left) (note: collected from the same area as SP.B.1807 of the 2020 campaign); as received by the laboratory including negative control cut from the same gauze (right).

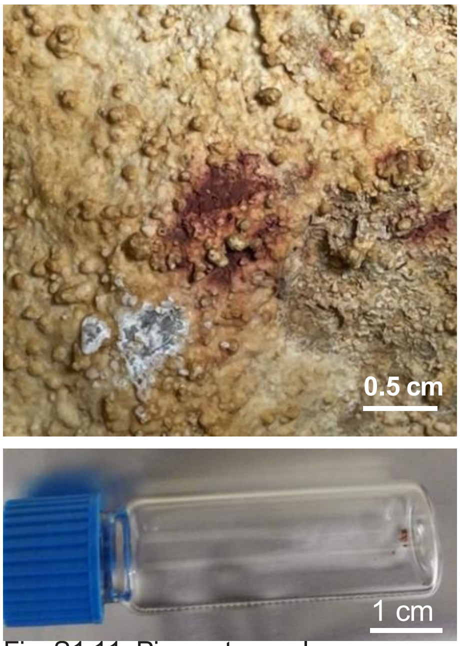

Fig. S1.11. Pigment sample SP.B.2206: cave wall before sampling (upper); sample in tube (lower).

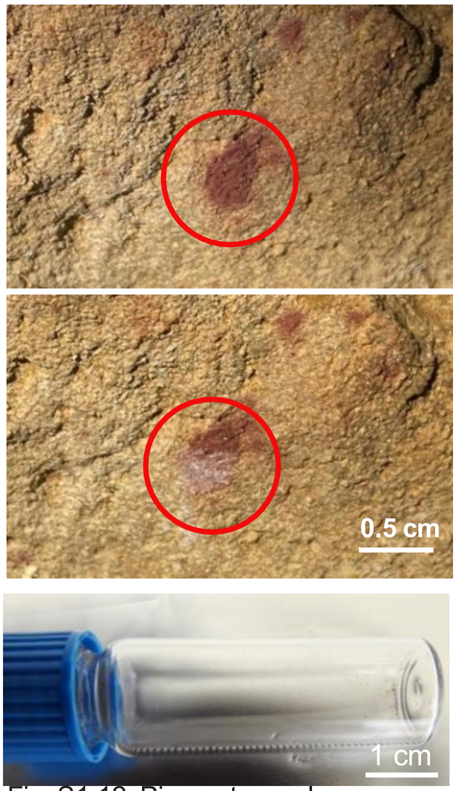

Fig. S1.12. Pigment sample SP.B.2207: cave wall before sampling (upper); after sampling (middle); sample in tube (lower).

Sample ID	Area.	Sample Type
SP.C.6761	CONJUNTO 6. Sample 1	Pigment (Wall)
SP.C.6764	CONJUNTO 6. Sample 4	Pigment (Wall)
SP.C.6765	CONJUNTO 6. Sample 5	Pigment (Wall)
SP.C.6766	CONJUNTO 6. Sample 6	Control (Wall)
SP.C.6770	CONJUNTO 6. Sample 10	Control (Wall)
SP.C.6771	CONJUNTO 6. Sample 11	Control (Wall)
SP.C.6773	CONJUNTO 6. Sample 13	Control (Wall)
SP.C.6775	CONJUNTO 6. Sample 15	Control (Wall)

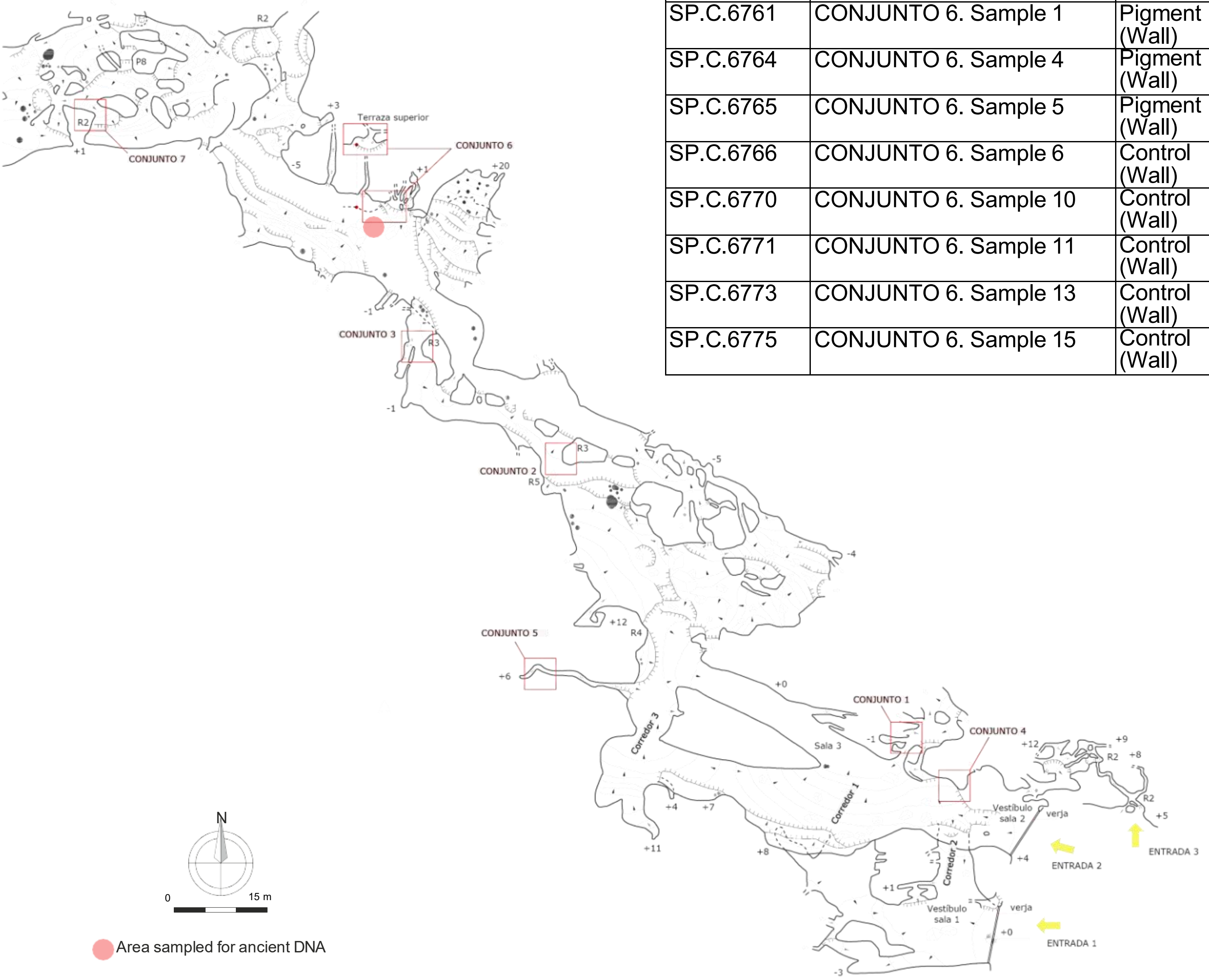

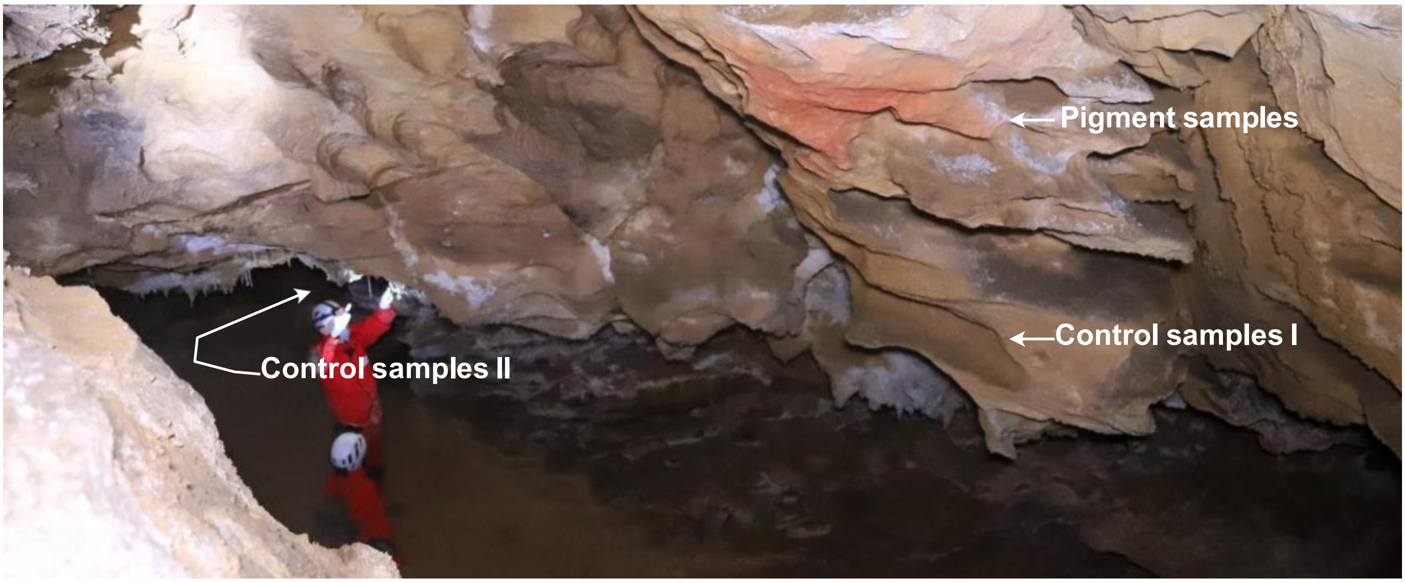

Fig. S2.1. Plan indicating area sampled for DNA analyses in Conjunto 6 of Cueva de Balmori (middle); list of samples screened (upper right); annotated overview of areas sampled (lower).

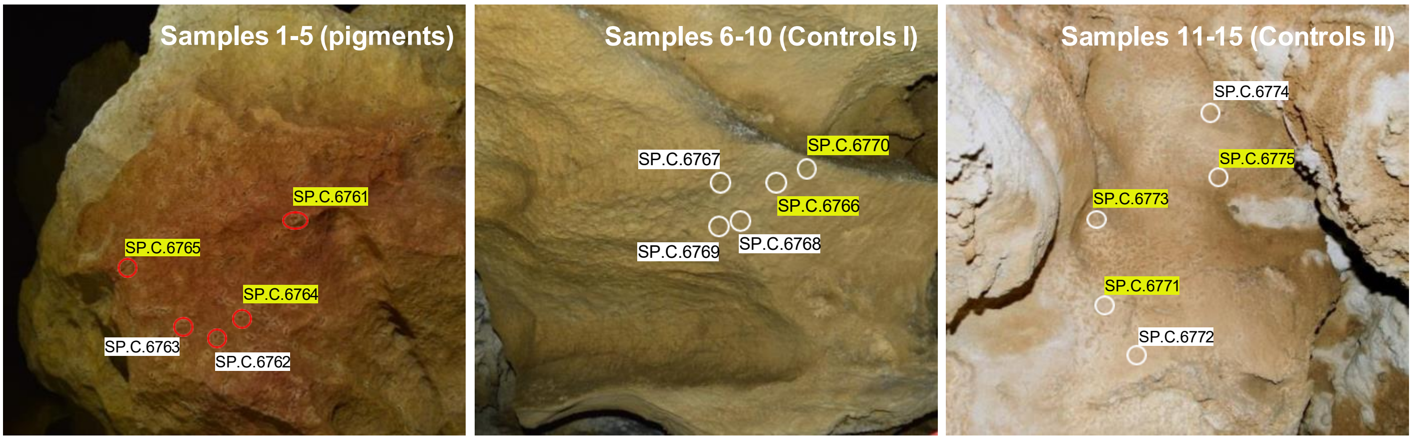

Fig. S2.2. Overview of areas sampled: for pigment sampling (left); for control sampling, “Controls I” (middle) & “Controls II” (right).

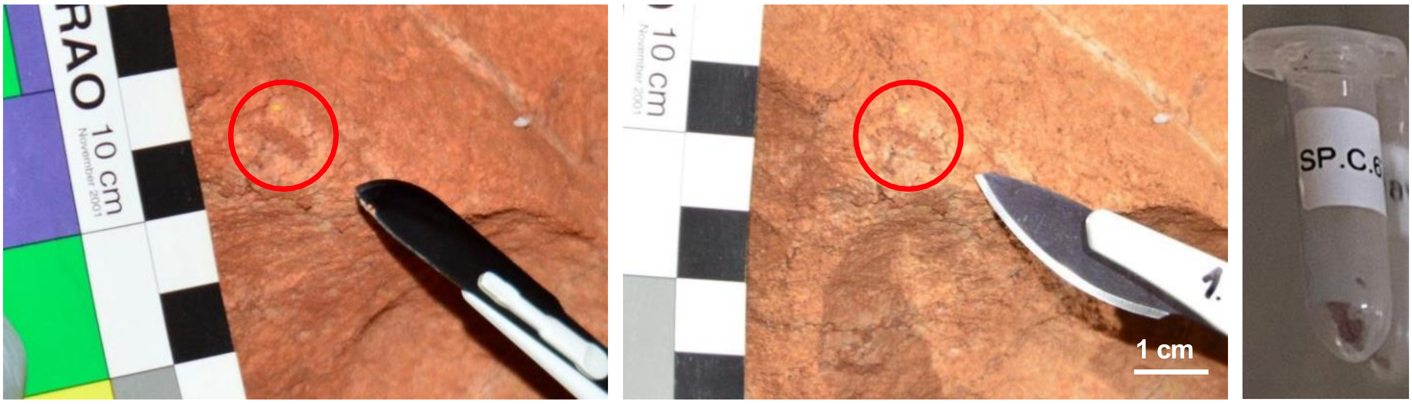

Fig. S2.3. Pigment sample SP.C.6761: cave wall before sampling (left); after sampling (middle); sample in tube (right).

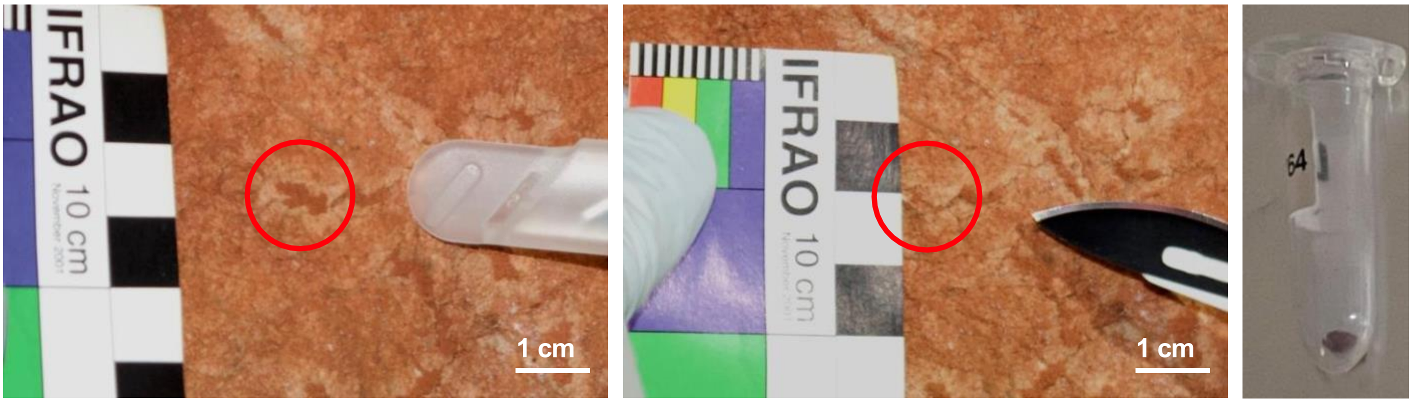

Fig. S2.4. Pigment sample SP.C.6764: cave wall before sampling (left); after sampling (middle); sample in tube (right).

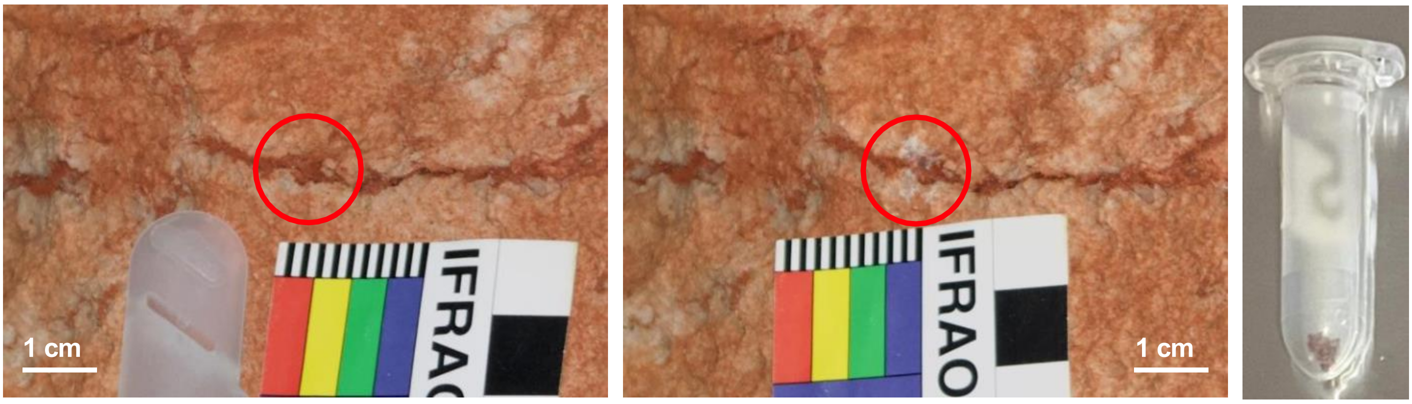

Fig. S2.5. Pigment sample SP.C.6765: cave wall before sampling (left); after sampling (middle); sample in tube (right).

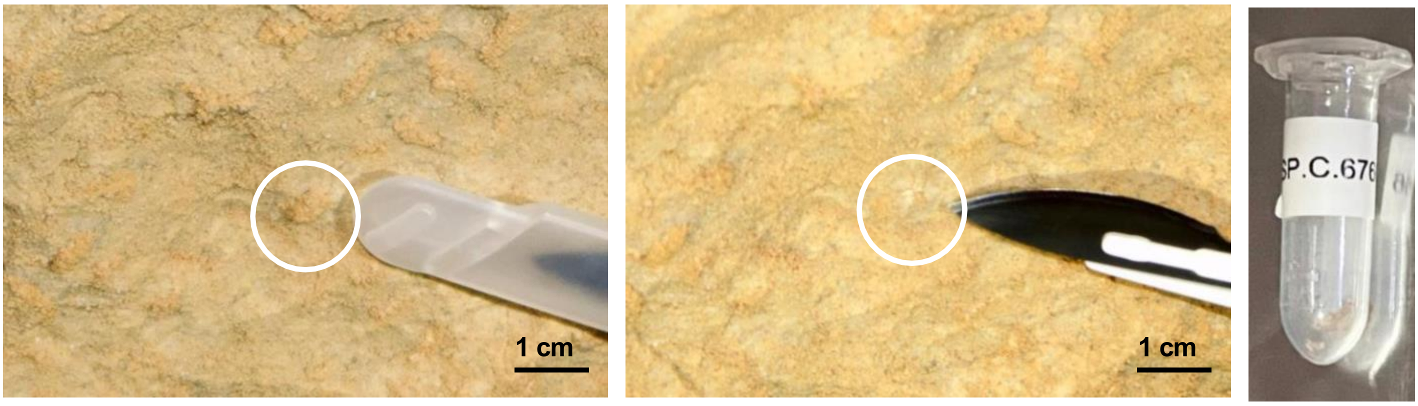

Fig. S2.6. Control sample SP.C.6766: cave wall before sampling (left); after sampling (middle); sample in tube (right).

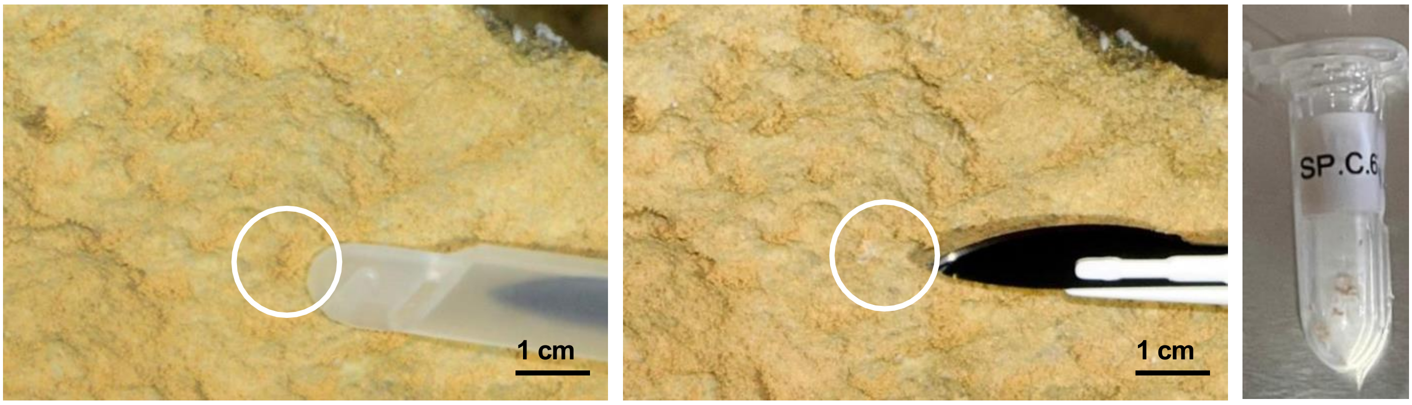

Fig. S2.7. Control sample SP.C.6770: cave wall before sampling (left); after sampling (middle); sample in tube (right).

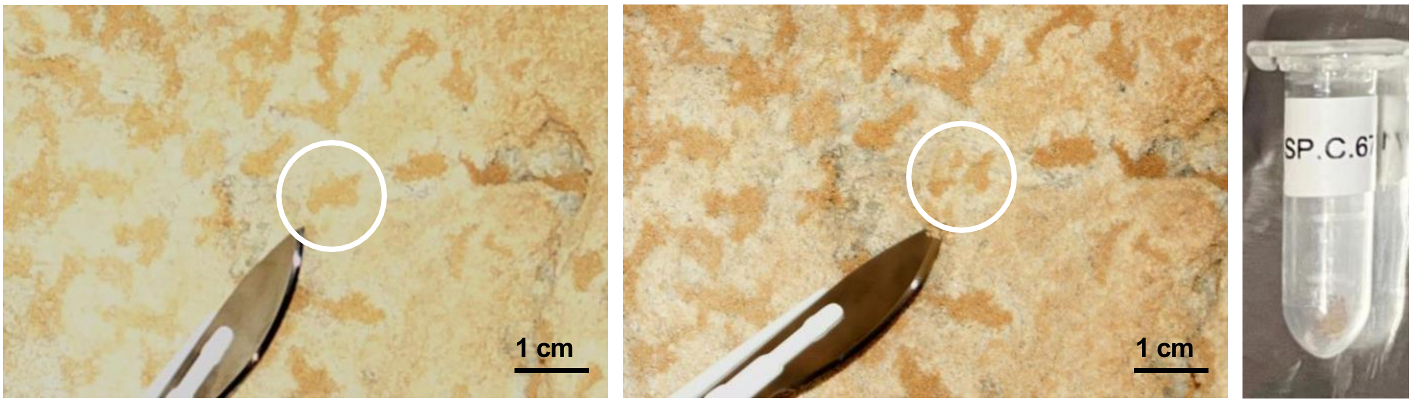

Fig. S2.8. Control sample SP.C.6771: cave wall before sampling (left); after sampling (middle); sample in tube (right).

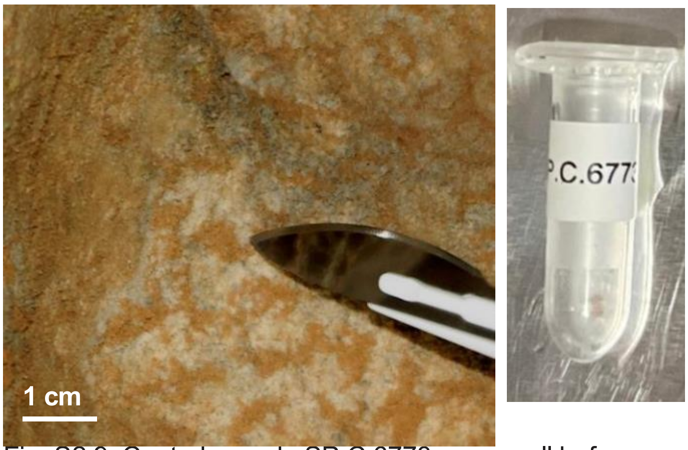

Fig. S2.9. Control sample SP.C.6773: cave wall before sampling (left); sample in tube (right).

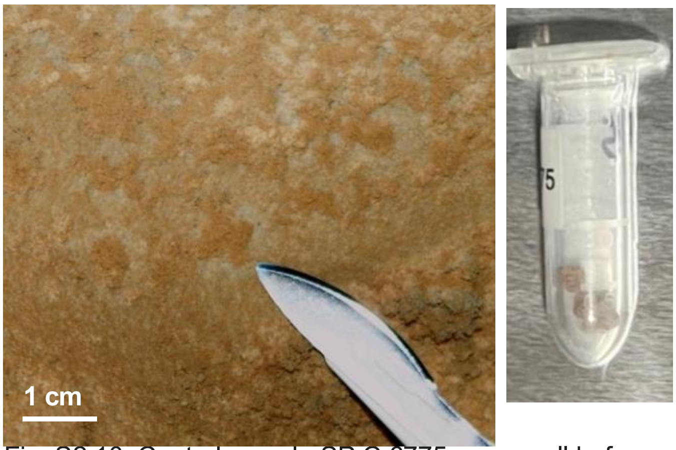

Fig. S2.10. Control sample SP.C.6775: cave wall before sampling (left); sample in tube (right).

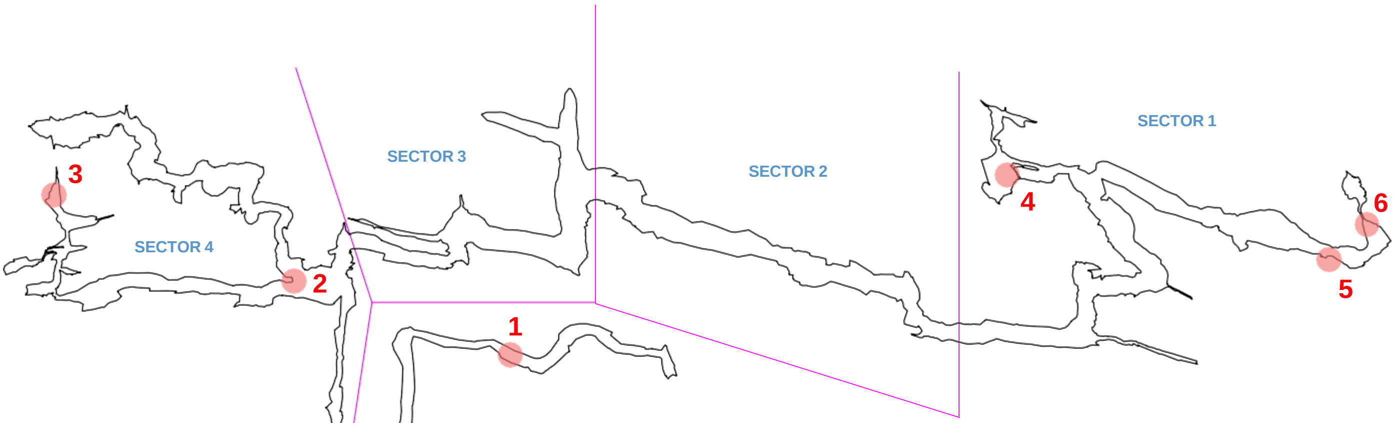

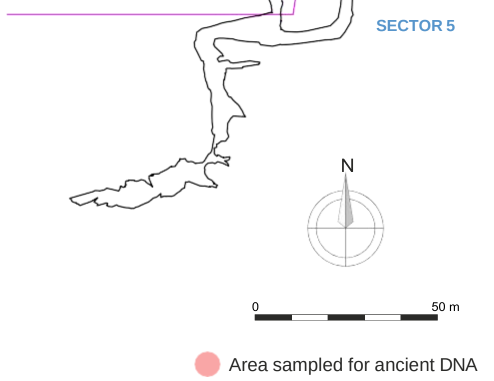

Sample ID	Area	Sample Type
SP.C.5449	Techo de los Puntos	Pigment (Wall)
SP.C.5451	Techo de los Puntos	Control (Wall)
SP.C.5455	Hilera de los Discos	Pigment (Wall)
SP.C.5457	Hilera de los Discos	Control (Wall)
SP.C.5459	Galería SESS	Pigment & Control (Wall)
SP.C.5460	Galería SESS	Control (Wall)
SP.C.5464	Galería SESS	Pigment (Wall)
SP.C.5465	Galería SESS	Control (Wall)
SP.C.5467	Sala de los Signos Rojos	Pigment (Wall)
SP.C.5468	Sala de los Signos Rojos	Control (Wall)

Fig. S3.1. Plan indicating areas sampled for DNA analyses in Cueva de Cudón: Techo de los Puntos (1), Hilera de los Discos (2), Galería SESS (3), Sala de los Signos Rojos (4), and sediment sampling in Sector 1: Profile L7 (5) and Profiles E20 & F22 (6) (upper); list of samples screened (lower).

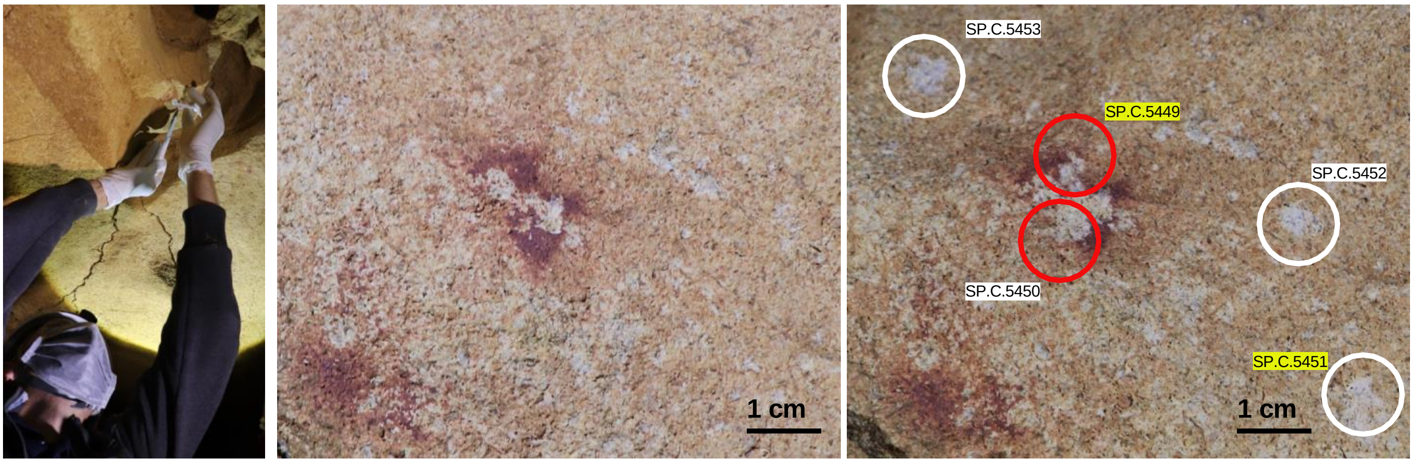

Fig. S3.2. Pigment and control sampling of SP.C.5449 & SP.C.5451: general location (left); cave wall before sampling (middle); after completed sampling with annotation of sampled locations (right).

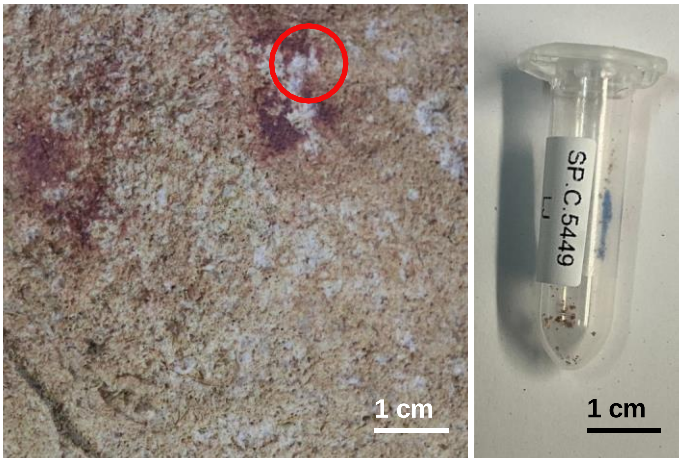

Fig. S3.3. Pigment sample SP.C.5449: cave wall after sampling (left); sample in tube (right).

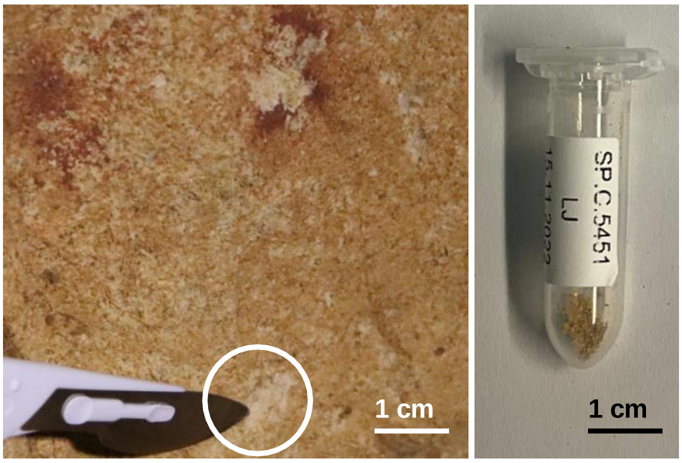

Fig. S3.4. Control sample SP.C.5451: cave wall after sampling (left); sample in tube (right).

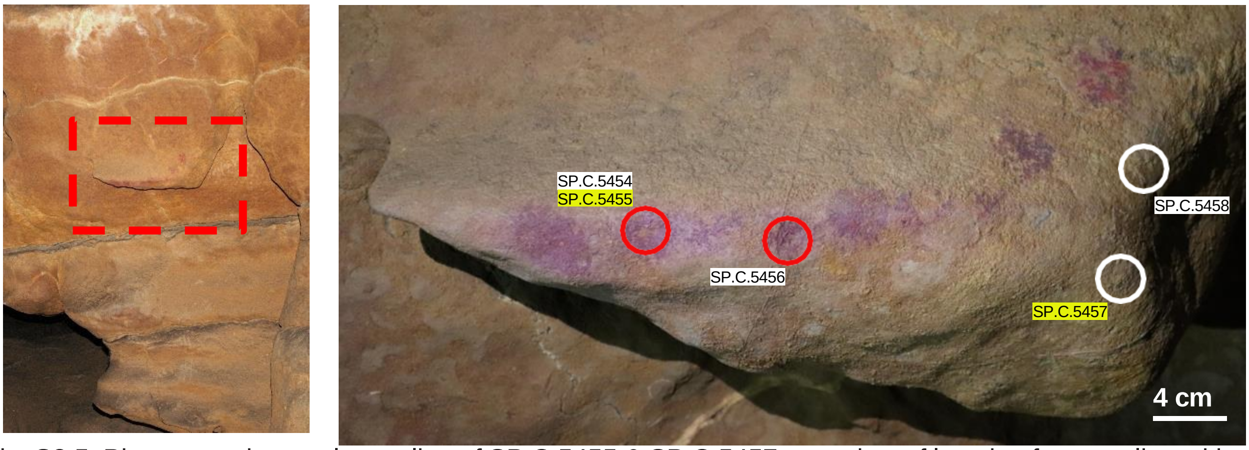

Fig. S3.5. Pigment and control sampling of SP.C.5455 & SP.C.5457: overview of location for sampling with sampled area highlighted in red (left); enlarged view of cave wall before sampling, showing annotated locations of samples (right).

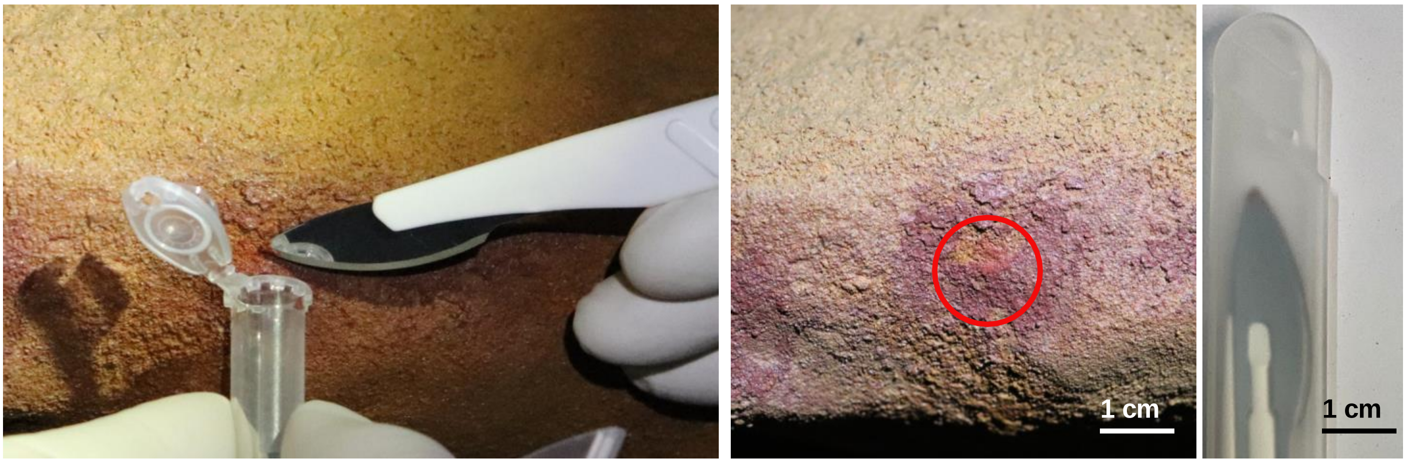

Fig. S3.6. Pigment sample SP.C.5455: cave wall during sampling of existing blemish (left); after sampling (middle); sample on scalpel (right).

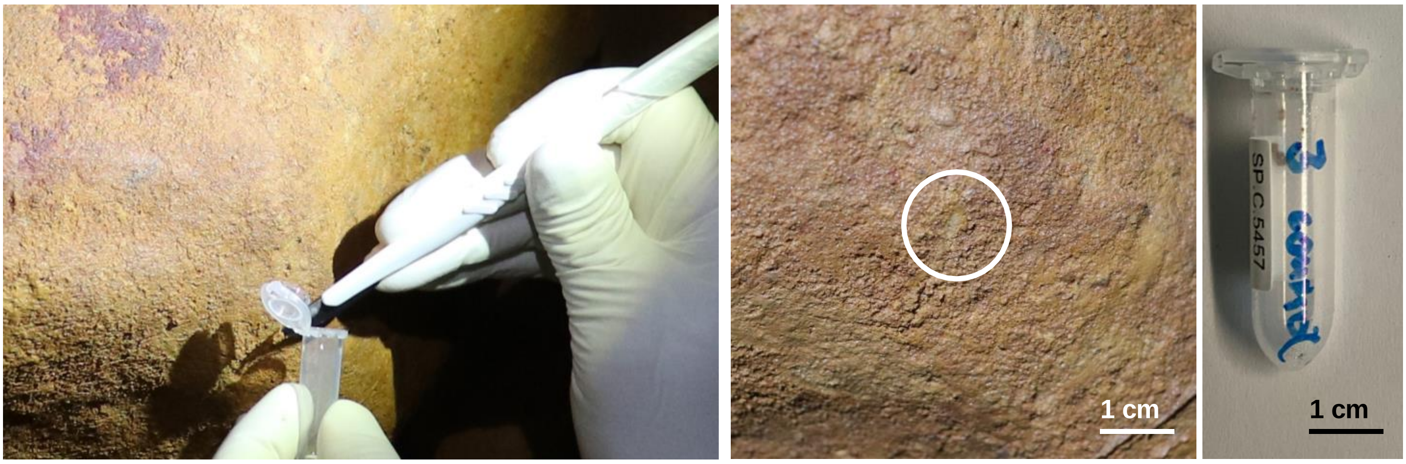

Fig. S3.7. Control sample SP.C.5457: cave wall during sampling (left); after sampling (middle); sample in tube (right).

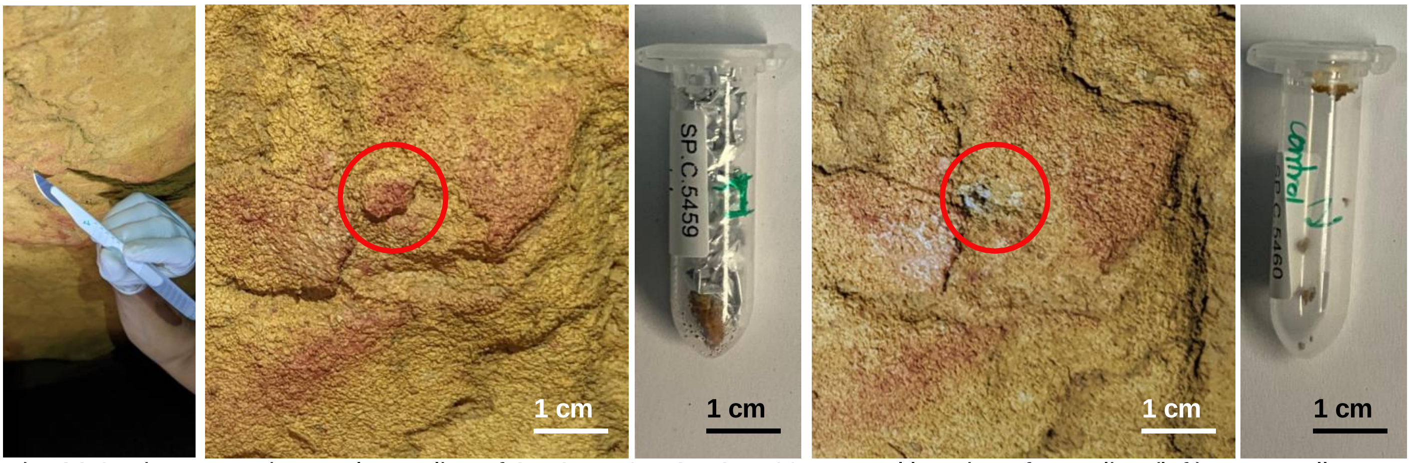

Fig. S3.8. Pigment and control sampling of SP.C.5459 & SP.C.5460: general location of sampling (left); cave wall before pigment sampling - sample in tube (middle); cave wall after control sampling (taken immediately beneath pigment sample) - sample in tube (right).

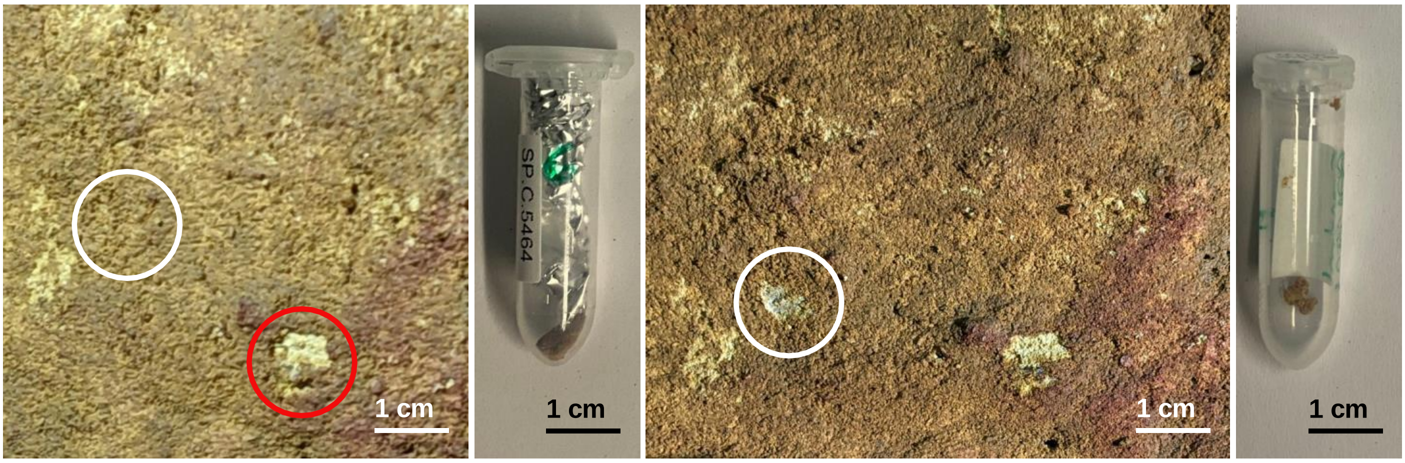

Fig. S3.9. Pigment and control sampling of SP.C.5464 & SP.C.5465: cave wall after pigment sampling/ before control sampling - sample in tube (left); cave wall after control sampling - sample in tube (right).

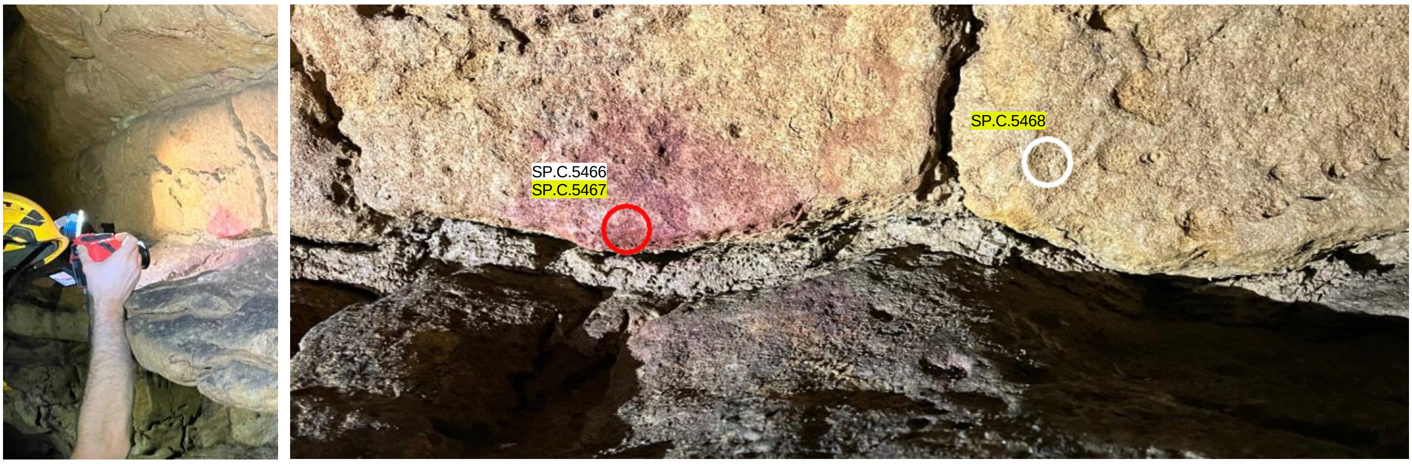

Fig. S3.10. Pigment and control sampling of SP.C.5467 & SP.C.5468: general location (left); before sampling with annotation of sampled locations (right).

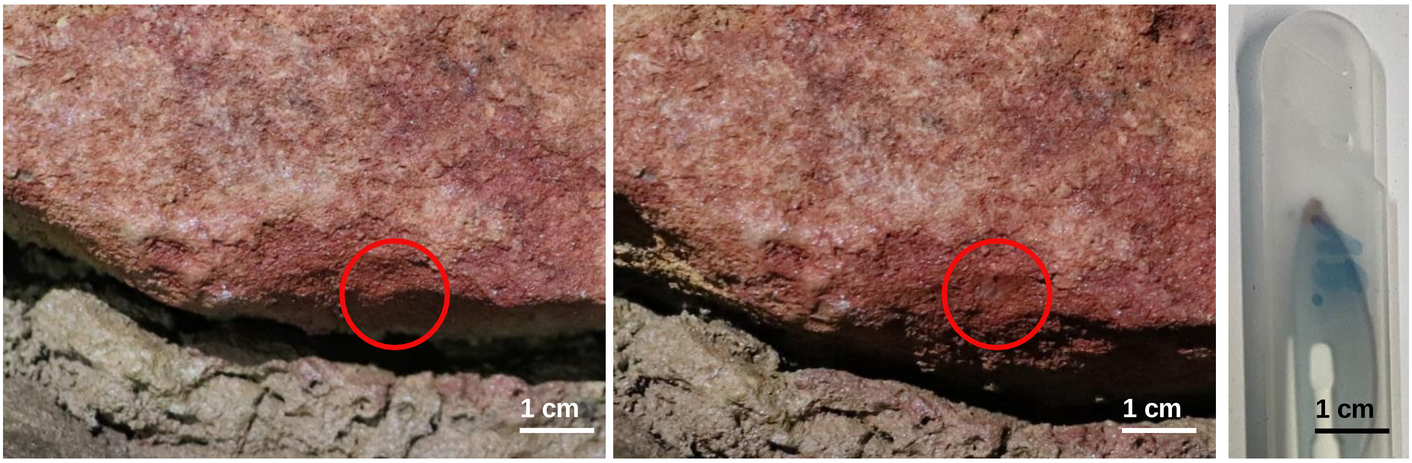

Fig. S3.11. Pigment sample SP.C.5467: cave wall before sampling (left); after sampling (middle); sample on scalpel used for DNA extraction (right).

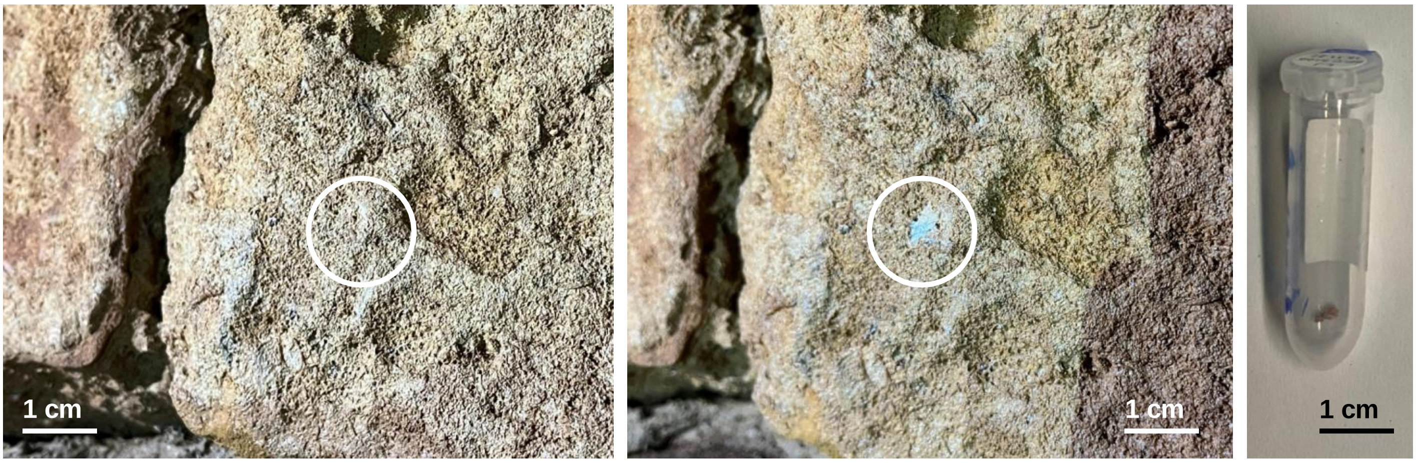

Fig. S3.12. Control sample SP.C.5468: cave wall before sampling (left); after sampling (middle); sample in tube (right).

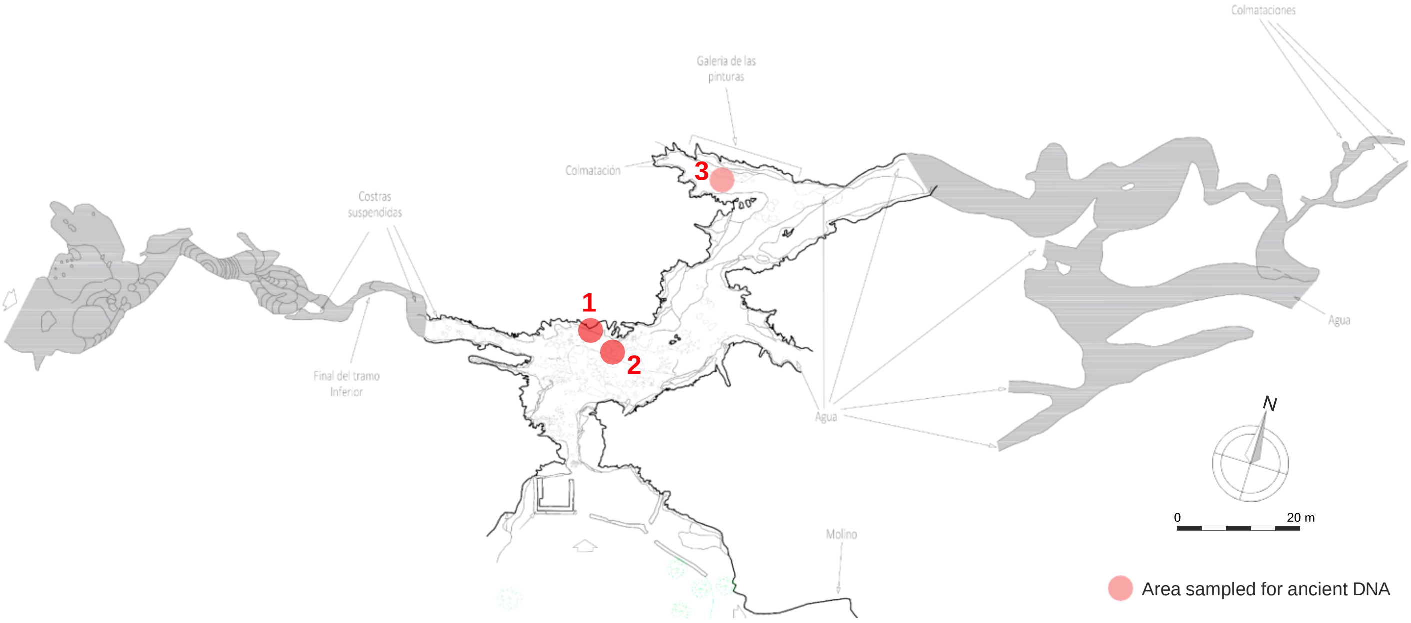

Sample ID	Area	Sample Type
SP.C.5541	Main hall: “Grid” (right)	Pigment (Wall)
SP.C.5542	Main hall: “Grid” (right)	Pigment (Wall)
SP.C.5543	Main hall: “Grid” (left)	Pigment (Wall)
SP.C.5544	Main hall: red stein at left of “Grid”	Pigment (Wall)
SP.C.5546	Main hall: below “left of Grid” sample	Control (Wall)
SP.C.5547	Main hall: below “left of Grid” sample	Control (Wall)

Sample ID	Area	Sample Type
SP.C.5556	Galeria de las Pinturas	Pigment (Passive)
SP.C.5558	Galeria de las Pinturas	Pigment (Passive)
SP.C.5560	Galeria de las Pinturas	Pigment (Passive)
SP.C.5562	Galeria de las Pinturas	Pigment (Passive)
SP.C.5564	Galeria de las Pinturas	Control (Floor)
SP.C.5565	Galeria de las Pinturas	Control (Floor)
SP.C.5566	Galeria de las Pinturas	Sediment
SP.C.5567	Galeria de las Pinturas	Sediment
SP.C.5568	Galeria de las Pinturas	Sediment
SP.C.5569	Galeria de las Pinturas	Sediment Control
SP.C.5570	Galeria de las Pinturas	Sediment Control

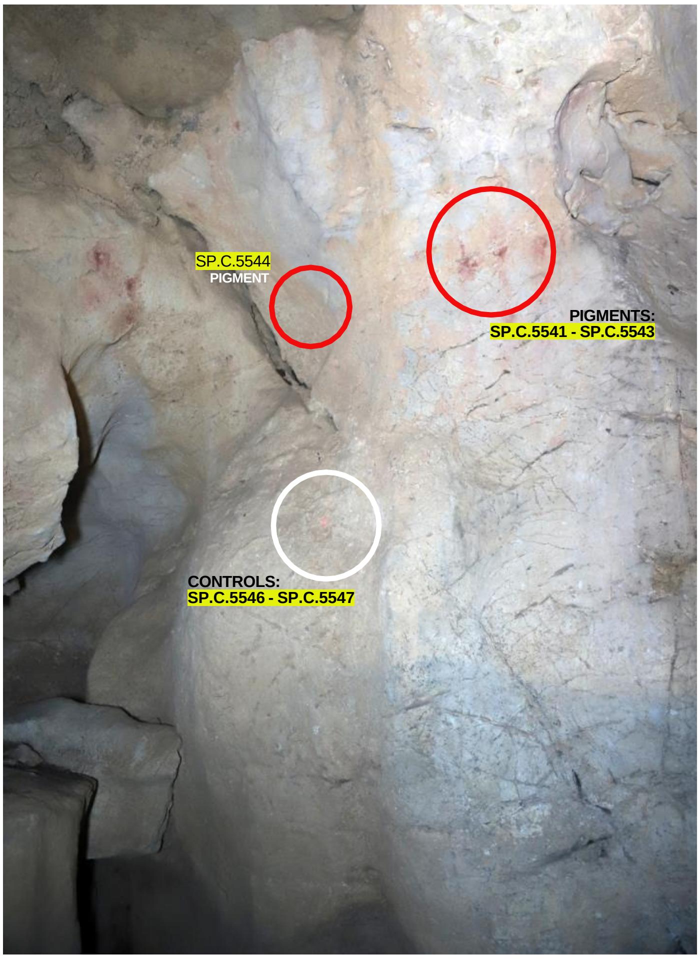

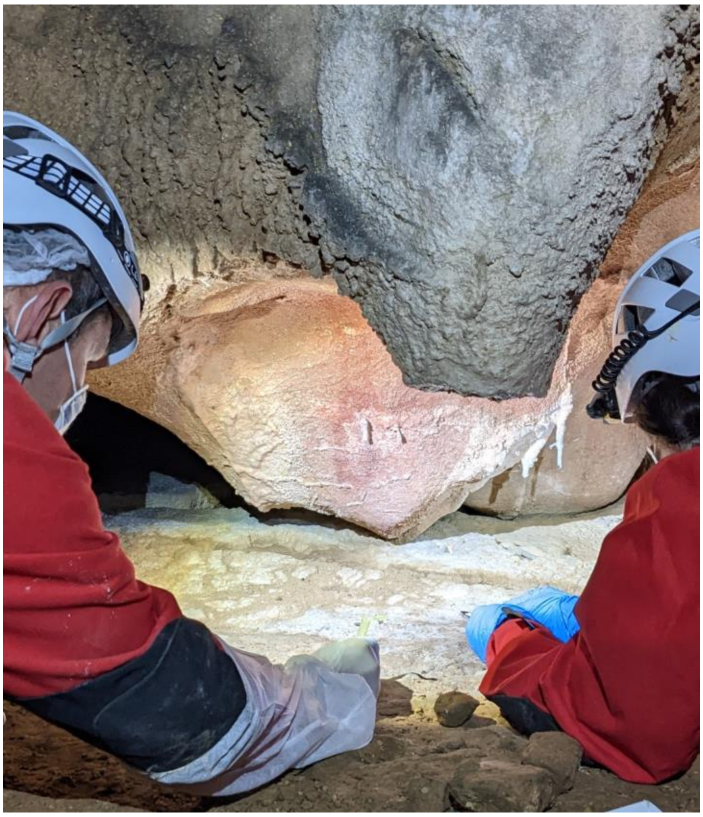

Fig. S4.1. Plan indicating areas sampled for DNA analyses at Cueva del Covarón: Main hall Panel 1 (1), sediment sampling in Main hall (2), and Galería de las Pinturas (3) (upper). List of samples screened for ancient DNA (middle). Overview of the sampled areas in the Main hall indicating areas of pigment vs. control samples (lower left). Overview of the area sampled for pigment flakes in Galería de las Pinturas (lower right).

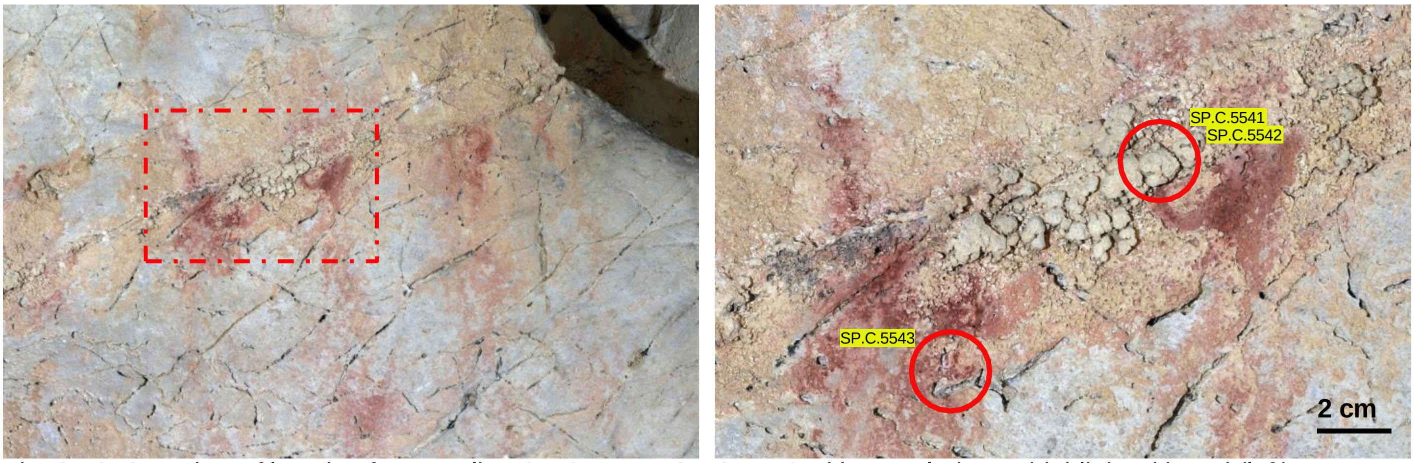

Fig. S4.2. Overview of location for sampling SP.C.5541 - SP.C.5543 with sampled area highlighted in red (left); cave wall before sampling (right).

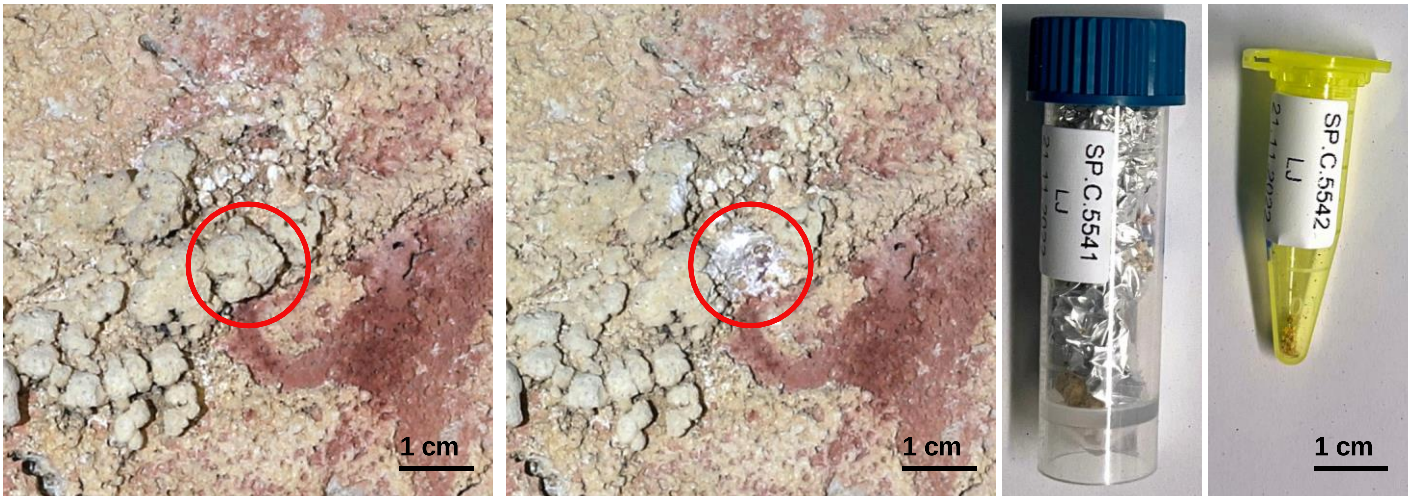

Fig. S4.3. Pigment samples SP.C.5541/ SP.C.5542: cave wall before sampling (left); after sampling (middle); samples in tubes (right). Note: Pigment sample SP.C.5542 was not sampled directly, rather the debris from sampling SP.C.5541 was caught on clean aluminum foil and collected in a sterile tube.

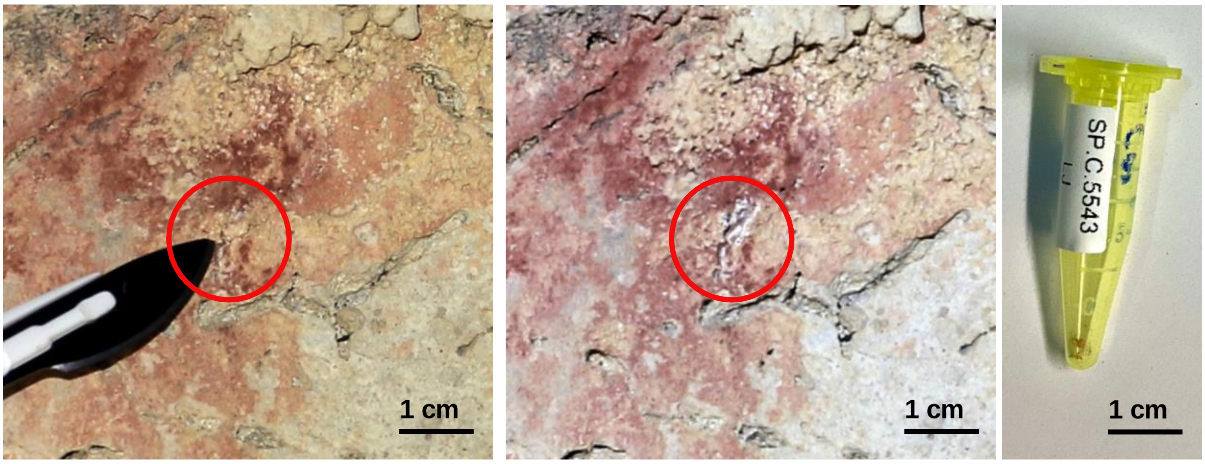

Fig. S4.4. Pigment sample SP.C.5543: cave wall before sampling (left); after sampling (middle); sample in tube (right).

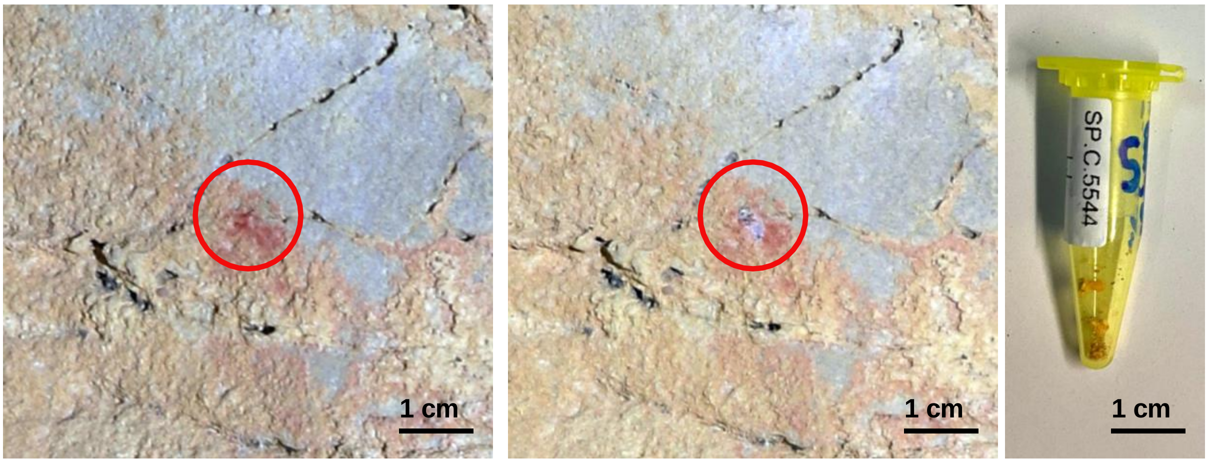

Fig. S4.5. Pigment sample SP.C.5544: cave wall before sampling (left); after sampling (middle); sample in tube (right).

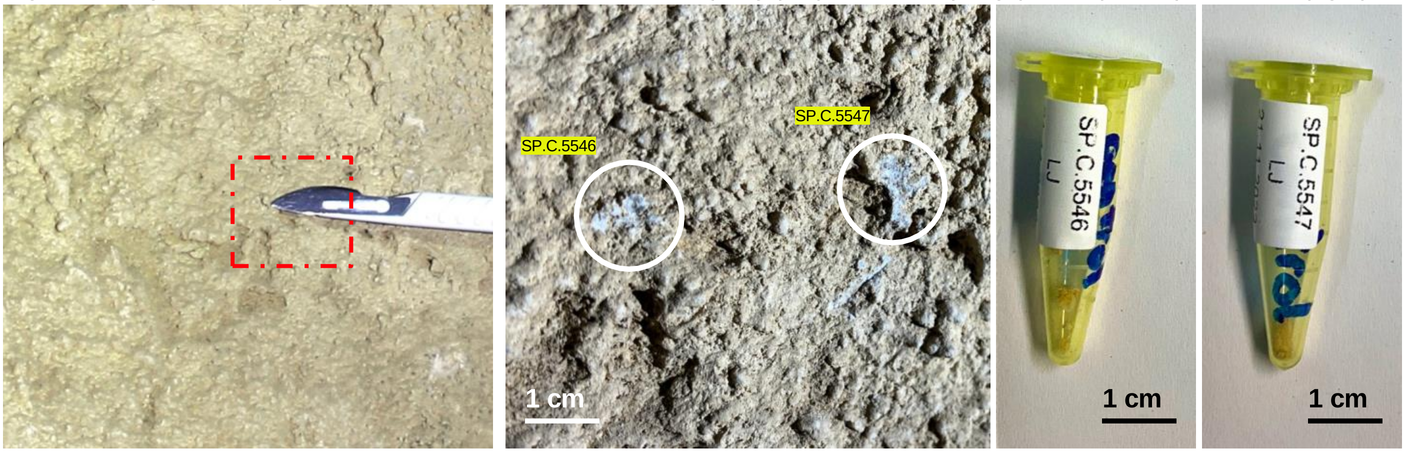

Fig. S4.6. Control samples SP.C.5546 & SP.C.5547: cave wall before sampling with sampled area highlighted in red (left); enlarged view after sampling (middle); samples in tubes (right).

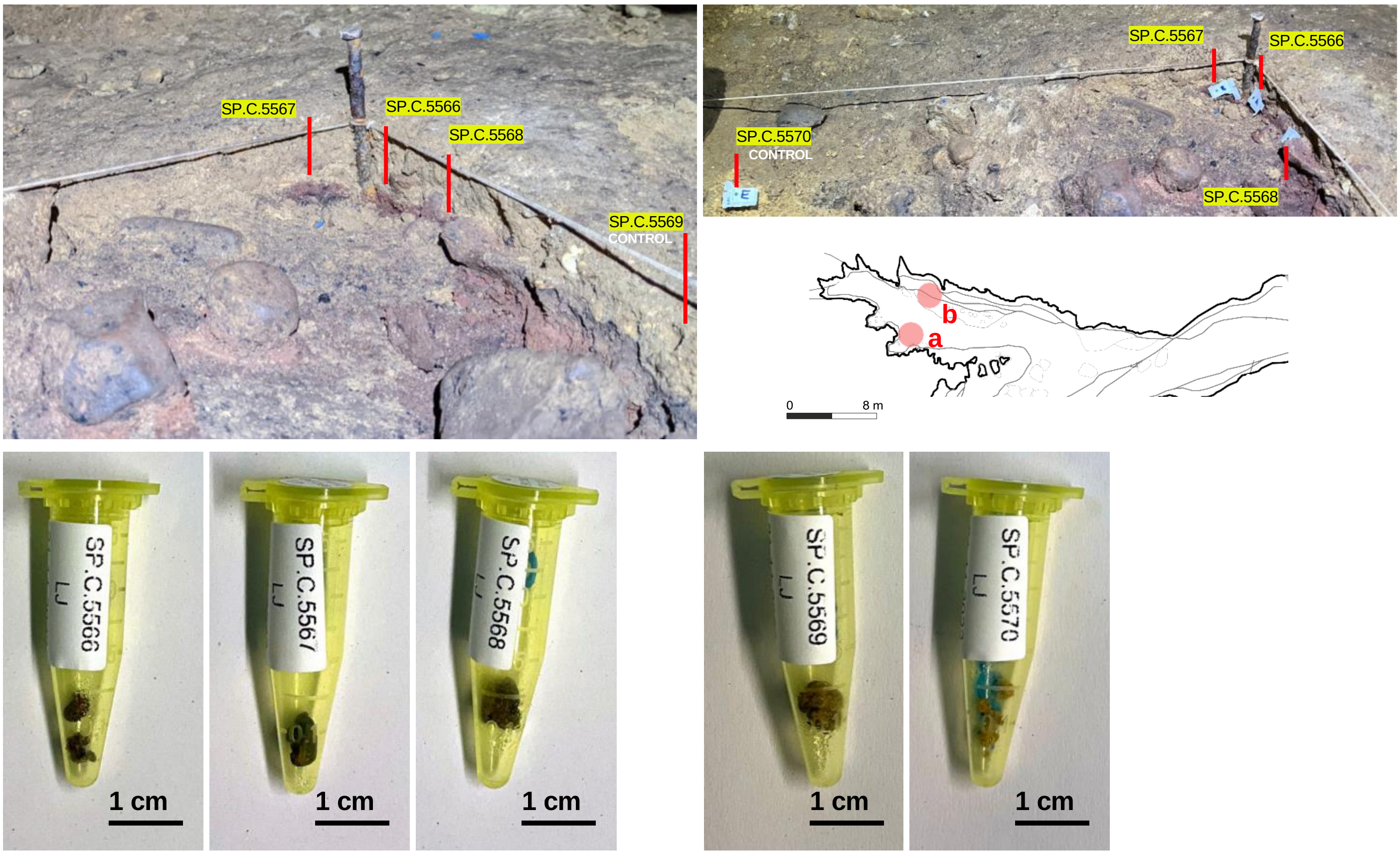

Fig. S4.7. Pigment and control samples in sediment SP.C.5566 - SP.C.5570: close up of area before sampling (control sample SP.C.5570 not shown) (upper left); after sampling, indicating areas of pigment vs. control samples (control sample SP.C.5569 not shown) (upper right); plan indicating areas sampled in Galería de las Pinturas: wall pigment (a); pigment in sediment (b) (middle right); samples in tubes (lower).

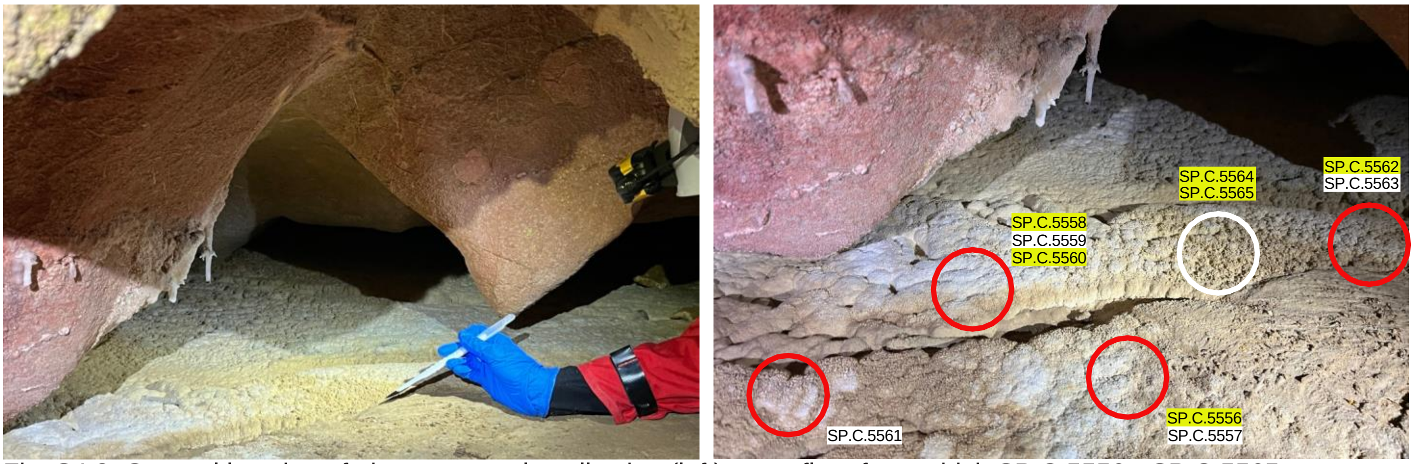

Fig. S4.8. General location of pigment sample collection (left); cave floor from which SP.C.5556 - SP.C.5565 were collected (right) - flakes of pigment collected from areas circled in red; control samples taken from area circled in white.

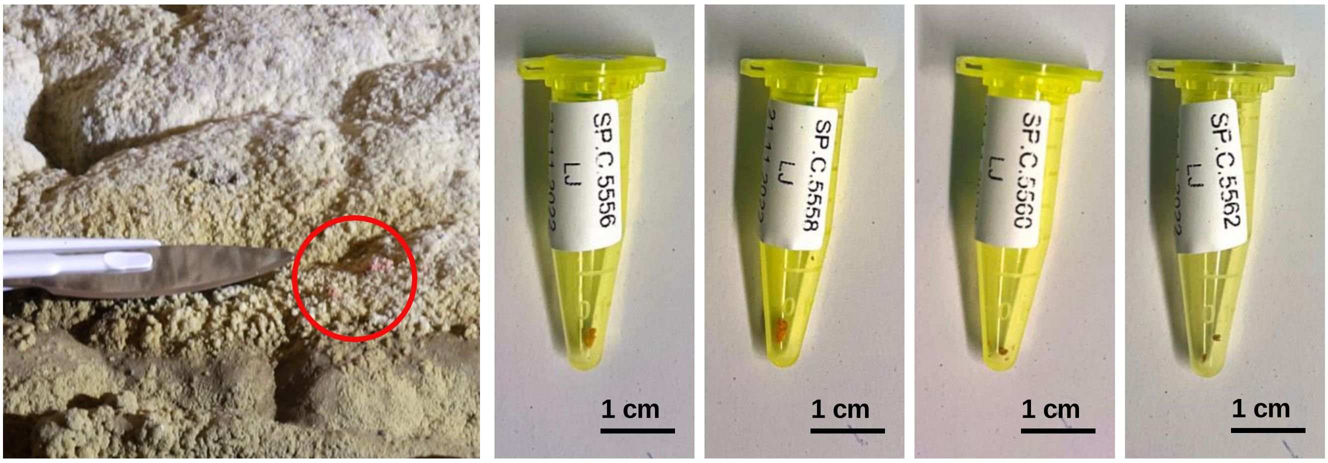

Fig. S4.9. Representative example of pigment flakes found on the cave floor and collected for SP.C.5556 - SP.C.5563 (left); samples in tubes (right).

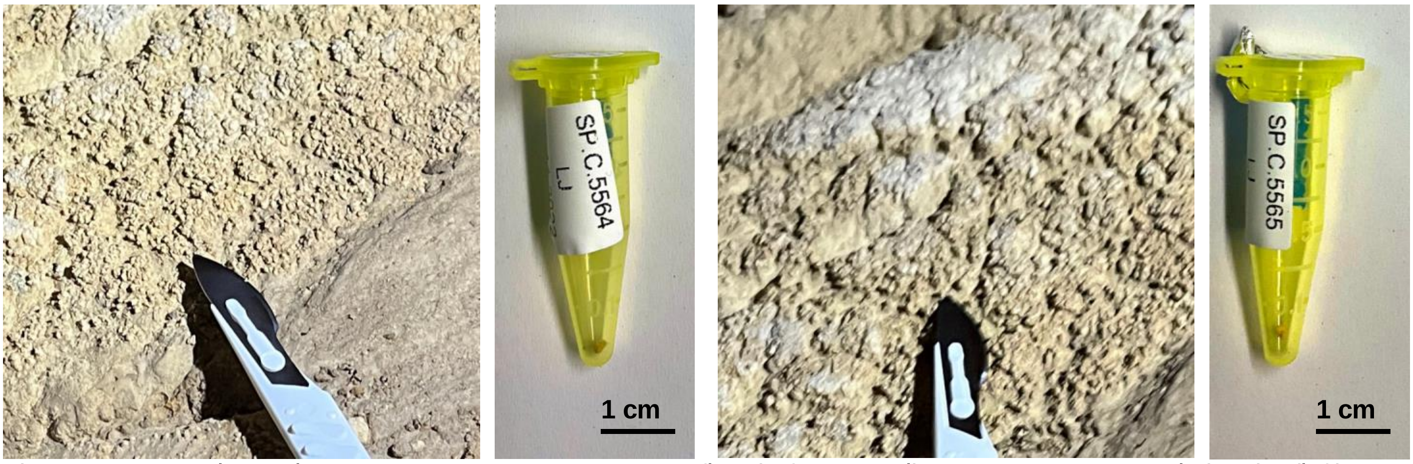

Fig. S4.10. Control samples SP.C.5564 & SP.B.5565: cave floor before sampling SP.C.5564 & sample in tube (left); cave floor before sampling SP.C.5565 & sample in tube (right).

Sample ID	Area	Sample Type
SP.C.6844	Panel B-VIII “Angular Sign”	Pigment (Wall)
SP.C.6845	Panel B-VIII “Angular Sign”	Pigment (Wall)
SP.C.6846	Panel B-VIII “Angular Sign”	Control (Wall)
SP.C.6847	Panel B-VIII “Angular Sign”	Control (Wall)
SP.C.6848	Panel B-VIII “Angular Sign”	Control (Wall)

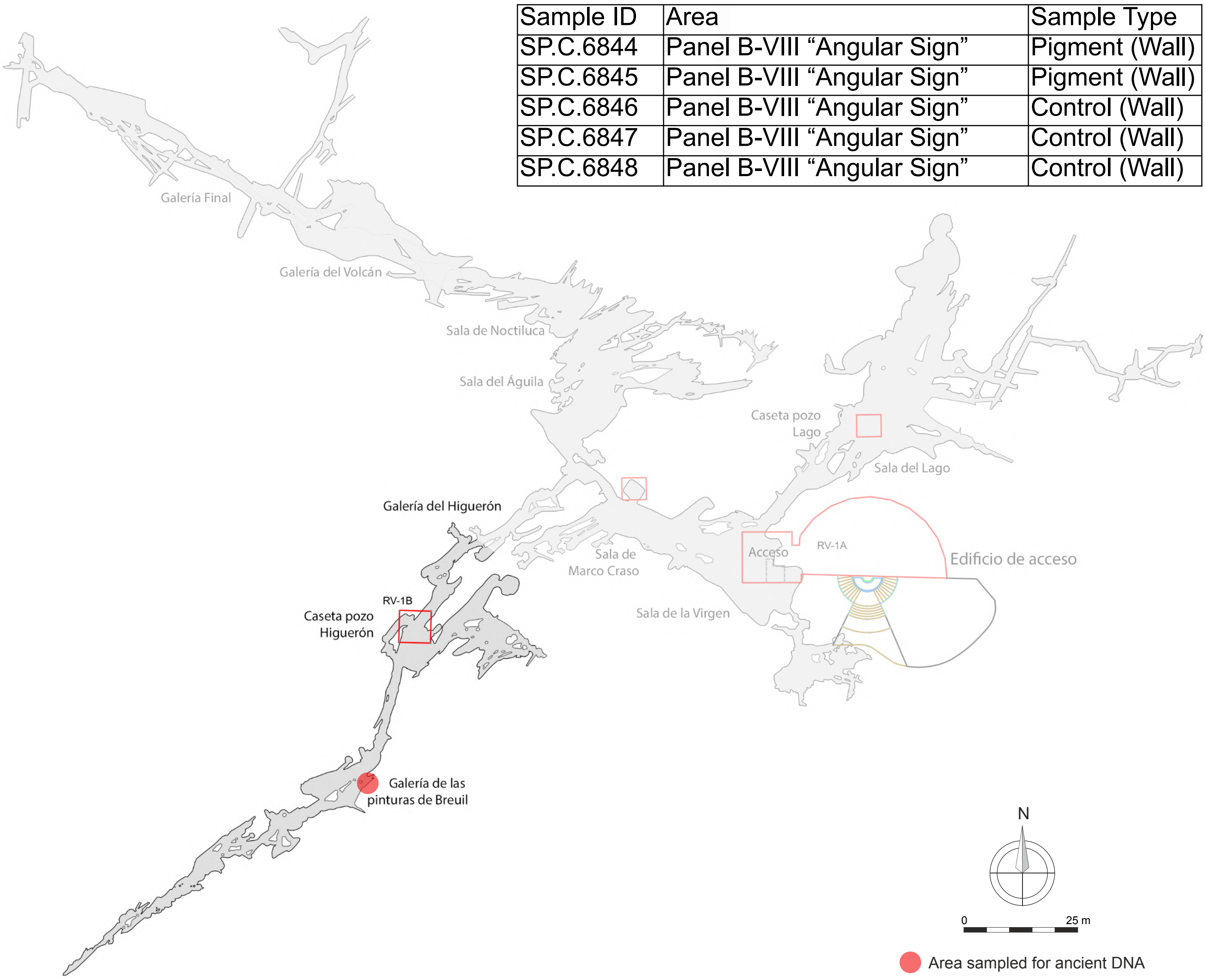

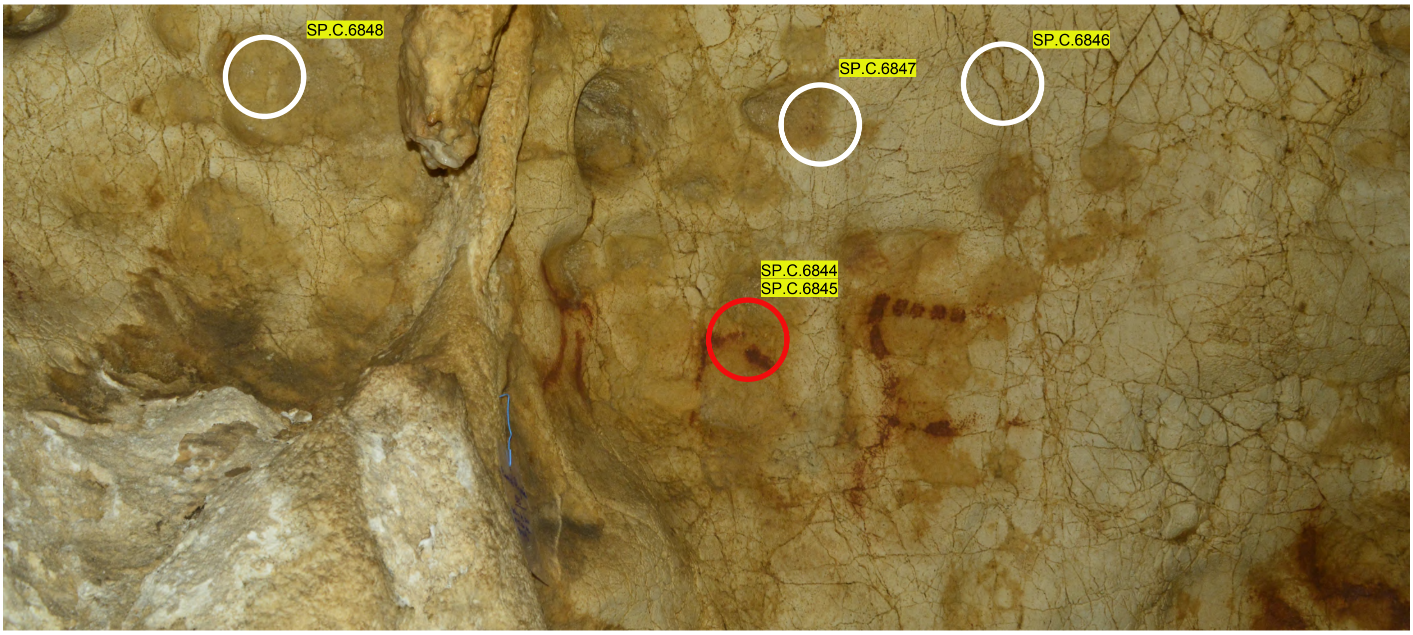

Fig. S5.1. Plan indicating area sampled for DNA analyses in Cueva del Higuerón (Sector B in the subterranean complex of El Cantal) (upper); list of samples screened (upper right); cave wall with annotation of sampled locations for pigment and control sampling (lower).

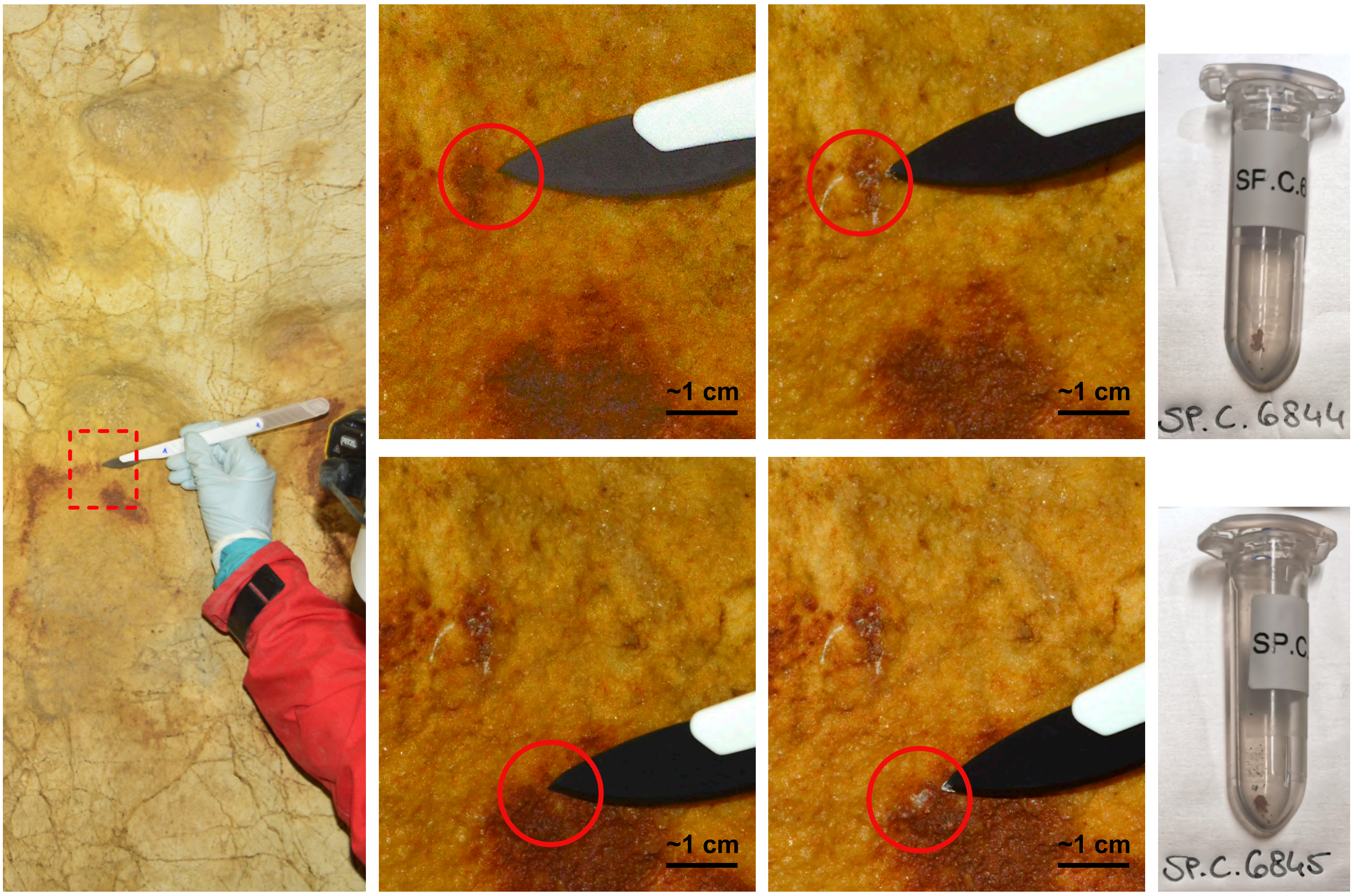

Fig. S5.2. Pigment samples SP.C.6844 & SP.C.6845: general location with sampled area highlighted in red (left); cave wall before sampling SP.C.6844 - after sampling - sample in tube (upper right); cave wall before sampling SP.C.6845 - after sampling - sample in tube (lower right).

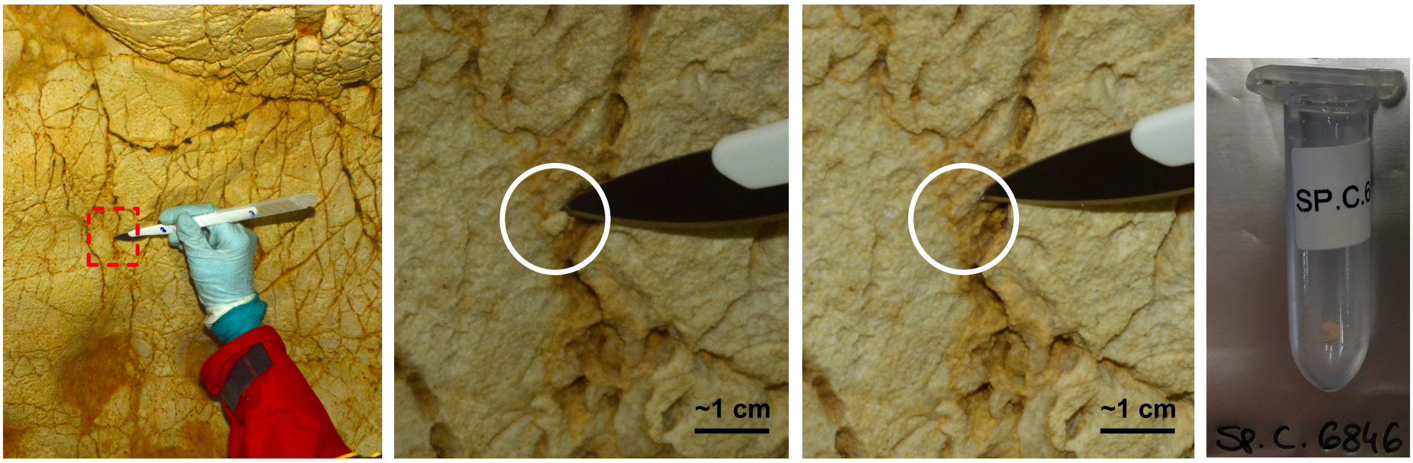

Fig. S5.3. Control sample SP.C.6846: general location with sampled area highlighted in red (left); cave wall before sampling - after sampling - sample in tube (right).

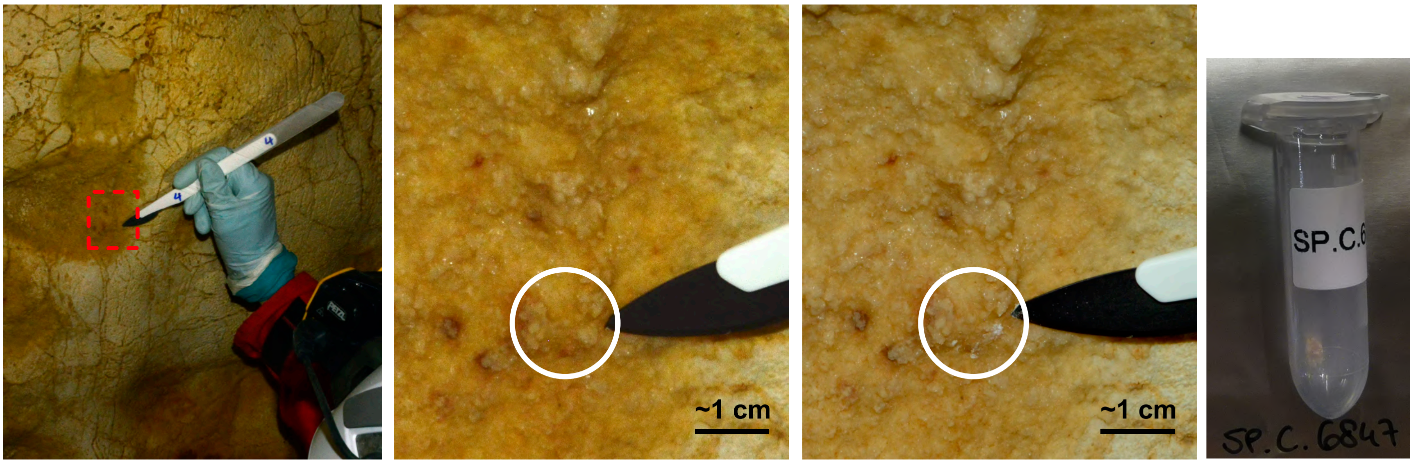

Fig. S5.4. Control sample SP.C.6847: general location with sampled area highlighted in red (left); cave wall before sampling - after sampling - sample in tube (right).

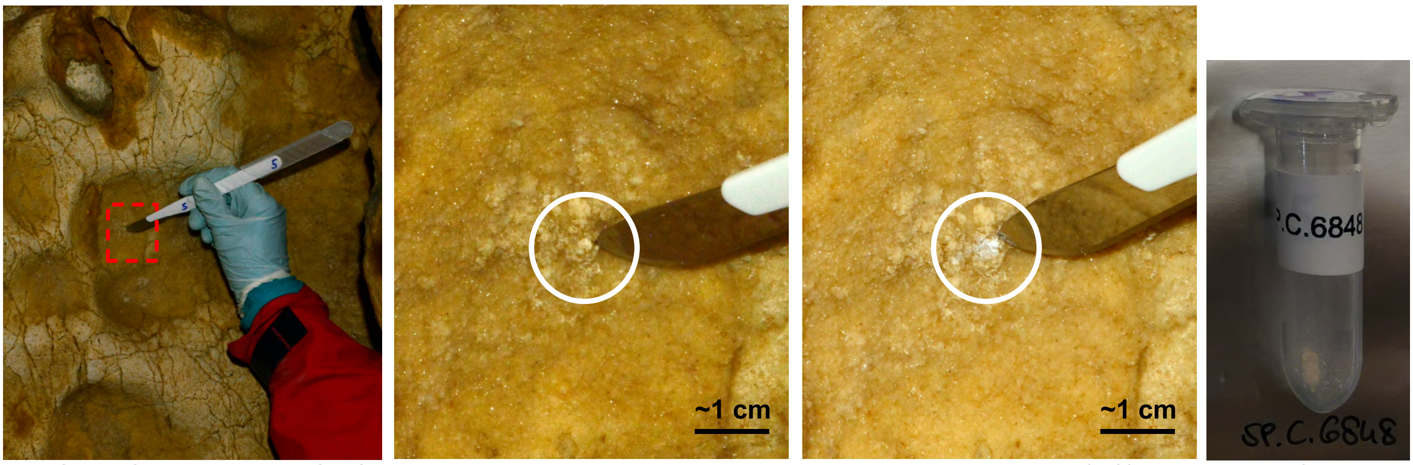

Fig. S5.5. Control sample SP.C.6848: general location with sampled area highlighted in red (left); cave wall before sampling - after sampling - sample in tube (right).

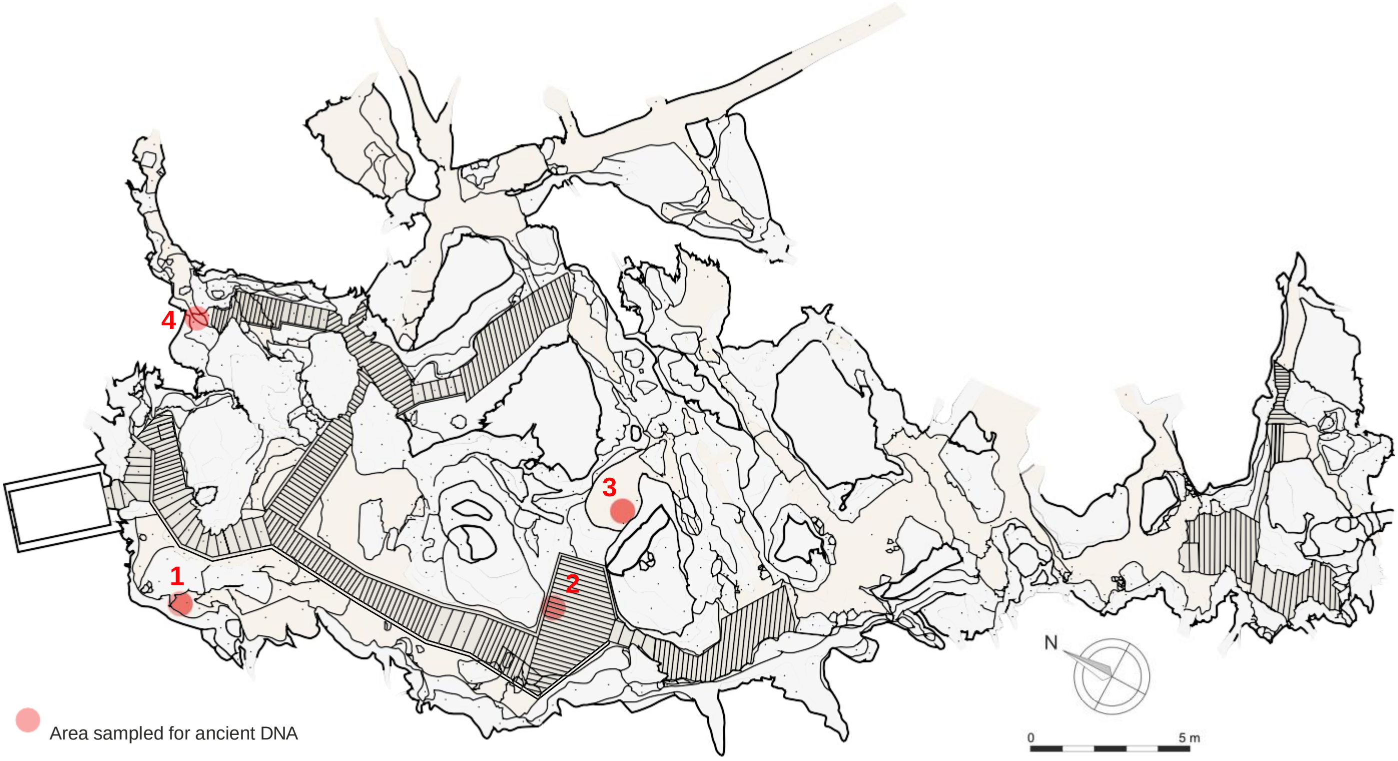

Sample ID	Area	Sample Type
SP.B.2674	Panel 11	Pigment & Control (Wall)
SP.B.2675	Panel 11	Pigment (Wall)
SP.C.6776	Panel 11	Pigment & Control (Wall)
SP.C.6778	Panel 11	Pigment & Control (Wall)
SP.C.6780	Panel 11	Pigment & Control (Wall)
SP.C.6783	Panel 11	Pigment & Control (Wall)
SP.C.6785	Panel 38	Pigment (Wall)
SP.C.6788	Panel 26	Pigment (Wall)
SP.C.6790	Panel 26	Pigment & Control (Wall)
SP.C.6792	Panel 64	Pigment (Wall)
SP.C.6793	Panel 64 (high)	Control (Wall)
SP.C.6795	Panel 64 (high)	Control (Wall)
SP.C.6797	Panel 64 (high)	Control (Wall)
SP.C.6799	Panel 64 (high)	Control (Wall)
SP.C.6801	Panel 64 (high)	Control (Wall)
SP.C.6803	Panel 64 (high)	Control (Wall)
SP.C.6804	Panel 64 (low)	Control (Wall)
SP.C.6806	Panel 64 (low)	Control (Wall)
SP.C.6808	Panel 64 (low)	Control (Wall)
SP.C.6810	Panel 64 (low)	Control (Wall)
SP.C.6812	Panel 64 (low)	Control (Wall)
SP.C.6813	Panel 64 (low)	Control (Wall)
SP.C.6814	Panel 26	Control (Wall)
SP.C.6816	Panel 26	Control (Wall)
SP.C.6817	Panel 64	Control (Wall)
SP.C.6818	Panel 64	Control (Wall)
SP.C.6820	Panel 38	Control (Wall)
SP.C.6821	Panel 38	Control (Wall)
SP.C.6822	Panel 38	Control (Wall)
SP.C.6826	Panel 11	Control (Wall)
SP.C.6827	Panel 11	Control (Wall)

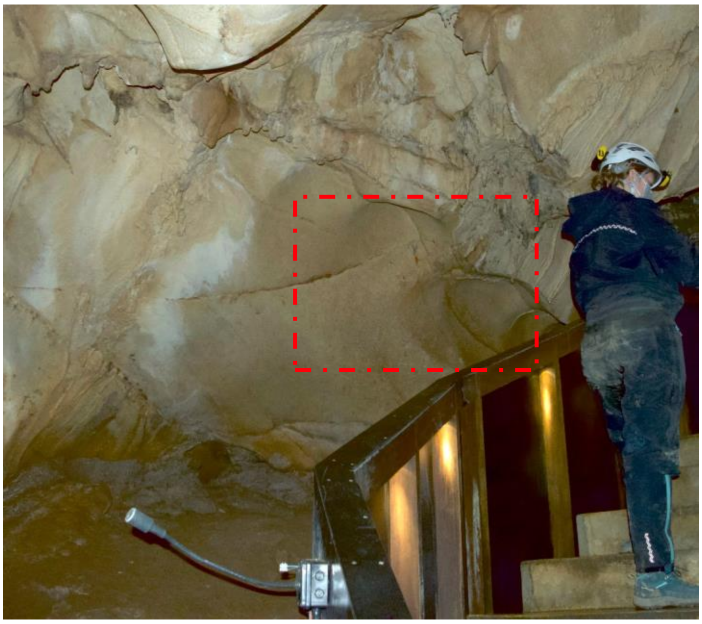

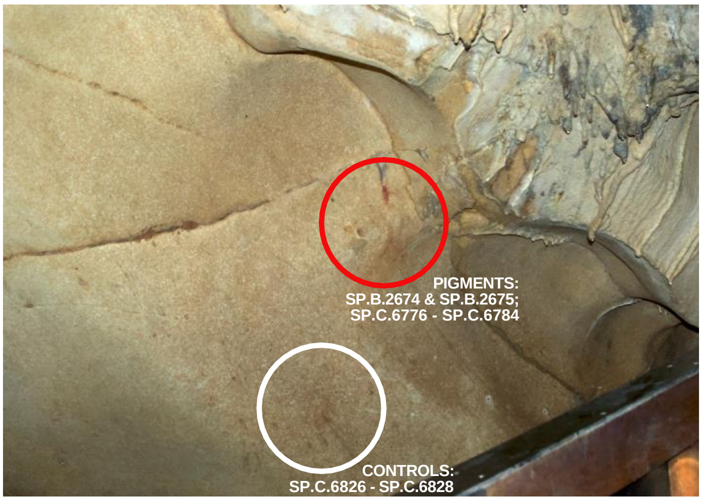

Fig. S6.1. Plan indicating areas sampled for DNA analyses at Gruta do Escoural: Panel 11 (1); Panel 26 (2); Panel 64 (3); Panel 38 (4) (upper). List of samples screened for ancient DNA (lower left); Panel 11 beyond staircase with enlarged view indicating areas of pigment vs. control samples (lower right).

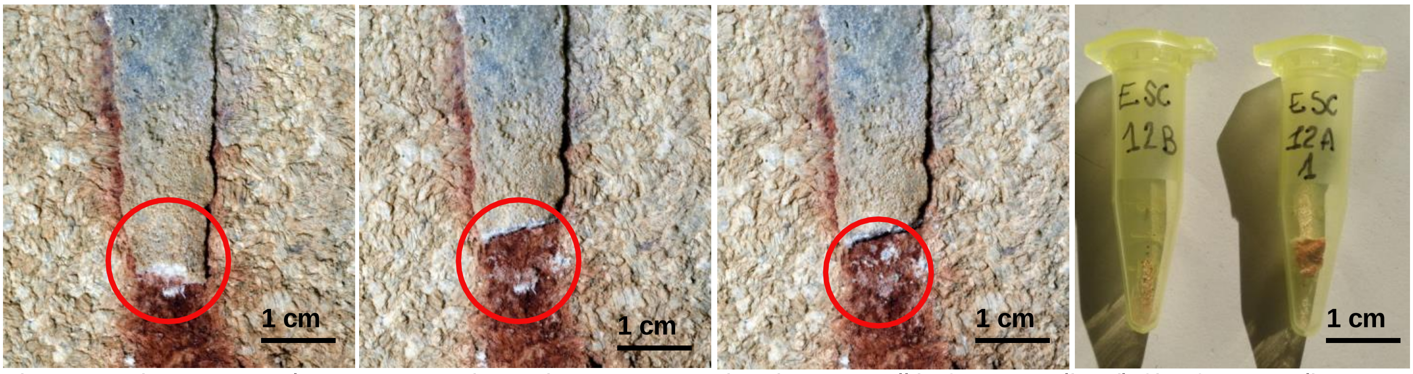

Fig. S6.2. Pigment samples SP.B.2674 (12A-1) & SP.B.2675 (12B): cave wall before sampling (left); after sampling 12A-1 (middle left); after sampling 12B (middle right); samples in tubes (right).

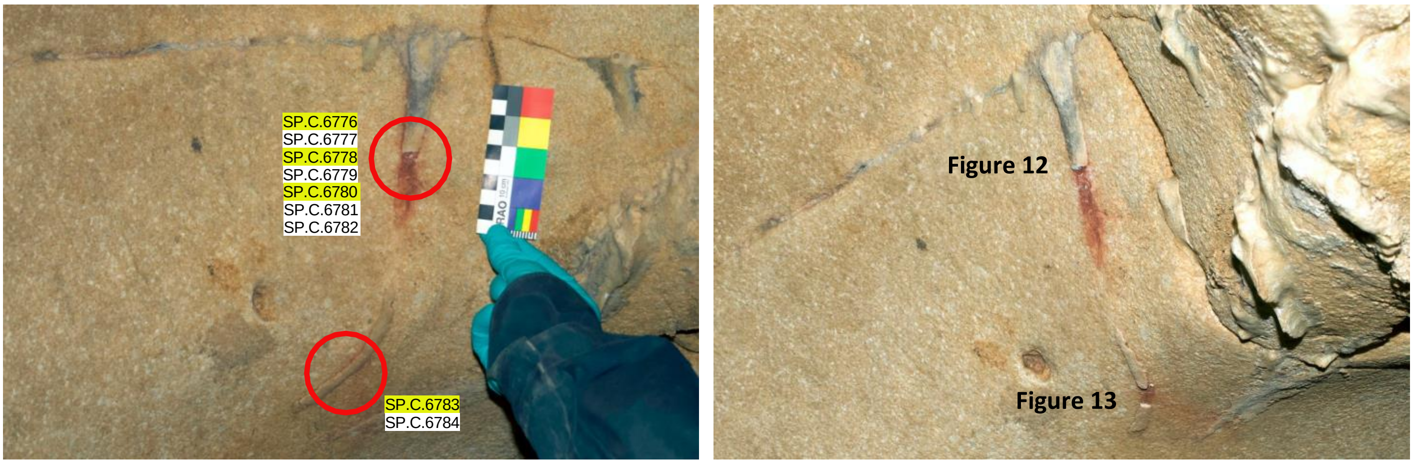

Fig. S6.3. Pigment sampling of SP.C.6776 - SP.C.6783: overview of sampled area with annotated locations of samples (left); after completed sampling (right).

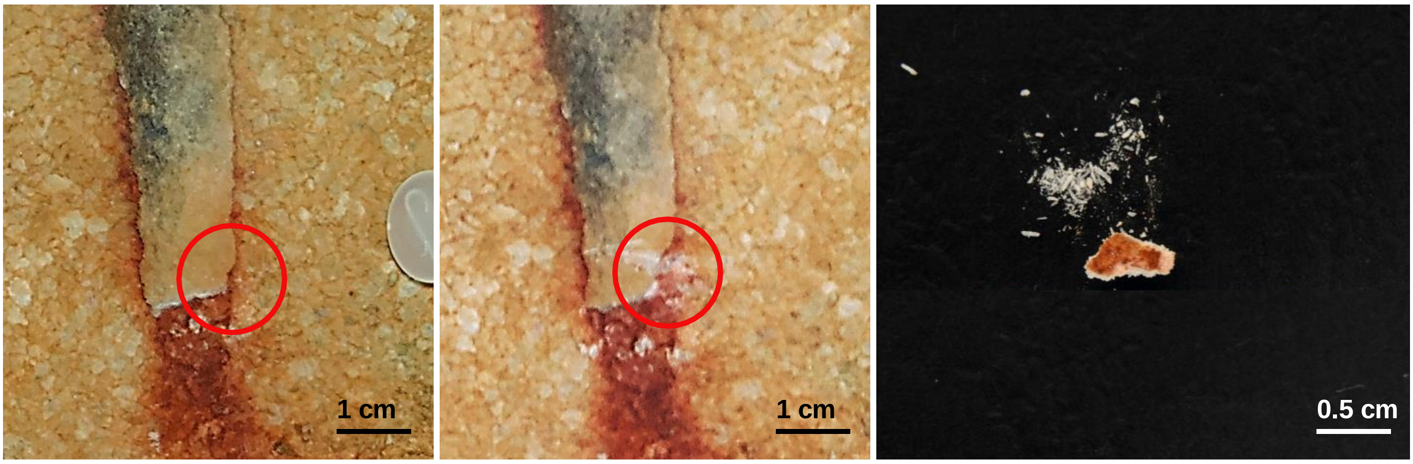

Fig. S6.4. Pigment sample SP.C.6776: cave wall before sampling (left); after sampling (middle); sample (right).

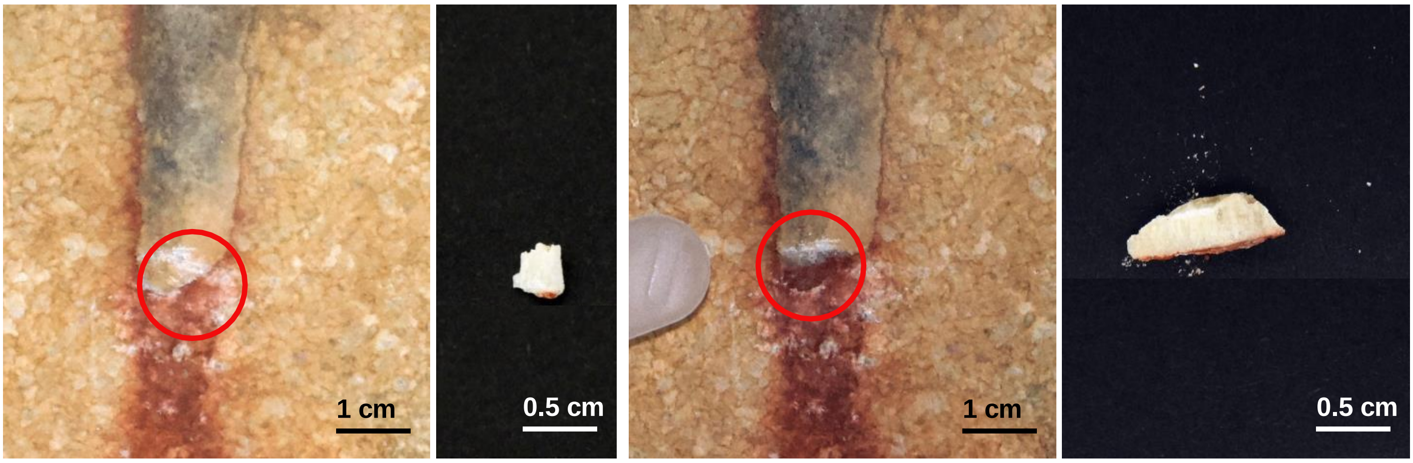

Fig. S6.5. Cave wall after sampling SP.C.6778 & resulting sample (left); after sampling SP.C.6780 & sample (right).

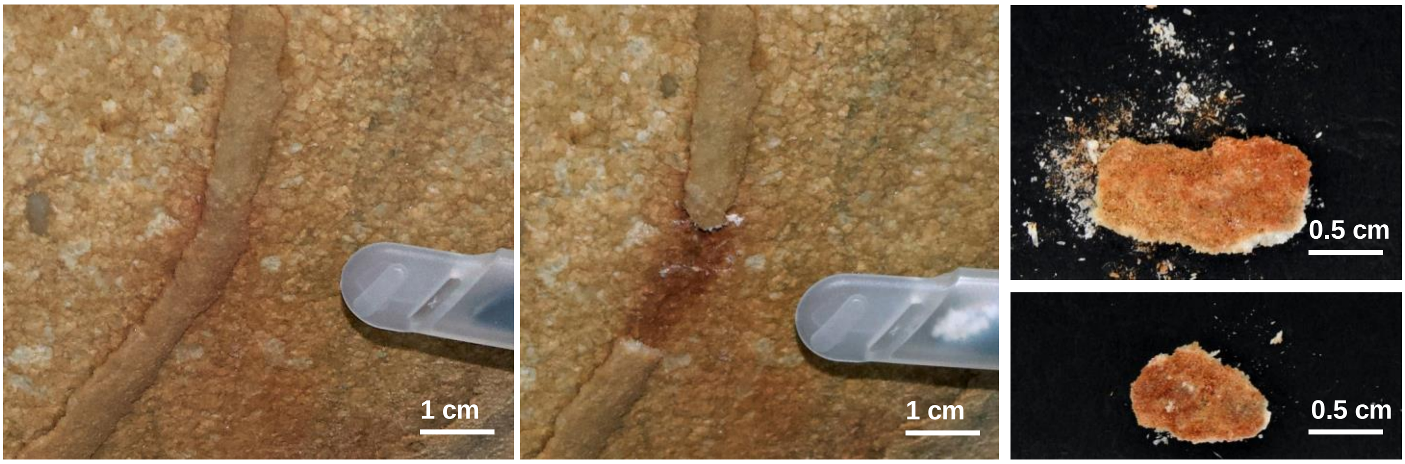

Fig. S6.6. Panel 11, figure 13. Pigment sample SP.C.6783 (subsamples Sub.C.7543 - Sub.C.7549): cave wall before sampling (left); after sampling (middle); subsample 1/Sub.C.7543 (lower right); subsample 3/ Sub.C.7545 (upper right).

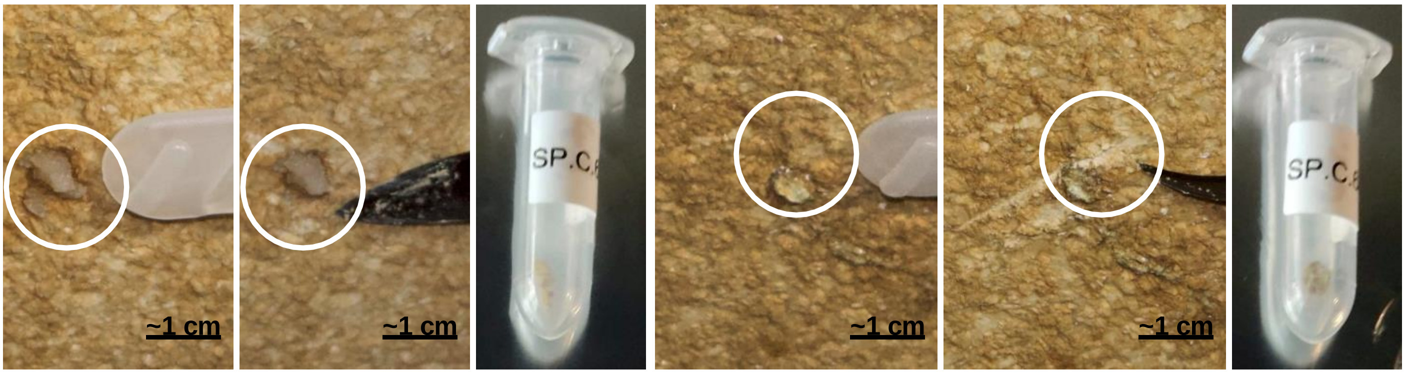

Fig. S6.7. Control sampling of area below Panel 11: control sampling of SP.C.6826: before sampling - after sampling - sample in tube (left); control sampling of SP.C.6827: before sampling - after sampling - sample in tube (right).

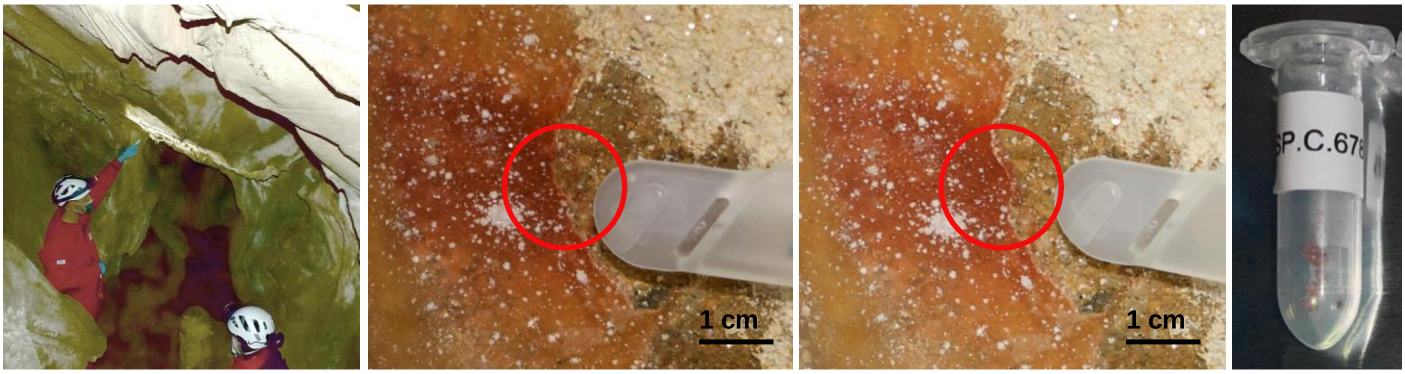

Fig. S6.8. Pigment sampling of SP.C.6785 at the Panel 38: overview of sampled area (left); before sampling - after sampling - sample in tube (right).

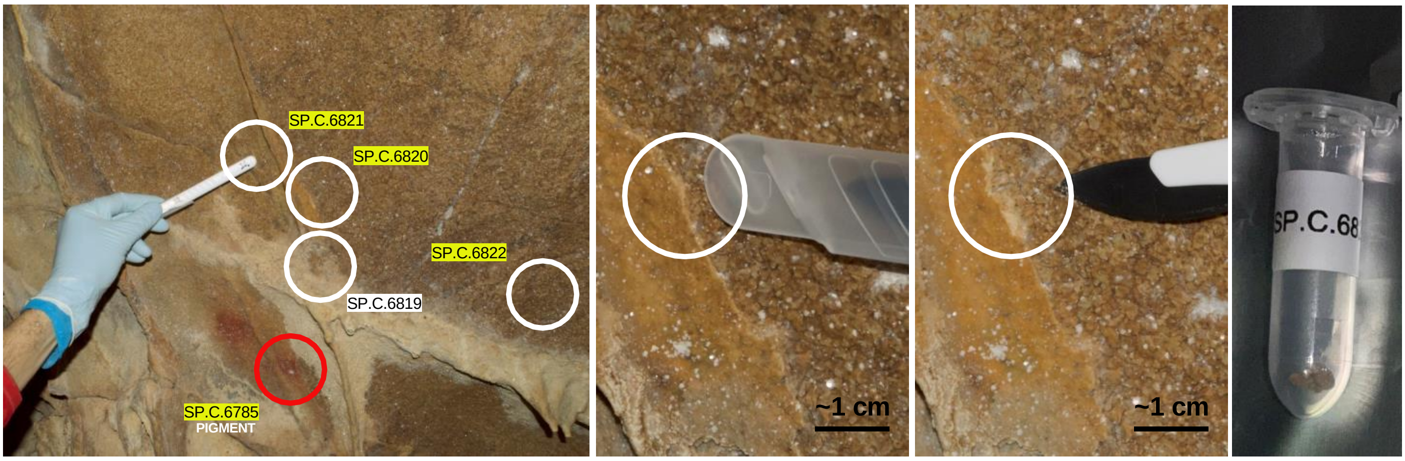

Fig. S6.9. Control sampling of area above Panel 38: overview of sampled area showing annotated locations of samples with pigment sample as reference (left); control sampling of SP.C.6820: before sampling - after sampling - sample in tube (right).

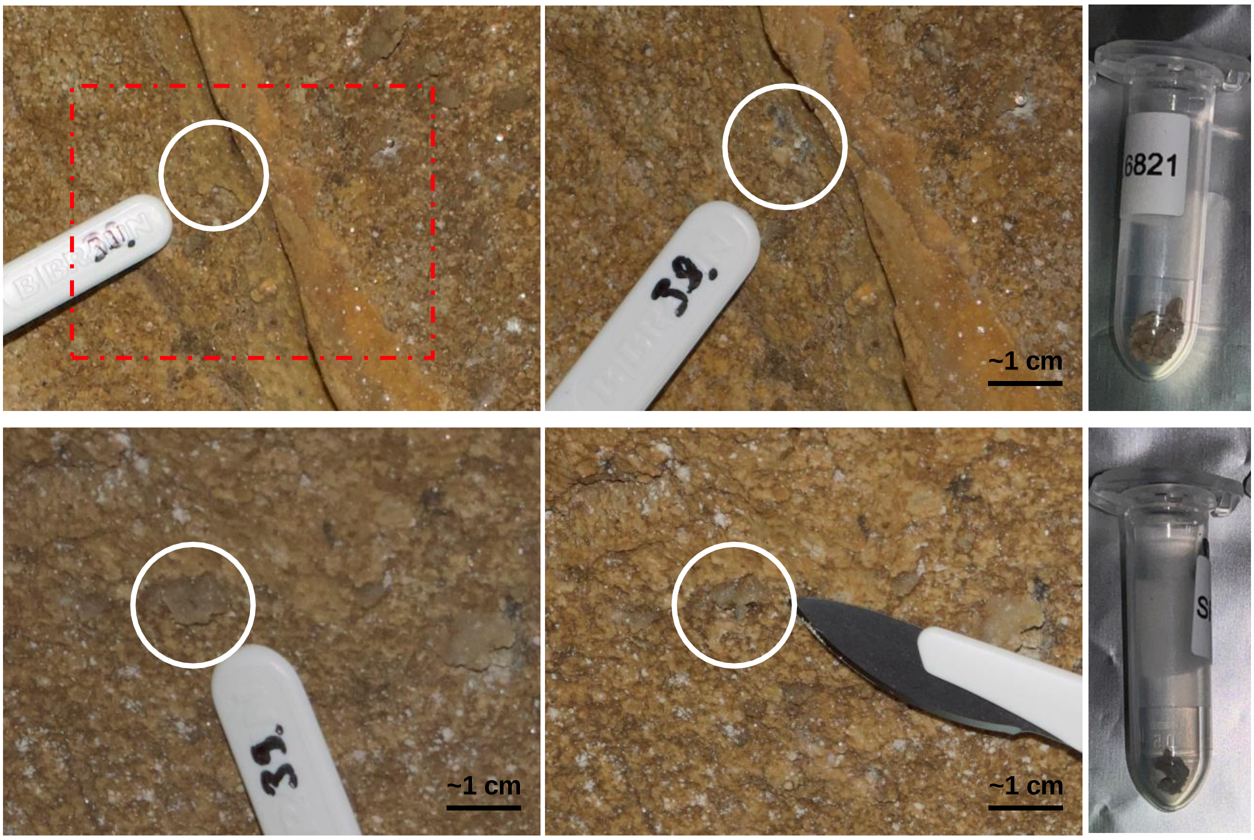

Fig. S6.10. Control sampling of wall above the Panel 38: control sample SP.C.6821: before sampling - after sampling - sample in tube (upper); control sample SP.C.6822: before sampling - after sampling - sample in tube (lower).

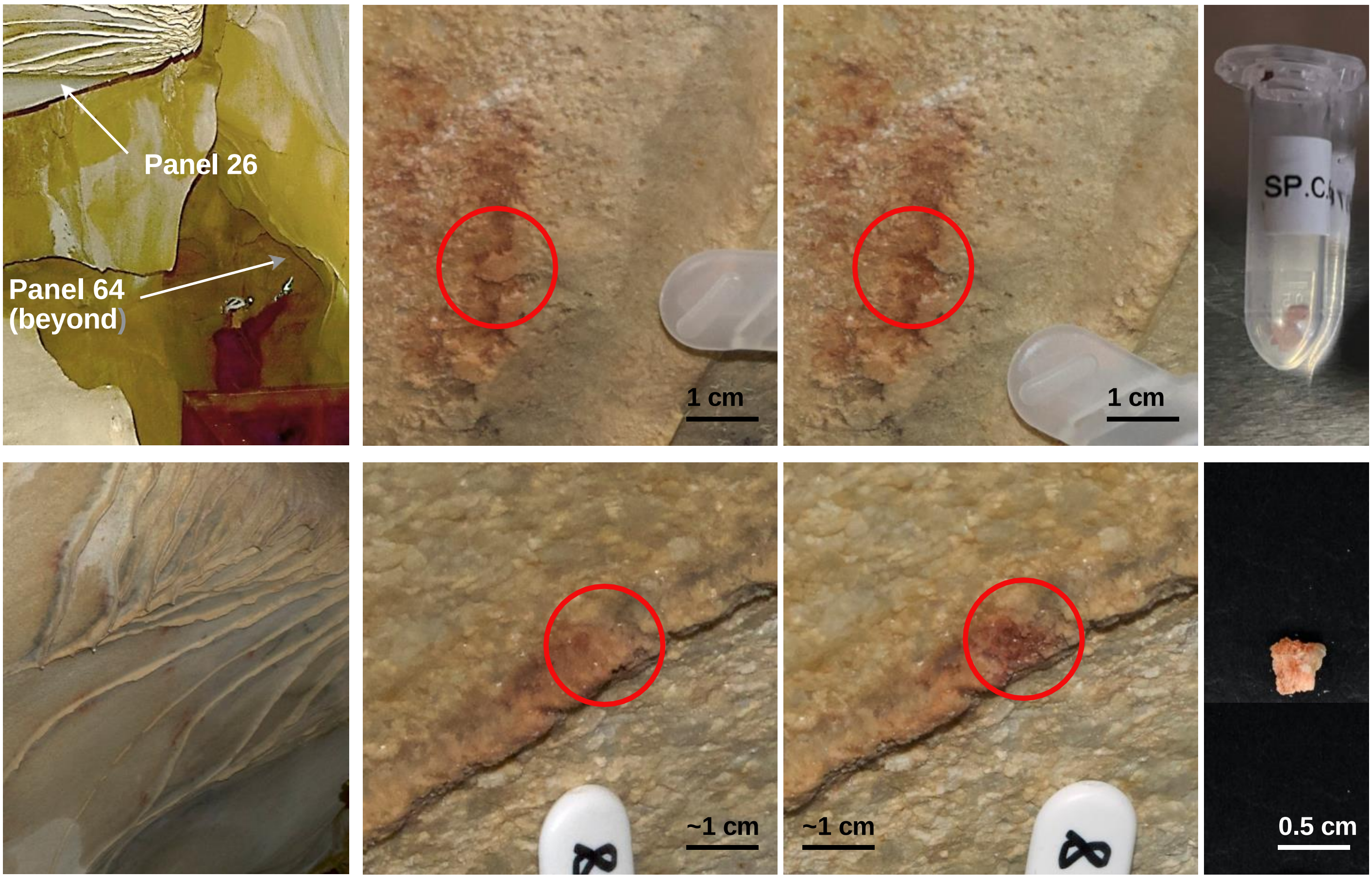

Fig. S6.11. Pigment sampling of SP.C.6788 & SP.C.6790 at the Panel 26: overview of sampled area (upper left); pigment sample SP.C.6788: before sampling - after sampling - sample in tube (upper right); enlarged view of area sampled in Panel 26 (lower left); pigment sample SP.C.6790: before sampling - after sampling - sample (lower right).

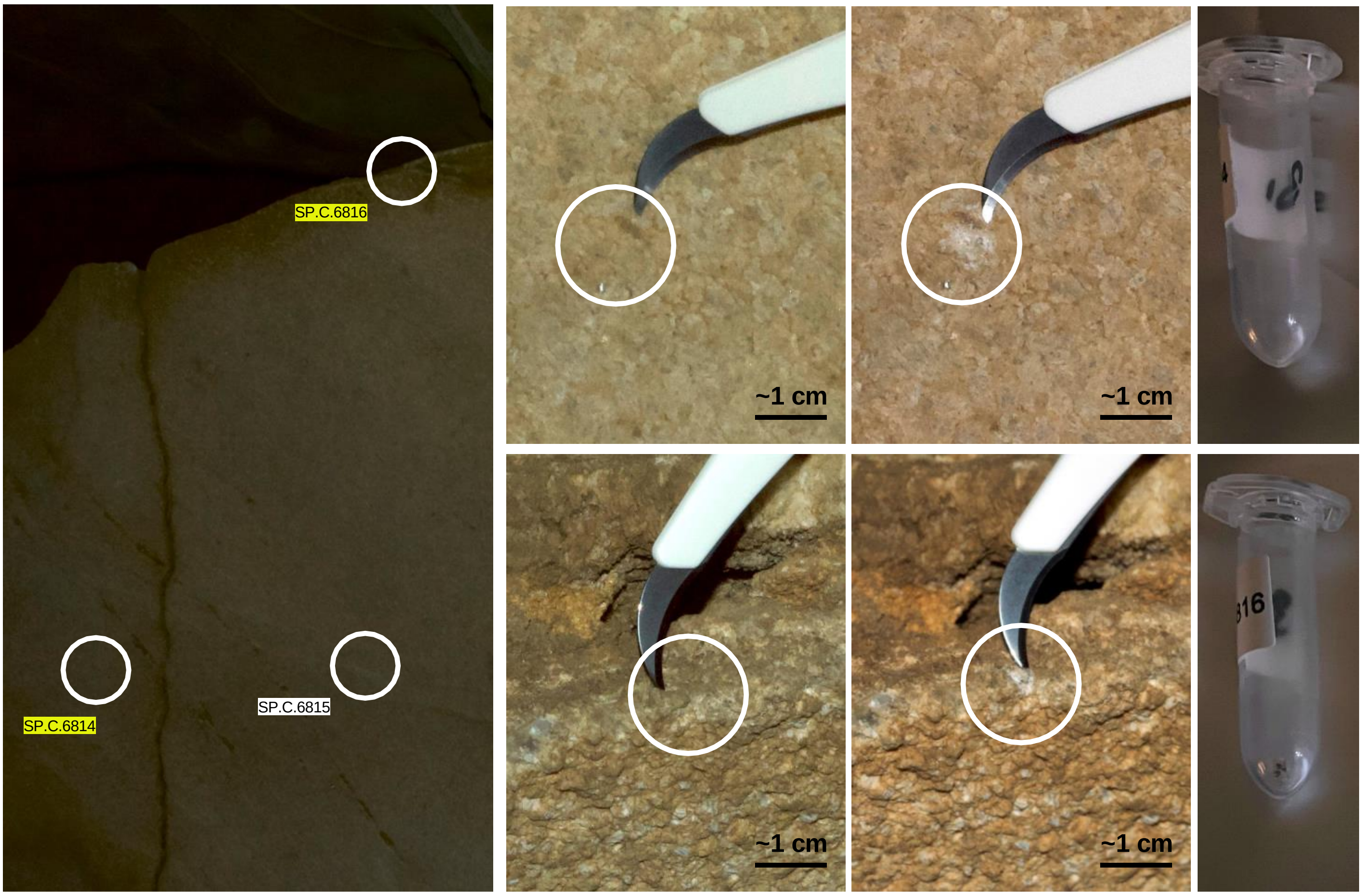

Fig. S6.12. Overview of the area sampled for controls at the wall below the Panel 26, showing annotated locations of samples (left); control sample SP.C.6814: before sampling - after sampling - sample in tube (upper right); control sample SP.C.6816: before sampling - after sampling - sample in tube (lower right).

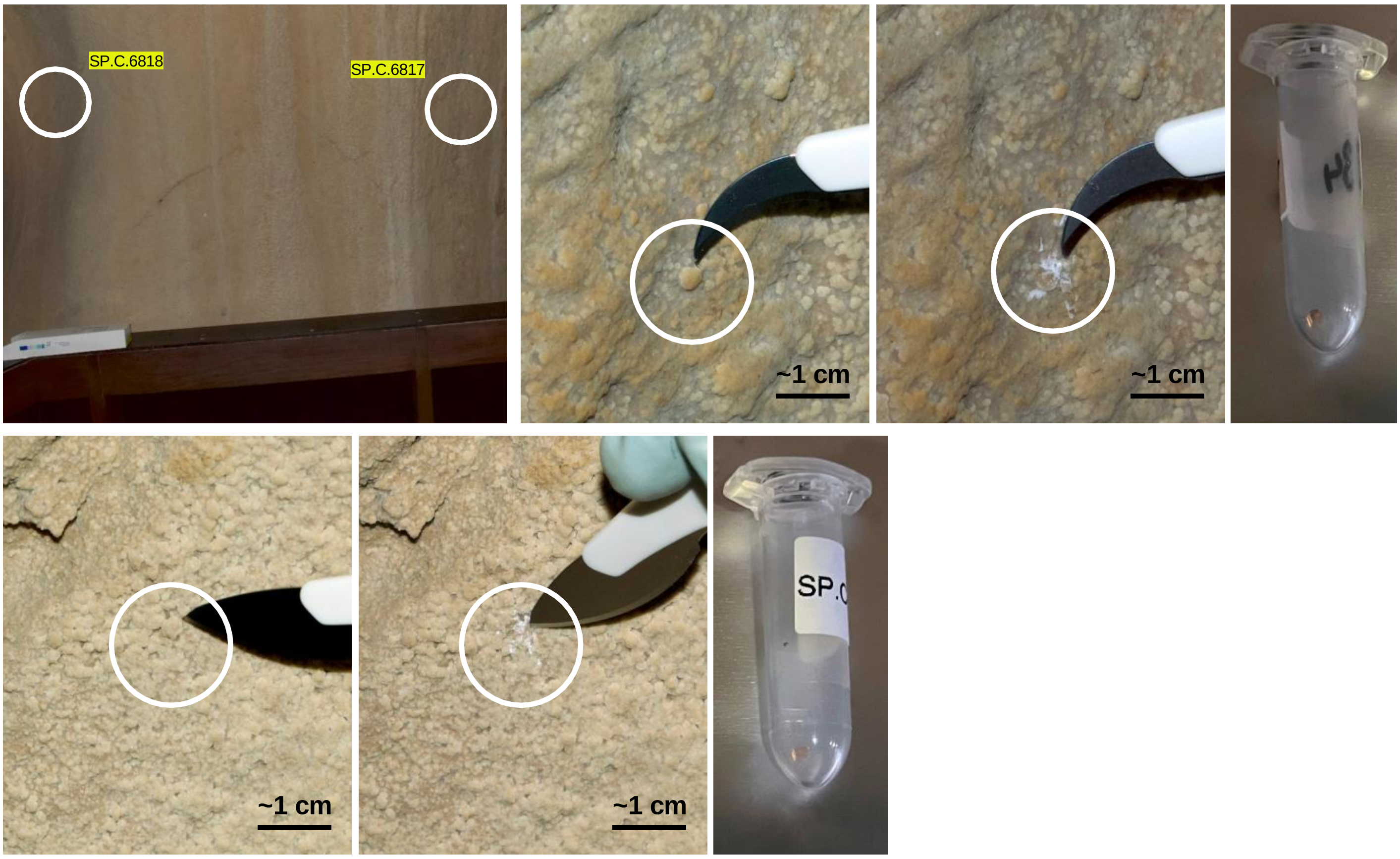

Fig. S6.13. Overview of area sampled for controls at the visitor wall, opposite the panel 26, showing annotated locations of samples (upper left). Control sample SP.C.6817: before sampling - after sampling - sample in tube (upper right). Control sample SP.C.6818: before sampling - after sampling - sample in tube (lower).

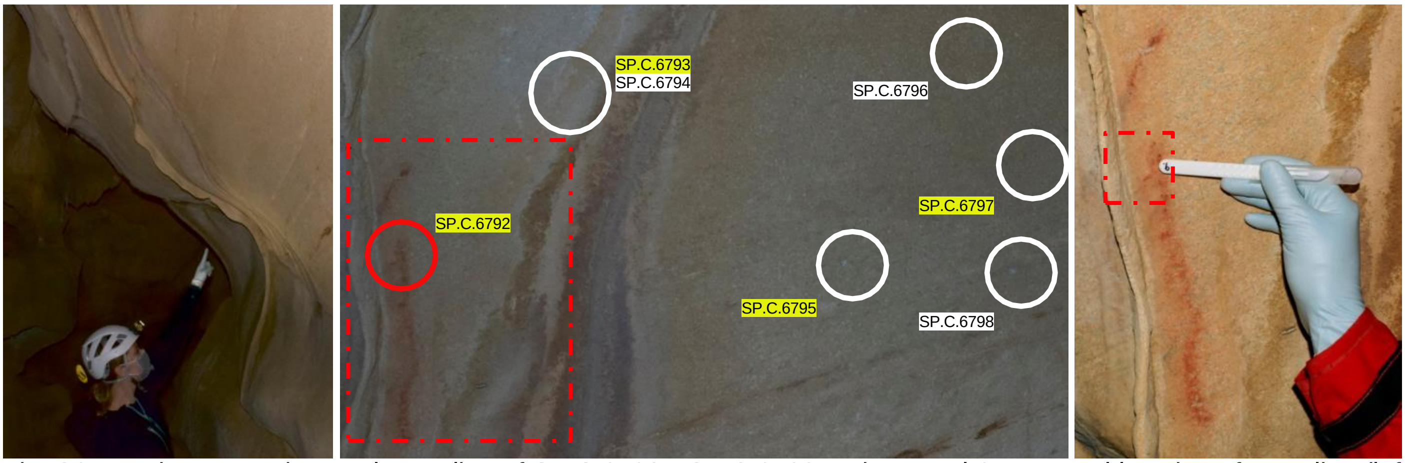

Fig. S6.14. Pigment and control sampling of SP.C.6792 - SP.C.6798 at the Panel 64: general location of sampling (left); overview of sampled area w/ annotated locations of samples (middle); enlarged view of area sampled for pigment (right).

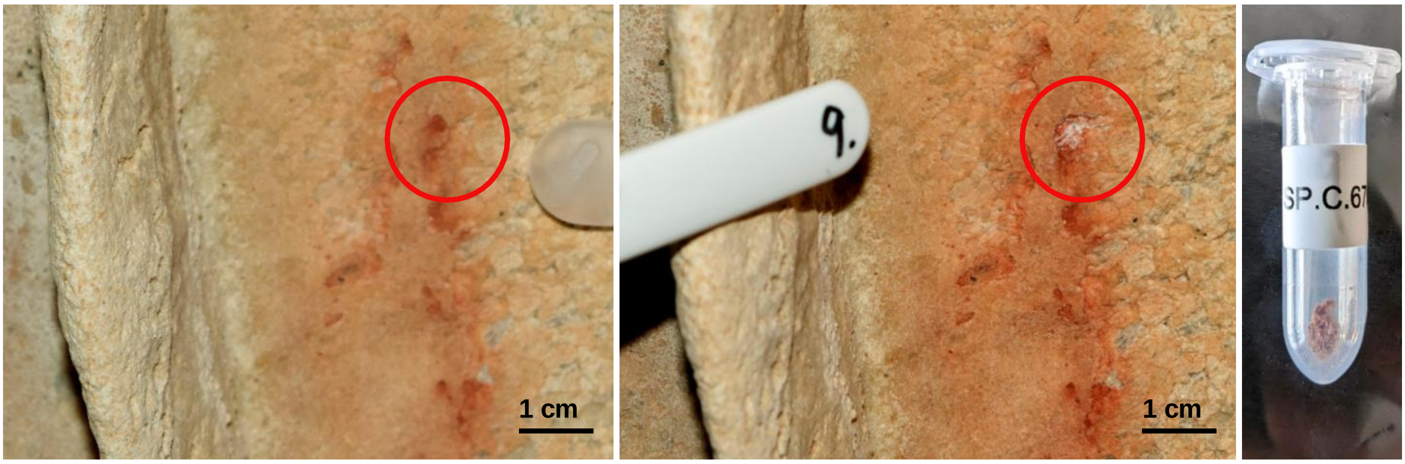

Fig. S6.15. Pigment sample SP.C.6792: before sampling (left); after sampling (middle); sample in tube (right).

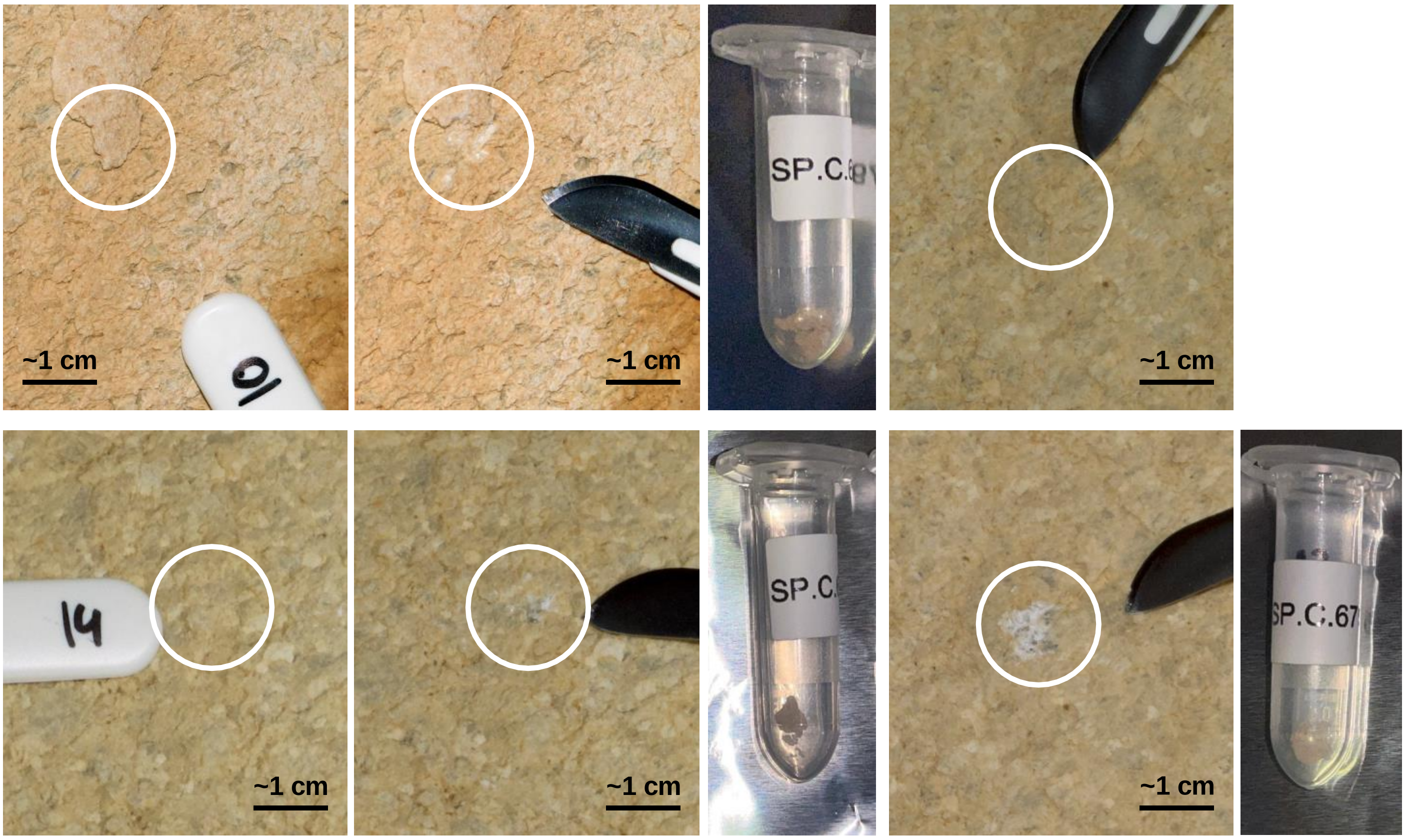

Fig. S6.16. Control sample SP.C.6793: before sampling - after sampling - sample in tube (upper left). Control sample SP.C.6795: before sampling (upper right); after sampling - sample in tube (lower right). Control sample SP.C.6797: before sampling - after sampling - sample in tube (lower left).

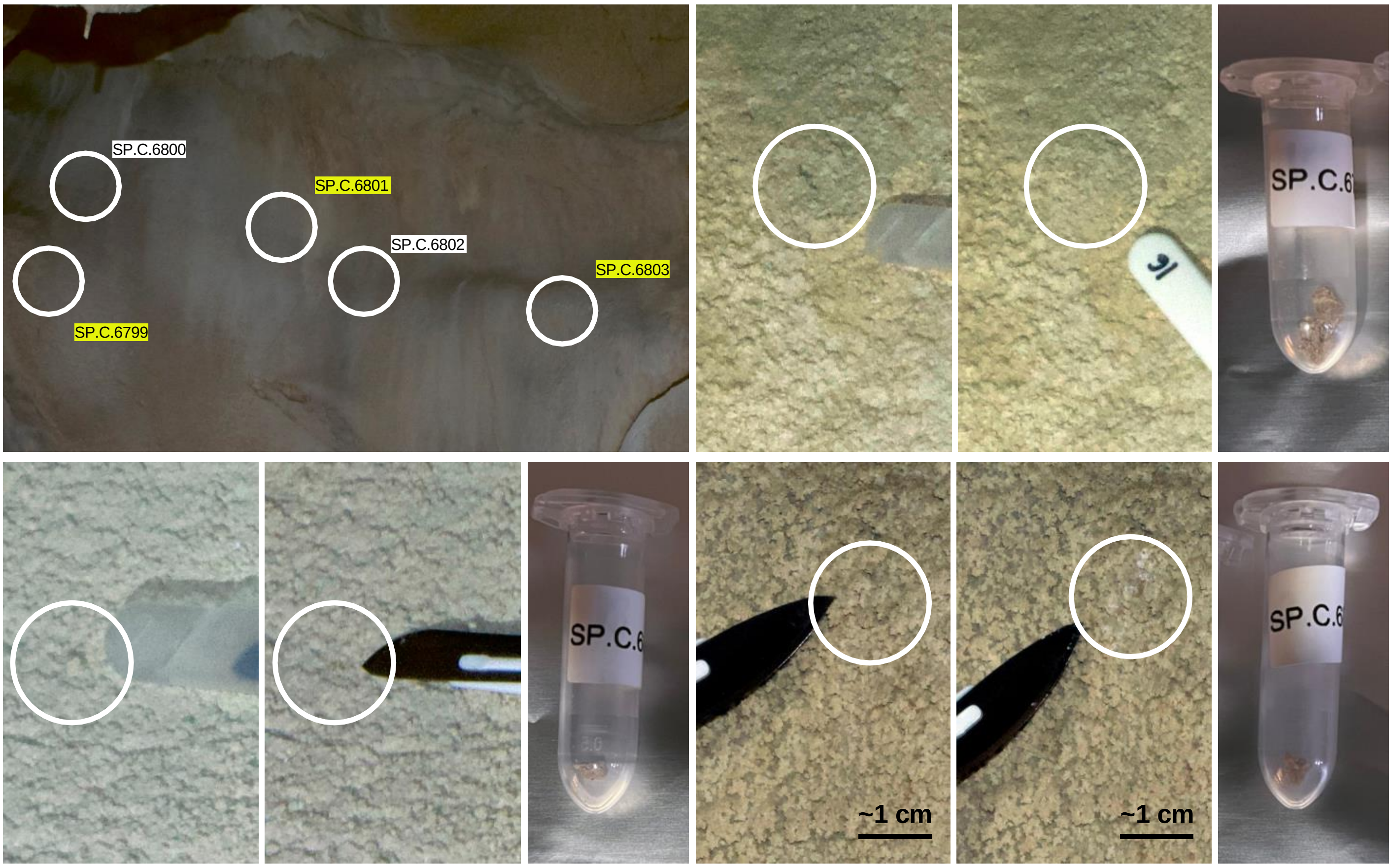

Fig. S6.17. Overview of the are sampled for controls at the wall opposite the Panel 64 (high), showing annotated locations of samples (upper left). Control sample SP.C.6799: before sampling - after sampling - sample in tube (upper right). Control sample SP.C.6801: before sampling - after sampling - sample in tube (lower left). Control sample SP.C.6803: before sampling - after sampling - sample in tube (lower right).

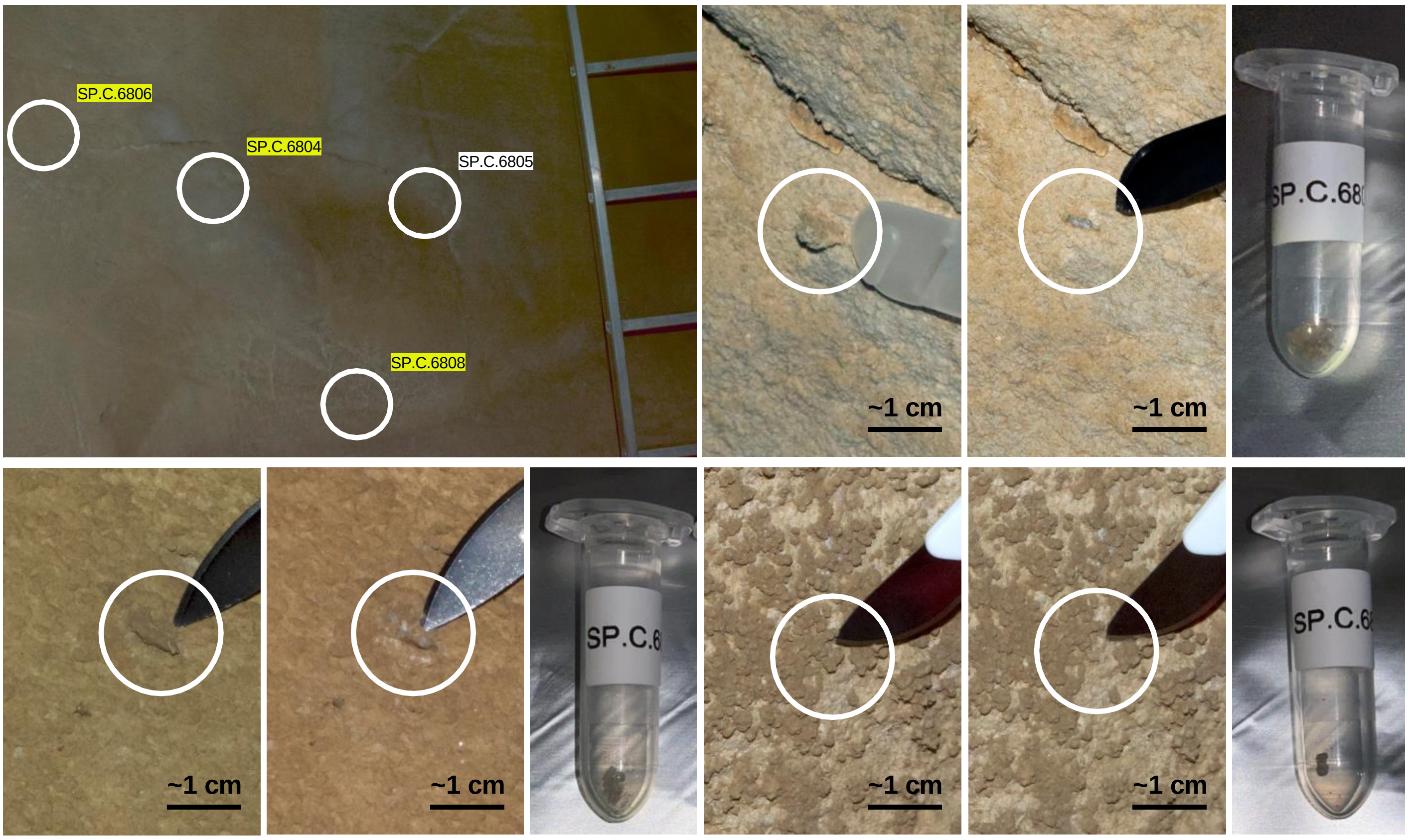

Fig. S6.18. Overview of area sampled for controls at the wall opposite the Panel 64 (low), showing annotated locations of samples (upper left). Control sample SP.C.6804: before sampling - after sampling - sample in tube (upper right). Control sample SP.C.6806: before sampling (middle); after sampling - sample in tube (lower left). Control sample SP.C.6808: before sampling - after sampling - sample in tube (lower right).

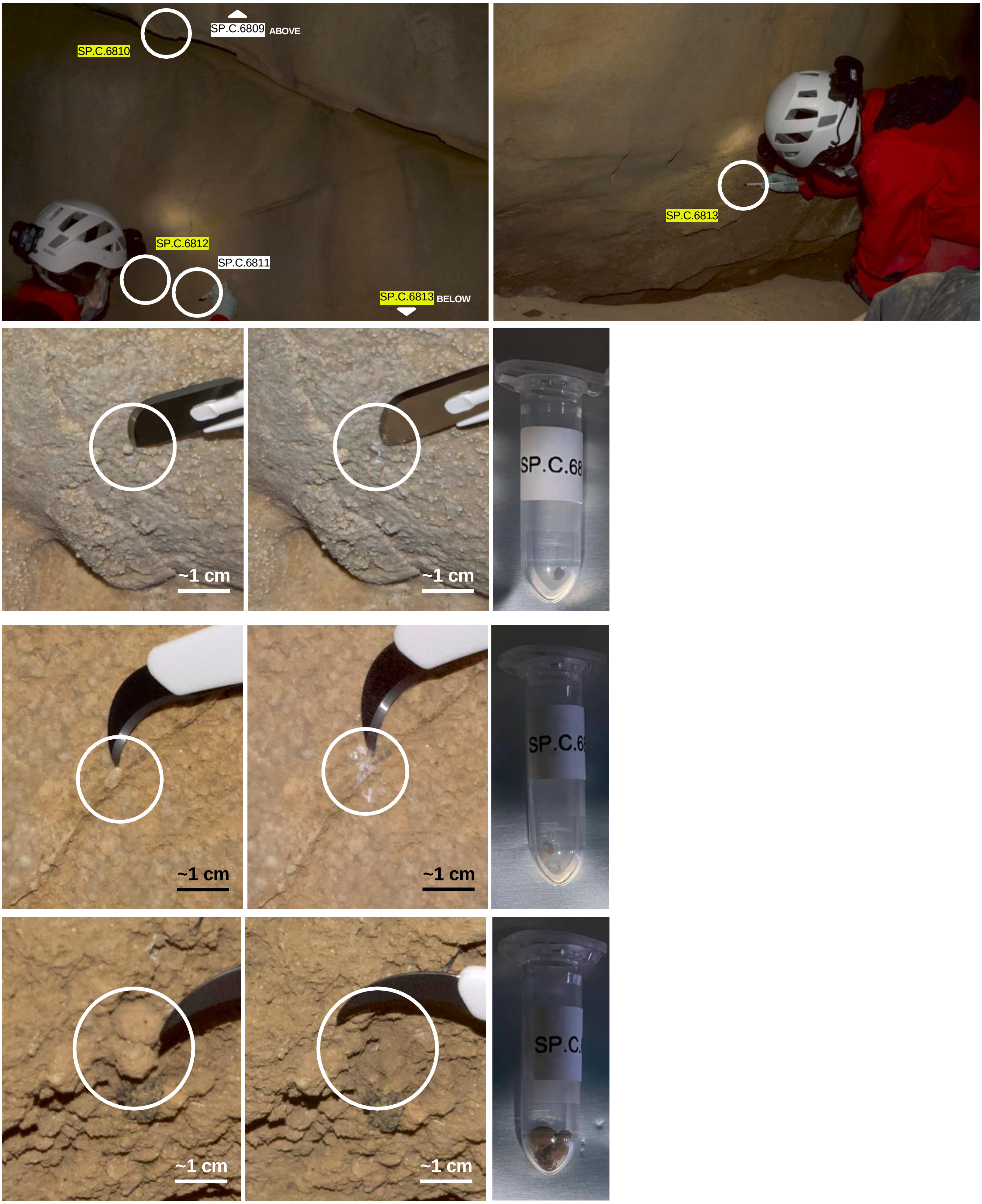

Fig. S6.19. Overview of area sampled for controls at the wall below the Panel 64 (low), showing annotated locations of samples (upper). Control sample SP.C.6810: before sampling - after sampling - sample in tube (upper middle). Control sample SP.C.6812: before sampling - after sampling - sample in tube (lower middle). Control sample SP.C.6813: before sampling - after sampling - sample in tube (lower).

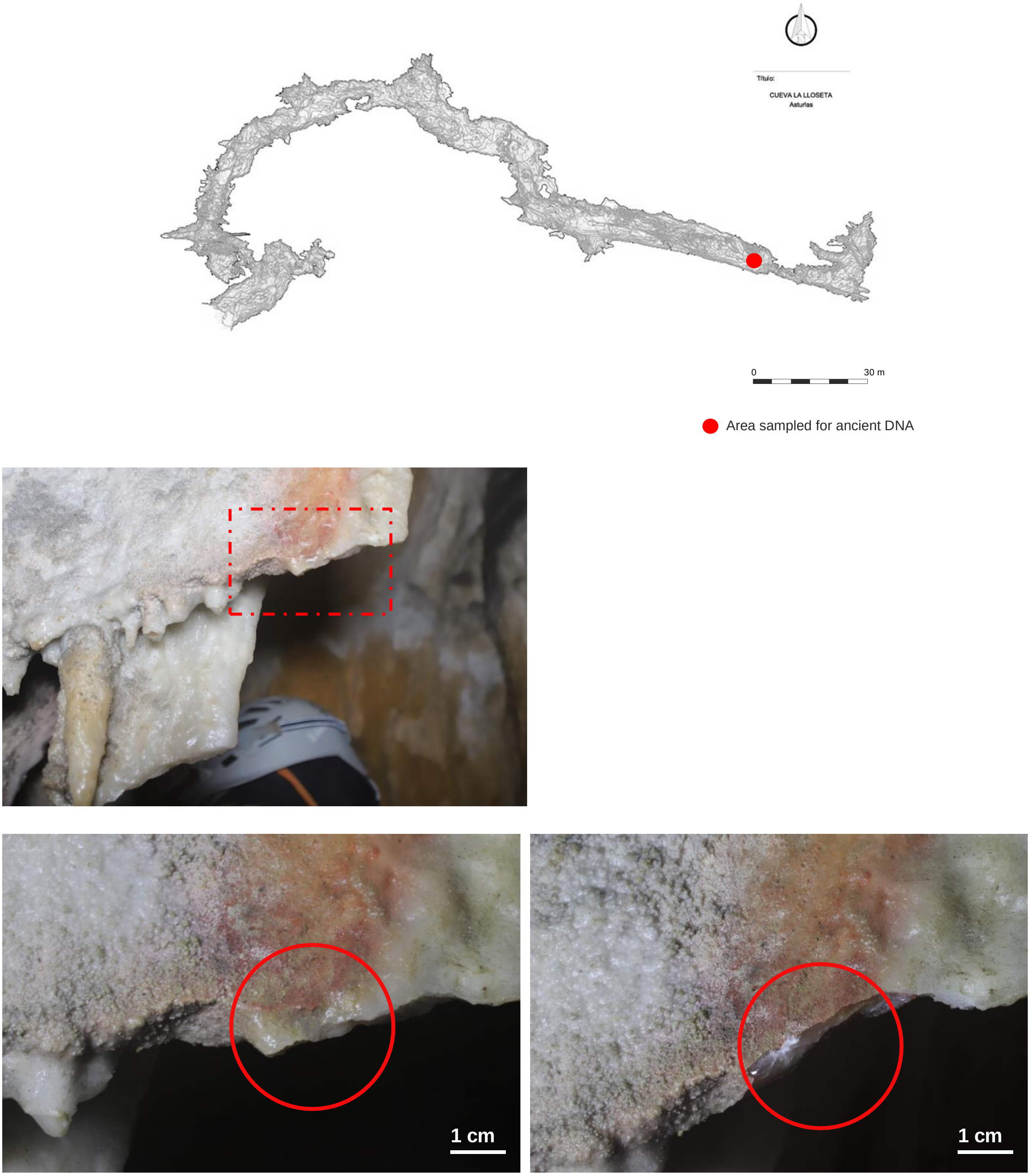

Fig. S7.1. Plan indicating area sampled for DNA analyses at Conjunto 9 of Cueva de la Lloseta (upper); overview of sampled area with enlarged sample location for SP.C.7182 highlighted in red (middle); before sampling (lower left); after sampling (lower right).

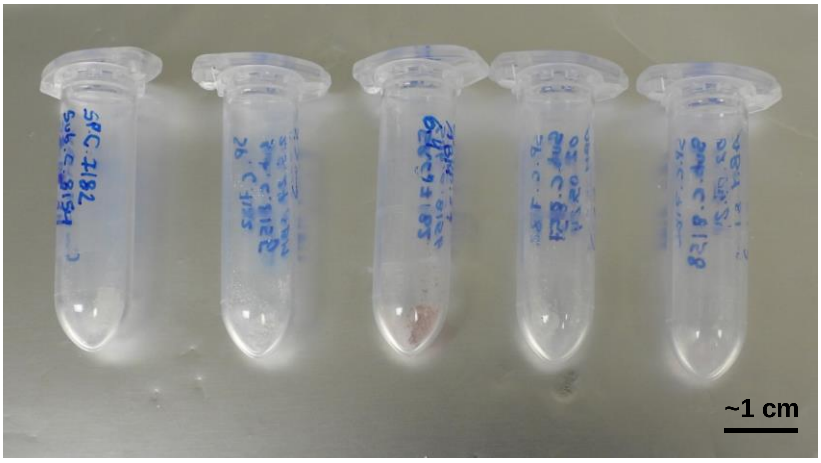

Sample ID	Subsample ID	Type
SP.C.7182	Sub.C.8155	Control
SP.C.7182	Sub.C.8156	Pigment
SP.C.7182	Sub.C.8157	Control
SP.C.7182	Sub.C.8158	Pigment

Fig. S7.2. Subsamples Sub.C.8154 - Sub.C.8158 in tubes (left); table indicating type of material (right).

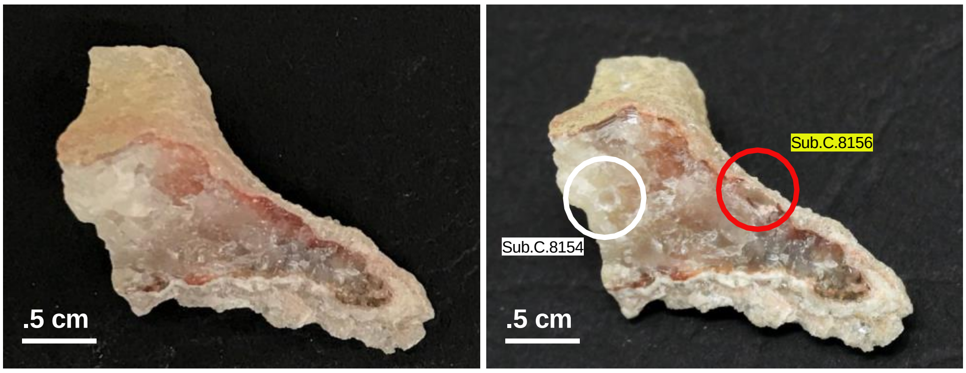

Fig. S7.3. Pigment and control subsamples Sub.C.8154 & Sub.C.8156: sample before sampling (left); after sampling (right).

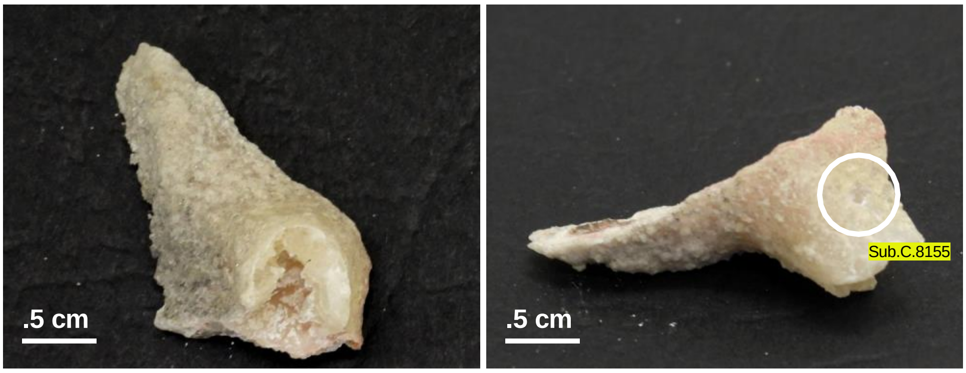

Fig. S7.4. Control subsample Sub.C.8155: sample before sampling (left); after sampling (right).

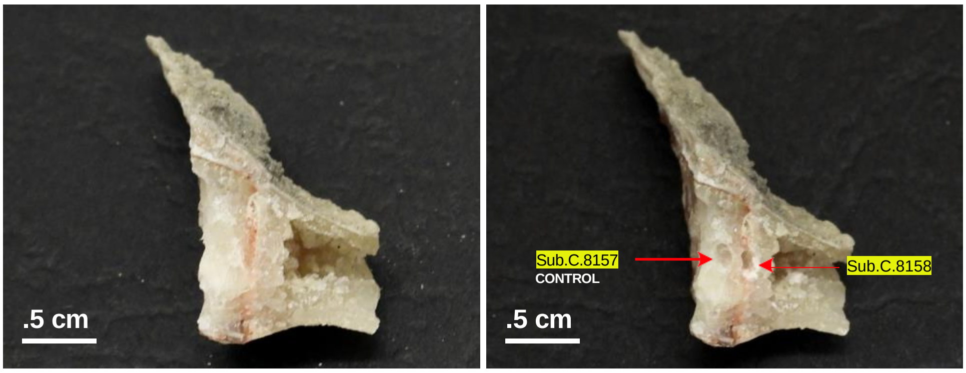

Fig. S7.5. Pigment and control subsamples Sub.C.8157 & Sub.C.8158: sample before sampling (left); after sampling (right).

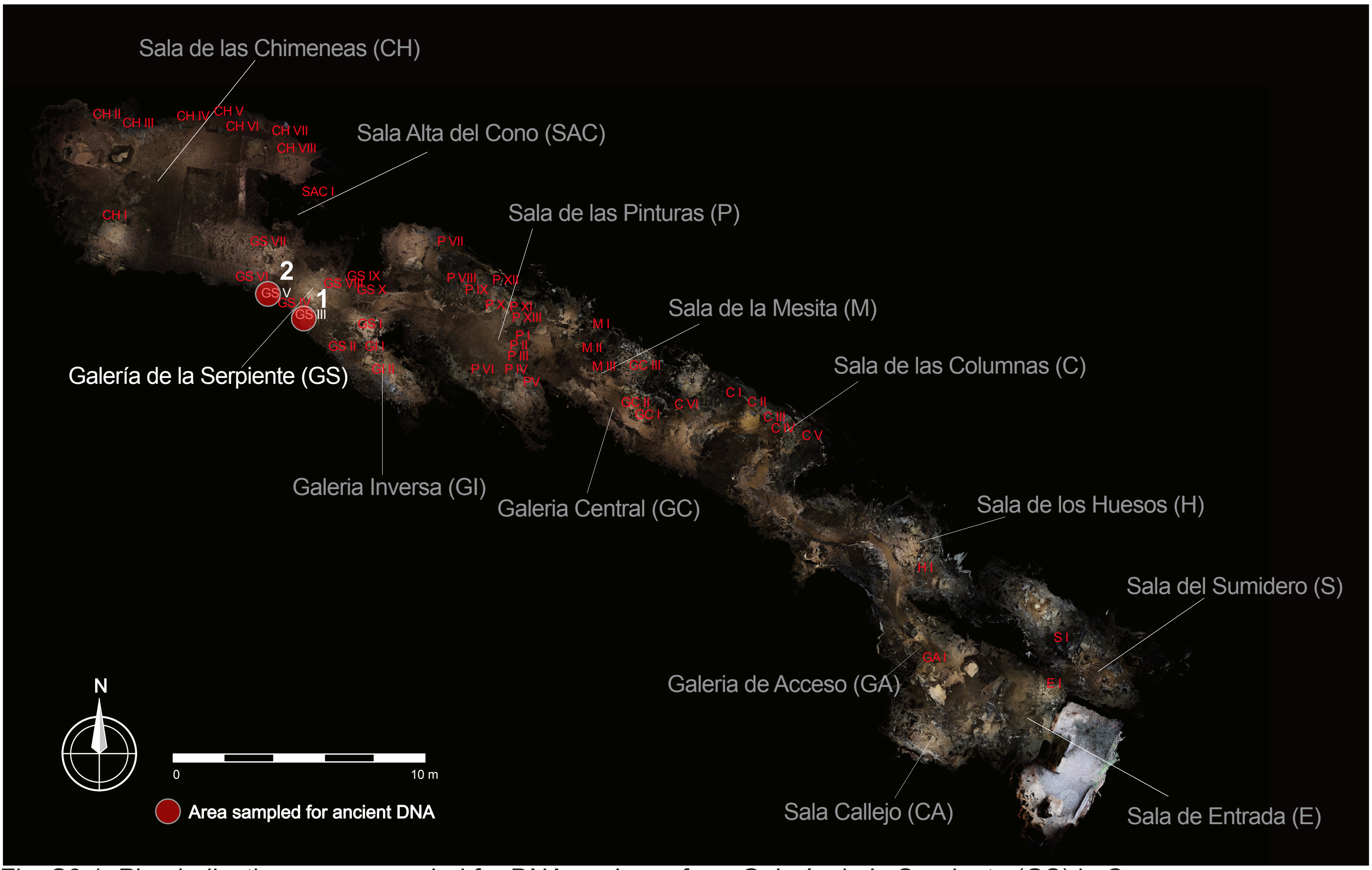

Fig. S8.1. Plan indicating areas sampled for DNA analyses from Galería de la Serpiente (GS) in Cueva Maltravieso: Panel GS III-3, Mano 53 (1); Panel GS V-4, Mano 59 (2).

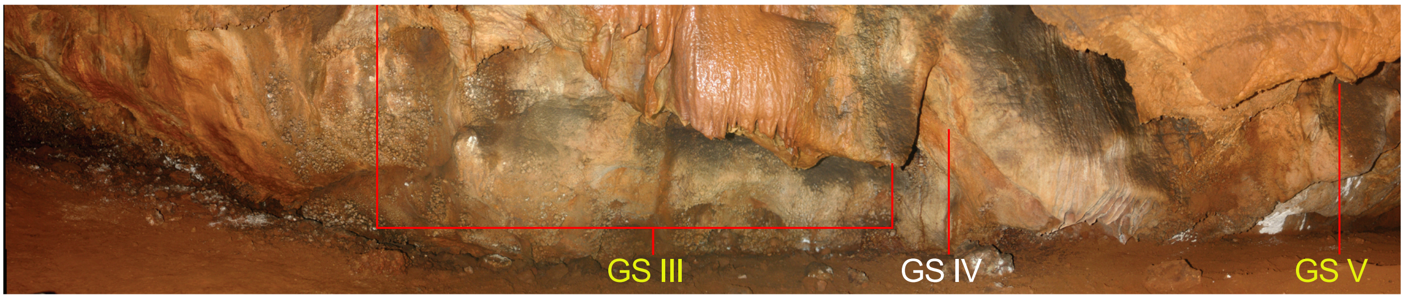

Sample ID	Area	Sample Type
SP.B.13	Spot 1 - Mano 53, panel GS-III	Control (Wall)
SP.B.15	Spot 1 - Mano 53, panel GS-III	Control (Wall)
SP.B.17	Spot 1 - Mano 53, panel GS-III	Control (Wall)
SP.B.18	Spot 1 - Mano 53, panel GS-III	Pigment (Wall)
SP.B.20	Spot 2 - Mano 53, panel GS-III	Control (Wall)
SP.B.23	Spot 2 - Mano 53, panel GS-III	Control (Wall)
SP.B.25	Spot 2 - Mano 53, panel GS-III	Pigment (Wall)
SP.B.28	Spot 1 - Mano 53, panel GS-III	Pigment (Wall)
SP.B.30	Mano 59, panel GS-V	Control (Wall)
SP.B.31	Mano 59, panel GS-V	Control (Wall)
SP.B.34	Mano 59, panel GS-V	Pigment (Wall)

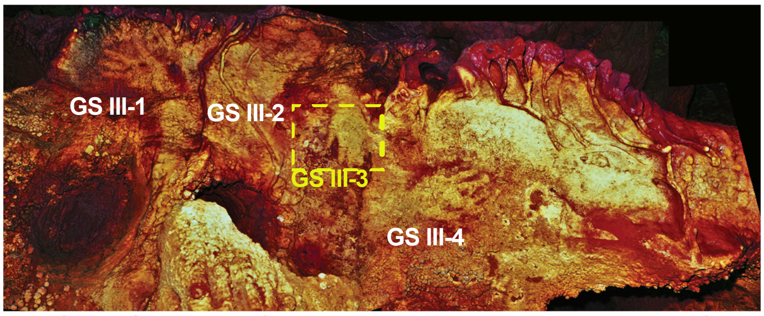

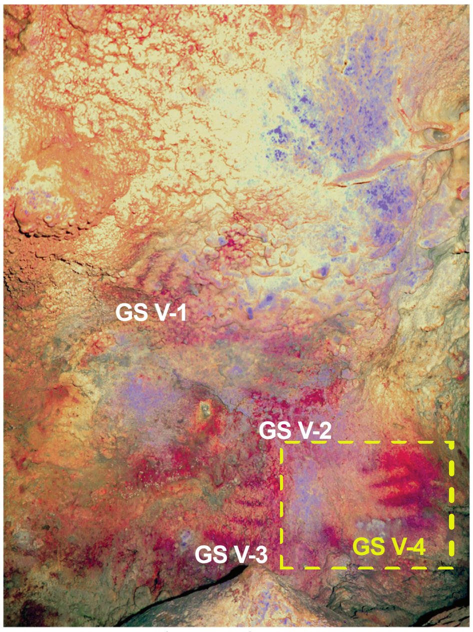

Fig. S8.2. Overview of areas sampled: Panels III, IV and V (upper); list of samples screened (middle); enlarged and enhanced view of Panel III (lower left) and Panel V (lower right) with sampled hand stencils highlighted in yellow.

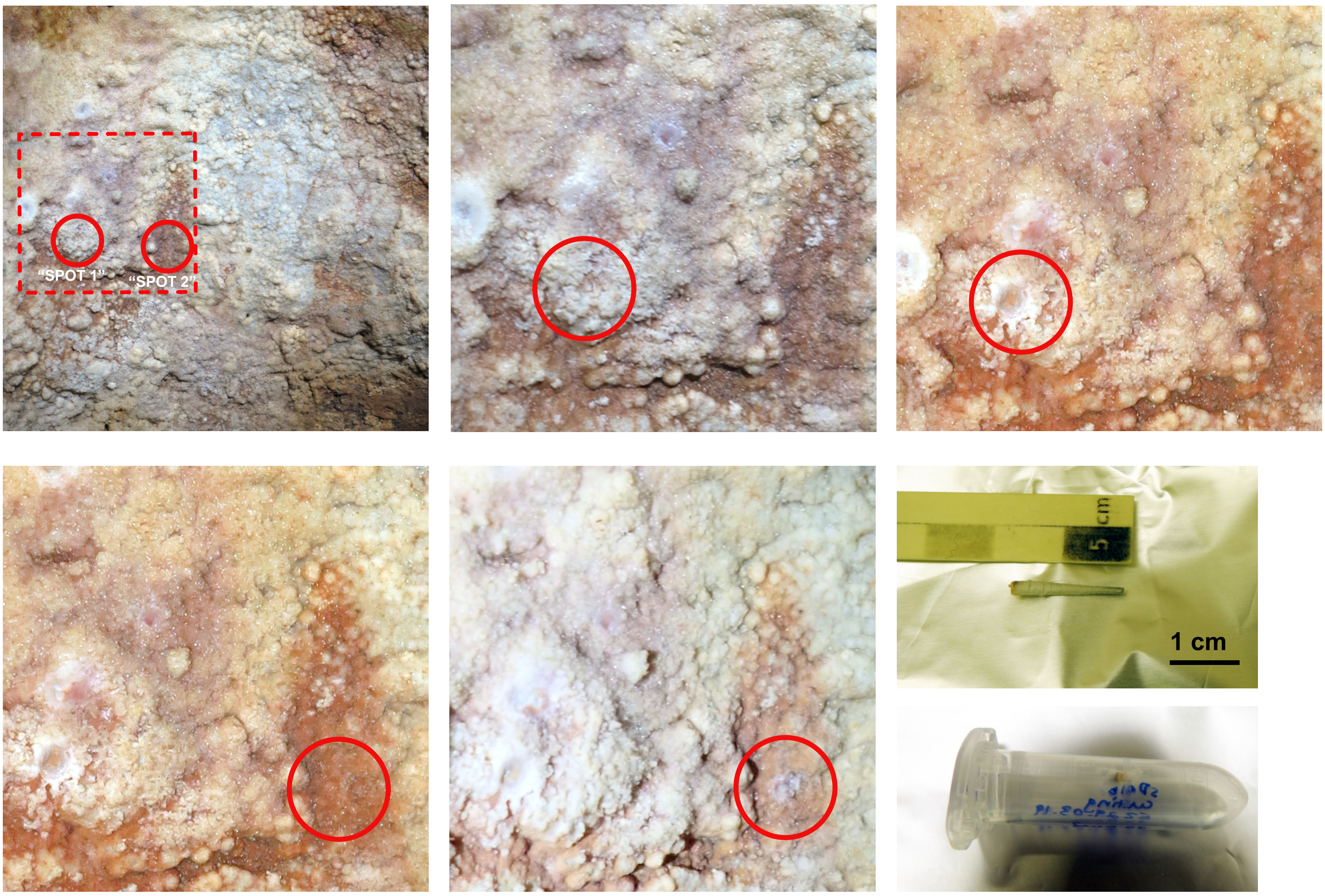

Fig. S8.3. Pigment and control sampling of hand stencil GS III-3 (SP.B.13 - SP.B.28): general location with the sampled area highlighted in red, showing annotated locations of samples (upper left); enlarged view of the cave wall before & after sampling "Spot 1" (upper right); before & after sampling "Spot 2" (lower left); representative example of sample (SP.B.25/ Spot 2) swab used: detached head and swab in tube (lower right).

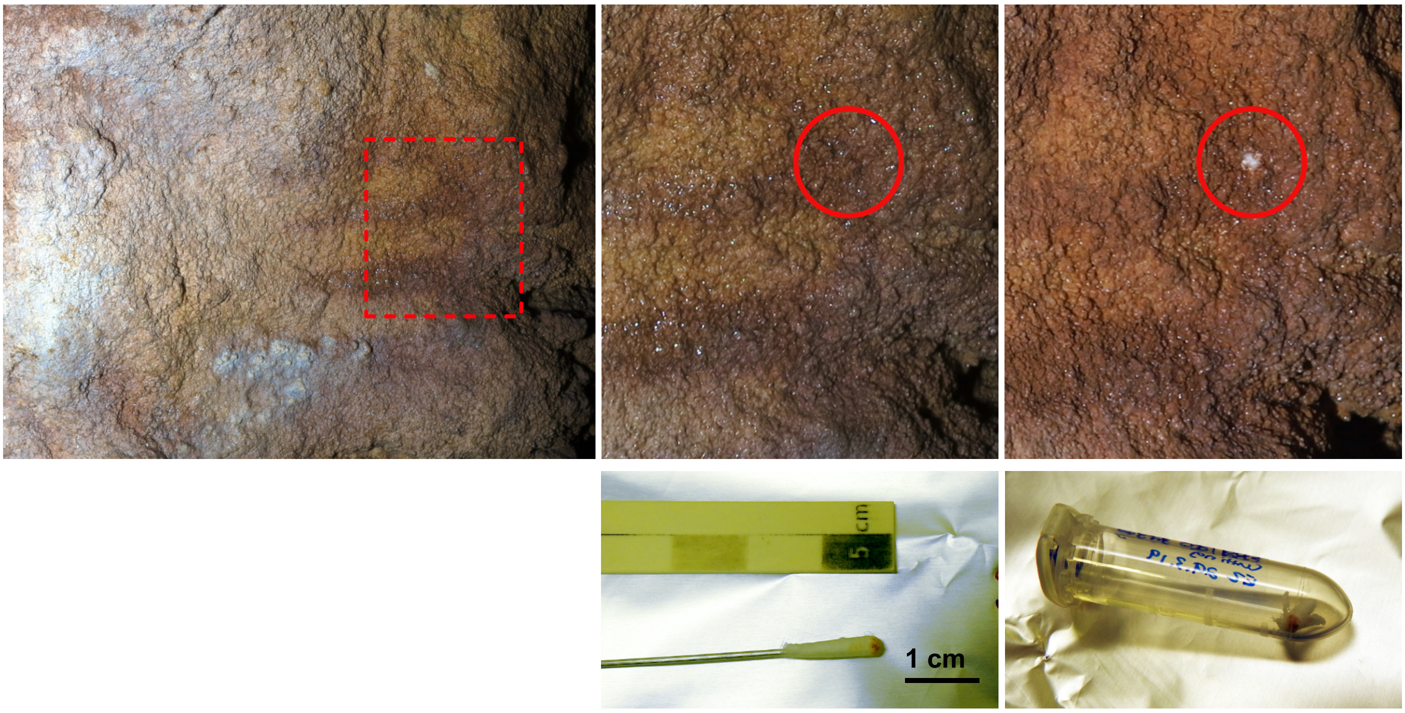

Fig. S8.4. Pigment and control sampling of hand stencil GS V-4 (SP.B.30 - SP.B.34): general location with the sampled area highlighted in red (upper left); enlarged view of the cave wall before sampling (upper middle); after sampling (upper right); sample SP.B.34 on sterile swab and detached in tube (lower right).

Sample ID	Area	Sample Type
SP.C.5518	At right of Pasaje 2.Fig. 8A/8B	Pigment (Wall)
SP.C.5519	Left of Fig. 8A/8B	Control (Wall)
SP.C.5520	Galería I: Fig. 3A	Pigment (Wall)
SP.C.5521	Galeria I: left of Fig. 3A	Control (Wall)
SP.C.5522	Galería I: Fig. 3 “Blown Disc”	Pigment (Wall)
SP.C.5523	Galería I: Fig. 4 “Fingertips”	Pigment (Wall)
SP.C.5524	Galería I: left of Fig. 3	Control (Wall)

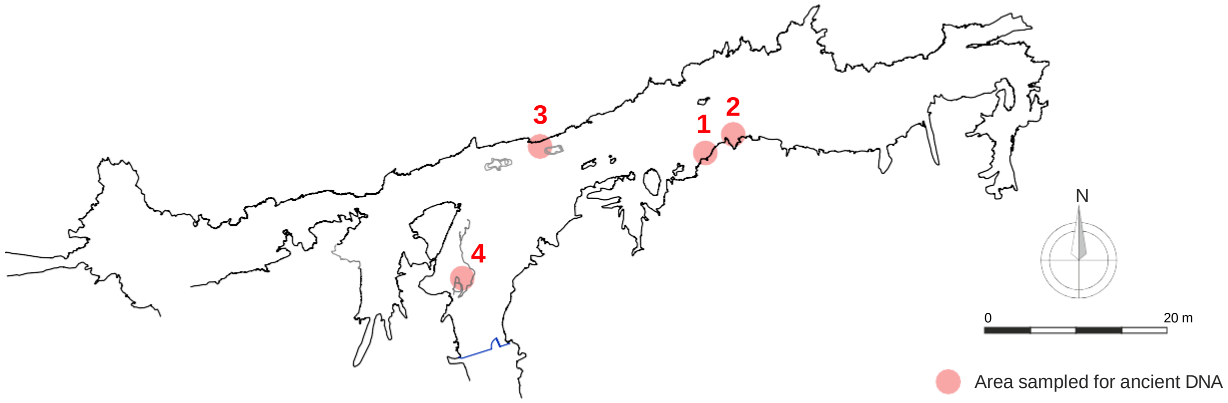

Fig. S9.1. List of samples screened (upper). Plan indicating areas sampled for DNA analyses in Cueva de Les Pedroses: Figure 8A/ 8B (1), Galería I (2), sediment sampling in Sector I (3), and sediment sampling in Sector III (4) (lower).

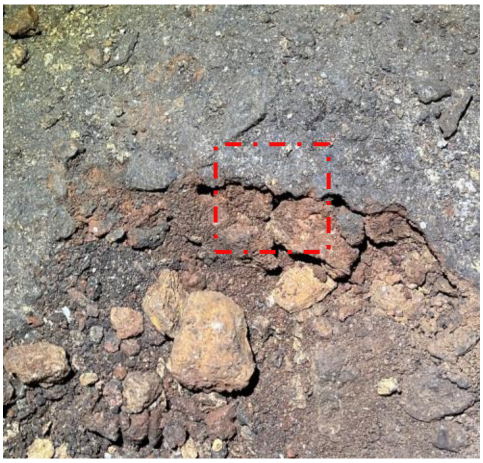

Sample ID	Area	Sample Type
SP.C.5525	Opposite “Galería I”	Sediment w/ Pigment
SP.C.5526	Opposite “Galería I”	Sediment w/ Pigment

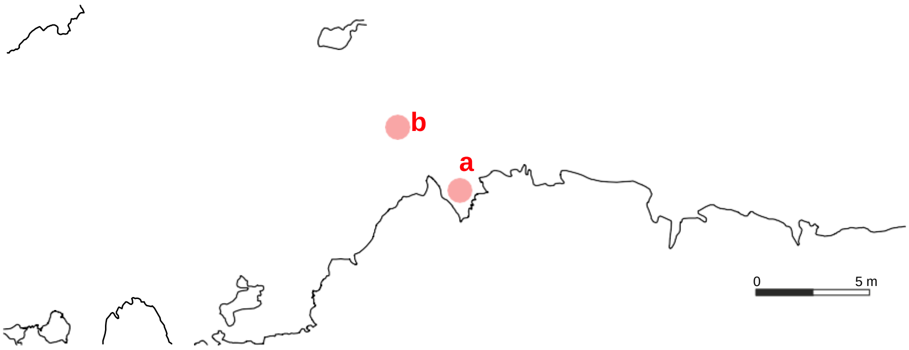

Fig. S9.2. Pigment samples in sediment SP.C.5525 & SP.C.5526: overview of sampled area highlighting sampled area in red (left); plan indicating areas sampled: wall pigments (a); pigment in sediment (b) (lower).

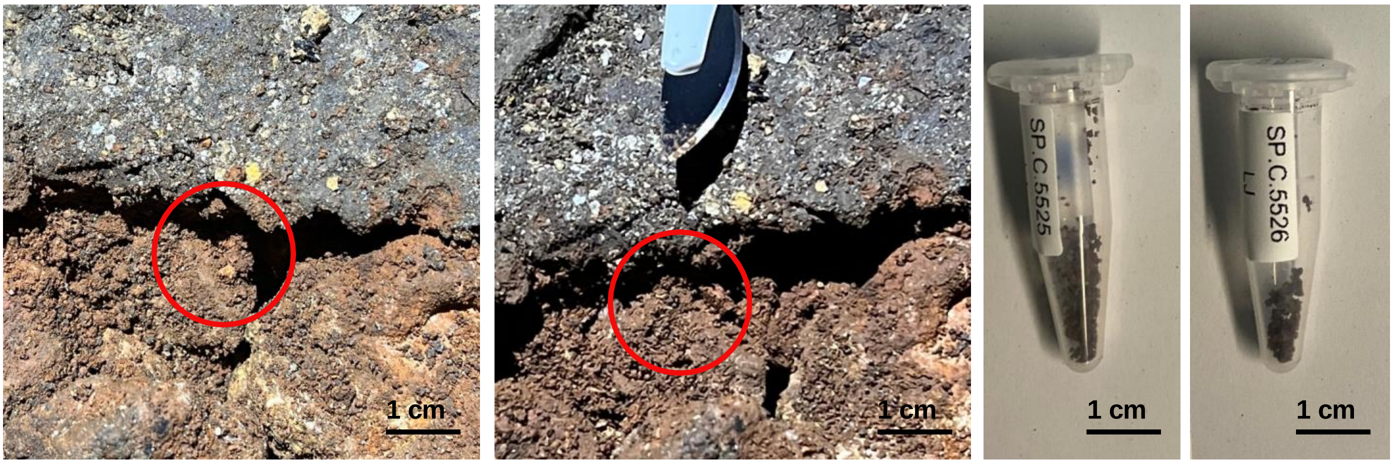

Fig. S9.3. Pigment samples in sediment SP.C.5525 & SP.C.5526: before sampling (left); after sampling (middle); successive samples in tubes (right).

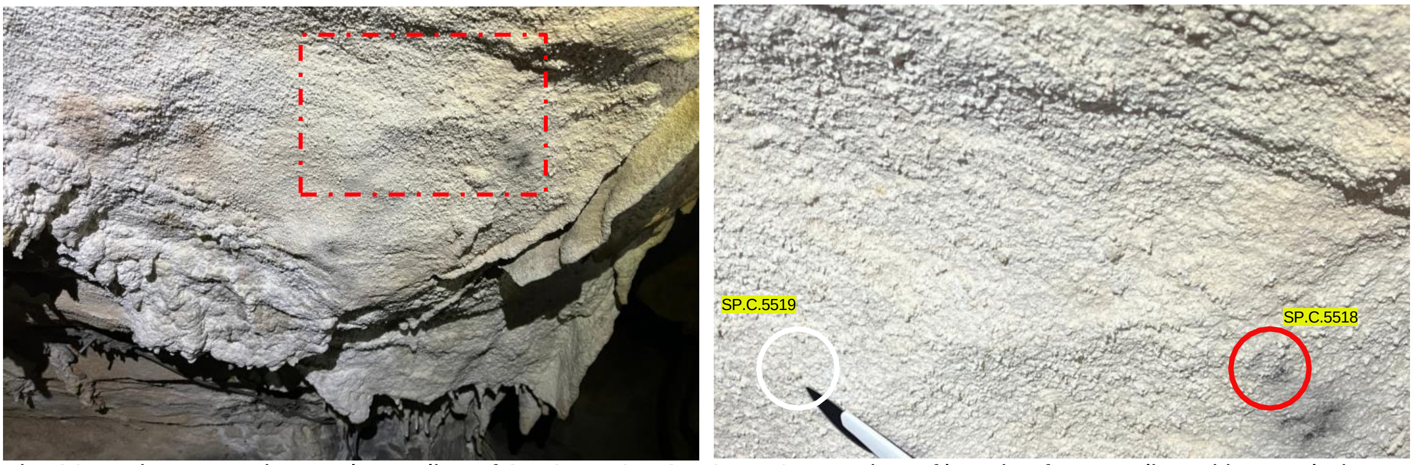

Fig. S9.4. Pigment and control sampling of SP.C.5518 & SP.C.5519: overview of location for sampling with sampled area highlighted in red (left); enlarged view of cave wall before sampling, showing annotated locations of samples (right).

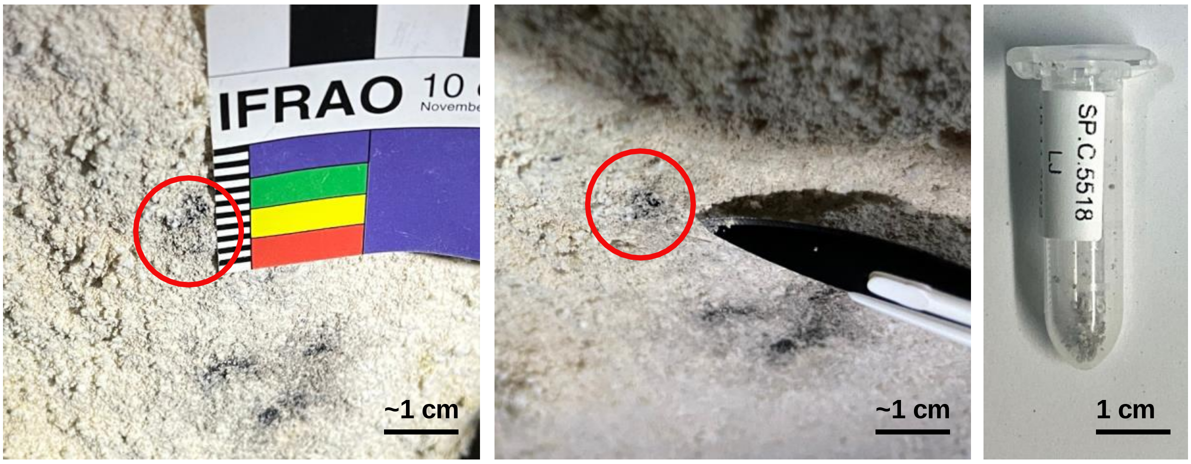

Fig. S9.5. Pigment sample SP.C.5518: cave wall before sampling (left); after sampling (middle); sample in tube (right).

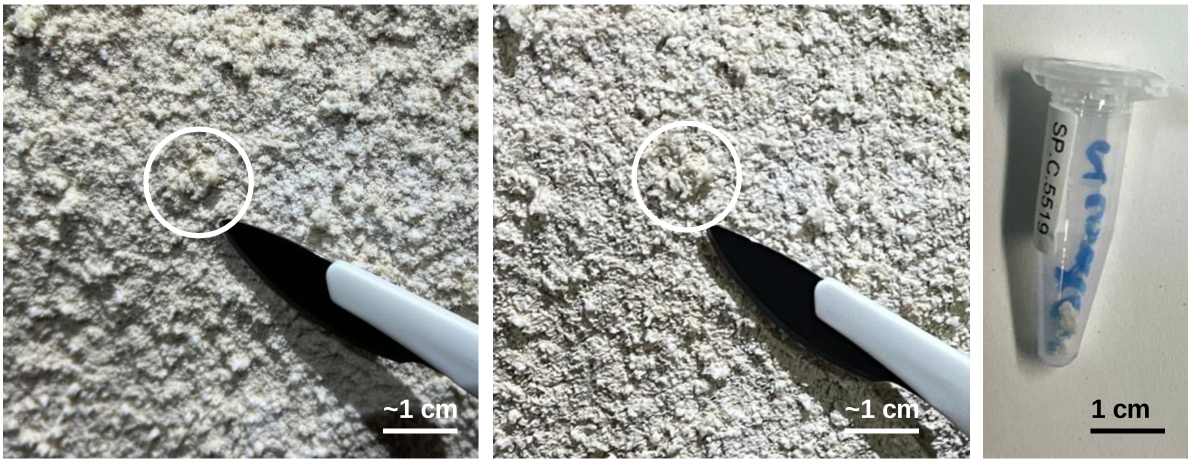

Fig. S9.6. Control sample SP.C.5519: cave wall before sampling (left); after sampling (middle); sample in tube (right).

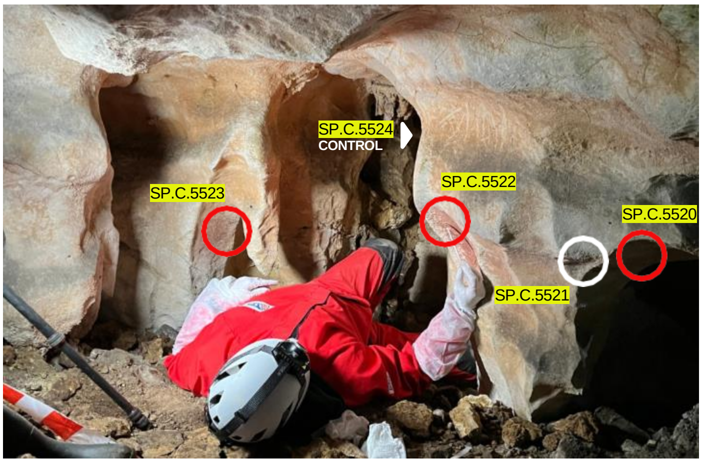

Fig. S9.7. General location of sampling pigments and controls SP.C.5520 - SP.C.5524.

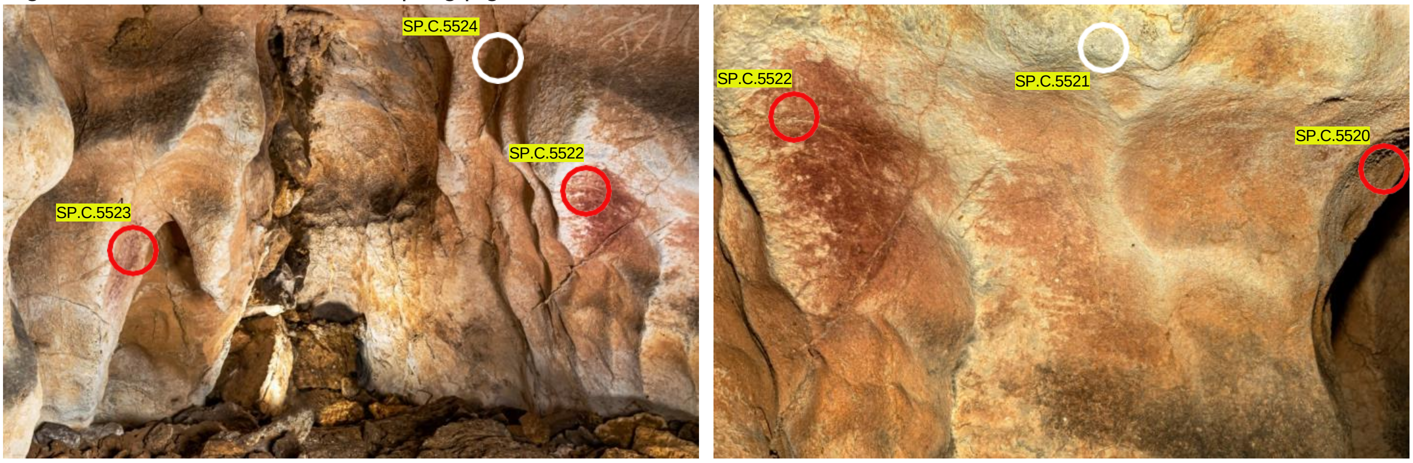

Fig. S9.8. Pigment and control sampling of SP.C.5522 - SP.C.5524 with annotated locations of samples (left); enlarged area of pigment & control sampling of SP.C.5520 & SP.C.5521, showing SP.C.5522 for reference (right).

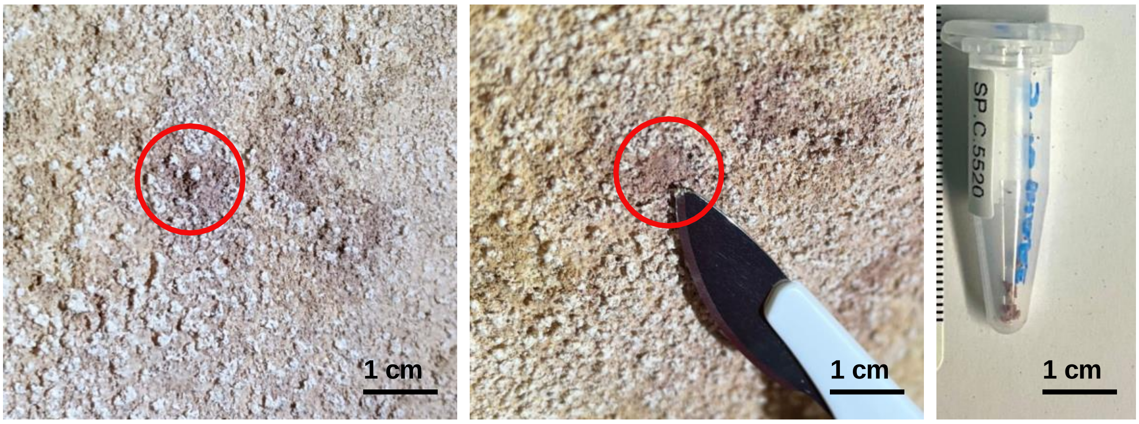

Fig. S9.9. Pigment sample SP.C.5520: cave wall before sampling (left); after sampling (middle); sample in tube (right).

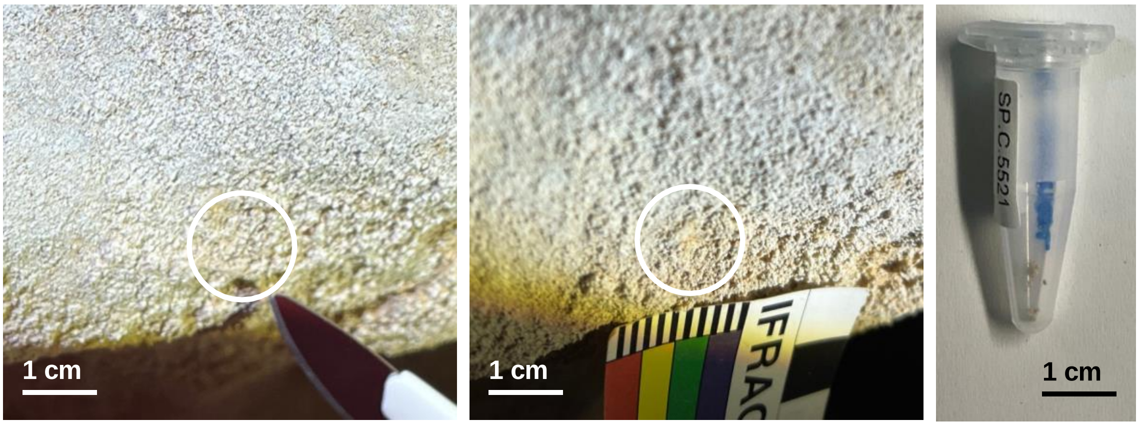

Fig. S9.10. Control sample SP.C.5521: cave wall before sampling (left); after sampling (middle); sample in tube (right).

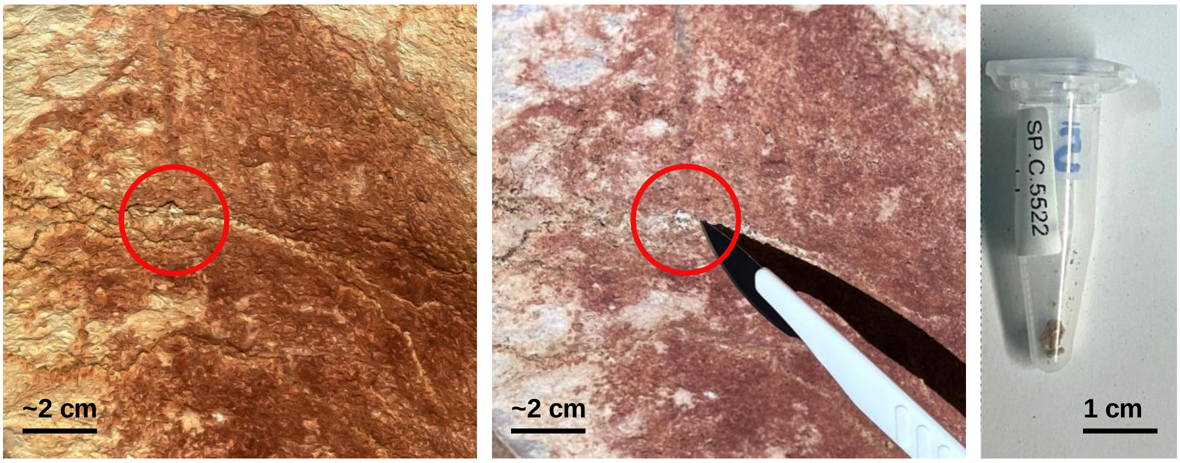

Fig. S9.11. Pigment sample SP.C.5522: cave wall before sampling (left); after sampling (middle); sample in tube (right).

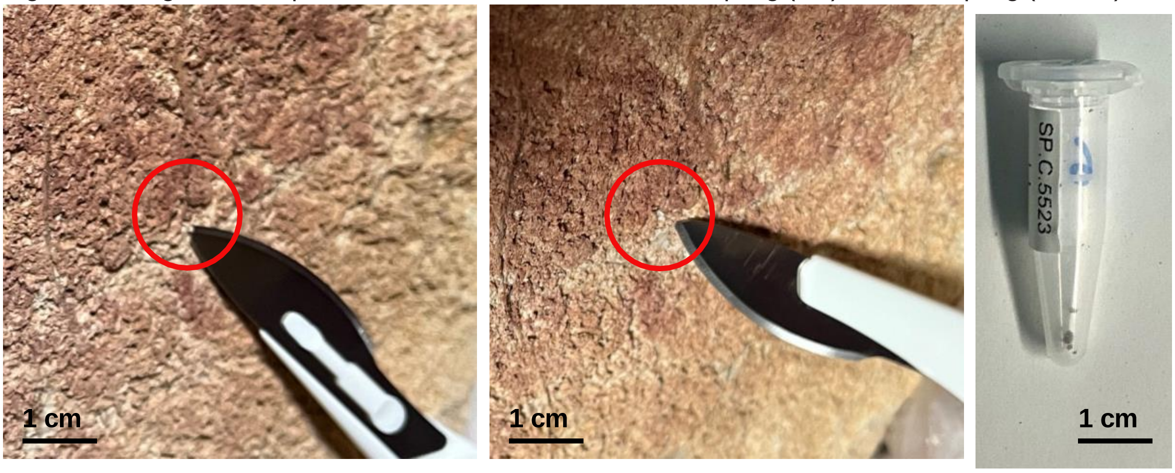

Fig. S9.12. Pigment sample SP.C.5523: cave wall before sampling (left); after sampling (middle); sample in tube (right).

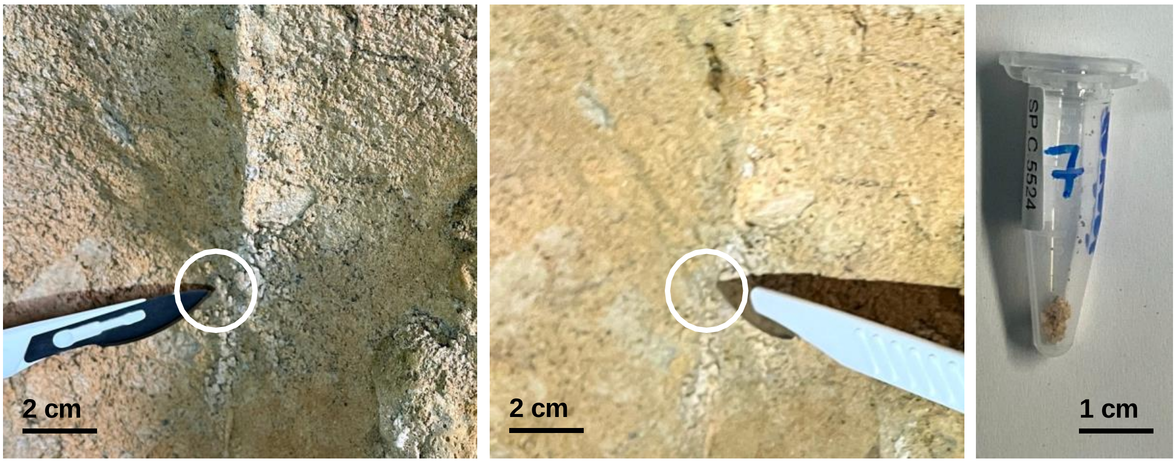

Fig. S9.13. Control sample SP.C.5524: cave wall before sampling (left); after sampling (middle); sample in tube (right).

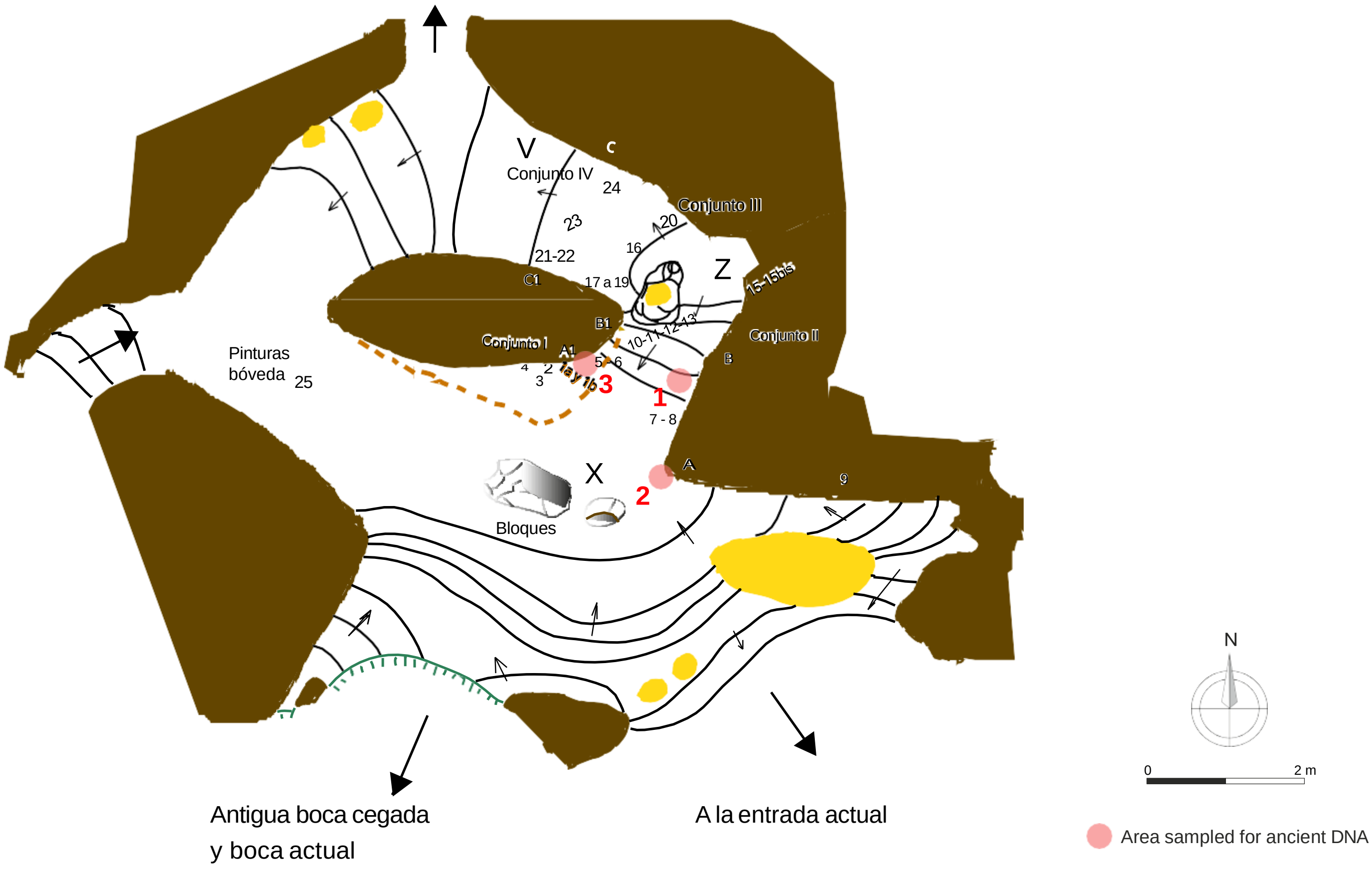

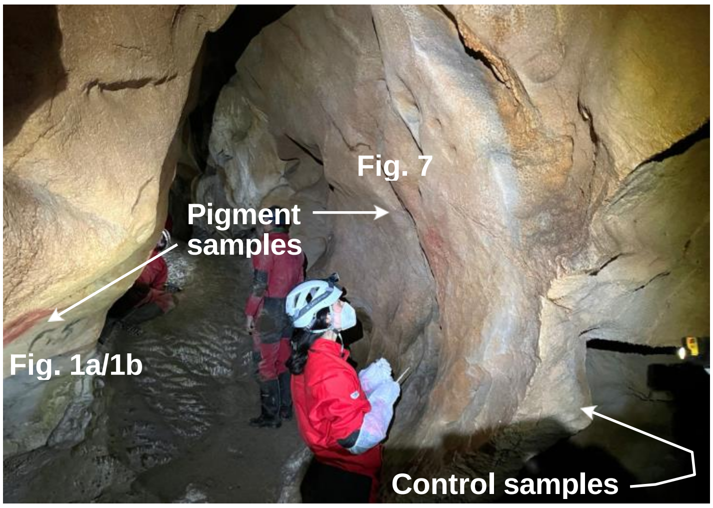

Sample ID	Area	Sample Type
SP.C.5508	Sala Central – Wall opposite Conjunto 1- Fig. 7	Pigment (Wall)
SP.C.5509	Sala Central – Wall opposite Conjunto 1	Control (Wall)
SP.C.5513	Sala Central- Conjunto 1 - Fig. 1a/1b	Pigment (Wall)
SP.C.5515	Sala Central- Conjunto 1 - Fig. 1a/1b	Pigment (Wall)
SP.C.5517	Sala Central- Conjunto 1 - Fig. 1a/1b	Pigment (Wall)

Fig. S10.1. Plan indicating areas sampled for DNA analyses in Cueva del Tebellín: Figure 7 (1), non-pigment controls (2), Figure 1a/1b (3) (upper); overview of areas sampled (lower left); list of samples screened (lower right).

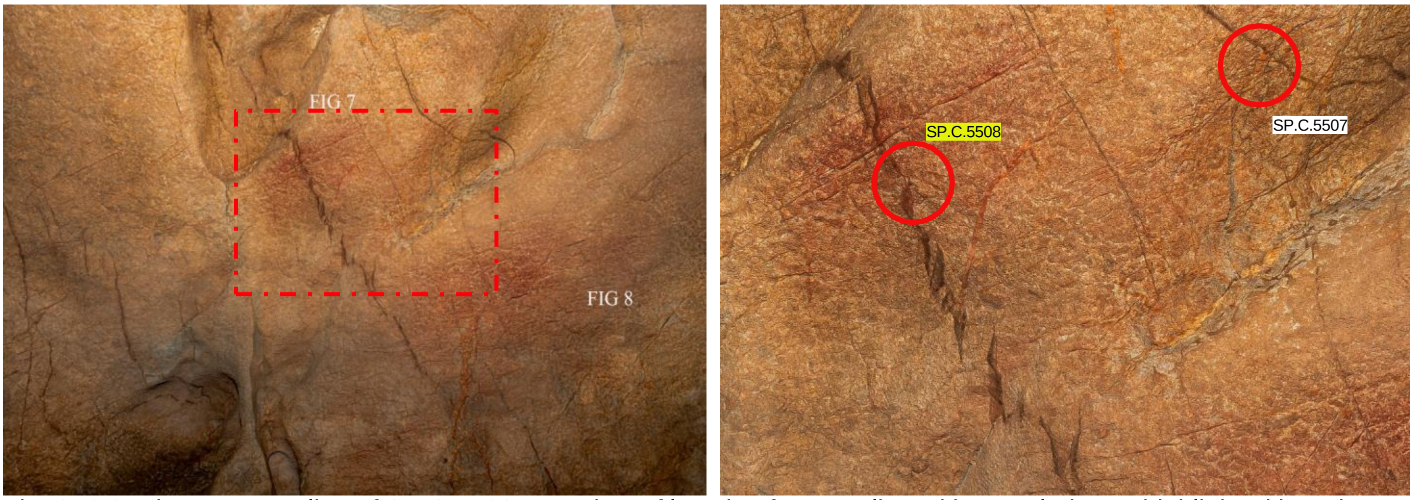

Fig. S10.2. Pigment sampling of SP.C.5508: overview of location for sampling with sampled area highlighted in red (left); enlarged view of cave wall before sampling showing annotated locations of samples (right).

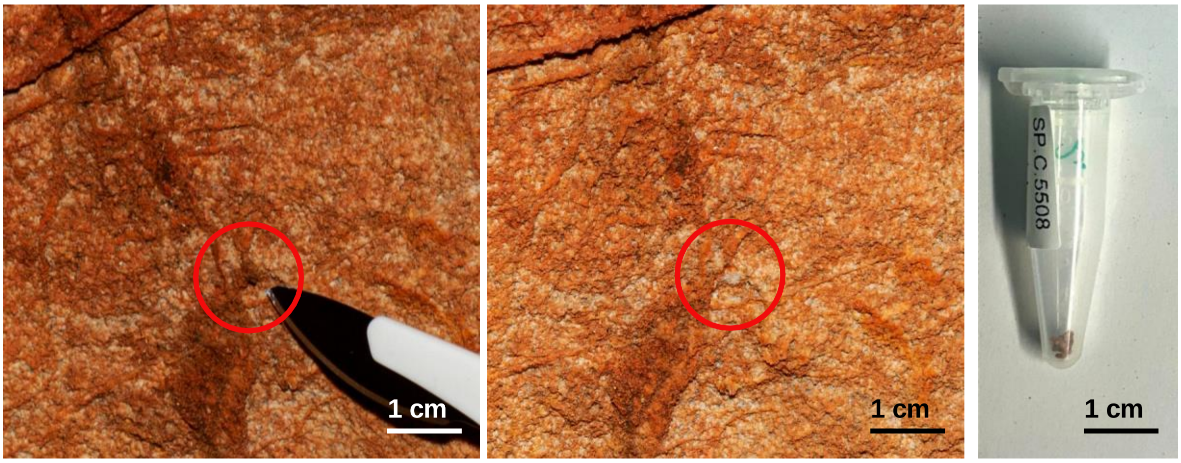

Fig. S10.3. Pigment sample SP.C.5508: cave wall before sampling (left); after sampling (middle); sample in tube (right).

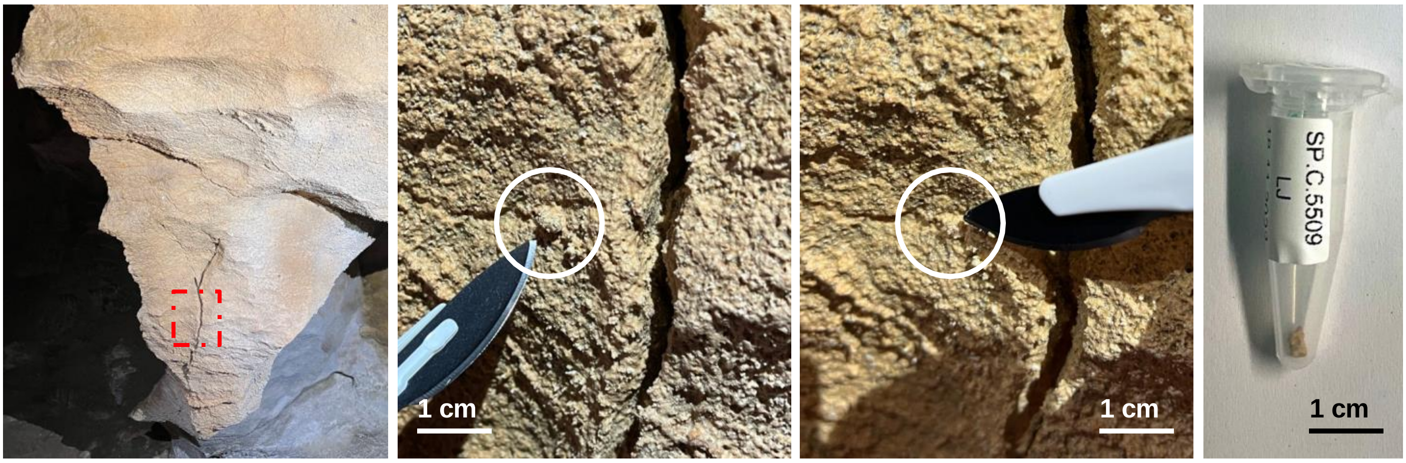

Fig. S10.4. Control sample SP.C.5509: overview of sampled area highlighted in red (left); enlarged view of cave wall before sampling - after sampling - sample in tube (right).

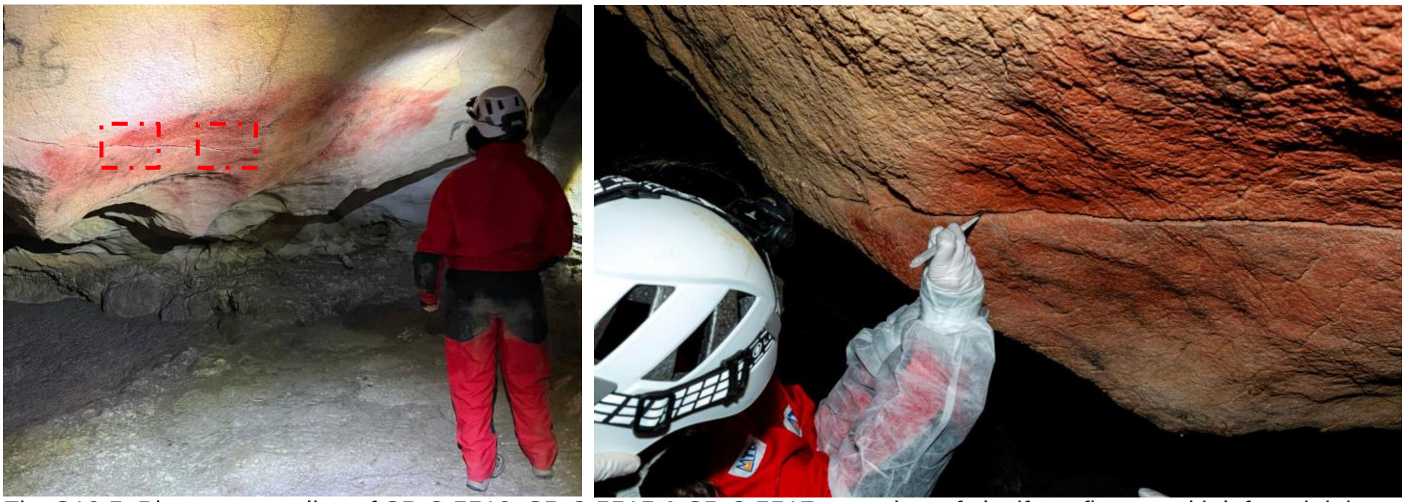

Fig. S10.5. Pigment sampling of SP.C.5513, SP.C.5515 & SP.C.5517: overview of claviform figures with left and right sampled areas highlighted in red (left); enlarged to show sampling of left area (right).

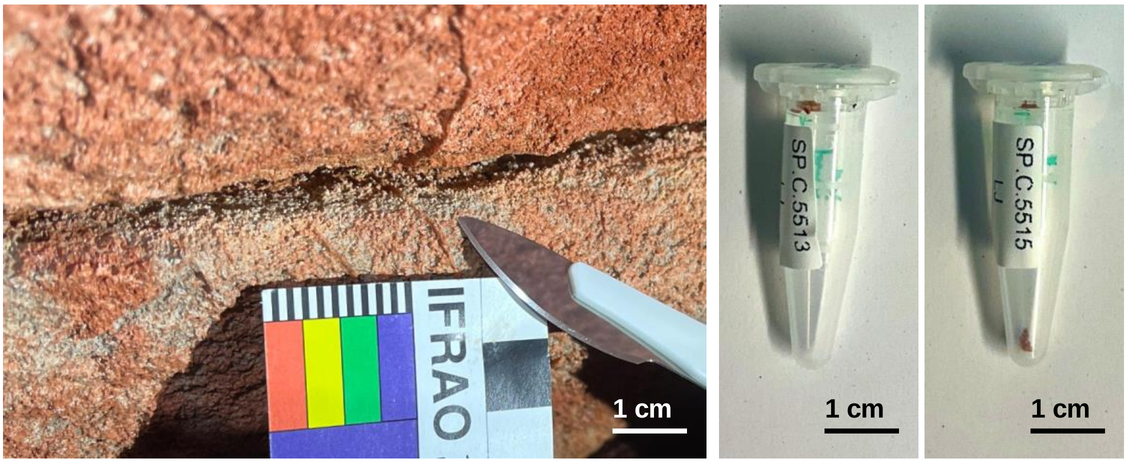

Fig. S10.6. Pigment samples SP.C.5513 & SP.C.5515: enlargement of left area of claviform from which samples were collected (left); samples in tubes (right).

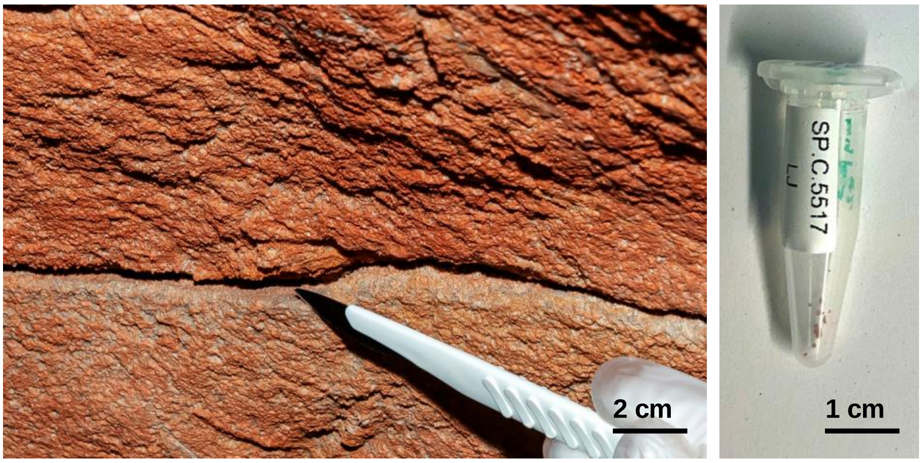

Fig. S10.7. Pigment sample SP.C.5517: enlargement of right area of claviform from which samples were collected (left); sample in tube (right).

Sample ID	Area	Sample Type
SP.C.5469	Galeria de los Discos	Pigment (Wall)
SP.C.6832	Galeria de los Discos	Pigment (Wall)
SP.C.6834	Galeria de los Discos	Pigment (Wall)
SP.C.6835	Galeria de los Discos	Control (Wall)
SP.C.6839	Galeria de los Discos	Control (Wall)
SP.C.6841	Sala de las Pinturas. Adjacent wall	Pigment (Wall)
SP.C.6842	Sala de las Pinturas. Adjacent wall	Control (Wall)

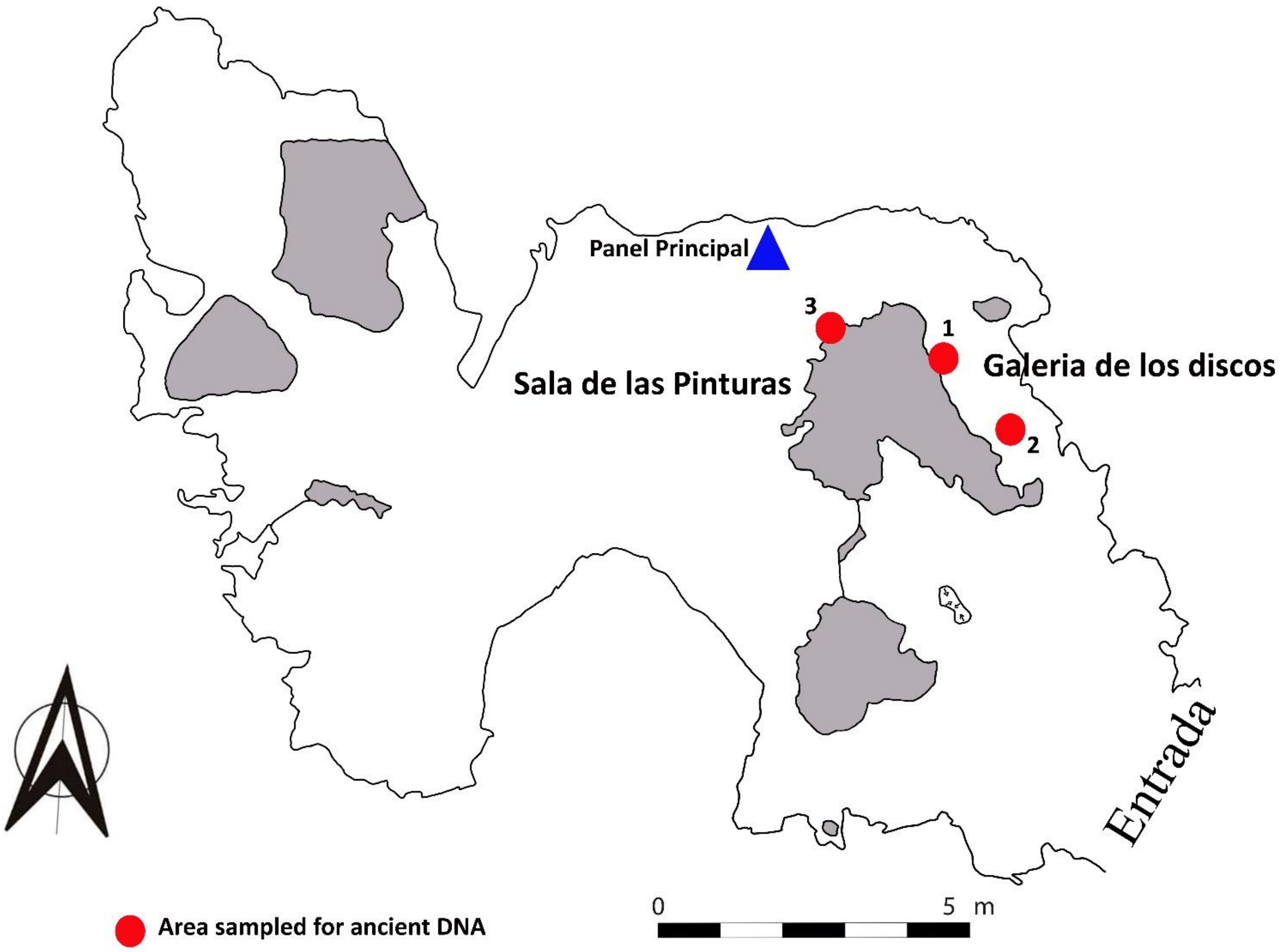

Fig S11.1. Plan indicating areas sampled for DNA analyses in Cueva del Toro: Galeria de los Discos, sampling spots CTO-2 and CTO-3 (1), Galeria de los Discos, control samples (2), Sala de las Pinturas, Adjacent wall (3); list of samples screened (upper right).

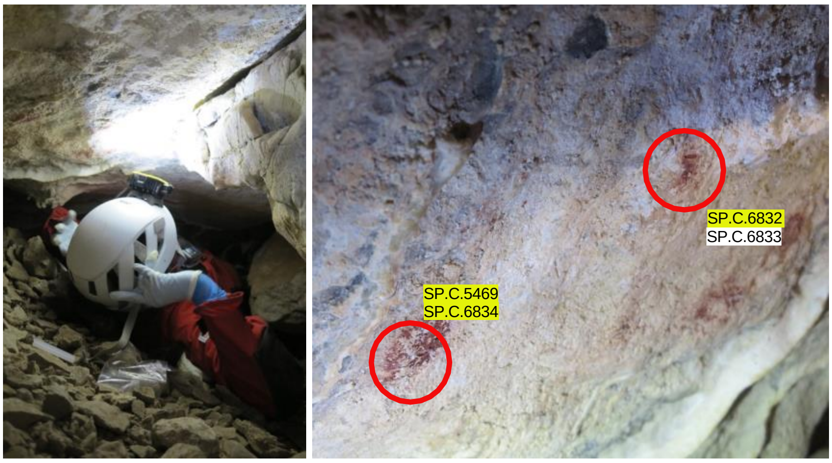

Fig S11.2. Pigment and control sampling of SP.C.5469 (2022) & SP.C.6832 - SP.C.6840: general location (left); annotated locations of pigment samples (right).

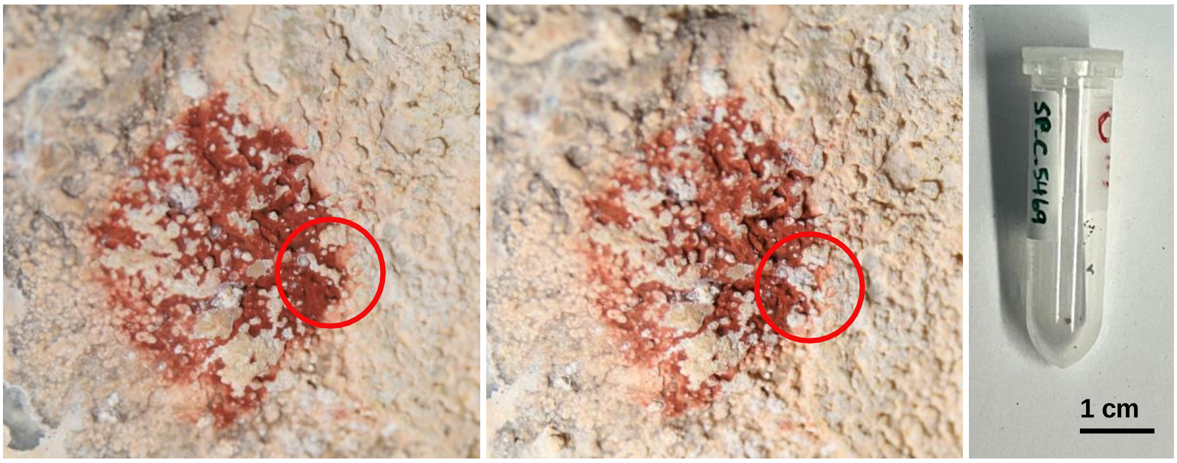

Fig S11.3. Pigment sample SP.C.5469: cave wall before sampling (left); after sampling (middle); sample in tube (right).

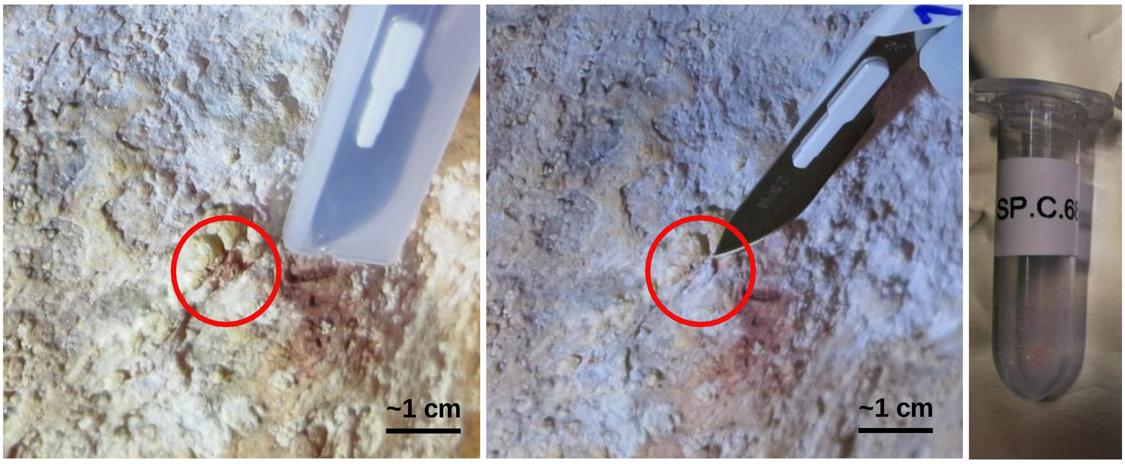

Fig S11.4. Pigment sample SP.C.6832: cave wall before sampling (left); after sampling (middle); sample in tube (right).

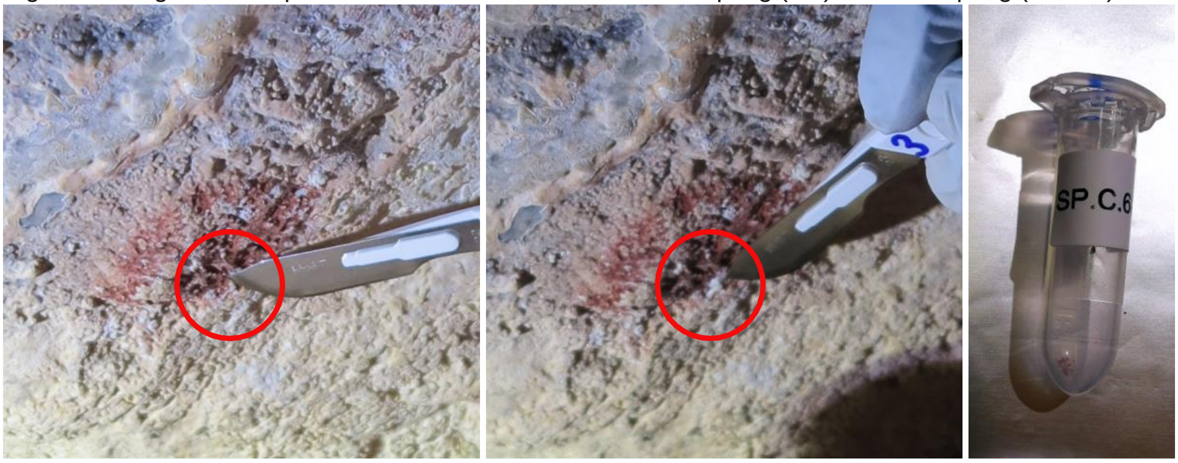

Fig S11.5. Pigment sample SP.C.6834: cave wall before sampling (left); after sampling (middle); sample in tube (right).

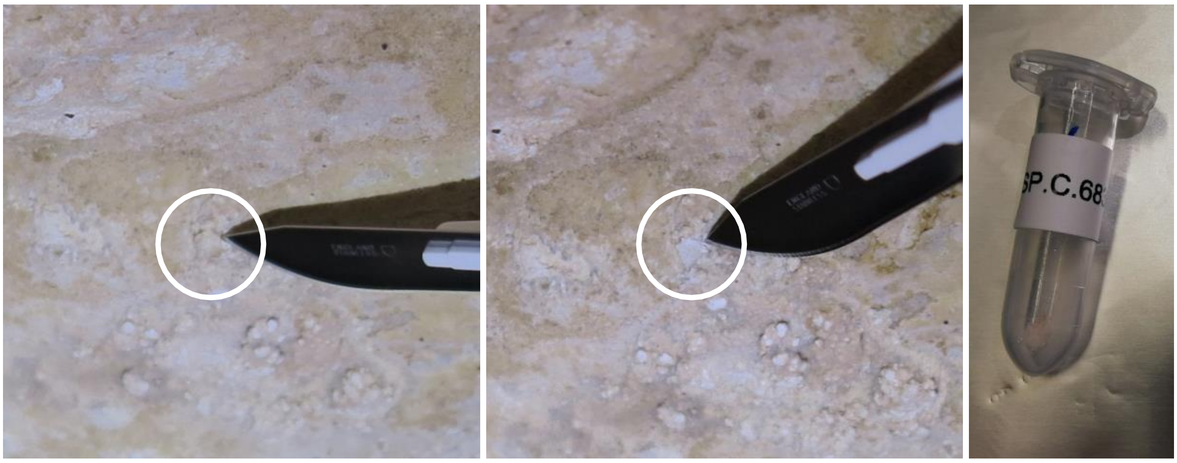

Fig S11.6. Control sample SP.C.6835: cave wall before sampling (left); after sampling (middle); sample in tube (right).

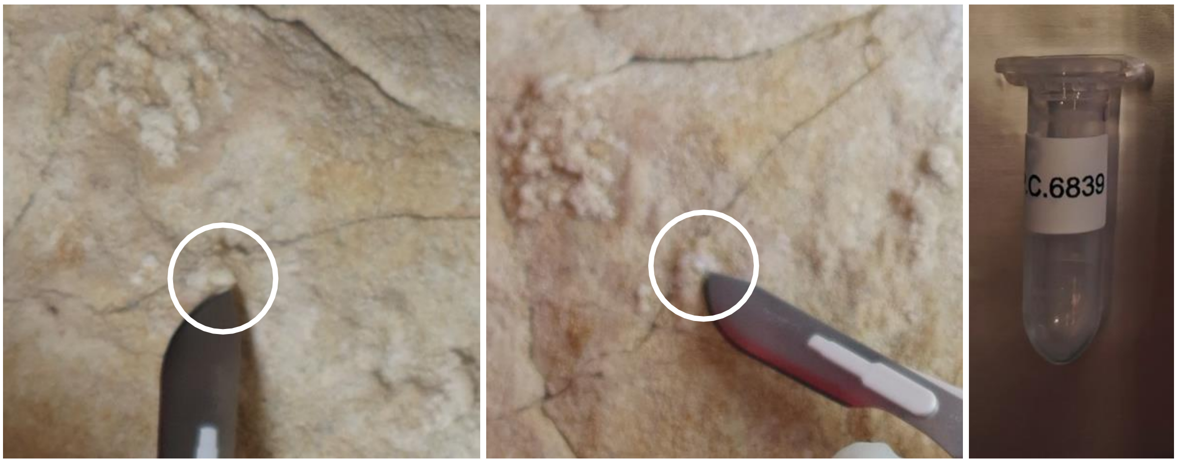

Fig S11.7. Control sample SP.C.6839: cave wall before sampling (left); after sampling (middle); sample in tube (right).

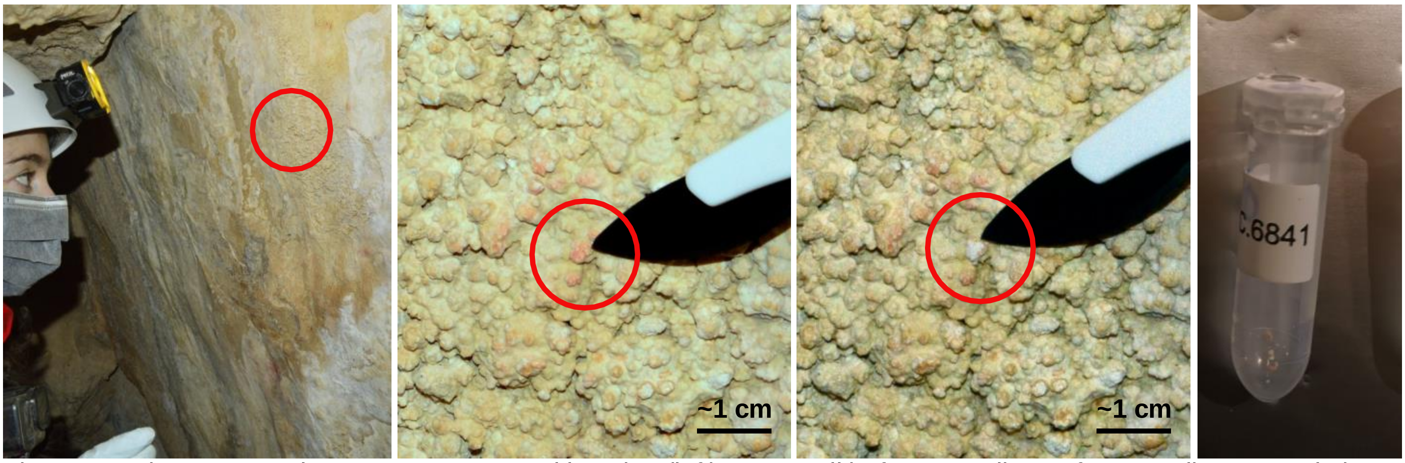

Fig S11.8. Pigment sample SP.C.6841: general location (left); cave wall before sampling - after sampling - sample in tube (right).

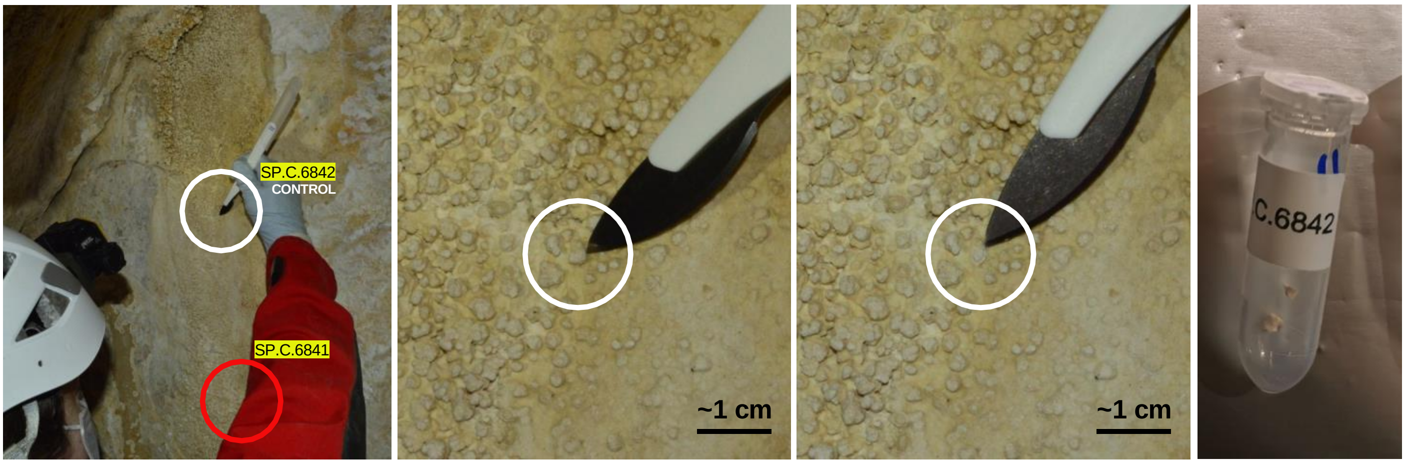

Fig S11.9. Control sample SP.C.6842: general location (left); cave wall before sampling - after sampling - sample in tube (right).

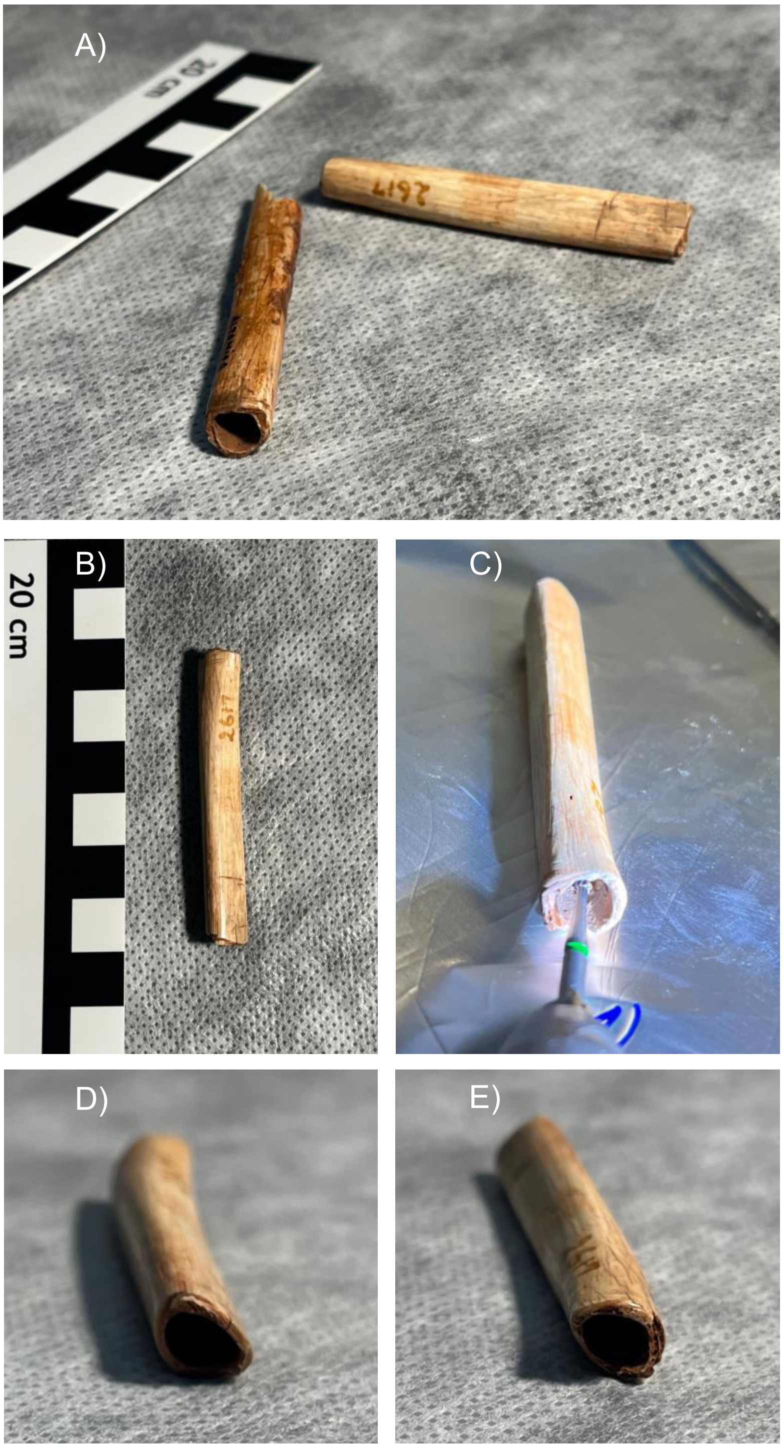

Fig. S12: Altamira airbrush A) Overview of the two bird bone diaphyses used together as an airbrush, B) Dimensions of ALT2617, the bone that was sampled, C) Sampling approach, D) End of the bone sampled for the first three micro-samples and E) End of the bone sampled for the other three micro-samples.

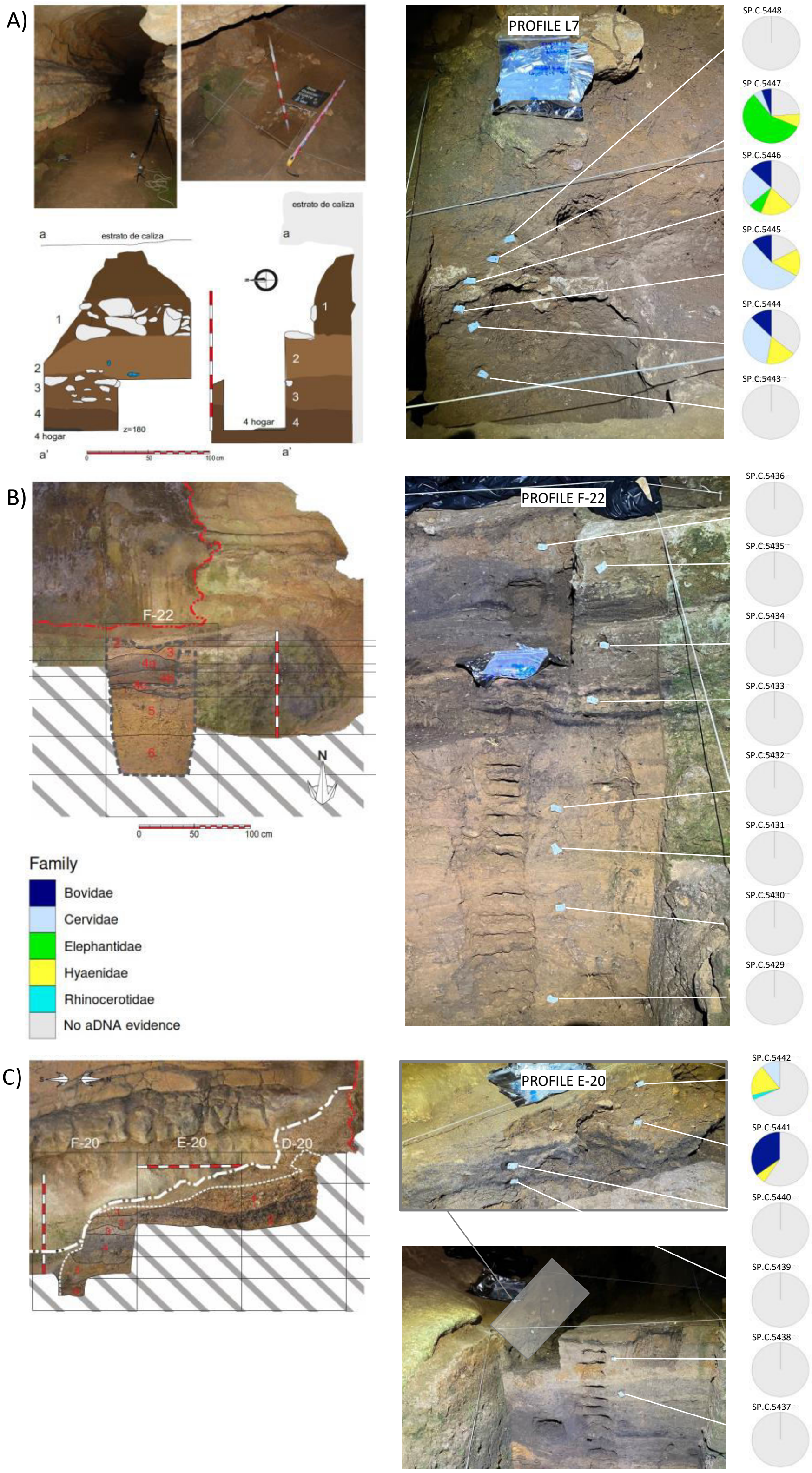

Fig. S13: Archaeological sediment profiles sampled at Cudón: A) Profile L-7, B) Profile F-22 and C) Profile E-20. The pie charts on the right show the relative abundance of mtDNA fragments assigned to 'ancient' biological families based on mammalian mtDNA capture. Fragments assigned to families lacking sufficient evidence for ancient DNA are shown in grey. Source data are provided as a Source Data file.

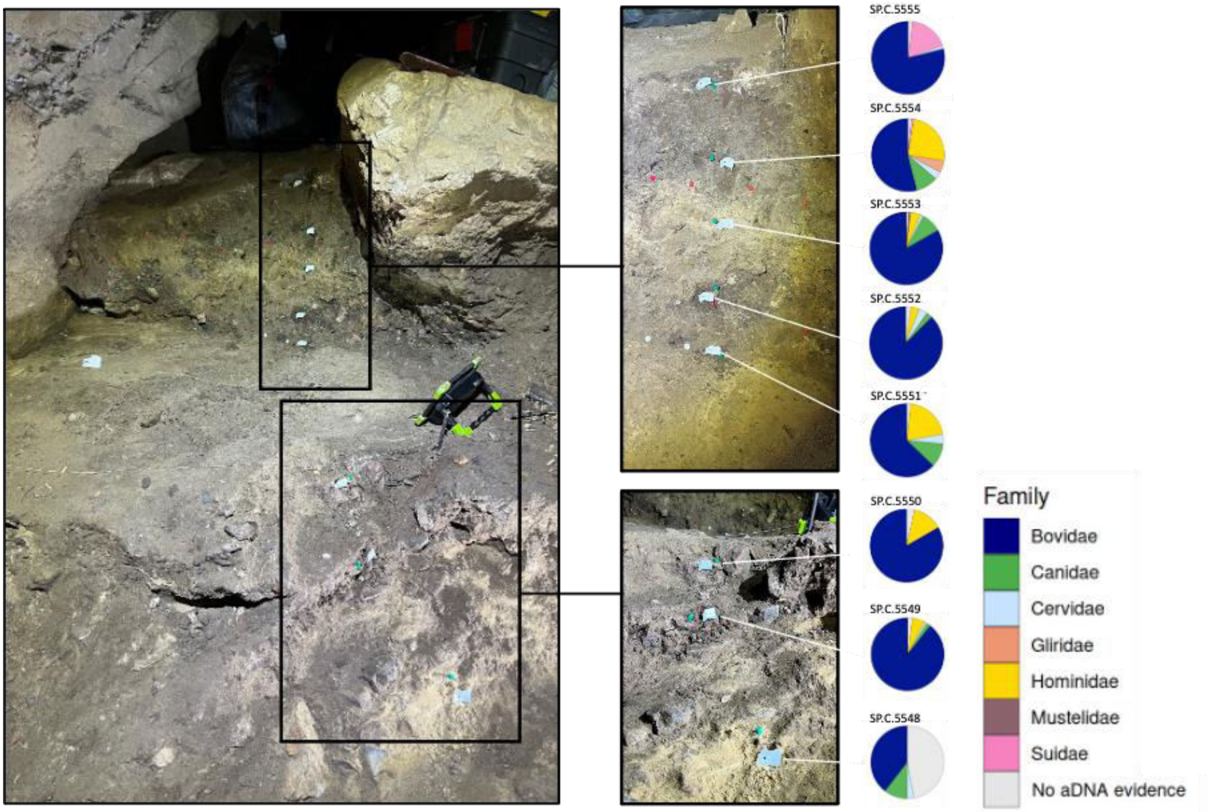

Fig. S14: Archaeological sediment profiles sampled at the Main Hall of Covarón. The pie charts on the right show the relative abundance of mtDNA fragments assigned to 'ancient' biological families based on mammalian mtDNA capture. Fragments assigned to families lacking sufficient evidence for ancient DNA are shown in grey. Source data are provided as a Source Data file.

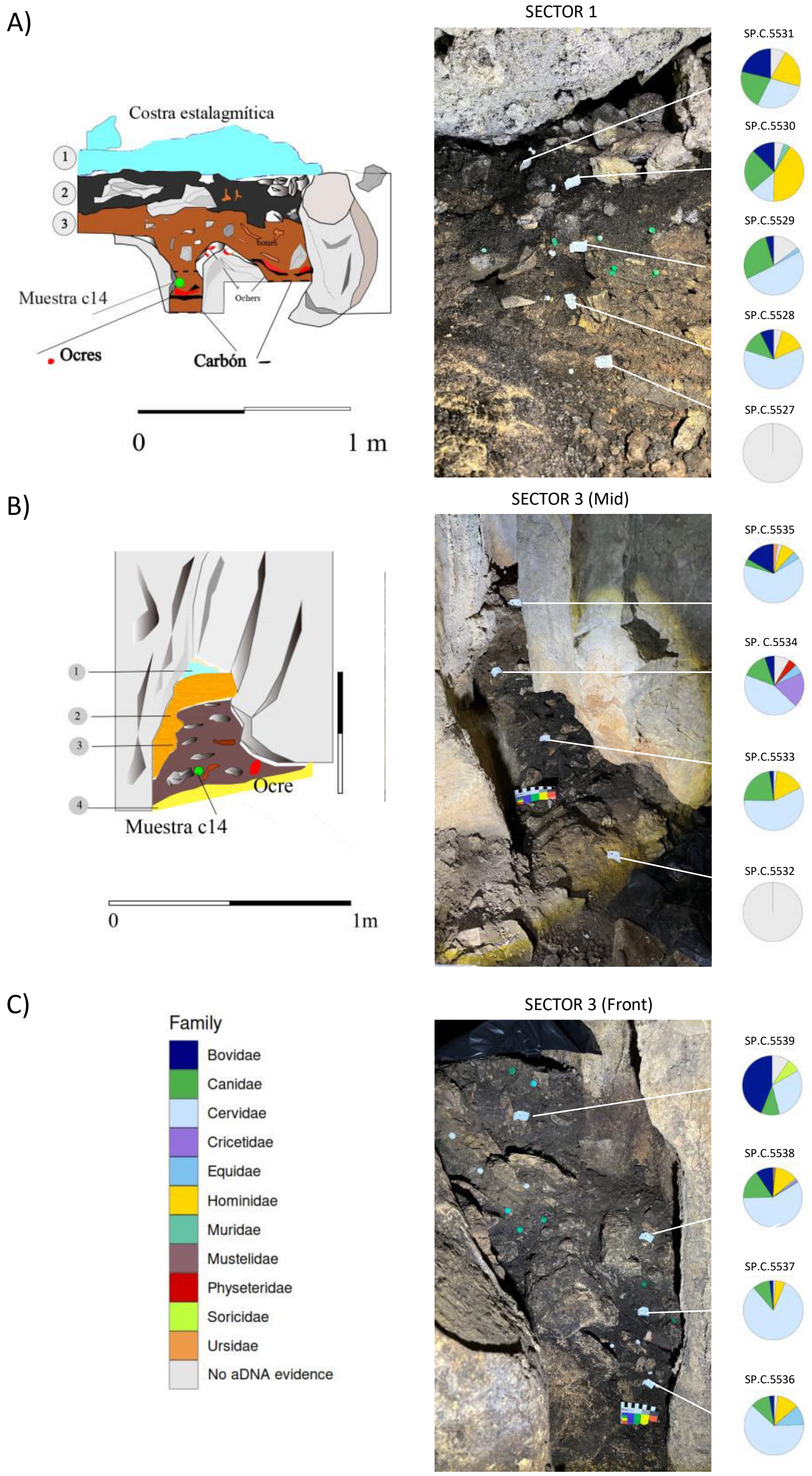

Fig. S15: Archaeological sediment profiles sampled at Les Pedroses in A) Sector 1, B) Sector 3 Mid and C) Sector 3 Front. The pie charts on the right show the relative abundance of mtDNA fragments assigned to 'ancient' biological families based on mammalian mtDNA capture. Fragments assigned to families lacking sufficient evidence for ancient DNA are shown in grey. Source data are provided as a Source Data file.

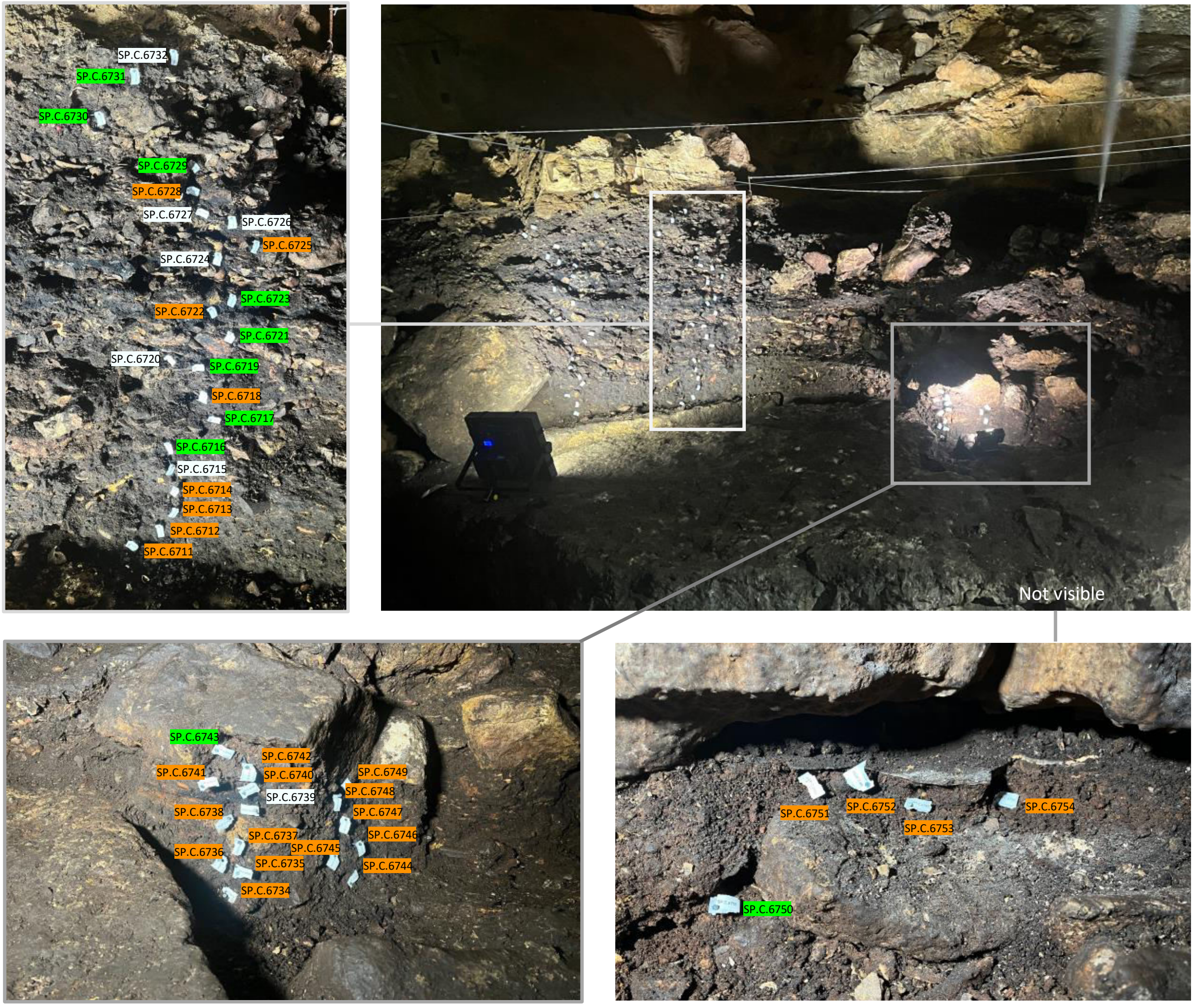

Fig. S16: Archaeological sediment profiles sampled at Altamira. Samples with green labels showed evidence for the preservation of ancient human mtDNA, samples with orange labels were negative, samples with white labels were not analysed in this study.

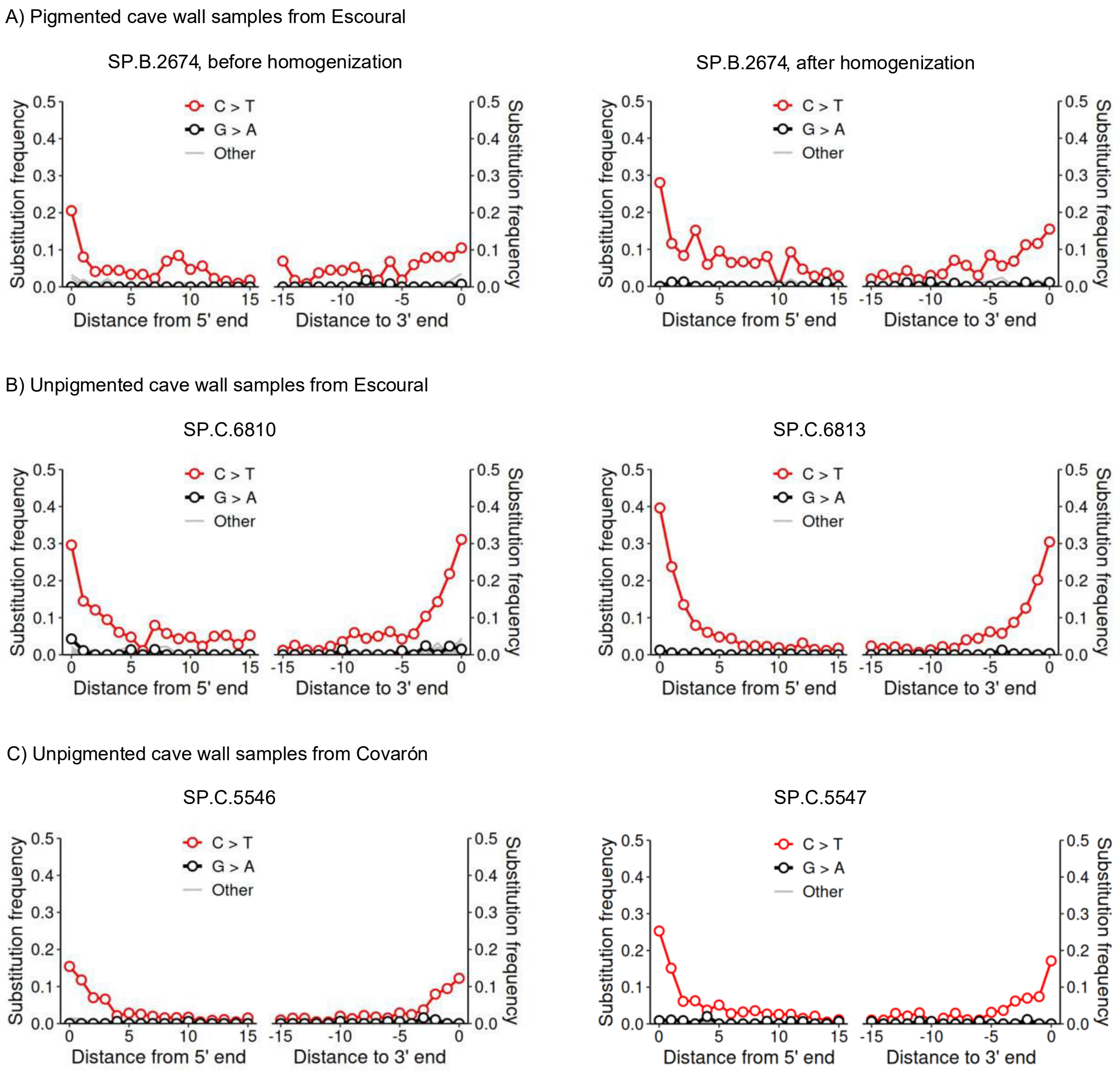

Fig. S17: Terminal C-to-T substitution frequencies in A) the two libraries prepared from the pigmented cave wall samples from Panel 11 in Escoural, B) Escoural (SP.C.6810 and SP.C.6813), and C) Covarón (SP.C.5546 and SP.C.5547). Source data are provided as a Source Data file.

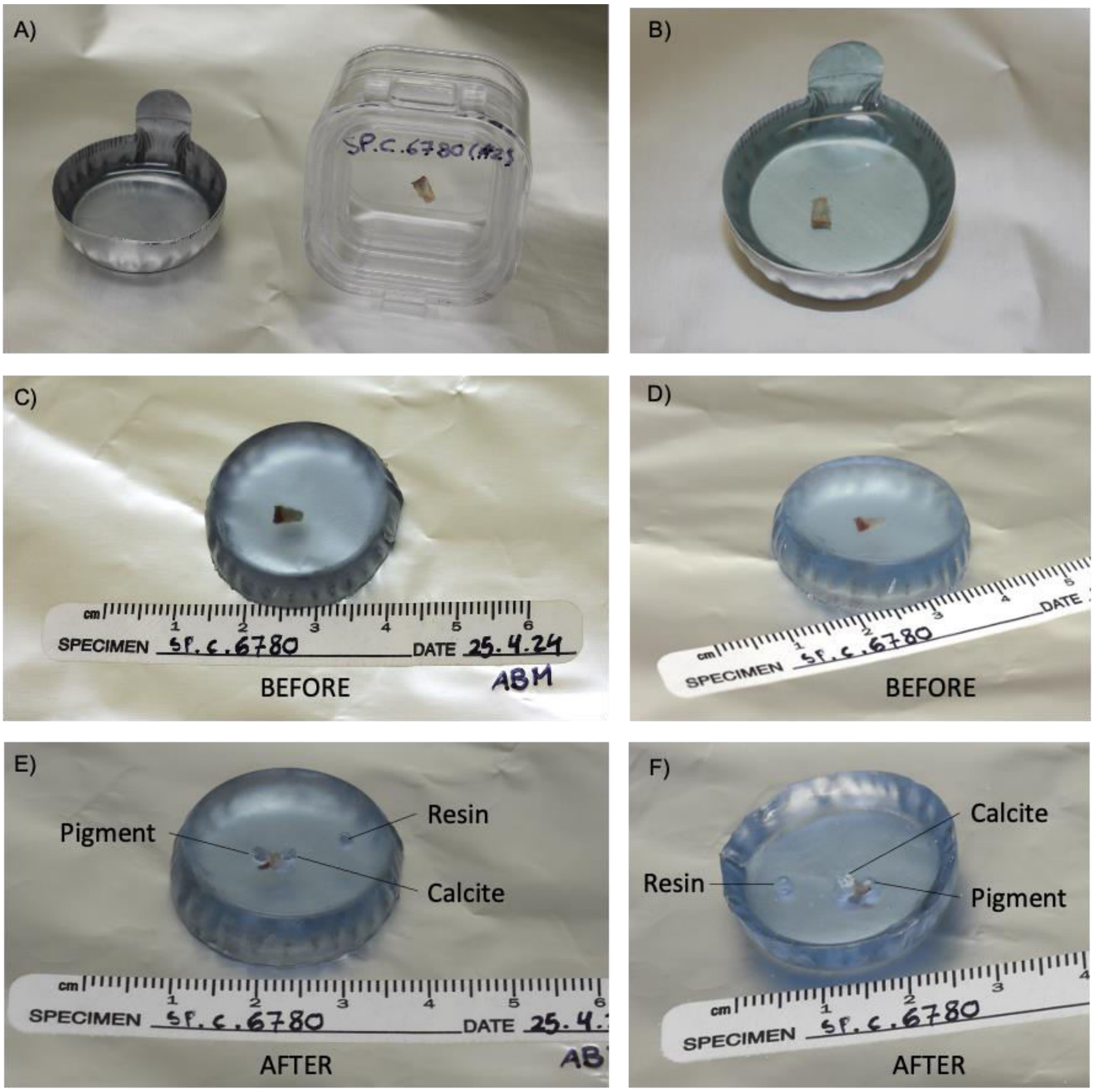

Fig. S18: Impregnated pigmented cave wall sample from Escoural A) Overview of the sample stored in a membrane box (left) and the mould used for the resin impregnation (right), B) Sample during impregnation, C and D) Before sampling, E and F) After sampling: sub-samples include resin (not processed), calcite (Sub.C.8160) and pigment (Sub.C.8163).

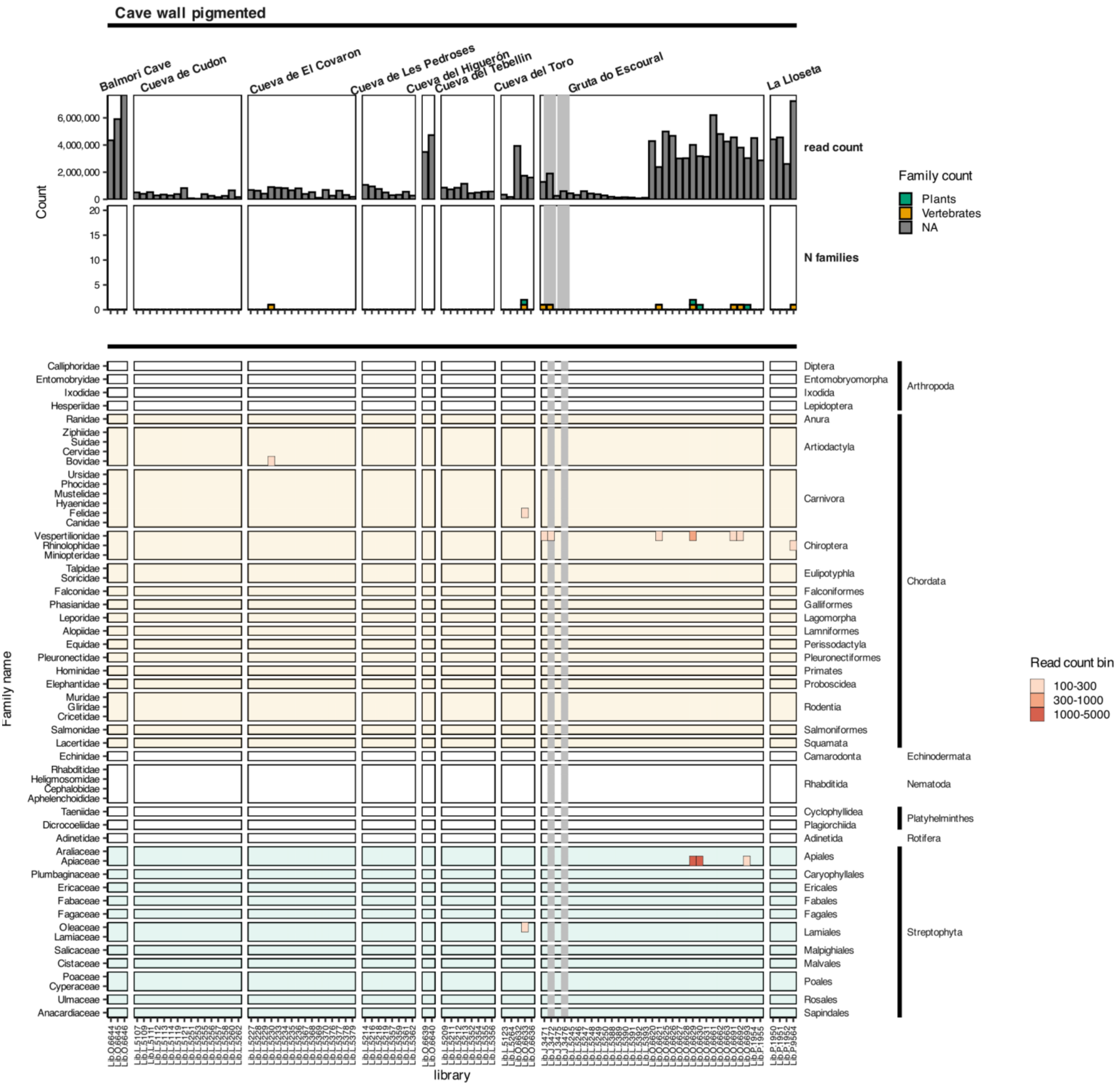

Fig. S19: Metagenomic analysis of shotgun data obtained from the pigmented cave wall samples.

Source data are provided as a Source Data file.

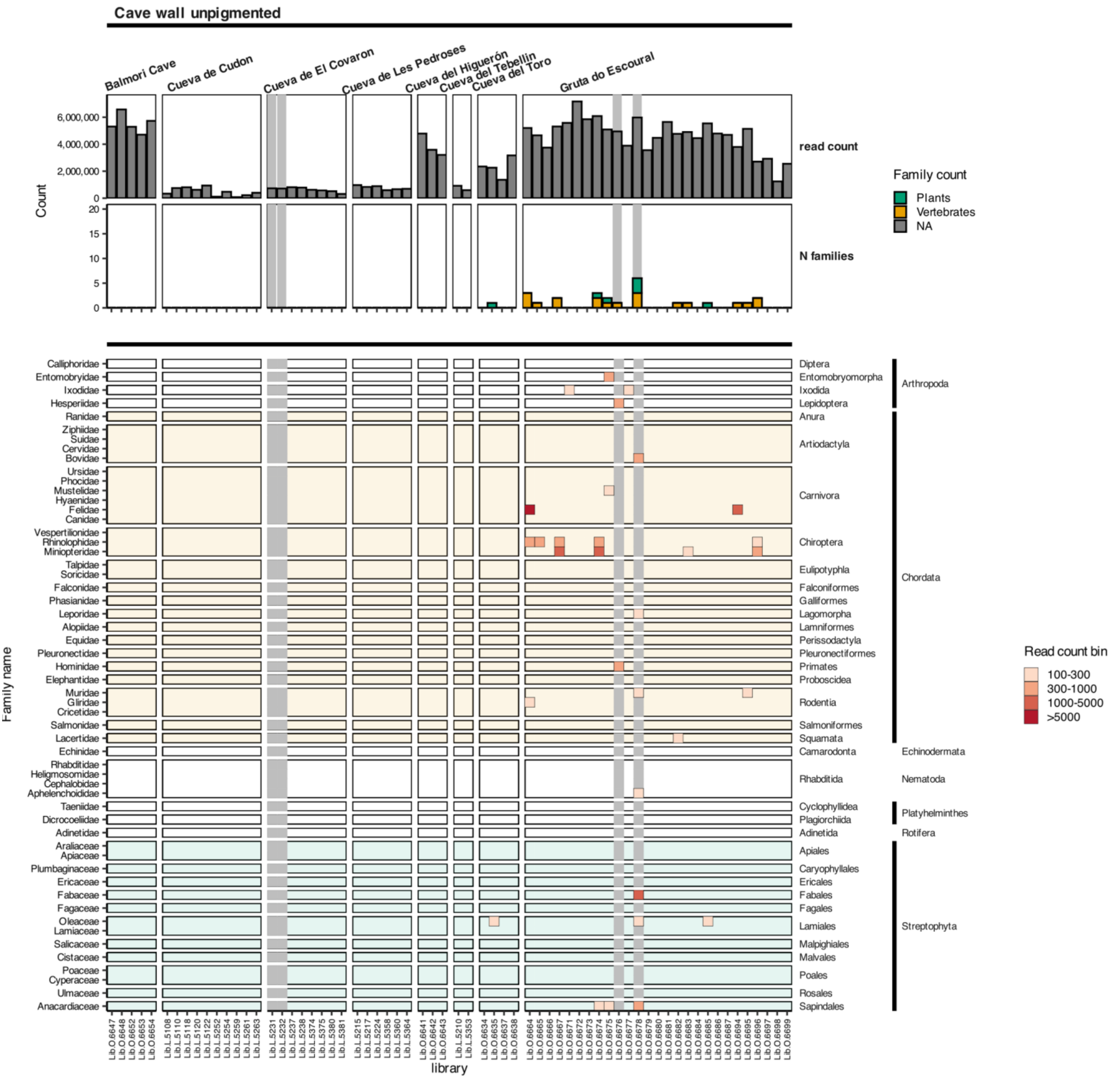

Fig. S20: Metagenomic analysis of shotgun data obtained from the unpigmented cave wall samples.

Source data are provided as a Source Data file.

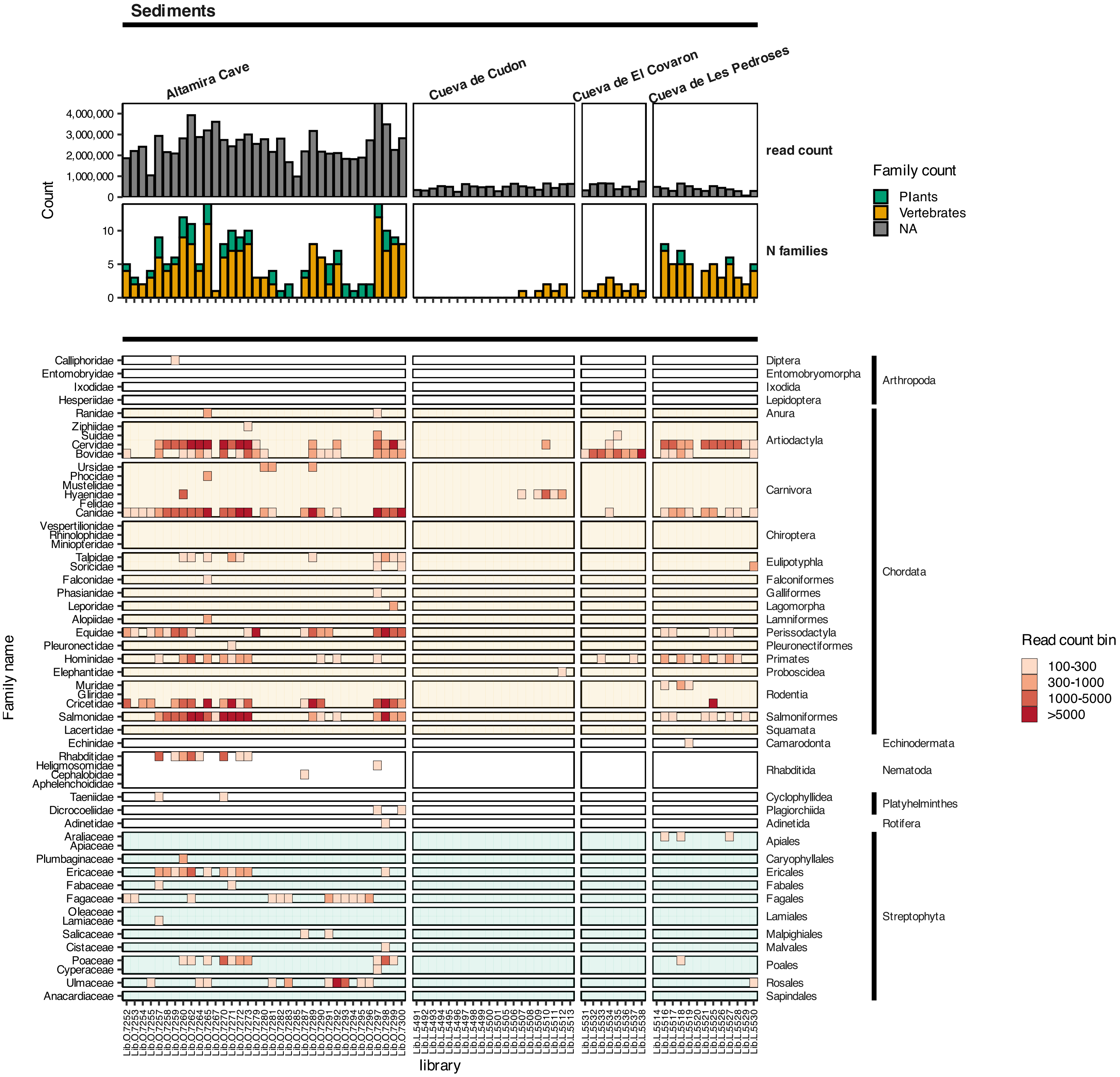

Fig. S21: Metagenomic analysis of shotgun data obtained from the sediment samples. Source data are provided as a Source Data file.

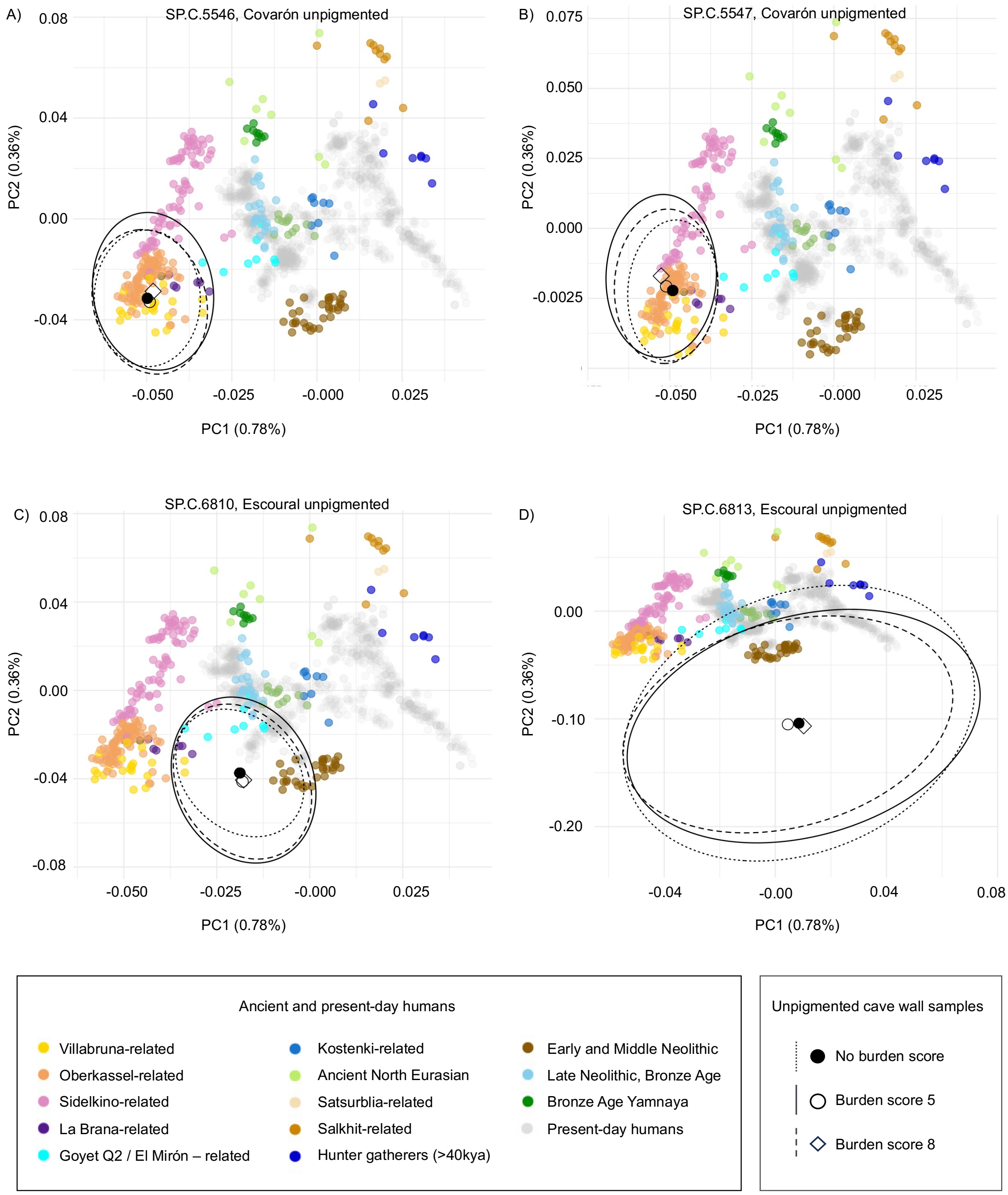

Fig. S22: Principal component (PC) analysis of Eurasian modern human genomes (grey) with ancient human genomes (coloured) and cave wall samples (black) projected on top. Each plot represents a different unpigmented cave wall sample with their respective 95% CI ellipsoid: A) SP.C.5546, B) SP.C.5547, C) SP.C.6810 and D) SP.C.6813. Target positions in the nuclear genome were filtered by mammalian sequence divergence ('burden score') to minimize faunal contamination in the analysis. Source data are provided as a Source Data file.

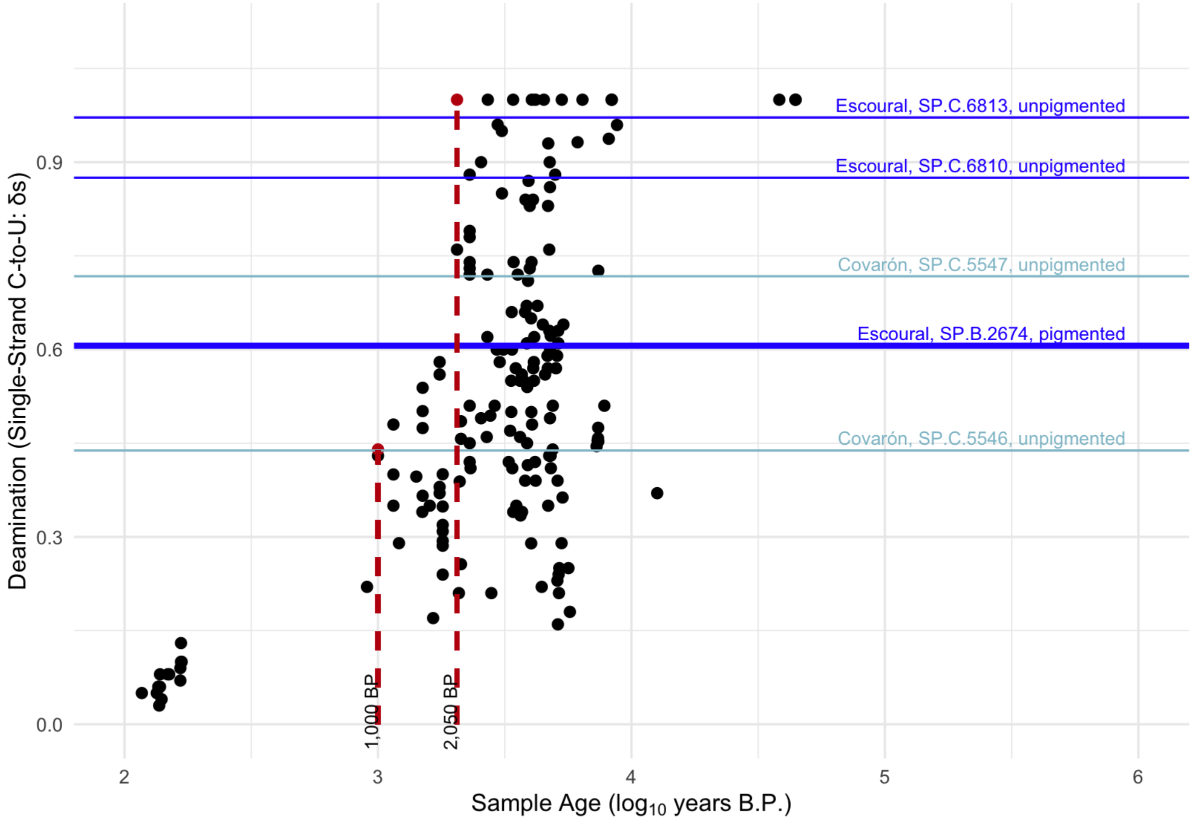

Fig. S23: Comparison of inferred deamination rates in single-stranded DNA overhangs between the cave wall samples containing ancient human DNA (coloured by caves) and a dataset of 185 published ancient DNA samples. Deamination in single-stranded overhangs was calculated using mapDamage 2.0 in "--single-stranded" mode to account for single-stranded library preparation. Permafrost samples were excluded from the analysis. Red lines indicate the youngest samples showing the same or higher deamination rates as the cave wall samples analysed in this study. These samples are dated to 1,000 and 2,050 years BP, respectively. Source data are provided as a Source Data file.

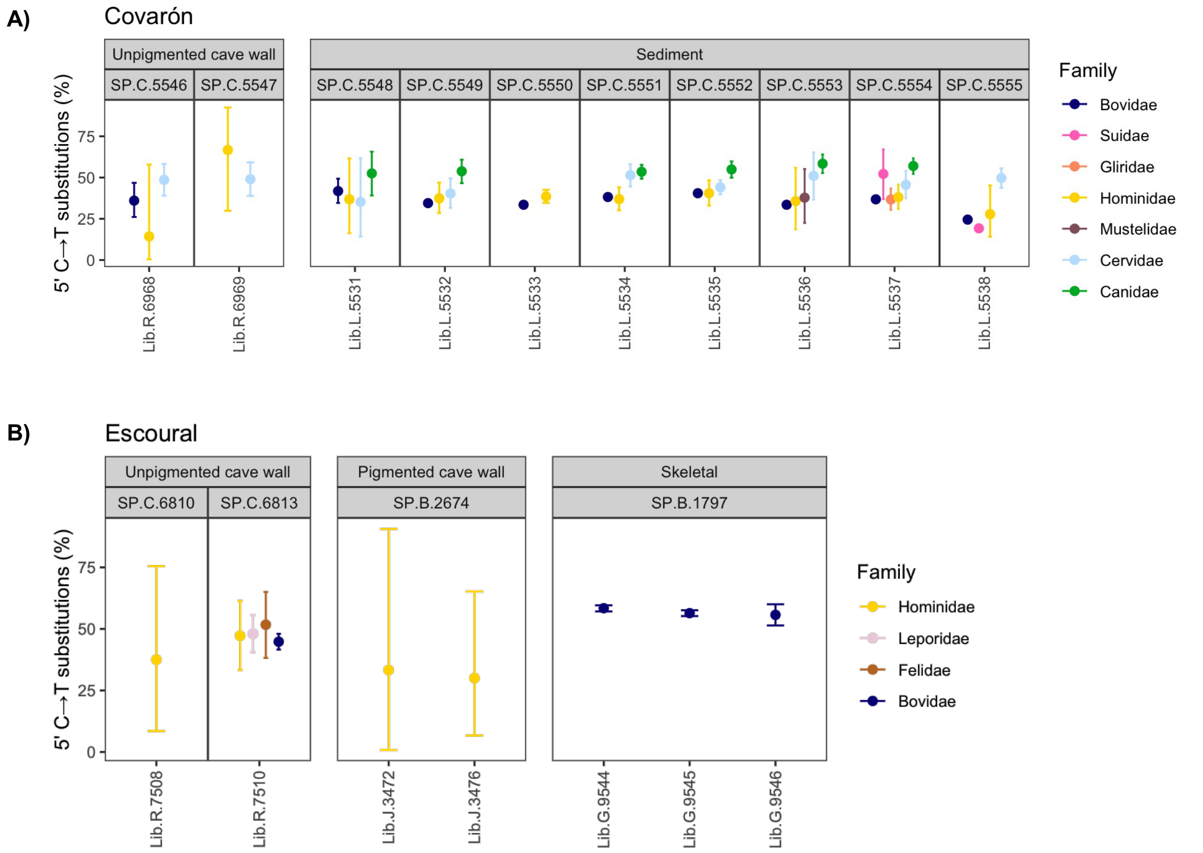

Fig. S24: Comparison of deamination rates (5' end only) between cave wall samples, sediments and bones using mtDNA fragments of all identified ancient families. Samples are shown grouped by site: A) Covarón and B) Escoural. To mitigate the effect of present-day human contamination, conditional substitution frequencies were plotted for the sequences assigned to Hominidae. For all other families, all sequences were used. Whiskers indicate 95% binomial confidence intervals. Source data are provided as a Source Data file.

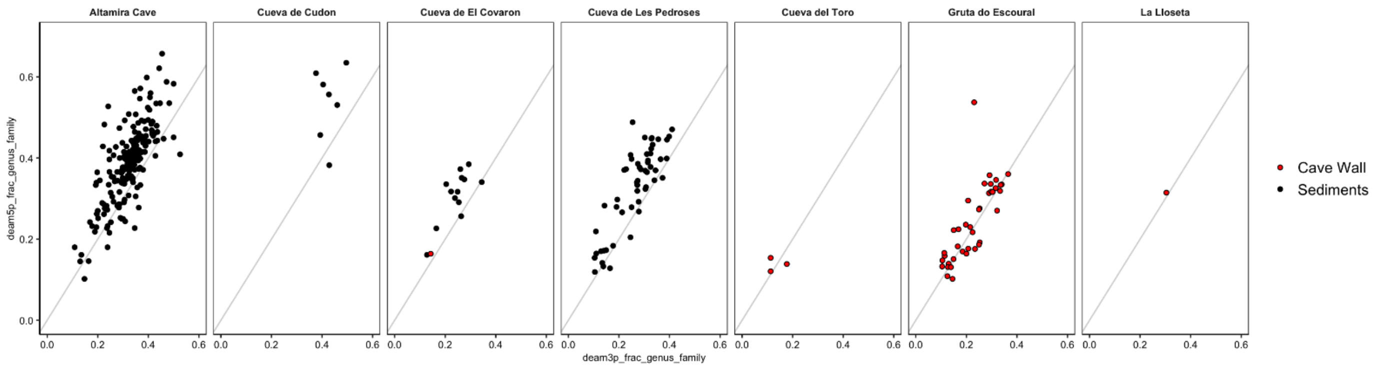

Fig. S25: Comparison of deamination rates (5' and 3' ends of molecules) between cave wall and sediment samples using shotgun data. Each dot represents sequences assigned to one mammalian family in one sample. Source data are provided as a Source Data file.

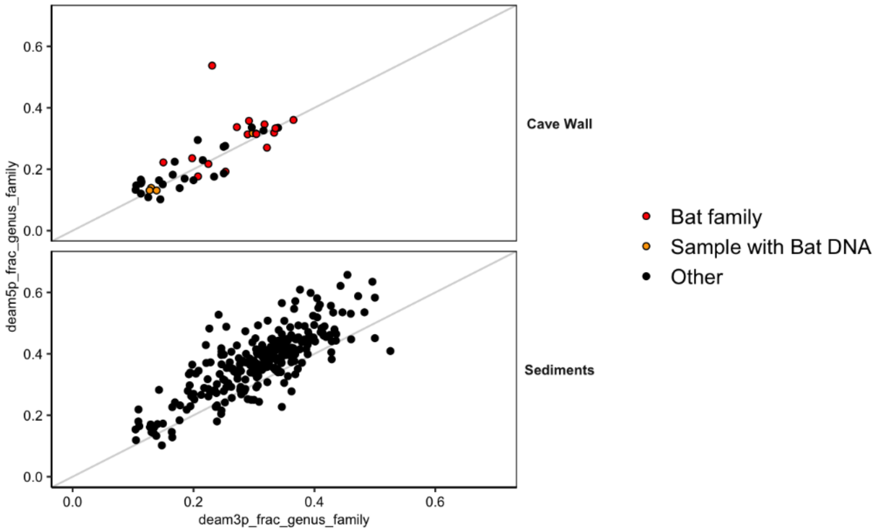

Fig. S26: Comparison of deamination rates (5’ and 3’ ends of molecules) between cave wall and sediment samples using shotgun data, stratified by the presence or absence of bat DNA in the same sample. This analysis was performed to determine whether bat faeces (thought to be the main contributor of bat DNA) facilitate DNA degradation. Each dot represents sequences assigned to one mammalian family in one sample. Source data are provided as a Source Data file.

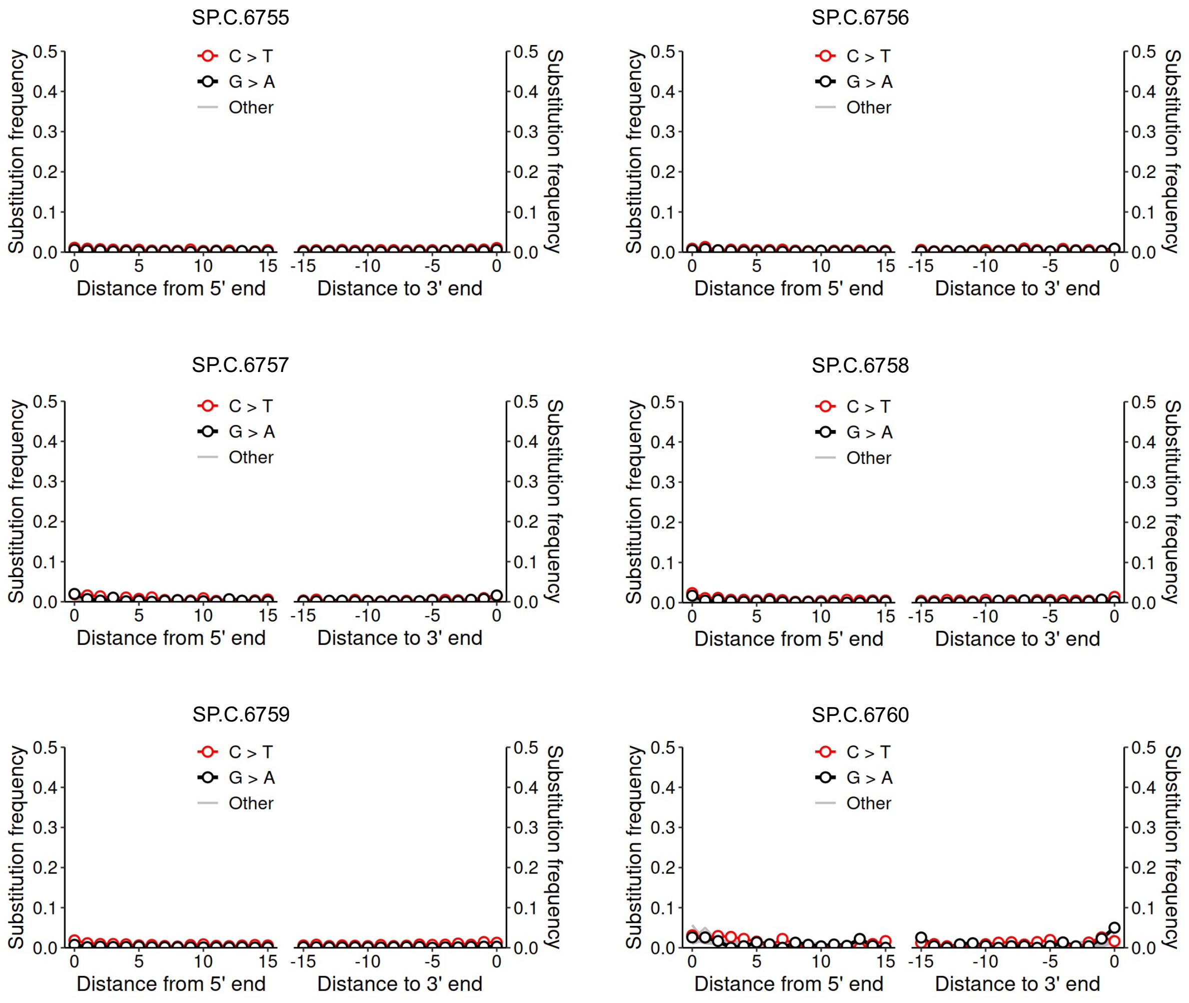

Fig. S27: Terminal C-to-T substitution frequencies of the subsamples taken from the Airbrush from Altamira). On the left: C-to-T substitution frequencies as directly observed; on the right: conditional C-to-T substitution frequencies in the merged data from both libraries. Source data are provided as a Source Data file.

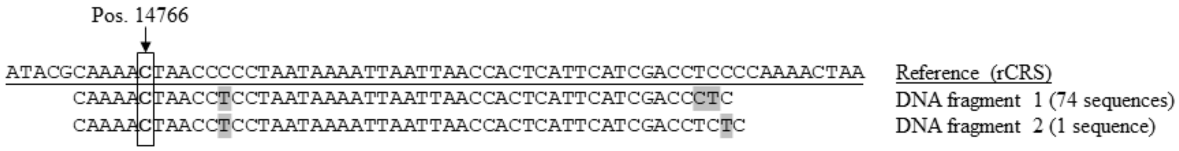

Fig. S28: Sequences overlapping the defining position for mitochondrial haplogroup HV in the pigmented sample from Escoural (SP.B.2674). The number of observed sequence reads prior to deduplication is shown on the right, suggesting that DNA fragments 1 represents a PCR artefact. Differences relative to the reference genome are highlighted in gray. The two substitutions observed near the 3’ end of DNA fragment 1, one of which leads to its classification as deaminated, could be eliminated by the insertion of a single cytosine.

Supplementary References

1. Vohr, S. H. *et al.* A phylogenetic approach for haplotype analysis of sequence data from complex mitochondrial mixtures. *Forensic Sci. Int. Genet.* **30**, 93–105 (2017).
2. Van Oven, M. PhyloTree Build 17: Growing the human mitochondrial DNA tree. *Forensic Sci. Int. Genet. Suppl. Ser.* **5**, e392–e394 (2015).
3. Behar, D. M. *et al.* A “Copernican” Reassessment of the Human Mitochondrial DNA Tree from its Root. *Am. J. Hum. Genet.* **90**, 675–684 (2012).
4. Li, H. & Durbin, R. Fast and accurate short read alignment with Burrows–Wheeler transform. *Bioinformatics* **25**, 1754–1760 (2009).
5. Zhao, H., Shen, J., Medico, L., Platek, M. & Ambrosone, C. B. Length heteroplasmies in human mitochondrial DNA control regions and breast cancer risk. *Int J Mol Epidemiol Genet* **1**, 184–192 (2010).
6. Quinlan, A. R. & Hall, I. M. BEDTools: a flexible suite of utilities for comparing genomic features. *Bioinformatics* **26**, 841–842 (2010).
7. Meyer, M. *et al.* A mitochondrial genome sequence of a hominin from Sima de los Huesos. *Nature* **505**, 403–406 (2014).
