# Supplementary material for: Investigating ancient human DNA preservation on cave walls and in rock art
Source: Nat Commun. 2026 Jun 23;17:5561. doi: 10.1038/s41467-026-74234-2 (PMC13291361; doi:10.1038/s41467-026-74234-2)
Supplement: Supplementary file 2 — Description of Additional Supplementary Files [file 41467_2026_74234_MOESM2_ESM.pdf]

Description of Additional Supplementary Files

- **Supplementary data 1:**
  Description: Overview of the samples analysed in this study, together with the lysates, DNA extracts, libraries, captured libraries and sequence data generated.

- **Supplementary data 2:**
  Description: Overview of the mammalian mtDNA captures performed, family-level taxonomic assignments of sequences using 'quicksand', and evaluation of ancient DNA damage patterns.

- **Supplementary data 3:**
  Description: Overview of the human mtDNA captures performed, identification of hominid sequences using 'quicksand', evaluation of ancient DNA damage patterns and assignment of hominin sequences to different branches in the hominin mtDNA tree.

- **Supplementary data 4:**
  Description: Overview of the shotgun sequence data generated and taxonomic assignments obtained.

- **Supplementary data 5:**
  Description: Overview of the human nuclear DNA captures performed, sequencing summary statistics, number of deaminated fragments recovered, and filtering of the data using Kraken.

- **Supplementary data 6:**
  Description: Overview of the assemblies used as reference for the shotgun sequence data analysis.

- **Supplementary data 7:**
  Description: Overview of the haplogroup estimate for relevant cave wall samples.
