# Supplementary material for: Investigating ancient human DNA preservation on cave walls and in rock art
Source: Nat Commun. 2026 Jun 23;17:5561. doi: 10.1038/s41467-026-74234-2 (PMC13291361; doi:10.1038/s41467-026-74234-2)
Supplement: Supplementary file 10 — Reporting Summary [file 41467_2026_74234_MOESM10_ESM.pdf]

nature portfolio

Corresponding author(s): Bossoms Mesa, Alba
Meyer, Matthias
Collado Giraldo, Hipólito

Last updated by author(s): Feb 27, 2026

Reporting Summary

Nature Portfolio wishes to improve the reproducibility of the work that we publish. This form provides structure for consistency and transparency in reporting. For further information on Nature Portfolio policies, see our Editorial Policies and the Editorial Policy Checklist.

Statistics

For all statistical analyses, confirm that the following items are present in the figure legend, table legend, main text, or Methods section.

n/a	Confirmed	
☐	☒	The exact sample size (*n*) for each experimental group/condition, given as a discrete number and unit of measurement
☐	☒	A statement on whether measurements were taken from distinct samples or whether the same sample was measured repeatedly
☒	☐	The statistical test(s) used AND whether they are one- or two-sided<br>*Only common tests should be described solely by name; describe more complex techniques in the Methods section.*
☒	☐	A description of all covariates tested
☒	☐	A description of any assumptions or corrections, such as tests of normality and adjustment for multiple comparisons
☐	☒	A full description of the statistical parameters including central tendency (e.g. means) or other basic estimates (e.g. regression coefficient) AND variation (e.g. standard deviation) or associated estimates of uncertainty (e.g. confidence intervals)
☒	☐	For null hypothesis testing, the test statistic (e.g. *F*, *t*, *r*) with confidence intervals, effect sizes, degrees of freedom and *P* value noted<br>*Give P values as exact values whenever suitable.*
☒	☐	For Bayesian analysis, information on the choice of priors and Markov chain Monte Carlo settings
☒	☐	For hierarchical and complex designs, identification of the appropriate level for tests and full reporting of outcomes
☒	☐	Estimates of effect sizes (e.g. Cohen's *d*, Pearson's *r*), indicating how they were calculated

Our web collection on statistics for biologists contains articles on many of the points above.

Software and code

Policy information about availability of computer code

Data collection	No software was used for data collection.
Data analysis	All software packages used for analysis are cited in the Methods section and in the Supplementary Information and all used packages are publicly available:<br><br>All sequence data processing included adapter trimming and read merging using leeHom (https://bioinf.eva.mpg.de/leehom/), BWA (version 0.5.10-evan.9-1-g44db244, https://github.com/mpieva/network-aware-bwa), bam-rmdup (https://github.com/mpieva/biohazard-tools/).<br><br>The computational pipelines used for metagenomic analyses in this study are available at https://github.com/Kevinnota/NED-flow, https://github.com/aurore-bit/SediQuest and https://github.com/mpieva/quicksand<br><br>For nuclear DNA analysis: samtools (https://github.com/samtools, version 1.3.1-21) and AuthentiCT (version 1.0.1, https://github.com/StephanePeyregne/AuthentiCT).

For manuscripts utilizing custom algorithms or software that are central to the research but not yet described in published literature, software must be made available to editors and reviewers. We strongly encourage code deposition in a community repository (e.g. GitHub). See the Nature Portfolio guidelines for submitting code & software for further information.

Data

Policy information about availability of data

All manuscripts must include a data availability statement. This statement should provide the following information, where applicable:

- Accession codes, unique identifiers, or web links for publicly available datasets
- A description of any restrictions on data availability
- For clinical datasets or third party data, please ensure that the statement adheres to our policy

The genetic data from this study have been deposited in the European Nucleotide Archive under accession number PRJEB97209.

Research involving human participants, their data, or biological material

Policy information about studies with human participants or human data. See also policy information about sex, gender (identity/presentation), and sexual orientation and race, ethnicity and racism.

Reporting on sex and gender	The biological sex of the ancient human individuals whose DNA was found in the cave wall samples was determined based on the nuclear DNA sequence data, by comparing sequence coverage of the autosomes and sex chromosomes.
Reporting on race, ethnicity, or other socially relevant groupings	N/A.
Population characteristics	N/A.
Recruitment	N/A.
Ethics oversight	N/A.

Note that full information on the approval of the study protocol must also be provided in the manuscript.

Field-specific reporting

Please select the one below that is the best fit for your research. If you are not sure, read the appropriate sections before making your selection.

☒ Life sciences ☐ Behavioural & social sciences ☐ Ecological, evolutionary & environmental sciences

For a reference copy of the document with all sections, see nature.com/documents/nr-reporting-summary-flat.pdf

Life sciences study design

All studies must disclose on these points even when the disclosure is negative.

Sample size

The sampling of rock art presents unique challenges that differ fundamentally from other types of material. As illustrated in Figure 2, pigment occurs in highly variable contexts: directly on the wall, beneath or within carbonate protuberances, under soft mineral layers, in fissures, as flakes that have fallen to the ground, etc. These differences, combined with conservation concerns, required us to select sampling strategies on a case-by-case basis to minimize the visual and structural impact of the sampling. Furthermore, restrictions on the amount and location of material that could be removed differed between sites for both the pigmented samples and the unpigmented controls, due to conservation regulations and site management.

As a result, we analysed DNA preservation in pigment samples collected in and around 24 rock art panels from 11 caves across Spain and Portugal. This included 45 pigmented cave wall samples, 52 unpigmented cave wall samples, 9 mixed cave wall samples (with possibility of subsampling pigmented and unpigmented portions independently); 77 sediment samples from archaeological profiles, 7 sediment samples from pigment-preparation areas, 10 skeletal remains and 1 airbrush made of bird bone.

The number of human libraries analysed in this study was further limited by identifying those specimens that had sufficient levels of ancient DNA preservation for downstream sequencing and analysis. For cave wall samples enriched for human mitochondrial DNA, this included 4 unpigmented cave wall samples and 1 pigmented cave wall sample. All of this is illustrated in Supplementary Tables Tables 1-5, and Figure 1.

Data exclusions

We used pre-established criteria in ancient DNA research for authentication: C-to-T substitution frequencies significantly exceeding 10% at both molecule ends - determined using binomial 95% confidence intervals - were considered indicative of ancient DNA. For human mtDNA analysis of non-sediment samples, the deamination threshold was relaxed to maximize sensitivity, requiring a significant enrichment of C to T substitution (>10%, as described above) at only one end of the DNA molecules, rather than both.

Moreover, for mitochondrial captures, since metagenomic analyses are prone to taxonomic misidentification - especially when only few sequences are assigned to a specific taxon - we verified the plausibility of all taxa showing evidence for ancient DNA based on geographic distribution. This further analysis removed a small number of taxa (Cebidae, Cercopithecidae and Hylobatidae), all of which were represented by < 20 DNA sequences.

	Sequence data from libraries enriched for human nuclear DNA were analysed following pre-established criteria in ancient DNA research, excluding sequences form the sequencing data that did not map to the human genome, sequences that were shorter than 35 base pairs and sequences mapping with a low mapping quality (<25); all of which are excluded to avoid incorporating sequences that are not endogenous to the individual(s) sequenced.
Replication	When possible, we took multiple samples from the same specimen to generate independent single-stranded DNA libraries. The results of reproducibility of the data generation and analyses are reported across the Supplementary Data Tables 1-5. To allow reproducibility of the downstream analyses, all filtering steps and the comparative data used in this study are detailed in the Methods and the Supplementary Information. Moreover, all of the data obtained in this study can be downloaded from the repositories indicated in the "Data availability" statement.
Randomization	Randomization is not relevant to this study. We first determined DNA preservation in all ancient specimens described in the "Sample size" section above, and then proceeded to analyse generated genome-wide data for all specimens which showed evidence of endogenous DNA preservation.
Blinding	Blinding was not relevant as we sampled ancient hominin specimens that were selected for this study based on their archaeological context (i.e. age and provenance), thus blinding would be inappropriate given the scarcity and the value of the sampled material. Blinding in downstream analyses was not relevant given that we analysed genome-wide data of the specimens in relation to publicly available datasets of present-day and ancient human genomes.

Reporting for specific materials, systems and methods

We require information from authors about some types of materials, experimental systems and methods used in many studies. Here, indicate whether each material, system or method listed is relevant to your study. If you are not sure if a list item applies to your research, read the appropriate section before selecting a response.

Materials & experimental systems

n/a	Involved in the study
☒	☐ Antibodies
☒	☐ Eukaryotic cell lines
☐	☒ Palaeontology and archaeology
☒	☐ Animals and other organisms
☒	☐ Clinical data
☒	☐ Dual use research of concern
☒	☐ Plants

Methods

n/a	Involved in the study
☒	☐ ChIP-seq
☒	☐ Flow cytometry
☒	☐ MRI-based neuroimaging

Palaeontology and Archaeology

Specimen provenance	The sampling activities presented in this article were carried out within the framework of the project FIRST ART (Ref. No. 0497_FIRST_ART_4_E), funded under the Interreg V-A Spain– Portugal (POCTEP) cross-border cooperation programme and coordinated by the Government of Extremadura. A more detailed information about the permits for each specimen is detailed in "Supplementary Information 12 – Revelant permits regarding sampled caves and archeological objects".
Specimen deposition	All specimens (bones, sediments and cave wall samples) are deposited in the Max Planck Institute for Evolutionary Anthropology, with the exception of the Altamira Airbrush, that never left the Museum of Prehistory and Archaeology of Cantabria . Scientists wanting to conduct research on these specimens should obtain permits from the institutions housing the specimens.
Dating methods	No new archaeological dates are provided in this study.

☐ Tick this box to confirm that the raw and calibrated dates are available in the paper or in Supplementary Information.

Ethics oversight	All approvals for specimen sampling have been obtained from the relevant institutions.

Note that full information on the approval of the study protocol must also be provided in the manuscript.

Plants

Seed stocks	N/A.
Novel plant genotypes	N/A.
Authentication	N/A.
