# Supplementary material for: Investigating ancient human DNA preservation on cave walls and in rock art
Source: Nat Commun. 2026 Jun 23;17:5561. doi: 10.1038/s41467-026-74234-2 (PMC13291361; doi:10.1038/s41467-026-74234-2)
Supplement: Supplementary file 11 — Transparent Peer Review file [file 41467_2026_74234_MOESM11_ESM.pdf]

nature portfolio

Peer Review File

Investigating ancient human DNA preservation on cave walls and in rock art

Corresponding Author: Dr Matthias Meyer

This file contains all reviewer reports in order by version, followed by all author rebuttals in order by version.

Version 0:

Reviewer comments:

Reviewer #1

(Remarks to the Author)
The work is presented as a technically sound study establishing that ancient human DNA can persist on cave walls, which is, in itself, an important finding. However, the limitations in linking this DNA to the creation of art may be more substantial than the work attempts to convey.
Overall, the manuscript represents an important methodological advance in paleogenetics, being the first systematic study to investigate DNA preservation in rock art using next-generation sequencing techniques.
Undoubtedly, the study represents a proposal that points the way toward a novel and necessary field of study, doing so with relatively broad sampling (11 caves, 24 panels) employing technically advanced data collection and processing systems, as well as anti-contamination protocols and methodological controls (see non-pigmented adjacent samples, sediments, etc.) and complementary metagenomic analysis that underscore the comprehensive nature of the study. Added to this is the analytical rigor with different authentication lines for ancient DNA, estimation of faunal contamination, etc., all while also providing contamination data and rates, which emphasizes the transparency of the process.
The robustness of the method is further certified by complementary data (chemical, chronological, archaeological context, etc.).
Nevertheless, it presents substantial limitations in its conclusions that may require more attention. In any case, on the other hand, this observation is inherent to any study that represents a true novelty, and this one certainly is.
Regarding conclusions about the authorship of the art, these appear overestimated and not fully supported. In this respect, for the most conclusive sample in the work (SP.B.2674, at Escoural), the authors' consideration that it "may be directly linked to its application on cave walls" does not appear absolutely conclusive. The only temporal evidence is that Escoural was sealed until the 4th millennium BC (cal. BCE), but this provides only a very broad terminus ante quem (>6,000 years).
Likewise, the authors themselves acknowledge that some other samples (non-pigmented) could point to the contribution of "other forms of human contact, unrelated to artistic production." However, they do not systematically explore these alternatives: post-creation ritual/ceremonial contact; torch maintenance activities; simple casual contact during occupations at different times; biofilms/microbial communities incorporating degraded environmental DNA.
It should not be forgotten that the success rate in the analysis is extremely low (1/24 = 4.2%), so that only one panel with art (out of 24) yielded human DNA, something that runs counter to general expectations if the DNA systematically came from the process of creating these artistic manifestations. The authors attribute this to degradation but do not consider that in sediments from the same site (Covarón) >26,000 human mtDNA fragments were recovered; or that the differential preservation suggests that rock art per se is not a systematic source of DNA from its creators. Although this would certainly be desirable.
One aspect that perhaps should be given more consideration could be possible modern contamination, an aspect that is somewhat underestimated. In a transparency exercise by the study's authors, the contamination rates in the samples (32.4-54.8% in SP.B.2674, the best sample with human DNA reported) are extremely high. The conditional substitution test assumes that molecules without deamination are all modern contaminants, but recent ancient molecules (<5,000 years) may have low deamination (see Kistler et al., 2017, PNAS 114:7099-7104), or there could even be the possibility that mixing ancient DNA from multiple time periods could create complex patterns.
Along these lines, the authors seem to conclude that three samples with co-occurrence of human-faunal DNA (SP.C.5546, SP.C.5547, SP.C.6813) represent "contamination with soil sediments." Regarding sex determination, the three "contaminated" samples are predominantly female, which is an interesting finding but should be statistically analyzed, given that one study (Vernot et al., 2021, Science 372:eabf1667) establishes this would be improbable if the source were a random mixture of sediments, since these show balanced sex ratios.

Likewise, an alternative interpretation could be inferred based on gender-specific activities. In this regard, ethnographic/archaeological studies (Dobres, 2000, Technology and Social Agency; Conkey and Hastorf, 1990, The Uses of Style in Archaeology; Zubieta, 2016, Jour. Anthr. Archae. 43...) document sexual division of labor in pigment processing.
Perhaps a certain contradiction (or need for clarification) is evident regarding the interpretation of fauna. The authors argue that the presence of felids, leporids, and herbivores indicates sediments, but the identified species (bovids, cervids) were also used as pigment sources (bone marrow), binders (fat), and tools (bones).
Perhaps the metagenomic analysis is underexplored. The shotgun analysis reveals bat predominance, but the authors do not discuss possible implications for DNA preservation (bats defecate/urinate on walls, creating microenvironments that could accelerate DNA degradation...). The microbial context is not explored in depth either.
To give more substance to the methodology employed, perhaps a parallel experimental analysis could be provided to validate (through simulated deposition experimentation) how much DNA is deposited when touching, spitting, painting, etc. on walls? These data could serve as a framework to calibrate expectations.
Regarding temporality, there may be some limitation that should be considered and qualified in the conclusion. The authors claim that the DNA is "several thousand years old" based only on pronounced degradation (deamination) and the sealing of Escoural until ±6,000 BP. The problem could lie in the fact that DNA degradation could be extremely variable depending on the microenvironment, and this should be specifically considered in the case study. Perhaps by applying DNA degradation modeling (thermal age modeling; Smith et al., 2003, Proc. R. Soc. B 270:939-945) and/or integrating empirically measured cave temperature/humidity.
Perhaps some works of similar themes could be reviewed/referenced:
Baales et al. 2017. Quat. Int. on DNA recovery from lithic tools. Directly relevant for discussing methodological alternatives.
Willerslev et al. 2003. Science 300:791-795 on environmental DNA in sediments. Pioneering in showing that DNA can persist without tissues, but emphasizes that the source is ambiguous (critical for authorship interpretations).

(Remarks on code availability)

Reviewer #2

(Remarks to the Author)
This manuscript explores whether Palaeolithic rock art surfaces can preserve ancient DNA, extending genetic inquiry beyond traditional archaeological sediments and artefacts. By analysing samples from rock art panels across 11 Iberian caves, the authors show that ancient DNA can indeed persist on cave walls, both in pigmented areas and in unpigmented sections. At Escoural Cave, human-only DNA recovered from a pigmented calcite crust and an adjacent unpigmented wall sample is interpreted as reflecting direct human contact, while other unpigmented samples containing mixed human-faunal DNA are interpreted as reflecting indirect deposition. Although establishing a direct link between DNA and the creators of the art remains challenging, this study provides compelling evidence for the potential of cave wall surfaces to retain ancient genetic material. We found this an innovative and well-crafted study and recommend publication. However, we do have some general comments that could help strengthen the archaeological integration and interpretive clarity of the manuscript.

1. Integration with archaeological data: The manuscript would benefit from a fuller integration of the archaeological context of the sampled sedimentary layers, art panels and sites. Although the methodology is very attractive, the fact that DNA seems to be recorded in cave walls independently of art raises the broader question of: What did we learn from this study in terms of cave art and associated/nearby archaeology?
Independently of the positive or negative results, however, the manuscript would gain by incorporating more detailed information on the stratigraphic setting of sampled archaeological sediments, age estimates for both the artworks (radiometric, when available, but at least stylistic correlations) and sedimentary layers, as well as archaeological content (if any) of the sedimentary layers sampled (in the SI if not in the main manuscript).
This is particularly important for the samples from Covarón, where ancient hominin DNA has been recovered and where the genetic dating spans a very broad range (~16,700–5,200 cal BP) (Lines 275-276) –is there some data on the stylistic affinity of the cave art that could help further constrain this period?

2. Issues of association between sampled materials and the paintings:
While in this study the interpretive consequences are limited because (correctly) no strong associations are claimed, it is important to acknowledge that both unpigmented cave walls, calcite layers overlying pigments and the sampled sediments may have substantially different ages than the paintings (see, for instance, Hoffmann et al. 2018, Science). These temporal offsets should be explicitly addressed, as it has implications for interpreting any recovered aDNA. In other words, if DNA is just randomly preserved in cave walls, how should one go about interpreting it if, in the future, a positive (and potentially unexpected) result is found in association with a pigmented area?

3. Sampling strategy: Building up on this, there is the issue of how to sample these challenging contexts. Though I acknowledge that important preservation constrains exist when sampling the rare and unique rock art panels and sampling strategies may vary from site-to-site, it was nonetheless unclear to me what was the sampling methodology and why this strategy was not made more standardized across different sites and/or panels within sites. If possible, would it not be relevant, for instance, to sample consistently from the calcite above the pigment (when present), the pigmented areas, below the pigmented areas and then sampling laterally adjacent and away from the paintings (see examples of this in Hoffmann et al 2016 Quaternary Geochronology)? Right now, each site has substantial different sampling protocols that are in no way standardized.

4. Interpretation of wall-sample positivity as contamination from cave-floor sediments:

The manuscript currently states that positive samples with co-occurrence of faunal and human DNA from the walls may reflect contamination originating from cave-floor sediments (e.g., Lines 227-228, 231-232, 323-325). However, the mechanisms by which sediments from the cave floor below could contaminate the walls located stratigraphically above are unclear and left unexplained. Would not alternative pathways – such as water percolation, dissolution and transport of DNA through walls and ceilings, infiltration from overlying soils, or genetic traces left by later cave occupants – better explain these results? Similar processes have been proposed to account for DNA found in speleothems where no sediment floor contamination is expected – and only mentioned in Lines 326-327. I would urge the authors to address these mechanisms of sources and transfers of DNA in caves, and consider citing work such as Brown et al. 2025, JAS that discuss just this.

Overall, these considerations raise broader questions: to what extent might any DNA detected on cave walls be influenced by such processes, and how can future studies robustly associate hominin DNA from pigmented areas with the individuals responsible for creating the art? Expanding the discussion of these issues would significantly strengthen the manuscript.

Other comments

- Although the extraction method proposed by the authors was tested on DNA-spiked clay and lime, can the author expand on how this extraction protocol works on "real" sediment where the binding mechanisms of DNA in soils/sediments is more complex than in DNA-spiked minerals? Also, did they see any effect of the initial mass of the sample affecting DNA extraction efficiency (e.g., Capo et al., 2021)?
- Was the cave wall surface routinely cleaned or mechanically removed prior to collecting samples for aDNA analysis?
- Fig.4: Consider using distinct colors in the top histogram to differentiate the sites and improve readability.
- It was encouraging to see that two hand stencils, that involve direct hand contacts to the cave walls, were sampled at Maltravieso, even though they did not yield aDNA, consistent with the lack of results from skeletal material at the site. Were additional hand stencils available for sampling, and if so, what was the rationale for not selecting them more extensively?
- Please specify "flakes" (e.g., Lines 146-147) - do you mean flakes of limestone?

(Remarks on code availability)

Reviewer #3

(Remarks to the Author)
I co-reviewed this manuscript with one of the reviewers who provided the listed reports. This is part of the Nature Communications initiative to facilitate training in peer review and to provide appropriate recognition for Early Career Researchers who co-review manuscripts.

(Remarks on code availability)

Reviewer #4

(Remarks to the Author)
The manuscript "Recovering ancient human DNA from rock art and cave walls" addresses a question that many, primarily archaeologists, have been asking for decades: is it possible to detect genetic traces of the individuals who created rock art within the pigments they used? The study is meticulously planned, and the analytical procedures are clearly described and carried out to the highest standards of the ancient DNA field.

To test whether human DNA can be recovered from rock art, the authors designed an appropriate and comprehensive sampling and analytical strategy. However, despite this well-executed approach, the results show that more human DNA was obtained from sites or samples without pigmentation than from the pigment itself. This suggests that the answer to the central question is, unfortunately, negative: we currently cannot recover meaningful human aDNA directly from rock art pigments. This raises a challenge, this is a well, designed and valuable study, but its primary outcome is a negative result. How should this be contextualized and interpreted?

I have some comments:
The title is somewhat misleading. The study does not recover ancient human DNA from the rock art itself, but from areas adjacent to it. This distinction should be made clearer.

The human DNA identified cannot be securely dated. While the damage patterns are consistent with ancient DNA, this alone is not sufficient evidence for chronological attribution in this context. A discussion of this limitation would strengthen the manuscript.

It would be beneficial to include a discussion of the preservation potential of DNA in rock art and cave wall matrices. What minerals or substrates are present? Sedimentary aDNA studies often address this to help readers understand why DNA is or isn't preserved, and a similar treatment here would improve interpretability.

Methodological concern (lines 478–479)
The manuscript notes that some samples were embedded in resin and hardened at 60°C for 2–7 days. This seems like potentially harsh treatment for ancient DNA. A clarification of how this process affects DNA integrity, or why it does not,

would be valuable.

Figure 3
This figure is confusing. The caption states that it shows “before and after” sampling, but most of the images appear to show only the post-sampling state. Either the figure or the caption should be clarified.

Figure 4
In the top panel of Figure 4, the meaning of the colour black is unclear. Additional explanation in the caption or legend is needed.

(Remarks on code availability)

Reviewer #5

(Remarks to the Author)
After all the previous success stories of obtaining authentic DNA not only from bones of archaic and modern humans from different, including early times, but more recently also from sediments and artifacts, an aDNA study on rock art was long awaited, and with this study is finally delivered in highest quality. While identifying the artist via aDNA analysis is impossible, and not expected in such scenario because DNA-based identification works in a strictly comparative way and always requires a reference sample of known identity, this is the first study demonstrating that aDNA, human and non-human, can be obtained from rock art. Moreover, detecting variation in detecting human DNA with and without animal DNA may suggest differences in ancient painting techniques. Overall, this is a long-awaited, well-executed, comprehensive study that includes samples from different caves and employs the appropriate study design with well-controlled sampling from pigmented and neighbouring unpigmented areas, additionally including sediment-based and bone-based aDNA analysis, and executed via state-of-the-art sample preparation and DNA analysis as well as data analyses methods. It sets the scientific and technical standards for future rock art studies at other sites. Moreover, this study provides strong motivation for authorities holding responsibility for caves with rock art to eventually allow more aDNA research on more rock art paintings in different countries. This manuscript clearly deserves publication in a highly respected scientific journal, such as this one, while personally, I would have expected this work to appear in one of the highest respected journals.

(Remarks on code availability)

Version 1:

Reviewer comments:

Reviewer #1

(Remarks to the Author)
The revised manuscript and the detailed point-by-point response provided by the authors highlights a carefully read of the comments. The reviewer thanks the effort. In general lines, the authors have made a substantial effort to address the reviewer’s concerns, both through textual revisions and also (and it is important) by incorporating additional analyses.
So, for this reviewer, the manuscript appears to have been significantly strengthened. Please, find below an assessment of the main issues took into account on the “response to referees” document.

Interpretation of the link between human DNA and the production of rock art
One of the central concerns raised in the initial review was that the manuscript may have overstated the link between the recovered human DNA and the authorship of cave art. The reviewers emphasized that the available evidence, particularly in Escoural, was insufficient to support a direct connection between the DNA signal and the act of painting.
Attending to that, the authors report having revised the abstract to explicitly state that the results do not conclusively link human DNA preservation to the creation of cave art, and indicate that the title has been modified so as not to imply such a connection. In addition to this, the “Discussion section” has been extensively revised to emphasize the limitations of the current evidence and to outline the types of data that would be required to establish a direct association between genetic material and artistic activity.
These changes represent a meaningful improvement in the interpretative caution of the original document.

Low success rate in recovering human DNA from rock art panels
On the first review there was noted that only one of the 24 sampled panels yielded human DNA, raising questions about whether rock art surfaces can be considered a systematic source of DNA from their creators. The authors acknowledge this limitation explicitly in the revised discussion, stating that pigmented surfaces rarely appear to retain detectable DNA after thousands of years. They also outline several possible explanations, including limited initial deposition, poor binding of DNA to the surface, or subsequent degradation.
These changes clarifies the methodological implications of the low recovery rate.

Modern contamination and interpretation of ancient DNA authenticity
Attending to relatively high contamination rate, the authors provide a detailed methodological response, explaining that the

strong deamination signals observed in the sequences remain detectable despite the presence of modern contamination. That was necessary to clarify that the conditional substitution test was used only to estimate deamination rates in the fraction of molecules considered ancient, and they considered that mixtures of molecules from different time periods could influence these estimates, generally leading to conservative values.
They also clarify aspects of the population genetic analyses and expand their discussion of sex determination, so they modify the manuscript to describe assignments more cautiously (e.g., “predominantly female”).

Interpretation of faunal DNA detected in the samples
At this point, the authors acknowledge that an important clarification was missing in the original manuscript: according to their metagenomic results, all non-bat faunal taxa were detected exclusively in unpigmented samples. They report that this point has now been clarified in the text. Thanks to this clarification, the text improves the transparency of the interpretation.

Potential role of bat activity in DNA degradation
The authors conducted an additional exploratory analysis comparing deamination rates across taxa in samples containing bat DNA. They state that this analysis did not reveal evidence that bat presence systematically affects DNA degradation. The authors chose not to include this analysis in the manuscript (because it could addressed a very specific hypothesis), but it could be interesting to include the analysis in some way (maybe in the supplementary material), so it might increase transparency.

Microbial context and metagenomic analysis
The authors explain that such analyses would require a different analytical framework and fall outside the scope of the present study, which focuses on plant and animal DNA potentially associated with pigment preparation.
That is right, and it sounds reasonable. Future analysis will be welcome.

Experimental calibration of DNA deposition
The authors acknowledge the value of experiments but note that they would require substantial additional work and would constitute a separate research project.
This reviewer asked about them just in case the experiments were already done. If not, such work is clearly fall beyond the scope of current paper.

Chronological interpretation of the recovered DNA
Focusing to this concern, the authors performed additional analyses comparing deamination patterns in their samples with a large dataset of ancient DNA samples compiled in previous studies, as well as they also compared damage patterns with sediment samples and archaeological bone material from relevant contexts.
Results have been incorporated into a new section of the Results, so that represent a substantial improvement to the manuscript and, according to this reviewer, provide a more robust framework for discussing the temporal dimension of the findings.

(Remarks on code availability)
Nothing to add

Reviewer #2

(Remarks to the Author)
The authors have greatly improved their manuscript. Previous comments were thoroughly addressed, and the manuscript is now ready for publication

(Remarks on code availability)

Reviewer #3

(Remarks to the Author)
I co-reviewed this manuscript with one of the reviewers who provided the listed reports. This is part of the Nature Communications initiative to facilitate training in peer review and to provide appropriate recognition for Early Career Researchers who co-review manuscripts.

(Remarks on code availability)

Reviewer #4

(Remarks to the Author)
Thank you for the opportunity to re-review this manuscript.

After carefully reading the authors’ rebuttal letter for “Investigating ancient human DNA preservation on cave walls and in rock art,” I am very pleased to see that they have taken the time to address not only my comments but also those of the other reviewers in a thorough and considered manner.

Having reviewed the revised manuscript, which is substantially improved, I have no further comments. The paper is now much clearer, and the study makes a well-articulated and meaningful contribution to the field.

(Remarks on code availability)

Open Access This Peer Review File is licensed under a Creative Commons Attribution 4.0 International License, which permits use, sharing, adaptation, distribution and reproduction in any medium or format, as long as you give appropriate credit to the original author(s) and the source, provide a link to the Creative Commons license, and indicate if changes were made.
In cases where reviewers are anonymous, credit should be given to 'Anonymous Referee' and the source.
The images or other third party material in this Peer Review File are included in the article's Creative Commons license, unless indicated otherwise in a credit line to the material. If material is not included in the article's Creative Commons license and your intended use is not permitted by statutory regulation or exceeds the permitted use, you will need to obtain permission directly from the copyright holder.
To view a copy of this license, visit https://creativecommons.org/licenses/by/4.0/

NOTE: All changes to the main manuscript text are highlighted in red to allow reviewers to easily identify the revisions.

Reviewer #1 (Remarks to the Author):

The work is presented as a technically sound study establishing that ancient human DNA can persist on cave walls, which is, in itself, an important finding. However, the limitations in linking this DNA to the creation of art may be more substantial than the work attempts to convey.
Overall, the manuscript represents an important methodological advance in paleogenetics, being the first systematic study to investigate DNA preservation in rock art using next-generation sequencing techniques.
Undoubtedly, the study represents a proposal that points the way toward a novel and necessary field of study, doing so with relatively broad sampling (11 caves, 24 panels) employing technically advanced data collection and processing systems, as well as anti-contamination protocols and methodological controls (see non-pigmented adjacent samples, sediments, etc.) and complementary metagenomic analysis that underscore the comprehensive nature of the study. Added to this is the analytical rigor with different authentication lines for ancient DNA, estimation of faunal contamination, etc., all while also providing contamination data and rates, which emphasizes the transparency of the process.
The robustness of the method is further certified by complementary data (chemical, chronological, archaeological context, etc.).
Nevertheless, it presents substantial limitations in its conclusions that may require more attention. In any case, on the other hand, this observation is inherent to any study that represents a true novelty, and this one certainly is.

We thank the reviewer for the thorough and constructive review of our manuscript. In response, we have revised the manuscript to include new analyses and to clarify the limitations of our study more explicitly. Detailed responses to each comment are provided below.

Regarding conclusions about the authorship of the art, these appear overestimated and not fully supported. In this respect, for the most conclusive sample in the work (SP.B.2674, at Escoural), the authors' consideration that it "may be directly linked to its application on cave walls" does not appear absolutely conclusive. The only temporal evidence is that Escoural was sealed until the 4th millennium BC (cal. BCE), but this provides only a very broad terminus ante quem (>6,000 years). Likewise, the authors themselves acknowledge that some other samples (non-pigmented) could point to the contribution of "other forms of human contact, unrelated to artistic production." However, they do not systematically explore these alternatives: post-creation ritual/ceremonial contact; torch maintenance activities; simple casual contact during occupations at different times; biofilms/microbial communities incorporating degraded environmental DNA.

Throughout the manuscript, we aimed to be cautious in our interpretation of the Escoural findings, explicitly noting that they do not constitute direct evidence for a link between ancient human DNA and rock art. Nevertheless, we appreciate the reviewer's comment and recognize that some of our original phrasing may have been interpreted as more suggestive than intended. In response, we have revised the abstract to read: "Although our results do not conclusively link ancient human DNA

preservation to the generation of cave art, we show that traces of human DNA can persist on cave walls for thousands of years.", and also changed the title of the paper to not suggest a direct link between the cave art and the human DNA retrieved. In the Discussion section, which we have completely revised, we have now added a sentence outlining the types of evidence that would be required to link DNA signals directly to the creation of rock art, making it very explicit that such evidence is not presented in our study.

Regarding forms of human contact other than art production that may leave DNA on cave walls, several basic modes of interaction (touching, rubbing, or deposition of bodily fluids) are already mentioned in the Discussion. These could be extended to include more specific activities as suggested by the reviewer, but we have avoided this in order to keep the text concise, as any list would become long and may still not be exhaustive. Importantly, we argue that indirect incorporation of environmental DNA (through biofilms for instance) is not a likely scenario for the deposition of human DNA, at least for the two samples that contained no mixtures with faunal DNA.

It should not be forgotten that the success rate in the analysis is extremely low (1/24 = 4.2%), so that only one panel with art (out of 24) yielded human DNA, something that runs counter to general expectations if the DNA systematically came from the process of creating these artistic manifestations. The authors attribute this to degradation but do not consider that in sediments from the same site (Covarón) >26,000 human mtDNA fragments were recovered; or that the differential preservation suggests that rock art per se is not a systematic source of DNA from its creators. Although this would certainly be desirable.

We agree that cave art does not appear to be a systematic source of human DNA, as is stated, for example, in the Discussion: "With ancient human DNA obtained from only one of 24 rock art panels sampled, despite generally favourable preservation conditions at many rock art sites, our findings suggest that pigmented surfaces themselves rarely retain enough DNA to remain detectable after thousands of years." This statement leaves open whether too little DNA was applied to the cave wall during art creation or whether DNA failed to bind to the surface or was not preserved over time.

One aspect that perhaps should be given more consideration could be possible modern contamination, an aspect that is somewhat underestimated. In a transparency exercise by the study's authors, the contamination rates in the samples (32.4-54.8% in SP.B.2674, the best sample with human DNA reported) are extremely high. The conditional substitution test assumes that molecules without deamination are all modern contaminants, but recent ancient molecules (<5,000 years) may have low deamination (see Kistler et al., 2017, PNAS 114:7099-7104), or there could even be the possibility that mixing ancient DNA from multiple time periods could create complex patterns. Along these lines, the authors seem to conclude that three samples with co-occurrence of human-faunal DNA (SP.C.5546, SP.C.5547, SP.C.6813) represent "contamination with soil sediments." Regarding sex determination, the three "contaminated" samples are predominantly female, which is an interesting finding but should be statistically analyzed, given that one study (Vernot et al., 2021, Science 372:eabf1667) establishes this would be improbable if the source were a random mixture of sediments, since these show balanced sex ratios.
Likewise, an alternative interpretation could be inferred based on gender-specific activities. In this

regard, ethnographic/archaeological studies (Dobres, 2000, Technology and Social Agency; Conkey and Hastorf, 1990, The Uses of Style in Archaeology; Zubieta, 2016, Jour. Anthr. Archae. 43...) document sexual division of labor in pigment processing.

The deamination signals in the human DNA sequences obtained from the cave wall samples are so strong that they remain detectable even in the presence of modern human contamination (see SI Figure 17, SI Table 3), providing clear evidence for the presence of ancient human DNA. The purpose of the conditional substitution test was to obtain better estimates of deamination rates in the fraction of molecules that are ancient, after depleting contamination. We agree that mixtures of younger and older ancient DNA could confound these estimates, but always in the direction of lowering them - below the actual deamination rates in the oldest fraction of molecules, by retaining sequences from the less deaminated younger molecules. They can thus be considered minimum estimates for deamination in the most degraded, or oldest, fraction of molecules.

In the population genetic analyses, only the newly added mitochondrial haplogroup identification and the nuclear DNA analysis of the Covarón samples could be meaningfully affected by modern human contamination. We explicitly note this possibility in the Supplementary Information Section 13 describing the newly added mtDNA haplogroup analysis. For the nuclear DNA analysis, which was successful only for the Covarón samples, the placement of these samples in the PCA alongside western hunter-gatherers and outside the genetic variation of present-day humans strongly indicates that contamination removal was effective.

Regarding sex determination of the human DNA, we were careful, at least for assignments leaning toward female X/autosome ratios, to describe them as “predominantly” female. We have now added the same wording for assignments leaning toward male, where it was previously missing. Given the wide confidence intervals of the X/autosome coverage ratios, it cannot be excluded for any of the samples that they represent mixtures of multiple individuals, potentially including individuals of both sexes. High-coverage mtDNA genomes would be required to determine whether internally consistent mtDNA sequences support the presence of a single individual. This strategy has been applied in Vernot et al. (2021), and also in Zavala et al. (2021, Nature 595, 399–403), but cannot be implemented here due to the limited data available. We would also like to note that Figure 4b in Vernot et al. (2021) shows that although sediments can contain mixtures of human DNA from individuals of different sexes, these often appear skewed towards one sex (not fully balanced). In addition, in about half of the cases presented in that study, DNA could be assigned to predominantly one sex, and in several instances to presumably a single individual. The sex biases observed in the samples containing faunal DNA, which we speculate originate from sediment transfer to the cave wall, are therefore fully compatible with previous findings from sediment DNA studies.

We appreciate the references to previous studies on sexual division of labour in pigment processing. These will be very valuable to consider in future work if clear links can be established between cave art samples and DNA from the individuals involved in their production. In the present study, the only DNA sample for which such a link might exist (SP.B.2674) yielded data that are too sparse to determine whether it originates from a male, female or mixed source.

Perhaps a certain contradiction (or need for clarification) is evident regarding the interpretation of fauna. The authors argue that the presence of felids, leporids, and herbivores indicates sediments, but the identified species (bovids, cervids) were also used as pigment sources (bone marrow), binders (fat), and tools (bones).
Perhaps the metagenomic analysis is underexplored. The shotgun analysis reveals bat predominance, but the authors do not discuss possible implications for DNA preservation (bats defecate/urinate on walls, creating microenvironments that could accelerate DNA degradation...). The microbial context is not explored in depth either.
To give more substance to the methodology employed, perhaps a parallel experimental analysis could be provided to validate (through simulated deposition experimentation) how much DNA is deposited when touching, spitting, painting, etc. on walls? These data could serve as a framework to calibrate expectations.

This comment made us realize an important omission in the main text, namely that all non-bat faunal species (*Bovidae*, *Cricetidae*, *Felidae*, *Muridae*, and *Mustelidae*) were found exclusively in unpigmented samples in the metagenomic analysis. Thus, our data provide no evidence for the use of animal products in pigment preparation. We have now clarified this in the text.

To test the hypothesis that bat faeces might contribute to DNA degradation, we performed an additional analysis of the metagenomic data in which we stratified deamination rates across all detected taxa according to whether they were found in samples that also contained bat DNA, used here as a proxy for the presence of bat faeces. While bat DNA itself showed a tendency toward higher deamination rates, no such effect was observed for non-bat taxa co-occurring with bat DNA. As this analysis did not support a direct effect of bat faeces on overall DNA preservation and addressed a very specific hypothesis, we chose to not include it in the manuscript.

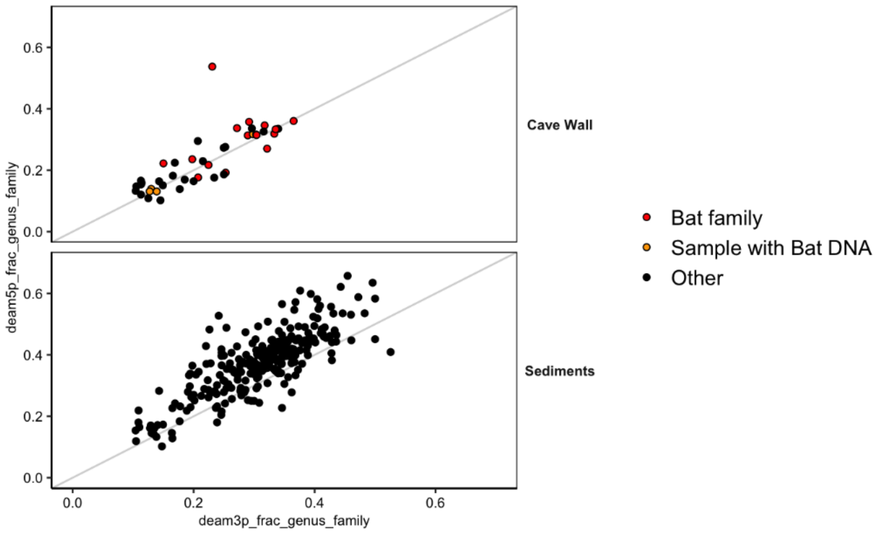

For the metagenomic analyses, we focused on plant and animal DNA, corresponding to sources that could have been deliberately used in pigment preparation. We did not investigate microbial profiles, as this would require a separate and complex analytical framework and would not directly address a specific hypothesis of the present study. However, all genetic data will be released together with the

publication and will be available to other researchers, including those with expertise in microbiology, who may wish to analyse the data further.

Finally, we agree that experimental work quantifying the amount of DNA deposited by humans through different types of contact with cave walls would be highly informative. Such experiments, however, are beyond the scope of the present study and would constitute a substantial follow-up research project, motivated by the findings reported here. We are currently considering such a study, but it would require at least one to two years to conduct properly in order to capture seasonal variation in temperature fluctuation and water flow along cave walls, to allow time for post-depositional DNA degradation processes to occur, and to obtain all necessary permits for this research.

Regarding temporality, there may be some limitation that should be considered and qualified in the conclusion. The authors claim that the DNA is "several thousand years old" based only on pronounced degradation (deamination) and the sealing of Escoural until ±6,000 BP. The problem could lie in the fact that DNA degradation could be extremely variable depending on the microenvironment, and this should be specifically considered in the case study. Perhaps by applying DNA degradation modeling (thermal age modeling; Smith et al., 2003, Proc. R. Soc. B 270:939-945) and/or integrating empirically measured cave temperature/humidity.
Perhaps some works of similar themes could be reviewed/referenced:
Baales et al. 2017. Quat. Int. on DNA recovery from lithic tools. Directly relevant for discussing methodological alternatives.
Willerslev et al. 2003. Science 300:791-795 on environmental DNA in sediments. Pioneering in showing that DNA can persist without tissues, but emphasizes that the source is ambiguous (critical for authorship interpretations).

In an attempt to further constrain the age of the human DNA recovered from the cave wall samples, we further investigated deamination patterns as suggested. It has been shown that deamination increases both with sample age and temperature, but to our knowledge no dataset exists that directly correlates deamination patterns inferred from high-throughput sequencing data with thermal age. We therefore took the dataset of 185 ancient samples compiled by Kistler et al. 2017 (NAR 45:6310-20), which summarized damage parameters in samples ranging from hundreds to tens of thousands of years in age, filtered out the permafrost samples and plotted a correlation between deamination rates in single-stranded overhangs (as inferred by mapDamage 2.0) and sample age. We then determined the same parameter for our cave wall samples and marked where they fall in the plot (Supplementary Figure 23).

As expected, the predictive power for inferring sample age from deamination rates is low due to the large variation in deamination rates in samples from similar ages. However, assuming that deamination does not proceed vastly faster on cave walls – in sediments, where DNA binding also involves mineral surfaces, they seem to be broadly compatible with those observed in bones – this analysis excludes ages below 2,000 years for the human DNA recovered from all cave walls samples except one sample from Covarón (minimum age ~1,000 years).

At Covarón, we additionally compared deamination rates between the cave wall samples and sediment samples from the site, which are broadly comparable. At Escoural, we compared deamination rates in the cave wall samples to those observed in a bovid bone from a Middle Palaeolithic deposit from the site, which likely formed around 49 ka (Alzate-Casallas et al., 2025). In this comparison, the deamination rates are lower in the human DNA from the cave wall (approximately half, but with large confidence intervals; see newly added Supplementary Figure 24). This suggests that the human DNA is younger than the bovid bone. If (reluctantly) assuming a linear relationship between deamination signals and age, a best guess for the age of the human DNA would be around 20-30 ka, which would correspond to an Upper Palaeolithic origin.

In a further attempt to constrain the ages of the human DNA recovered from the cave wall samples, we also performed mitochondrial haplogroup assignment (Supplementary Information Section 13). While this analysis suggests haplogroup U5a'b for the two Covarón samples, which is consistent with their nuclear genetic affinity to Western hunter-gatherers, it did not provide sufficient resolution to meaningfully constrain the age of the Escoural samples.

Overall, when combining genetic and archaeological evidence, the age of the human DNA from the Escoural cave wall samples is at least 5,000 years, with a Pleistocene age being entirely possible. The Covarón cave wall samples are constrained to between approximately 5,000 and 17,000 years based on their nuclear genetic affinity to previously sequenced ancient individuals.

We have added a stand-alone section summarizing these results to the Results section.

Reviewer #2 (Remarks to the Author):

This manuscript explores whether Palaeolithic rock art surfaces can preserve ancient DNA, extending genetic inquiry beyond traditional archaeological sediments and artefacts. By analysing samples from rock art panels across 11 Iberian caves, the authors show that ancient DNA can indeed persist on cave walls, both in pigmented areas and in unpigmented sections. At Escoural Cave, human-only DNA recovered from a pigmented calcite crust and an adjacent unpigmented wall sample is interpreted as reflecting direct human contact, while other unpigmented samples containing mixed human-faunal DNA are interpreted as reflecting indirect deposition. Although establishing a direct link between DNA and the creators of the art remains challenging, this study provides compelling evidence for the potential of cave wall surfaces to retain ancient genetic material. We found this an innovative and well-crafted study and recommend publication. However, we do have some general comments that could help strengthen the archaeological integration and interpretive clarity of the manuscript.

We thank the reviewer for their positive and constructive evaluation of the manuscript. Our responses to the specific points that were raised are provided below.

1. Integration with archaeological data: The manuscript would benefit from a fuller integration of the archaeological context of the sampled sedimentary layers, art panels and sites. Although the methodology is very attractive, the fact that DNA seems to be recorded in cave walls independently

of art raises the broader question of: What did we learn from this study in terms of cave art and associated/nearby archaeology?
Independently of the positive or negative results, however, the manuscript would gain by incorporating more detailed information on the stratigraphic setting of sampled archaeological sediments, age estimates for both the artworks (radiometric, when available, but at least stylistic correlations) and sedimentary layers, as well as archaeological content (if any) of the sedimentary layers sampled (in the SI if not in the main manuscript).
This is particularly important for the samples from Covarón, where ancient hominin DNA has been recovered and where the genetic dating spans a very broad range (~16,700–5,200 cal BP) (Lines 275-276) –is there some data on the stylistic affinity of the cave art that could help further constrain this period?

We have expanded the Results section to include a new section on temporal constraints for the ancient human DNA recovered from the cave walls. This section incorporates additional analyses of deamination patterns, attempts mitochondrial haplogroup assignments and briefly summarizes the archaeological contexts of Escoural and Covarón while providing references for further reading. As suggested by the reviewer, we also discuss the possible ages of the cave art at Escoural and Covarón based on their styles, which are consistent with an Upper Palaeolithic attribution while not excluding younger ages. In addition, we now provide brief summaries of the occupational histories of both sites, together with references to published work describing the archaeological assemblages, sedimentary contexts, and cave art in greater detail.

At Covarón, human DNA was recovered exclusively from unpigmented samples, and it is likely that this DNA derives from sediment that was transferred from the cave floor to the walls. Consequently, we cannot establish new links between the cave art and the archaeological record at this site. Similarly, at Escoural, although a link between the creation of cave art and the human DNA recovered from one of the pigmented samples (SP.B.2674) is possible, the amount of genetic data retrieved from this sample is insufficient to determine the sex of the individual(s), mitochondrial haplogroups, or nuclear genetic affinities. Nonetheless, the demonstration that ancient human DNA can be preserved on cave walls, even in the absence of clear links to cave art, opens new opportunities for future research, as discussed in the final paragraph of the substantially revised Discussion section.

2. Issues of association between sampled materials and the paintings:
While in this study the interpretive consequences are limited because (correctly) no strong associations are claimed, it is important to acknowledge that both unpigmented cave walls, calcite layers overlying pigments and the sampled sediments may have substantially different ages than the paintings (see, for instance, Hoffmann et al. 2018, Science). These temporal offsets should be explicitly addressed, as it has implications for interpreting any recovered aDNA. In other words, if DNA is just randomly preserved in cave walls, how should one go about interpreting it if, in the future, a positive (and potentially unexpected) result is found in association with a pigmented area?

We agree that the manuscript lacked a discussion on how associations between rock art and recovered DNA could be established. We have therefore added a section to the Discussion addressing this issue, which now reads: “Establishing a direct link between DNA and art production

would require evidence that the DNA in the pigment sample consistently differs from that in unpigmented areas, whether in its abundance, the likelihood of successful recovery, or its population genetic affinities to past human groups. Alternatively, when pigment is trapped beneath carbonate crusts, the pigmented areas as well as overlying and potentially underlying unpigmented parts can be sampled and analysed separately to determine whether DNA recovery is consistently restricted to the pigmented portions, as we have attempted here for the Escoural crust. This approach is also commonly used in uranium-thorium dating of rock art,[9, 20,54] which can in principle be combined with DNA analysis. If sufficient human DNA from a single individual were obtained in future studies, genetic dating may also become possible, as has been demonstrated for DNA recovered from sediments.[5,35]".

3. Sampling strategy: Building up on this, there is the issue of how to sample these challenging contexts. Though I acknowledge that important preservation constrains exist when sampling the rare and unique rock art panels and sampling strategies may vary from site-to-site, it was nonetheless unclear to me what was the sampling methodology and why this strategy was not made more standardized across different sites and/or panels within sites. If possible, would it not be relevant, for instance, to sample consistently from the calcite above the pigment (when present), the pigmented areas, below the pigmented areas and then sampling laterally adjacent and away from the paintings (see examples of this in Hoffmann et al 2016 Quaternary Geochronology)? Right now, each site has substantial different sampling protocols that are in no way standardized.

We agree that, ideally, a fully standardized protocol across sites and panels would be desirable. In practice, however, the sampling of rock art presents unique challenges that differ fundamentally from other types of material. As illustrated in Figure 2, pigment occurs in highly variable contexts: directly on the wall, beneath or within carbonate protuberances, under soft mineral layers, in fissures, as flakes that have fallen to the ground, etc. These differences, combined with conservation concerns, required us to select sampling strategies on a case-by-case basis to minimize the visual and structural impact of the sampling. Furthermore, restrictions on the amount and location of material that could be removed differed between sites for both the pigmented samples and the unpigmented controls, due to conservation regulations and site management. To clarify this for the reader, we have added the following sentence to the Methods section: "Sampling strategies were adapted for each site and panel to account for the variable contexts in which pigments occurred, while minimizing visual impact on the rock art and adhering to site-specific restrictions defined by the permit-granting authorities."

We would also like to emphasize that, when we began this study, we were exploring new territory. Naturally, our results now inform, in hindsight and for future work, what more optimal sampling strategies might have been. For instance, when designing the sampling strategy, we did not anticipate recovering ancient human DNA outside pigmented areas. We also did not expect the limited amounts obtained from the pigmented samples. In fact, we had hoped for a clearer contrast between pigmented samples and controls, which would have allowed more direct interpretations.

4. Interpretation of wall-sample positivity as contamination from cave-floor sediments:
The manuscript currently states that positive samples with co-occurrence of faunal and human DNA from the walls may reflect contamination originating from cave-floor sediments (e.g., Lines 227-228,

231-232, 323-325). However, the mechanisms by which sediments from the cave floor below could contaminate the walls located stratigraphically above are unclear and left unexplained. Would not alternative pathways – such as water percolation, dissolution and transport of DNA through walls and ceilings, infiltration from overlying soils, or genetic traces left by later cave occupants – better explain these results? Similar processes have been proposed to account for DNA found in speleothems where no sediment floor contamination is expected – and only mentioned in Lines 326-327. I would urge the authors to address these mechanisms of sources and transfers of DNA in caves, and consider citing work such as Brown et al. 2025, JAS that discuss just this.

We agree that these aspects were not clearly enough addressed in the previous version of the manuscript. We have now revised the Discussion to more explicitly highlight the contrast with findings from previous studies of sediments and stalagmites, which suggest that the exclusive deposition of human DNA, in the absence of other faunal DNA, is a highly unusual finding and is not consistent with indirect modes of DNA deposition, such as water percolation. However, for samples in which both faunal and human DNA were recovered, indirect deposition remains a plausible explanation.

The revised section also more explicitly describes how the indirect deposition of DNA could have taken place. The relevant passage now reads: "Two cave wall samples from Escoural (one pigmented and one unpigmented) contained ancient human but no detectable faunal DNA. This contrasts with findings from multiple studies,[4,5,35,36,52] which have shown that when human DNA is present in sediments, it is almost always mixed with faunal DNA, indicating that other mammalian species generally contribute more DNA to the environment than humans. Similarly, ancient faunal, but not human DNA, is also the only type of mammalian DNA recovered from stalagmites, which have been shown to incorporate environmental DNA during mineral crust formation.[54] The exclusive presence of human DNA among the mammalian DNA identified in the two Escoural cave wall samples therefore points to direct human contact (such as touching, rubbing, or deposition of bodily fluids) as the most likely source of DNA deposition on the cave wall. In contrast, in three additional unpigmented cave wall samples, two from Covarón and one from Escoural, human DNA co-occurred with faunal DNA, consistent with indirect deposition. This may have occurred via sediment transfer (for example through people touching the ground and subsequently the cave walls as they moved through the cave) or through other mechanisms such as movement of DNA in percolating water.[54]".

Overall, these considerations raise broader questions: to what extent might any DNA detected on cave walls be influenced by such processes, and how can future studies robustly associate hominin DNA from pigmented areas with the individuals responsible for creating the art? Expanding the discussion of these issues would significantly strengthen the manuscript.

To address these questions more specifically, the revised Discussion now includes a paragraph dedicated to the types of evidence that would be required to establish links between DNA recovered from cave walls and the production of cave art.

Other comments

• Although the extraction method proposed by the authors was tested on DNA-spiked clay and lime,

can the author expand on how this extraction protocol works on “real” sediment where the binding mechanisms of DNA in soils/sediments is more complex than in DNA-spiked minerals? Also, did they see any effect of the initial mass of the sample affecting DNA extraction efficiency (e.g., Capo et al., 2021)?

As part of an ongoing study, we are further exploring DNA extraction methods for sediments, including investigations of DNA binding and release using real sediment samples. Preliminary results from these experiments suggest that the EDTA-containing extraction buffer used in the Dabney method is highly effective at disrupting mineral–DNA interactions. In future work, we plan to extend these experiments to the cave wall control samples collected in the present study to assess whether further improvements in DNA recovery from these mineral surfaces are possible. We now hint at this in the Discussion: "It should also be investigated whether DNA extraction protocols can be improved specifically for cave wall surfaces."

When working with sediments, we observe good correlations between sediment input mass and DNA yield in some cases. In other cases however, no linear input–output relationship is observed. We attribute this primarily to the co-extraction of inhibitors that interfere with library preparation, rather than to inefficient DNA recovery during extraction itself. These effects can be quantified using a synthetic spike-in, as shown in Table S1, column S (“Efficiency Library Preparation”). Notably, several sediment samples in our study appear highly inhibited, although they still produced meaningful results, whereas such inhibition was not observed for the cave wall samples.

• Was the cave wall surface routinely cleaned or mechanically removed prior to collecting samples for aDNA analysis?

Our priority while sampling was to minimize the impact upon the cave walls, and specially the cave art. The additional act of cleaning could have increased the chance of producing alteration of adjacent areas of the panels. Furthermore, because many of the pigments were directly exposed on the cave wall, cleaning would not have been possible in any case.

Nevertheless, when choices were available, we preferentially sampled areas that were out of arm’s reach from the current level of the cave floor or that are hidden today and are therefore unlikely to have attracted attention by recent visitors.

• Fig.4: Consider using distinct colors in the top histogram to differentiate the sites and improve readability.

Thank you for this suggestion. The figure did indeed need improvement. We have decided against using colours in the top panel to avoid confusion with the colouring of the mammalian families. Instead, we have added frames and background patterning to differentiate the various sites and sample types and to visually link results from the same samples.

• It was encouraging to see that two hand stencils, that involve direct hand contacts to the cave walls, were sampled at Maltravieso, even though they did not yield aDNA, consistent with the lack of

results from skeletal material at the site. Were additional hand stencils available for sampling, and if so, what was the rationale for not selecting them more extensively?

We agree that sampling additional hand stencils would be valuable, particularly given that DNA preservation conditions at Maltravieso are not optimal. We have added this as a suggestion for future work in the Discussion: “For example, it would be desirable to test DNA preservation in additional samples from figurative rock art, or in hand stencils from sites with better DNA preservation than Maltravieso.” Unfortunately, for the present study, we did not have permission to sample hand stencils at other sites.

• Please specify “flakes” (e.g., Lines 146-147) - do you mean flakes of limestone?

These flakes contained a thin layer of mineral in addition to the pigment. We had previously specified this only in the methods section (“flakes of pigment-containing minerals”), but have now also changed the main text accordingly (“pigment-containing mineral flakes”).

Reviewer #3 (Remarks to the Author):

I co-reviewed this manuscript with one of the reviewers who provided the listed reports. This is part of the Nature Communications initiative to facilitate training in peer review and to provide appropriate recognition for Early Career Researchers who co-review manuscripts.

We thank the reviewer for their assistance in reviewing this manuscript.

Reviewer #4 (Remarks to the Author):

The manuscript “Investigating ancient human DNA preservation on cave walls and in rock art” addresses a question that many, primarily archaeologists, have been asking for decades: is it possible to detect genetic traces of the individuals who created rock art within the pigments they used? The study is meticulously planned, and the analytical procedures are clearly described and carried out to the highest standards of the ancient DNA field.

To test whether human DNA can be recovered from rock art, the authors designed an appropriate and comprehensive sampling and analytical strategy. However, despite this well-executed approach, the results show that more human DNA was obtained from sites or samples without pigmentation than from the pigment itself. This suggests that the answer to the central question is, unfortunately, negative: we currently cannot recover meaningful human aDNA directly from rock art pigments. This raises a challenge, this is a well, designed and valuable study, but its primary outcome is a negative result. How should this be contextualized and interpreted?

We thank Reviewer #4 for carefully evaluating our manuscript and providing comments and suggestions.

We agree that, with respect to its initial primary objective - recovering DNA from rock art to identify its makers - the results are largely negative, although a possible connection between the ancient

human DNA recovered from the Escoural sample and the maker(s) of the art cannot be excluded. At the same time, the question of whether DNA can be recovered from rock art has long been discussed, and we expect that many researchers will be interested in the first systematic evaluation of this possibility. Importantly, and unexpectedly, our results demonstrate that ancient human DNA can be preserved on cave walls, opening new avenues of research that have not previously been explored. We have reworked the Discussion section to more clearly highlight both the negative and positive aspects of these findings and their implications.

I have some comments:
The title is somewhat misleading. The study does not recover ancient human DNA from the rock art itself, but from areas adjacent to it. This distinction should be made clearer.

We agree that the title, in its brevity, could suggest that ancient human DNA recovered from the rock art sample is unambiguously linked to the art. We have therefore changed the title to "Investigating ancient human DNA preservation on cave walls and in rock art".

The human DNA identified cannot be securely dated. While the damage patterns are consistent with ancient DNA, this alone is not sufficient evidence for chronological attribution in this context. A discussion of this limitation would strengthen the manuscript.

We have added a section to the Results that directly addresses the question of the potential age of the human DNA sequences recovered from the cave wall samples. This provides additional evidence that the DNA is at least several thousand years old, while acknowledging that it cannot be dated more precisely. In the revised Discussion, we have added a paragraph outlining the types of evidence that would be required in future studies to establish a stronger link between ancient DNA and the producers of cave art.

It would be beneficial to include a discussion of the preservation potential of DNA in rock art and cave wall matrices. What minerals or substrates are present? Sedimentary aDNA studies often address this to help readers understand why DNA is or isn't preserved, and a similar treatment here would improve interpretability.

Thank you for pointing out that interactions between DNA and mineral surfaces were not discussed in the original manuscript. We have now addressed this in the Introduction by adding: "Given the various potential techniques used to apply pigments to cave walls[13] and the ability of DNA to bind to mineral surfaces,[4,14] it is tempting to speculate that rock art might preserve DNA from its creators." As no chemical analyses were performed to determine the composition of the sampled mineral surfaces, we cannot further correlate our results with substrate-specific differences, but we agree that this could be a topic of interest for follow-up studies.

Methodological concern (lines 478–479)
The manuscript notes that some samples were embedded in resin and hardened at 60°C for 2–7 days. This seems like potentially harsh treatment for ancient DNA. A clarification of how this process affects DNA integrity, or why it does not, would be valuable.

The Massilani et al. 2022 study, which we cite in this context, explicitly tested the effect of resin impregnation, including the high-temperature treatment, on DNA retrieval and found no detrimental effects. We have now clarified in the Methods that impregnation using this validated procedure does not interfere with ancient DNA recovery.

Figure 3
This figure is confusing. The caption states that it shows “before and after” sampling, but most of the images appear to show only the post-sampling state. Either the figure or the caption should be clarified.

Due to space constraints, we selected the images that seemed most informative for documenting the exact locations of the samples. We have now corrected the figure legend to “The images on the right show close-up views taken before and/or after sampling.”

Figure 4
In the top panel of Figure 4, the meaning of the colour black is unclear. Additional explanation in the caption or legend is needed.

We have removed the black colour to avoid confusing with the colouring of the mammalian families and made additional revisions suggested by reviewer #2 to improve clarity.

Reviewer #5 (Remarks to the Author):

After all the previous success stories of obtaining authentic DNA not only from bones of archaic and modern humans from different, including early times, but more recently also from sediments and artifacts, an aDNA study on rock art was long awaited, and with this study is finally delivered in highest quality. While identifying the artist via aDNA analysis is impossible, and not expected in such scenario because DNA-based identification works in a strictly comparative way and always requires a reference sample of known identity, this is the first study demonstrating that aDNA, human and non-human, can be obtained from rock art. Moreover, detecting variation in detecting human DNA with and without animal DNA may suggest differences in ancient painting techniques. Overall, this is a long-awaited, well-executed, comprehensive study that includes samples from different caves and employs the appropriate study design with well-controlled sampling from pigmented and neighbouring unpigmented areas, additionally including sediment-based and bone-based aDNA analysis, and executed via state-of-the-art sample preparation and DNA analysis as well as data analyses methods. It sets the scientific and technical standards for future rock art studies at other sites. Moreover, this study provides strong motivation for authorities holding responsibility for caves with rock art to eventually allow more aDNA research on more rock art paintings in different countries. This manuscript clearly deserves publication in a highly respected scientific journal, such as this one, while personally, I would have expected this work to appear in one of the highest respected journals.

We thank the reviewer for the enthusiasm and the supportive comments.
